# A Narrative Review on Strategies for the Reversion of Prediabetes to Normoglycemia: Food Pyramid, Physical Activity, and Self-Monitoring Innovative Glucose Devices

**DOI:** 10.3390/nu15234943

**Published:** 2023-11-28

**Authors:** Mariangela Rondanelli, Gaetan Claude Barrile, Alessandro Cavioni, Paolo Donati, Elisa Genovese, Francesca Mansueto, Giuseppe Mazzola, Zaira Patelli, Martina Pirola, Claudia Razza, Stefano Russano, Claudia Sivieri, Alice Tartara, Eugenio Marzio Valentini, Simone Perna

**Affiliations:** 1 Department of Public Health, Experimental and Forensic Medicine, University of Pavia, 27100 Pavia, Italy; gaetanclaude.barrile01@universitadipavia.it (G.C.B.); alessandro.cavioni01@universitadipavia.it (A.C.); elisa.genovese01@universitadipavia.it (E.G.); francesca.mansueto01@universitadipavia.it (F.M.); giuseppe.mazzola02@universitadipavia.it (G.M.); zaira.patelli01@universitadipavia.it (Z.P.); martina.pirola03@universitadipavia.it (M.P.); claudia.razza01@universitadipavia.it (C.R.); claudia.sivieri01@universitadipavia.it (C.S.); alice.tartara01@universitadipavia.it (A.T.); eugeniomarzio.valentini01@universitadipavia.it (E.M.V.); 2 AICUBE srl, 20090 Trezzano sul Naviglio, Italy; paolo.donati@theaicube.com (P.D.); stefano.russano@theaicube.com (S.R.); 3 Department of Food, Environmental and Nutritional Sciences, Division of Human Nutrition, University of Milan, 20133 Milan, Italy; simoneperna@hotmail.it

**Keywords:** prediabetes, impaired fasting glycemia, food pyramid, glucose monitoring system

## Abstract

In 2019, “Nutrition Therapy for Adults with Diabetes or Prediabetes: A Consensus Report” was published. This consensus report, however, did not provide an easy way to illustrate to subjects with prediabetes (SwPs) how to follow a correct dietary approach. The purpose of this review is to evaluate current evidence on optimum dietary treatment of SwPs and to provide a food pyramid for this population. The pyramid built shows that everyday consumption should consist of: whole-grain bread or potatoes eaten with their skins (for fiber and magnesium) and low glycemic index carbohydrates (GI < 55%) (three portions); fruit and vegetables (5 portions), in particular, green leafy vegetables (for fiber, magnesium, and polyphenols); EVO oil (almost 8 g); nuts (30 g, in particular, pistachios and almonds); three portions of dairy products (milk/yogurt: 300–400 g/day); mineral water (almost 1, 5 L/day for calcium intake); one glass of wine (125 mL); and three cups of coffee. Weekly portions should include fish (four portions), white meat (two portions), protein plant-based food (four portions), eggs (egg portions), and red/processed meats (once/week). At the top of the pyramid, there are two pennants: a green one means that SwPs need some personalized supplementation (if daily requirements cannot be satisfied through diet, vitamin D, omega-3, and vitamin B supplements), and a red one means there are some foods and factors that are banned (simple sugar, refined carbohydrates, and a sedentary lifestyle). Three to four times a week of aerobic and resistance exercises must be performed for 30–40 min. Finally, self-monitoring innovative salivary glucose devices could contribute to the reversion of prediabetes to normoglycemia.

## 1. Introduction

In light of the increase in the incidence and prevalence of Type 2 diabetes mellitus (T2DM) worldwide, numerous studies have focused on the early identification of individuals at risk of developing this disease, so as to be able to implement interventional strategies to delay the onset. This has led to the recognition of the term “prediabetes”, a dysglycemic state straddling normal glucose tolerance and the frank diagnosis of T2DM [1]. “Prediabetes”, in fact, is a term used to indicate a condition in which blood glucose levels are suggestive of abnormal glucose metabolism, but are not yet able to meet the diagnostic criteria of full-blown diabetic disease [2]. The definitions of prediabetes most frequently used are those of the American Diabetes Association (ADA) and the International Committee of Experts and the World Health Organization (WHO). Both definitions are based on measurements of fasting glucose concentrations, two hours after the oral glucose challenge, and/or glycated hemoglobin (HbA1c) values. The latter has only recently been included in the definition. However, there has been discussion of how useful this is in clinical practice [3,4] since HbA1c does not always seem to be able to identify all subjects with prediabetes and the only way to identify the alteration of glucose metabolism is through the dosage of fasting blood glucose and through the oral glucose challenge test [5].

A systematic review that considered 16 cohort studies for a total of 44,203 subjects observed over 5–6 years concluded that, as HbA1c increased, the risk of developing T2DM significantly and progressively increased: the risk increases from 9 to 25% in 5 years with a hemoglobin glycosy between 5.5% and 6.0% (37–42 mmol/mol), and 25 to 50% with a hemoglobin glycosy between 6.0% and 6.5% (42–48 mmol/mol), with a relative risk 20 times greater than those who had a HbA1c level of 5% [6].

Additional studies suggest that a HbA1c of 5.7% (39 mmol/mol) or greater is associated with a risk of T2DM similar to individuals who participated in the Diabetes Prevention Program (DPP) and were defined as high-risk, and their baseline HbA1c was a strong predictor for the development of T2DM during DPP and its follow-up [7,8].

Therefore, it is reasonable to consider that an HbA1c between 5.7 and 6.4% (39–47 mmol/mol) could identify individuals with prediabetes.

Muscle IR, considered a trigger of T2DM, may have been present for decades before the beta-cell function is impaired [9]. Furthermore, it has been reported that there is a significant increase in beta-cell function 3 to 4 years before the diagnosis of T2DM, followed by a large decrease thereafter [10]. The continuous increase in IR accompanied by a reduction in beta cell functionality leads to abnormal blood sugar levels, such that prediabetes can evolve into full-blown T2DM.

Given these premises, prediabetes should not be seen as a clinical entity in and of itself, but more as a condition of increased risk for both T2DM and cardiovascular disease. In fact, prediabetes is also associated with obesity (particularly visceral obesity), dyslipidemia (with high triglycerides and low HDL cholesterol), and hypertension. Therefore, the presence of this condition should serve as input to initiate a comprehensive screening of all cardiovascular risk factors [2].

The prevalence of prediabetes obviously depends on the age group of the population studied, with the elderly having a higher prevalence, as well as on the definition used, with a higher prevalence when using the ADA criteria as a result of the difference in cut-off values used against WHO criteria [11].

According to recent studies, the rate of progression from prediabetes to T2DM is about 25% in 3–5 years and it is estimated that 70% of individuals with prediabetes will develop T2DM in their lifetime [10,12,13,14,15,16,17].

If left untreated, 37% of people with prediabetes could develop T2DM in 4 years [18]. However, where appropriate lifestyle changes are made, long-term studies have shown that the risk of progression can be reduced by delaying the onset of T2DM to 10 years, avoiding long-term complications related to T2DM [18].

In this sense, lifestyle interventions appear to be very promising [18].

Given this background, even if in 2019, the “Nutrition Therapy for Adults with Diabetes or Prediabetes: A Consensus Report” was published, [19]. This consensus report does not provide an easy way to illustrate to subjects with prediabetes (SwPs) how to follow the correct dietary approach. The purpose of this review is to evaluate current evidence on optimum dietary treatment of SwPs and to provide a food pyramid for this population.

## 2. Materials and Methods

**Figure 1:**
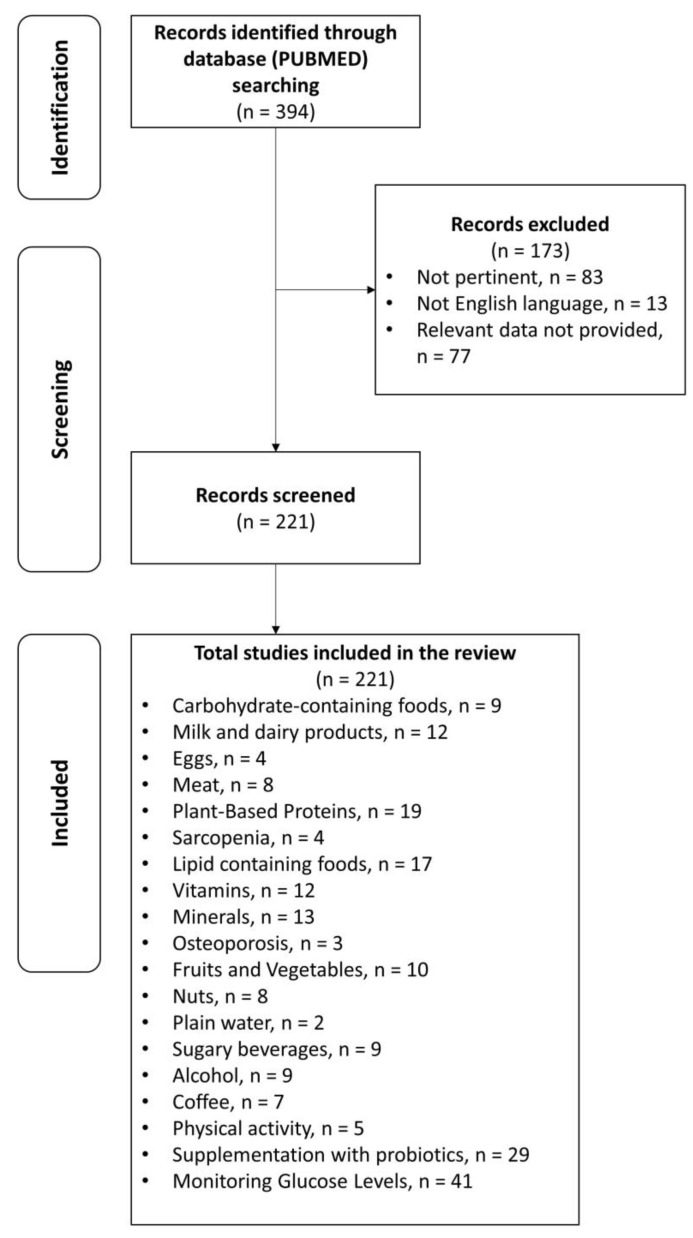
Flow chart of the eligible studies.

**Figure 2:**
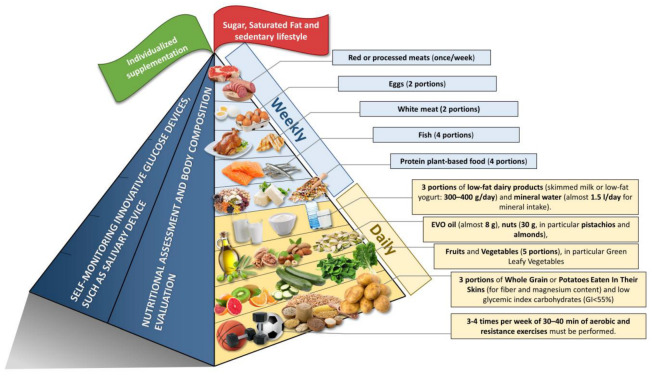
Ideal food pyramid for prediabetes.

This narrative review was performed following these steps [20,21,22]:

(1) Configuration of a working group: three operators skilled in clinical nutrition (one acting as a methodological operator and two participating as clinical operators); (2) formulation of the revision question on the basis of considerations made in the abstract: “the state of the art on ideal dietary therapy in order to obtain the reversion of prediabetes to normoglycemia”; (3) identification of relevant studies: a research strategy was planned on PubMed (Public MEDLINE run by the National Center of Biotechnology Information (NCBI) of the National Library of Medicine of Bethesda (Bethesda, MD, USA)) as follows: (a) definition of the keywords (prediabetes, IFG, IGT, foods, nutrients, and dietary supplements), allowing the definition of the interest field of the documents to be searched, grouped in inverted commas (“. . .”), and used separately or in combination; (b) use of the Boolean (a data type with only two possible values: true or false) AND operator, which allows the establishment of logical relations among concepts; (c) research modalities: advanced search; (d) limits: time limits: papers published in the last 30 years; humans; languages: English; (e) manual search performed by senior researchers experienced in clinical nutrition through the revision of reviews and individual articles on ideal dietary therapy in order to the state of the art on ideal dietary therapy in order to obtain the reversion of prediabetes to normoglycemia published in journals qualified in the Index Medicus; (4) analysis and presentation of the outcomes: the data extrapolated from the “revised studies” were collocated in tables; in particular, for each study specified, the author and year of publication and the study characteristics; for each topic, we built three types of tables depending on the type of study: table with reviews and meta-analyses, table with observational human studies, and table with interventional human studies; in the tables (obviously except for reviews and meta-analyses), only studies on humans are reported, while, in the text, in vitro studies and studies on animal models are also cited, if useful to explain some mechanisms of action. Moreover, in tables, for all studies, the level of evidence has been added [23,24]; (5) The analysis was carried out in the form of a narrative review of the reports. At the beginning of each section, the keywords considered and the kind of studies chosen have been reported. We evaluated, as suitable for the narrative review, the studies of any design that considered the relevance of diet, foods, nutrients, and dietary supplements in order to obtain the reversion of prediabetes to normoglycemia. Figure 1 shows the eligible studies and Figure 2 represents proper nutrition and lifestyle to prevent or support the treatment of prediabetes, specifying the quality and amount of food needed to provide ideal dietary management and to construct a food pyramid.

## 3. Results

### 3.1. Carbohydrate-Containing Food

This research was conducted based on the keywords: “prediabetes” OR “diet” OR “diet and prediabetes” OR “carbohydrate” OR “Mediterranean diet” OR “ketogenic diet”. Nine studies have been referenced, including: one consensus statement, one consensus report, one cohort study, one prospective study, one randomized dose response trial, one randomized controlled trial, one randomized pilot trial, one randomized crossover trial, and one prospective observational study.

Table 1 shows the studies that evaluated the relationship between carbohydrate-containing foods and prediabetes with their strength of evidence.

### 3.2. Protein Foods

#### 3.2.1. Milk and Dairy Products

This research was conducted based on the keywords: “milk consumption” OR “dairy consumption” OR “cheese” OR “yogurt” AND “risk of type 2 diabetes” OR “prediabetes” OR “glycemic control” OR “risk for developing diabetes”. The following studies were included: two randomized controlled trial, one systematic review, three prospective cohort studies, two cross-sectional studies, one retrospective cohort study, two narrative review, and one experimental study. Table 2A,B shows the studies that evaluated the relationship between milk/dairy products and prediabetes with their strength of evidence.

#### 3.2.2. Eggs

This research was conducted based on the keywords: “eggs consumption” OR “eggs” AND “risk of type 2 diabetes” OR “prediabetes” OR “glycemic control” OR “risk for developing diabetes”. The following studies were included: one randomized controlled trial, one systematic review, one meta-analysis, and one report from USDA.

Table 2C,D shows the studies that evaluated the relationship between eggs and prediabetes with their strength of evidence.

#### 3.2.3. Meat

This research was conducted based on the keywords: “red meat” OR “read meat consumption” OR “meat” AND “prediabetes” OR “risk of type 2 diabetes” OR “glycemic control” OR “risk for developing diabetes”. The following studies were included: one cross-sectional study, two meta-analyses combined with three prospective cohort studies, one prospective study, and one systematic review.

Table 2E,F shows the studies that evaluated the relationship between meat and prediabetes with their strength of evidence.

#### 3.2.4. Plant Based Protein

This research was conducted based on the keywords: “legumes” OR “soy proteins” OR “soy”, “peanuts” OR “vegetal protein” OR “plant-based proteins” AND “diabetes” OR “prediabetes” OR “impaired fasting plasma glucose” OR “glycemia”. The following studies were included: four meta-analyses, two prospective cohort studies, one systematic review, two randomized controlled trials, one report from the WHO, three narrative reviews, one case–control study, four in vitro studies, and one in vivo animal study.

Table 2G,H shows the studies that evaluated the relationship between plant based protein and prediabetes with their strength of evidence.

### 3.3. Sarcopenia

This research was conducted based on the keywords: “Sarcopenia” OR “muscle loss” AND “prediabetes” OR “diabetes” OR “impaired fasting glucose”. The following studies were included: one cross-sectional study, one meta-analysis, and two retrospective studies.

Table 3A,B shows the studies that evaluated the relationship between sarcopenia and prediabetes with their strength of evidence.

### 3.4. Lipid-Containing Food

This research was conducted based on the keywords: “Lipids AND prediabetes” OR “Lipids AND diabetes” OR “Fatty Acids AND prediabetes “OR “Diabetes prevention AND lipids” OR “Diabetes prevention AND fatty acids” OR “olive oil AND prediabetes” OR “fish AND prediabetes”. In total, 17 articles were consulted, including 4 narrative reviews, 1 systematic review, 2 meta-analyses, and 11 clinical trials (7 randomized controlled trials, 3 cohort studies, and 1 case–control studies).

Table 4A,B shows the studies that evaluated the relationship between lipid-containing food and prediabetes with their strength of evidence.

### 3.5. Vitamins

This research was conducted based on the keywords: “vitamins intake” OR “vitamins supplementation” OR “micronutrient intake” AND “prediabetes” OR “type 2 diabetes” OR “hyperglycemia” OR “diabetes risk”. In total, 12 articles were consulted, including 3 cross-sectional studies, 1 randomized controlled trial, 3 prospective studies, 1 case–control study, 1 systematic review, and 3 meta-analyses.

Table 5A,B shows the studies that evaluated the relationship between Vitamins and prediabetes with their strength of evidence.

### 3.6. Minerals

This research was conducted based on the keywords: “minerals intake” OR “minerals supplementation” OR “micronutrient intake” AND “prediabetes” OR “type 2 diabetes” OR “hyperglycemia” OR “diabetes risk”. In total, 13 articles were consulted, including 3 cross-sectional studies, 2 randomized controlled trials, 3 prospective studies, 1 retrospective study, 3 meta-analyses, and 1 narrative review.

Table 6A,B shows the studies that evaluated the relationship between minerals and prediabetes with their strength of evidence.

### 3.7. Osteoporosis

This research was conducted based on the keywords: “osteoporosis” OR “osteopenia” OR “fracture risk” OR “bone mineral density” AND “prediabetes” OR “type 2 diabetes” OR “hyperglycemia” OR “diabetes risk”. Three articles were consulted: one systematic review, one narrative review, and one cross-sectional multistage study.

Table 7 shows the studies that evaluated the relationship between osteoporosis and prediabetes with their strength of evidence.

### 3.8. Fruits and Vegetables

This research was conducted based on the keywords: “Fruit” OR “vegetables” OR “fiber” AND “prediabetes” OR “type 2 diabetes” OR “hyperglycemia” OR “diabetes risk”. In total, 10 articles were consulted: 1 systematic review, 2 narrative reviews, 1 cross-sectional study, 4 prospective studies, 1 in vivo animal study, and 1 meta-analysis.

Table 8A,B shows the studies that evaluated the relationship between fruits and vegetables and prediabetes with their strength of evidence.

### 3.9. Nuts

This research was conducted based on the keywords: “nuts” OR “almons” OR “nuts consumption” AND “prediabetes” OR “type 2 diabetes” OR “hyperglycemia” OR “diabetes risk”. Eight articles were consulted: two narrative reviews, one cross-sectional study, and five randomized controlled trials.

Table 9 shows the studies that evaluated the relationship between nuts and prediabetes with their strength of evidence.

### 3.10. Hydratation

#### 3.10.1. Plain Water

This research was conducted based on the keywords: “still water” OR “water intake” OR “hydration” AND “prediabetes” OR “diabetes risk” “type 2 diabetes”. Two articles were consulted: two cross-sectional studies.

Table 10A shows the studies that evaluated the relationship between plain water and prediabetes with their strength of evidence.

#### 3.10.2. Sugary Beverages

This research was conducted based on the keywords: “SSB intake” OR “sugary beverage” OR “Sugar sweetened beverage” AND non-sugar sweeteners (NSS), AND “prediabetes” OR “diabetes risk” “type 2 diabetes”. Nine articles were consulted: two randomized controlled trials, one cross-sectional study, one meta-analysis, one systematic review with a meta-analysis of RCT studies, one systematic review, one guideline from the WHO, one standard of care in diabetes, and one narrative review.

Table 10B,C shows the studies that evaluated the relationship between sugary beverages and prediabetes with their strength of evidence.

### 3.11. Alcohol

This research was conducted based on the keywords: “alcohol” OR “ethanol” OR “polyphenol” OR “resveratrol” OR “metabolic syndrome” AND “prediabetes” OR “type 2 diabetes” OR “hyperglycemia” OR “diabetes risk”. Nine studies have been referenced, including: one randomized controlled trial, one systematic review, three narrative reviews, two prospective cohort studies, and two cross-sectional studies.

Table 11A,B shows the studies that evaluated the relationship between alcohol and prediabetes with their strength of evidence.

### 3.12. Coffee

This research was conducted based on the keywords: “coffee” OR “chlorogenic acid” OR “caffeine” AND “prediabetes” OR “type 2 diabetes” OR “hyperglycemia” OR “diabetes risk”. Seven studies have been referenced, including: two meta-analyses, one randomized controlled trial, one narrative review, one prospective cohort study, one in vitro study, and one cross-sectional study.

Table 12A,B shows the studies that evaluated the relationship between coffee and prediabetes with their strength of evidence.

### 3.13. Physical Activity

This research was conducted based on the keywords: “prediabetes” OR “treating prediabetes” OR “prevention of diabetes” AND “physical activity” OR “exercise” OR “high-intensity interval training”. Five studies have been referenced, including: two meta-analyses, one meta-analysis of randomized controlled trials, one consensus report, one randomized controlled trial, and one systematic review.

Table 13A,B shows the studies that evaluated the relationship between physical activity and prediabetes with their strength of evidence.

### 3.14. Dietary Supplementation with Probiotics

This research was conducted based on the keywords: “gut microbiota” OR “pre-biotics” OR “probiotics” OR “synbiotics” AND “Prediabetes” OR “Blood glucose” OR “Diabetes mellitus” OR “Metabolic syndrome”. In total, 29 were sourced: 5 narrative reviews, 5 systematic reviews, 3 systematic reviews and meta-analyses, 7 clinical trials (4 cohort studies and 3 case–control studies), 2 in vitro studies, and 7 studies on animal models.

Table 14A,B shows the studies that evaluated the relationship between microbiota and prediabetes with their strength of evidence.

### 3.15. Monitoring Glucose Levels

This research was conducted based on the keywords: “glucose levels” OR “saliva collection method” OR “saliva” OR “salivary amylase” OR “serum glucose” OR “blood glucose” AND “prediabetes” OR “type 2 diabetes” OR “hyperglycemia” OR “diabetes risk” OR “diabetes diagnosis”. In total, 41 articles were consulted: 15 narrative reviews, 11 cross-sectional studies, 7 randomized controlled trials, 2 systematic reviews, 1 prospective study, 2 retrospective studies, 1 case–control study, 1 position statement, and 1 experimental study.

Table 15A,B shows the studies that evaluated the relationship between monitoring glucose levels and prediabetes with their strength of evidence.

**Table 1:** (**A**) Carbohydrate-containing foods and prediabetes interventional studies; and (**B**) carbohydrate-containing foods and prediabetes observational studies.

**(A) Carbohydrate-Containing Foods and Prediabetes Interventional Studies**								
**Authors**	**Type of Study**	**Population Characteristics**	**Type of Intervention**	**Duration**	**End Point**	**Results**	**Conclusion**	**Strength of Evidence**
Saslow L.R. et al., 2017 [25]	Parallel-group randomized (1:1) trial	Randomize 34 adults with glycated hemoglobin (HbA1c) > 6.0% and elevated body weight (BMI > 25) to a LCK diet (*n* = 16) or a MCCR diet (*n* = 18).	Participants attended 19 classes. All participants were encouraged to be physically active, get sufficient sleep, and practice behavioral adherence strategies based on positive affect and mindful eating.	12 months	Considerable uncertainty exists about the optimal level of carbohydrate intake. Previous research suggests that an ad libitum very-low-carbohydrate ketogenic diet (LCK) may improve metabolic measures in adults with T2DM and reduce the need for diabetes-related medications.	Compared to the MCCR group, the LCK group reported consuming fewer non-fiber grams of carbohydrates (6 and 12 months), more grams of fat (6 and 12 months), and more grams of protein (12 months), but not a different number of calories per day	Adults with prediabetes or noninsulin-dependent type 2 diabetes may be able to improve glycemic control with less medication by following an ad libitum very-low-carbohydrate ketogenic diet compared to a moderate-carbohydrate, calorie-restricted low-fat diet.	High
Saslow L.R. et al. 2014 [26]	Randomized pilot trial	Enrolled and randomized 34 participants to the MCCR (*n* = 18) or LCK (*n* = 16) diet groups.	MCCR diet (medium-carbohydrate, low-fat, calorie-restricted, carbohydrate-counting diet) consistent with guidelines from ADA. LCK diet (very-low-carbohydrate, high-fat, non-calorie-restricted diet) whose goal is to induce a low level of ketosis, here referred to as LCK (low-carbohydrate, ketogenic).	3 months	Compared a MCCR representative of conventional diabetic diet recommendations to an LCK (≤50 g carbohydrates per day not including fiber) in persons with HbA1c > 6.0%. The primary outcome measure was change in glycated hemoglobin (HbA1c) from baseline to 3 months.	Both groups had significant weight loss, even though the LCK group tended to lose more weight that the MCCR group (although only significant to the *p* = 0.09 level), even though only the MCCR group aimed to restrict calories.	In overweight and obese individuals with type 2 diabetes, a very-low-carbohydrate, high-fat, non-calorie-restricted diet may be more effective at improving blood glucose control than a medium-carbohydrate, low-fat, calorie-restricted, carbohydrate-counting diet that remains the standard for most diabetes education efforts.	High
Gardner C.D. et al., 2022 [27]	Randomized, crossover, interventional trial	40 participants aged ≥18 years with prediabetes or T2DM followed the well-formulated ketogenic diet (WFKD) and the Mediterranean-plus diet (Med-Plus) for 12 weeks each, in random order.	Compared two low-carbohydrate diets with three key similarities (nonstarchy vegetables, avoid added sugars and refined grains) and three key differences (avoid legumes, fruits, and whole, intact grains) for their effects on glucose control and cardiometabolic risk factors in individuals with prediabetes and T2DM.	3 months	The primary outcome was the percentage change in glycated hemoglobin (HbA1c) after 12 weeks on each diet.	The primary analysis was of 33 participants with complete data. The HbA1c values did not differ between diets at 12 weeks.	HbA1c values were not different between diet phases after 12 weeks, but improved from baseline on both diets, likely due to several shared dietary aspects.	High
**(B) Carbohydrate-Containing Foods and Prediabetes Observational Studies**								
**Authors**	**Type of Study**	**Population Characteristics**	**Type of Intervention**	**Duration**	**End Point**	**Results**	**Conclusion**	**Strength of Evidence**
Cea-Soriano L. et al., 2021 [28]	Cohort prospective study	1184 participants with prediabetes based on levels of fasting plasma glucose and/or glycated hemoglobin.	Hazard ratios of diabetes onset were estimated by Cox proportional regression models associated to high versus low/medium adherence to Mediterranean diet. Different propensity score methods were used to control for potential confounders.	4.2 years	Evaluate the effect from high adherence to Mediterranean diet on the risk of diabetes.	Incidence rate of diabetes in participants with high versus low/medium adherence to Mediterranean diet was 2.9 versus 4.8 per 100 persons-years. The hazard ratios adjusted for propensity score and by inverse probability weighting (IPW) had identical magnitude: 0.63 (95% confidence interval, 0.43–0.93). The hazard ratio in the adjusted model using propensity score matching 1:2 was 0.56 (95% confidence interval, 0.37–0.84).	These propensity score analyses suggest that high adherence to Mediterranean diet reduces diabetes risk in people with prediabetes.	Moderate
Filippatos T.D. et al., 2016 [29]	Cohort Prospective Study	3042 men and women (>18 year) were enrolled for the study.	In 2011 and 2012, the 10-year follow-up examinations were performed, including a working sample of *n* = 1875 participants without diabetes at baseline. Adherence to the Mediterranean diet at baseline evaluation was assessed using the MedDietScore (range 0–55).	10 years	Examine the effect of the Mediterranean diet on diabetes and CVD risk in subjects with impaired fasting glucose (IFG, i.e., fasting plasma glucose 100–125 mg/dL).	The prediabetic subjects (343) had a significantly higher incidence of diabetes (25% vs. 10%, *p* < 0.001) and CVD (17.8% vs. 12.3%, *p* = 0.007) compared with subjects with normal glucose values. A significant trend towards lower diabetes and CVD incidence was observed with medium and high adherence to the Mediterranean diet compared with low adherence (*p* < 0.001). High adherence to the Mediterranean diet (>35/55 score) was associated with lower 10-year incidence of diabetes and CVD.	High adherence to the Mediterranean diet is associated with a low risk of developing diabetes and CVD in prediabetic subjects.	Moderate
Li L. et al., 2022 [30]	Prospective observational study	9793 adults with prediabetes from the NHANES 1999–2014 (age > 20 years old).	Dietary intake was measured by 24 h recalls in the NHANES. From 1999 to 2002, one 24 h dietary recall was conducted in person in the NHANES Mobile Examination Center. From 2003 to 2014, dietary intake was measured by a 24 h dietary recall for two nonconsecutive days.	15 years	Examine the associations of different types of lower-carbohydrate diets (LCDs) and lower-fat diets (LFDs) with mortality among individuals with prediabetes.	Higher healthy LCD score was associated with favorable blood glucose, insulin, HOMA-IR, C-reactive protein (CRP), and blood lipids, whereas higher healthy LFD score was associated with lower blood glucose and CRP at baseline (all *p*-trend < 0.05).	Healthy LCD and LFD scores were significantly associated with lower all-cause mortality, whereas unhealthy LCD and LFD scores tended to be associated with higher all-cause mortality, among people with prediabetes.	Moderate

**Table 2:** (**A**) Milk and dairy products and prediabetes interventional studies; (**B**) milk and dairy products and prediabetes observational and experimental studies; (**C**) milk and dairy products and prediabetes review and meta-analysis; (**D**) eggs and prediabetes interventional studies; (**E**) eggs and prediabetes review and meta-analysis; (**F**) meat and prediabetes observational studies; (**G**) meat and prediabetes review and meta-analysis; (**H**) plant-based protein foods and prediabetes interventional studies; (**I**) plant-based protein foods and prediabetes observational studies; and (**J**) plant-based protein foods and prediabetes reviews and meta-analyses.

**(A) Milk and Dairy Products and Prediabetes Interventional Studies**								
Authors	**Type of Study**	**Population Characteristics**	**Type of Intervention**	**Duration**	**End Point**	**Results**	**Conclusion**	**Strength of Evidence**
Rideout T.C. et al., 2013 [31]	Randomized controlled trial	Twenty-three healthy subjects completed a randomized, crossover trial.	Randomly assigned to one of two treatment groups: a high-dairy-supplemented group instructed to consume four servings of dairy per day (HD); or a low-dairy-supplemented group limited to no more than two servings of dairy per day (LD).	12 months	Examine the influence of long-term (6 month) dairy consumption on metabolic parameters in healthy volunteers.	Body weight and composition, energy expenditure, blood pressure, blood glucose, and blood lipid and lipoprotein responses did not differ (*p* > 0.05) between the LD and HD groups. HD consumption improved (*p* < 0.05) plasma insulin (−9%) and insulin resistance (−11%, *p* = 0.03) as estimated by HOMA-IR compared with the LD group.	Study results suggest that high dairy consumption (4 servings/d) may improve insulin resistance without negatively impacting bodyweight or lipid status under free-living conditions.	High
Akhavan T. et al., 2010 [32]	Randomized controlled study	Experiment 1: 16 men; experiment 2: 12 men and 10 women.	Whey protein (10–40 g) in 300 mL water was provided in experiment 1, and whey protein (5–40 g) and whey protein hydrolysate (10 g) in 300 mL water were provided in experiment 2.		To describe the effect of whey protein or its hydrolysate when consumed before a meal on food intake, pre- and postmeal satiety, and concentrations of blood glucose and insulin in healthy young adults.	In experiment 1, 20–40 g WP suppressed food intake (*p* < 0.0001) and 10–40 g WP reduced postmeal blood glucose concentrations and the area under the curve (AUC) (*p* < 0.05). In experiment 2, 10–40 g WP, but not WPH, reduced postmeal blood glucose AUC and insulin AUC in a dose-dependent manner (*p* < 0.05).	WP consumed before a meal reduces food intake, postmeal blood glucose and insulin, and the ratio of cumulative blood glucose to insulin AUCs in a dose-dependent manner.	High
**(B) Milk and Dairy Products and Prediabetes Observational and Experimental Studies**								
** Authors **	**Type of Study**	**Population Characteristics**	**Type of Intervention**	**Duration**	**End Point**	**Results**	**Conclusions**	**Strength of Evidence**
Séverin Sindayikengera and Wen-shui Xia [33]	Experimental study		Whey protein chemical composition, protein solubility, amino acid composition, essential amino acid index (EAA index), biological value (BV), nutritional index (NI), chemical score, enzymic protein efficiency ratio (E-PER), and in vitro protein digestibility (IVPD) were determined.			The results indicated that the enzymatic hydrolysis of WPC 80 and sodium caseinate by Protamex improved the solubility and IVPD of their hydrolysates.	The nutritional qualities of WPC 80, sodium caseinate, and their hydrolysates were good and make them appropriate for food formulations or as nutritional supplement.	Moderate
Slurink I.A. et al., 2022 [34]	Prospective cohort study	2262 participants without (pre-) diabetes at enrolment (mean age 56 ± 7.3 years; 50% male).	Consumption of total dairy and dairy types.	6.4 ± 0.7 years of follow-up	Investigate prospective associations of consumption of total dairy and dairy types with incident prediabetes in a Dutch population-based study.	810 participants (35.9%) developed prediabetes. High-fat fermented dairy, cheese, and high-fat cheese were associated with a 17% (*p*-trend = 0.04), 14% (*p*-trend = 0.04), and 21% (*p*-trend = 0.01) lower risk of incident prediabetes, respectively, in top compared to bottom quartiles, after adjustment for confounders. Total dairy and other dairy types were not associated with prediabetes risk in adjusted models.	The highest intake of high-fat fermented dairy, cheese, and high-fat cheese were associated with a lower risk of prediabetes, whereas other dairy types were not associated. Cheese seems to be inversely associated with type 2 diabetes risk, despite high levels of saturated fatty acids and sodium.	Moderate
Slurink I.A. et al., 2022 [35]	Prospective cohort study	6770 participants (aged 62 ± 4 years, 59% female) free of (pre-)diabetes.		11.3 ± 4.8 years	To examine associations between dairy-type intake with prediabetes risk and longitudinal insulin resistance.	1139 incident prediabetes cases were documented (18.8%); a higher intake of high-fat yogurt was associated with lower prediabetes risk; higher intake of high-fat milk was associated with lower prediabetes risk.	A higher intake of high-fat yogurt was associated with a lower prediabetes risk and lower longitudinal insulin resistance. Additionally, high-fat milk was associated with a lower prediabetes risk.	Moderate
Hruby A. et al., 2017 [36]	Retrospective cohort study	2809 participants [mean ± SD age: 54.0 ± 9.7 year; body mass index (in kg/m^2^): 27.1 ± 4.7; 54% female].	Consumption of milk-based products.		To assess associations between consumption of milk-based products, incident prediabetes, and progression to T2D in the Framingham Heart Study Offspring Cohort.	902 (48%) developed prediabetes.	Associations of dairy with incident prediabetes or diabetes varied both by dairy product and type and by baseline glycemic status in this middle-aged US population.	Moderate
Slurink I.A. et al., 2023 [37]	Prospective cohort study	4891 participants with normal glucose tolerance (aged 49.0 ± 12.3 year, 57% female).	Consumption of dairy, including different types of dairy products.	12 years	To examine the relationship between the consumption of dairy, including different types of dairy products and risk of prediabetes.	765 (15.6%) incident cases of prediabetes were observed. The mean intake of dairy foods was 2.4 ± 1.2 servings/d, mostly consisting of low-fat milk (0.70 ± 0.78 servings/d) and high-fat milk (0.47 ± 0.72 servings/d).	Protective associations were found for high-fat dairy types, whereas neutral associations were seen for low-fat dairy types.	Moderate
Brouwer-Brolsma E.M. et al., 2018 [38]	Cross-sectional study	112,086 adults	A broad variety of dairy subgroups.	/	To assess cross-sectional associations of a broad variety of dairy subgroups with prediabetes and newly diagnosed type 2 diabetes (ND-T2DM) among Dutch adults.	Median dairy product intake was 324 (interquartile range 227) g/d; 25 549 (23%) participants had prediabetes; and 1305 (1%) had ND-T2DM. After full adjustment, inverse associations were observed of skimmed dairy (OR100 g 0.98; 95% CI 0.97, 1.00), fermented dairy (OR100 g 0.98; 95% CI 0.97, 0.99), and buttermilk (OR150 g 0.97; 95% CI 0.94, 1.00) with prediabetes.	Data showed inverse associations of skimmed and fermented dairy products with prediabetes. Positive associations were observed for full-fat and non-fermented dairy products with prediabetes and ND-T2DM.	Moderate
Pestoni G. et al., 2021 [39]	Cross-sectional study	1305 participants of the cross-sectional population-based KORA FF4 study.	/	/	To identify dietary patterns and to investigate their association with prediabetes, undetected diabetes, and prevalent diabetes.	Participants following the Western pattern had significantly higher chances of having prediabetes (odds ratio [OR] 1.92; 95% confidence interval [CI] 1.35, 2.73), undetected diabetes (OR 10.12; 95% CI 4.19, 24.43), or prevalent diabetes (OR 3.51; 95% CI 1.85, 6.67), compared to participants following the Prudent pattern.	These results suggest a very important role of dietary habits in the prevention of prediabetes and type 2 diabetes.	Moderate
**(C) Milk and Dairy Products and Prediabetes Review and Meta-Analysis**								
**Authors**	**Type of Study**	**Number of Studies**	**Subjects (Total)**		**End Point**	**Result**	**Conclusion**	**Strenght of Evidence**
Hidayat K. et al., 2019 [40]	Narrative review	/			Providing a clear presentation of the potential implementation of milk proteins as a dietary supplement in the prevention and management of T2DM by summarizing the relevant supporting evidence for this particular topic.	The results from these trials showed that milk proteins may have functional properties for stimulating postprandial insulin, resulting in lower postprandial glucose levels.		Low
Hoffman J.R. and Falvo M.J., 2004 [41]	Narrative review	/			To examine and analyze key factors responsible for making appropriate choices on the type of protein to consume in both athletic and general populations.			Low
Yau W.J. et al., 2020 [42]	Systematic review	Ninety-five articles involving a total of 11,211 participants were included in this review.	Nutritional strategies were broadly classified into four groups: low-calorie diet, low-glycemic-index diet, specific foods, and a combination of diet and exercise		Review on recently reported nutritional interventions for individuals with prediabetes.	More than 50% of reported interventions resulted in significant improvements in these parameters.		High
**(D) Eggs and Prediabetes Interventional Studies**								
**Authors**	**Type of Study**	**Population Characteristics**	**Type of Intervention**	**Duration**	**End Point**	**Results**	**Conclusion**	**Strenght Of Evidence**
Pourafshar S. 2018 [43]	RCT	42 overweight or obese individuals between the ages of 40 and 75 years with pre- and type II diabetes were included.	Participants were randomly assigned to receive either one large egg per day or an equivalent amount of egg substitute for 12 weeks. Blood samples were obtained to analyze lipid profile and biomarkers associated with glycemic control at all time points.	12 weeks	Evaluated if egg consumption may improve factors associated with glycemic control and insulin sensitivity.	Regular egg consumption resulted in improvements of fasting blood glucose, which was significantly (*p* = 0.05) reduced by 4.4% at the final visit in the egg group. Participants in the egg group had significantly (*p* = 0.01) lower levels of homeostatic model assessment of insulin resistance (HOMA-IR) at all visits.	Regular egg consumption has been shown to lead to an improvement in fasting blood sugar and insulin resistance levels (HOMA-IR). There were no significant changes in the levels of total cholesterol and LDL cholesterol.	High
**(E) Eggs and Prediabetes Review and Meta-Analysis**								
**Authors**	**Type of Study**	**Number of Studies**	**Subjects (Total)**		**End Point**	**Result**	**Conclusion**	**Strenght of Evidence**
Richard C., 2017 [44]	Sistematic review	Six original controlled randomized trials.	/		Evaluated the link between egg consumption and the main cardiovascular risk factors in subjects with type 2 diabetes or at risk of the disease (prediabetes, insulin resistance, or metabolic syndrome)	The majority of studies found that egg consumption did not affect major CVD risk factors. Consumption of 6 to 12 eggs per week had no impact on plasma concentrations of total cholesterol, low-density lipoprotein-cholesterol, triglycerides, fasting glucose, insulin, or C-reactive protein.	Results from randomized controlled trials suggest that consumption of 6 to 12 eggs per week, in the context of a diet that is consistent with guidelines on cardiovascular health promotion, has no adverse effect on major CVD risk factors in individuals at risk for developing diabetes or with type 2 diabetes	High
Drouin-Chartier J.P., 2020 [45]	Systematic review and meta-analysis of Prospective cohort studies	Three prospective cohort studies.	82,750 women from the Nurses’ Health Study (NHS; 1980–2012), 89,636 women from NHS II (1991–2017), and 41,412 men from the Health Professionals Follow-Up Study (HPFS; 1986–2016) with no diabetes, cardiovascular disease, and baseline cancer.		Evaluated the association between egg consumption and T2D risk	Documented 20,514 incident cases of T2D in the NHS, NHS II, and HPFS. In the pooled multivariable model adjusted for updated BMI, lifestyle, and dietary confounders, a 1 egg/d increase was associated with a 14% higher T2D risk. There were, however, significant differences by geographic region (P for interaction = 0.01). Each 1 egg/d was associated with higher T2D risk among US studies (RR: 1.18; 95% CI: 1.10, 1.27; *I*^2^ = 51.3%), but not among European (RR: 0.99; 95% CI: 0.85, 1.15; *I*^2^ = 73.5%) or Asian (RR: 0.82; 95% CI: 0.62, 1.09; *I*^2^ = 59.1%) studies.	Results from the updated meta-analysis show no overall association between moderate egg consumption and risk of T2D.	High
**(F) Meat and Prediabetes Observational Studies**								
**Authors**	**Type of Study**	**Population Characteristics**	**Type of Intervention**	**Duration**	**End Point**	**Results**	**Conclusion**	**Strenght of Evidence**
Nguyen et al., 2022 [46]	Cross-sectional study	3000 subjects, aged 40–60 years, living in rural communes in Khánh Hòa Province for at least six months.	Anthropometry results, blood samples for biochemical measurements and questionnaire information via face-to-face interviews were collected.	2–4 years	Examine the association between daily consumption of red/processed meat (0–99 g, 100–199 g or ≥200 g) and preDM/DM	The relative-risk ratios for DM were 1.00 (reference), 1.11 (95% CI = 0.75, 1.62) and 1.80 (95% CI = 1.40, 2.32) from the lowest to the highest red/processed meat consumption categories (*p*-trend = 0.006). The corresponding values for preDM were 1.00 (reference), 1.25 (95% CI = 1.01, 1.54), and 1.67 (95% CI = 1.20, 2.33) (*p*-trend = 0.004).	Increased red/processed meat consumption, but not poultry consumption, was positively associated with the prevalence of preDM/DM in rural communes in Khánh Hòa Province, Vietnam.	Moderate
Song et al., 2004 [47]	Prospective cohort study	37,309 participants in the Women’s Health Study aged ≥5 years who were free of cardiovascular disease, cancer, and type 2 diabetes.	Validated semi-quantitative food-frequency questionnaire.	8.8 years	To prospectively assess the relation between red meat intake and incidence of type 2 diabetes.	It was documented that there were 1558 incident cases of type 2 diabetes and positive associations were found between intakes of red meat and processed meat and risk of type 2 diabetes. Comparing women in the highest quintile with those in the lowest quintile, the multivariate-adjusted relative risks (RRs) of type 2 diabetes were 1.28 for red meat (95% CI 1.07–1.53, *p* < 0.001 for trend) and 1.23 for processed meat intake (1.05–1.45, *p* = 0.001 for trend).	Higher consumption of total red meat, especially various processed meats, may increase risk of developing type 2 diabetes in women.	Moderate
**(G) Meat and Prediabetes Review and Meta-Analysis**								
**Authors**	**Type of Study**	**Number of Studies**	**Subjects (Total)**	**End Point**	**Result**	**Conclusion**		**Strenght of Evidence**
Pan A. et al., 2011 [48]	Meta-analysis combined with three cohort studies	Three cohort studies + one meta analysis.	Meta-analysis (442,101 participants and 28,228 diabetes cases).	Evaluated the association between unprocessed and processed red meat consumption and incident T2D in US adults.	Documented 13,759 incident T2D cases. Both unprocessed and processed red meat intakes were positively associated with T2D risk in each cohort (all *p*-trend <0.001). The pooled HRs (95% CIs) for a one serving/d increase in unprocessed, processed, and total red meat consumption were 1.12, 1.32, and 1.14, respectively. The results were confirmed by a meta-analysis: the RRs (95% CIs) were 1.19 and 1.51 for 100 g unprocessed red meat and for 50 g processed red meat, respectively.	Red meat consumption, particularly processed red meat, is associated with an increased risk of T2D		High
Pan A. et al., 2013 [49]	Meta-analysis combined with three cohort studies	Three cohort studies	26,357 men in the Health Professionals Follow-Up Study (1986–2006), 48,709 women in the Nurses’ Health Study (1986–2006), and 74,077 women in the Nurses’ Health Study II (1991–2007).	Evaluate the association between changes in red meat consumption during a four-year period and subsequent four-year risk of T2DM in US adults.	Documented 7540 incident T2DM cases. Compared with the reference group of no change in red meat intake, increasing red meat intake of more than 0.50 servings per day was associated with a 48% elevated risk. Reducing red meat consumption by more than 0.50 servings per day from baseline to the first four years of follow-up was associated with a 14% lower risk	Increasing red meat consumption over time is associated with an elevated subsequent risk of T2DM, and the association is partly mediated by body weight. Limiting red meat consumption over time confers benefits for T2DM prevention.		High
Sanders L.M. et al., 2022 [50]	Systematic review and meta-analysis of randomized controlled trials	21 RCTs	Adult humans (≥18 y of age).	Evaluating the effects of diets containing red meat compared to diets with lower or no red meat, on markers of glucose homeostasis in adults.	Compared to diets with reduced or no red meat intake, there was no significant impact of red meat intake on insulin sensitivity, insulin resistance, fasting glucose, fasting insulin, glycated hemoglobin, pancreatic beta-cell function, or glucagon-like peptide-1. Red meat intake modestly reduced postprandial glucose compared to meals with reduced or no red meat intake.	The results suggest red meat intake does not impact most glycemic and insulinemic risk factors for T2D. Further investigations are needed on other markers of glucose homeostasis.		High
**(H) Plant-Based Protein Foods and Prediabetes Interventional Studies**								
**Authors**	**Type of Study**	**Population Characteristics**	**Type of Intervention**	**Duration**	**End Point**	**Results**	**Conclusion**	**Strength of Evidence**
Kwak J.H. et al., 2010 [51]	RCT	42 prediabetic or diabetic subjects.	Subjects with prediabetes and type 2 DM. Black soy peptide intervention group or placebo control group.	12 weeks	Effect of black soy peptide supplementation on glucose control in subjects with prediabetes and newly diagnosed type 2 diabetes mellitus.	Subjects with fasting glucose ≥ 110 mg/dL who consumed black soy peptides tended to have lower fasting glucose levels (two-tailed test, *p* = 0.098; one-tailed test, *p* = 0.049) and had a significant reduction in 2 h PG level (two-tailed *p* = 0.012, one-tailed *p* = 0.006), compared with baseline levels. The changes in 2 h PG levels were also statistically significant in the intervention group (−41.25 ± 13.67 mg/dL) compared with the placebo group (12.42 ± 9.80 mg/dL; two-tailed *p* = 0.015, one-tailed *p* = 0.008).	Black soy peptide supplementation may be beneficial for controlling fasting blood glucose levels and 2 h PG levels.	High
Reis C.E. et al., 2011 [52]	RCT	13 subjects (4 men and 9 women), with a mean age of 28.5 ± 10 years.	Four types of test meals were consumed: raw peanuts with skin (RPS), roasted peanuts without skin, ground-roasted peanuts without skin (GRPWS), or control meal. The test meals had the same nutrient composition.	24 h	Evaluate the effect of peanut processing on glycemic response, and energy and nutrients intake.	The area under the glycemic response curve after GRPWS was lower (*p* = 0.02) than the one obtained for RPS. There was no treatment effect on energy intake, macronutrients, and fiber consumption.	The consumption of ground-roasted peanuts may favor the control and prevention of diabetes due to its reduction on postprandial glucose response.	High
**(I) Plant-Based Protein Foods and Prediabetes Observational Studies**								
**Authors**	**Type of Study**	**Population Characteristics**	**Type of Intervention**	**Duration**	**End Point**	**Results**	**Conclusion**	**Strength of Evidence**
Becerra-Tomás N. et al., 2017 [53]	Prospective cohort study	3349 participants without type 2 diabetes at baseline. Aged 55–80 years.	-	4.3 years	Examine the associations between consumption of total legumes and specific subtypes, and type 2 diabetes risk.	266 new cases of type 2 diabetes. Individuals in the highest quartile of total legume and lentil consumption had a lower risk of diabetes than those in the lowest quartile (HR: 0.65; 95% CI: 0.43, 0.96; *p*-trend = 0.04; and HR: 0.67; 95% CI: 0.46–0.98; *p*-trend = 0.05, respectively).	A frequent consumption of legumes, particularly lentils, may provide benefits on type 2 diabetes prevention in older adults.	Moderate
Shams H. et al., 2008 [54]	Case–control study	30 patients with type 2 diabetes mellitus.	Two groups. Group A normal diet. Group B normal diet with 50 g cooked lentil and 6 g canula oil (substitute of 30 g bread and 20 g cheese)	6 weeks	Assess the effects of cooked lentil on serum blood glucose and lipid profile among type 2 diabetic patients.	Total cholesterol and fasting blood glucose decreased significantly in regimen containing lentils (*p* < 0.05).	Consumption of cooked lentil in breakfast led to reduction of FBS and TC and improvement of glycemic control in type 2 diabetic patients.	Moderate
Jiang R. et al., 2002 [55]	Prospective cohort study	83,818 women, aged 34 to 59 years, no history of diabetes, cardiovascular disease, or cancer.	Subjects completed a validated dietary questionnaire at baseline in 1980, and were followed-up for 16 years.	16 years	Examine prospectively the relationship between nut consumption and risk of type 2 diabetes.	3206 new cases of type 2 diabetes were documented. The consumption was inversely associated with risk of type 2 diabetes.	A higher consumption of nut and peanut butter may have some benefits in lowering risk of type 2 diabetes in women.	Moderate
**(J) Plant-Based Protein Foods and Prediabetes Reviews and Meta-Analyses**								
**Authors**	**Type of Study**	**Number of Studies**	**Subjects (Total)**	**End Point**	**Result**	**Conclusion**		**Strenght of Evidence**
Ley S.H. et al., 2014 [56]	Narrative review	-	-	To examine the role of diet in prevention and management of diabetes in the scientific literature.	-	-		Low
Kim Y., Keogh J.B., Clifton P.M., 2016 [57]	Narrative review	-	-	To provide a comprehensive overview of the anti-diabetic effects of commonly consumed dietary polyphenols.	In vitro and in vivo studies have shown that dietary polyphenolic compounds improved glucose homeostasis in the intestine, liver, muscle adipocytes, and pancreatic β-cells, as well as through prebiotic effects in the digestive tract.	Polyphenols may play a role in diabetes treatment and prevention but further investigations are needed.		Low
Schwingshackl L. et al., 2018 [58]	Meta-analysis	66 randomized trials (86 reports) comparing 10 food groups.	3595 participants	To assess the effects of main food groups (refined grains, whole grains, nuts, legumes, fruits and vegetables, eggs, dairy, fish, red meat, and sugar-sweetened beverages) on intermediate-disease markers across randomized intervention trials.	Legumes, but also whole grains, nuts, and refined grains, were more effective at reducing HOMA-IR (−1.01 to −0.53) compared with eggs and dairy. No significant effects were detected for HbA1c.	Increased intake of legumes (but also nuts and whole grains) is more effective at improving metabolic health than other food groups.		High
Schwingshackl L. et al., 2017 [59]	Meta-analysis	103 studies were included in the meta-analysis.	-	Synthesize the knowledge about the relation between intake of 12 major food groups (whole grains, refined grains, vegetables, fruits, nuts, legumes, eggs, dairy, fish, red meat, processed meat, and sugar-sweetened beverages) with risk of all-cause mortality.	An inverse association was observed for the highest compared with lowest legume intake categories (RR: 0.96; 95% CI: 0.93, 1.00; *I*^2^ = 48%; *p*-heterogeneity = 0.01). Consumption of legumes was associated with a decreased by 16% risk of all-cause mortality with increasing intake of legumes up to 150 g daily intake.	Legume intake up to 150 g/day may be helpful in reducing mortality for all causes (including diabetes and its complications).		High
Tang J. et al., 2020 [60]	Meta-analysis	15 cohort studies	565,810 individuals and 32,093 incident cases (diabetes occursions)	Summarize the longitudinal associations between legume and soy intake and risk of type 2 diabetes.	In dose–response analysis, significant linear inverse associations were observed for tofu, soy protein, and soy isoflavones (all *p* < 0.05). Overall quality of evidence was rated as moderate for total legumes and low for total soy and soy subtypes.	Dietary intakes of tofu, soy protein, and soy isoflavones, but not total legumes or total soy, are inversely associated with incident type 2 diabetes.		High
Li W. et al., 2018 [61]	Meta-analysis	Eight studies with 19 reports	335,230 participants	Evaluate the relationship between soy intake and type 2 diabetes mellitus risk.	A significant inverse association was shown between soy intake and type 2 diabetes mellitus risk with an overall RR of 0.77 (95% CI = 0.66–0.91) with high heterogeneity. Moreover, there was an obvious relationship between soy protein and isoflavones intake and risk of T2DM with the summary RR was 0.88 (95% CI = 0.80–0.97) with no heterogeneity.	Soy products and soy constituents (soy protein and soy isoflavones) may be associated with a lower risk of type 2 diabetes mellitus.		High

**Table 3:** (**A**) Sarcopenia and prediabetes observational studies; and (**B**) sarcopenia and prediabetes review and meta-analysis.

**(A) Sarcopenia and Prediabetes Observational Studies**								
**Authors**	**Type of Study**	**Population Characteristics**	**Type of Intervention**	**Duration**	**End Point**	**Results**	**Conclusion**	**Strength of Evidence**
Xu J. et al., 2023 [62]	Population study	16,116 U.S. adults aged 20–59 year with dual energy X-ray absorptiometry (DXA) from the National Health and Nutrition Examination Surveys (NHANES).	Evaluation of sarcopenia by DXA.	/	To explore the specific association between sarcopenia and prediabetes based on large population samples.	Sarcopenia was strongly associated with an increased risk of prediabetes after full adjustment (OR = 1.21, 95CI%: 1.05, 1.39, *p* = 0.009).	Sarcopenia was positively associated with prevalent prediabetes, especially IGT in the non-elderly.	Moderate
Kaga H. et al., 2022 [63]	Cross-sectional study	1629 elderly (mean age 73.1 ± 5.4 years) living in Japan.	Evaluation of glucose metabolism (75 g oral glucose tolerance test and glycated hemoglobin) and sarcopenia (BIA and hand grip strength).	/	To examine the relationship between sarcopenia and prediabetes.	Prediabetes and diabetes are independent risk factors for sarcopenia in men (prediabetes, odds ratio [OR] = 2.081 [95% confidence interval {CI}: 1.031–4.199]; diabetes, OR = 2.614 [95% CI: 1.362–5.018]) and diabetes, but not prediabetes, is an independent risk factor for sarcopenia in women (prediabetes, OR = 1.036 [95% CI: 0.611–1.757]; diabetes, OR = 2.099 [95% CI: 1.146–3.844]).	Although diabetes mellitus is associated with sarcopenia in both sexes, prediabetes is associated with sarcopenia in men, but not in women.	Moderate
Li S. et al., 2023 [64]	Observational study	22,482 adults aged ≥20 years in the National Health and Nutrition Examination Survey (NHANES).	Sarcopenia defined as ASMBMI (appendicular skeletal muscle mass/body mass index) < 0.789 for males, and <0.512 for females.	/	To explore the detailed correlation between prediabetes and sarcopenia.	Sarcopenia was directly correlated with prediabetes [OR (95% CI) = 1.230 (1.057, 1.431), *p* = 0.008] and T2DM [OR (95% CI) = 2.106 (1.625, 2.729), *p* < 0.001]. In non-T2DM population, HbA1c was negatively correlated with ASMBMI [β (95% CI) = −0.009 (−0.013, −0.005), *p* < 0.001].	Prediabetes is associated with increased risk of sarcopenia. HbA1c is an independent risk factor for loss of appendicular skeletal muscle mass and sarcopenia when HbA1c greater than 5.2% in the male non-T2DM population.	Moderate
**(B) Sarcopenia and Prediabetes Review and Meta-Analysis**								
**Authors**	**Type of Study**	**Number of Studies**	**Subjects (Total)**	**End Point**	**Result**	**Conclusion**		**Strenght of Evidence**
Qiao Y.S. et al., 2021 [65]	Meta-analysis	16 observational studies.	/	To investigate the association between the presence of sarcopenia and HbA1c values, prediabetes, diabetes, and its complications.	Three studies showed that high HbA1c levels lead to loss of muscle mass, and one study showed that people with prediabetes had lower muscle mass, strength, and performance than non-diabetic population. Seven studies showed that people with diabetes had a higher risk of sarcopenia than those without diabetes. The remaining five studies suggested that diabetic complications increased the risk of sarcopenia.	Subjects with prediabetes had reduced mass, strength, and muscle performance compared to non-diabetics.		High

**Table 4:** (**A**) Lipid-containing foods and prediabetes interventional studies; (**B**) lipid-containing foods and prediabetes observational studies; and (**C**) lipid-containing foods and prediabetes review and meta-analysis.

**(A) Lipid-Containing Foods and Prediabetes Interventional Studies**								
**Authors**	**Type of Study**	**Population Characteristics**	**Type of Intervention**	**Duration**	**End Point**	**Results**	**Conclusion**	**Strength of Evidence**
Díaz-Rizzolo et al., 2021 [66]	RCT	152 subjects with fasting glucose between 100–124 mg/dL aged ≥65 yo randomly distributed among control group (CG, *n* = 77) and sardine group (SG, *n* = 75).	Both groups received same T2D-prevention nutritional during a year but only SG had to add 200 g of sardine per week.	1 years	The study hypothesized that the consumption of twice a week of sardine, during one year, would reduce T2D-developing risk in a population with prediabetes (preDM) and old age.	Subjects in SG, significantly compared to CG, decreased percentage of individuals classified in a very high-risk group to develop T2D (*p* = 0.035). SG showed a lower HOMA-IR (*p* = 0.032).	A year T2D-prevention diet with sardine supplementation has a greater protective effect against developing T2D and CV events.	High
Rajabi-Naeeni M. et al., 2020 [67]	RCT	168 women of reproductive age with prediabetes and hypovitaminosis D, assigned to four groups.	Placebo group (omega-3 and vitamin D placebos); the omega-3 group (omega-3 supplements and vitamin D placebos); the vitamin D group (omega-3 placebos and vitamin D supplements); co-supplementation group. Every 2 weeks, the groups received 50,000 IU pearls of vitamin D and twice-daily doses of 1000-mg omega-3 tablets or placebos.	8 weeks	Determine the effectiveness of vitamin D and omega-3 co-supplementation on glycemic control and serum lipid profiles in women of reproductive age with prediabetes and hypovitaminosis D.	A significant reduction was observed in fasting glucose, insulin, homeostasis model assessment beta cell function, weight, and waist circumference in the co-supplementation group compared to the other three groups (*p* < 0.05).	Vitamin D and omega-3 co-supplementation improved fasting serum glucose, insulin, high-density lipoprotein-cholesterol level, homeostasis model assessment beta cell function, weight, and waist circumference in women of reproductive age with prediabetes and hypovitaminosis D.	High
Weta I.W. et al., 2017 [68]	RCT	45 young obese women randomized into two groups.	22 participants were supplemented with 2000 mg and 1000 mg of LA and ALA (Intervention), and 23 participants were given placebo (Control).	12 weeks	Elucidate the effect of the linoleic acid (LA) and alfa linolenic acid (ALA) supplementation to fasting plasma glucose in young women obese patients.	In the Intervention group, there was no significant change in fasting plasma glucose, contrary to it being increased (*p* = 0.007) in the Control group. The impaired plasma glucose increased in the Control group (OR = 8, *p* = 0.039), but not in the Intervention group (*p* = 0.508).	Restriction energy intake with supplementation 2000 mg and 1000 mg of LA and ALA control prediabetes of young obese women.	High
Jamilian M. et al., 2018 [69]	Randomized, double-blinded, placebo-controlled clinical trial	60 subjects, aged 18–40 years old with PCOS.	50,000 IU vitamin D every 2 weeks plus 2000 mg/day omega-3 fatty acid from fish oil (*n* = 30) or placebo (*n* = 30).	12 weeks	Evaluate the effect of the co-administration of vitamin D and omega-3 fatty acid on clinical, metabolic, and genetic parameters in women with polycystic ovary syndrome (PCOS).	Vitamin D and omega-3 fatty acid co-administration significantly decreased serum high-sensitivity C-reactive protein (hs-CRP) (*p* = 0.001) and malondialdehyde (MDA) levels (*p* < 0.001), and significantly increased plasma total antioxidant capacity (TAC) levels (*p* = 0.003) compared with the placebo. Vitamin D and omega-3 fatty acid co-supplementation significantly downregulated gene expression of interleukin-1 (IL-1) (*p* = 0.03), and upregulated vascular endothelial growth factor (VEGF) (*p* = 0.004) in PBMCs of subjects with PCOS, when compared with placebo.	The co-administration of vitamin D and omega-3 fatty acid for 12 weeks had beneficial effects on mental health parameters, serum total testosterone, hs-CRP, plasma TAC and MDA levels, and gene expression of IL-1 and VEGF among women with PCOS.	High
Njike, V.Y. et al., 2021 [70]	RCT cross-over	20 adults (mean age 56.1 years; 10 women, 10 men) at risk for T2DM.	Subjects were assigned to one of two possible sequence permutations of two different single dose treatments (50 mL of high-polyphenolic EVOO or 50 mL of refined olive oil without polyphenols), with 1-week washout.		To compare the effects of high-polyphenolic extra-virgin olive oil (EVOO) and refined olive oil without polyphenols on endothelial function (EF) in adults at risk for type 2 diabetes mellitus (T2DM).	EVOO acutely improved EF as compared to refined olive oil (1.2 ± 6.5% versus −3.6 ± 3.8%; *p* = 0.0086).	High-polyphenolic EVOO acutely enhanced EF in the study cohort, whereas refined olive oil did not.	High
Carnevale, R. et al., 2017 [71]	RCT	30 IFG patients	Patients were randomly allocated to a meal containing or not 10 g of EVOO in a cross-over design.	6 months	Was investigated if EVOO affects postprandial glucose and lipid profile in patients with impaired fasting glucose (IFG).	The meal containing EVOO was associated with a reduction of glucose (*p* = 0.009) and DPP4 activity (*p* < 0.001) and a significant increase of insulin (*p* < 0.001) and GLP-1 (*p* < 0.001) compared with the meal without EVOO.	In IFG patients, EVOO improves postprandial glucose and lipid profile with a mechanism probably related to incretin upregulation.	High
Bartimoccia, S. et al., 2022 [72]	Clinical trial	20 patients with impaired fasting glucose (IFG) and 20 healthy subjects (HS) matched for sex and age.	Variables were measured before and after a Mediterranean diet with 10 g EVOO added or not or in IFG patients before and after intake of 40 g chocolate with EVOO added or not.		The hypothesis that EVOO improves postprandial glycemia by reducing gut-permeability-derived low-grade endotoxemia was tested.	IFG had higher levels of LPS and zonulin. Two hours after a meal intake containing EVOO, IFG patients showed a less significant increase of blood glucose, a more marked increase of blood insulin and GLP1, and a significant reduction of LPS and zonulin compared to IFG patients not given EVOO.	Addition of EVOO to a Mediterranean diet or chocolate improves gut permeability and low-grade endotoxemia.	High
**(B) Lipid-Containing Foods and Prediabetes Observational Studies**								
**Authors**	**Type of study**	**Population Characteristics**	**Type of Intervention**	**Duration**	**End Point**	**Results**	**Conclusion**	**Strength of Evidence**
Abshirini et al., 2020 [73]	Case–control study	150 subjects with normal fasting glucose and 147 prediabetic subjects, aged 35–65.	FFQ with 168 food items.		Determine the association of DFQ (dietary fat quality) and fatty acid intake with prediabetes.	A positive association between intake of total SFA, myristic acid, palmitic acid, and prediabetes, and a negative association among *n*-3 PUFA, eicosapentaenoic, docosahexaenoic, and arachidonic acid intake and prediabetes.	Higher intake of dietary *n*-3 fatty acids was adversely related, whereas SFA intake was positively related to prediabetes morbidity.	Moderate
Mirmian P. et al., 2018 [74]	Cohort prospective study	2139 adults, free of T2DM, aged 20–70 y-old.	Diet information was collected with the use of a validated questionnaire at baseline.	5.8 years	Examine the association between fatty acid quantity and quality with risk of T2DM in adults.	Identified 143 incident T2D cases. When extreme quintiles were compared, cholesterol, monounsaturated fatty acids, polyunsaturated fatty acids, and ω-3 fatty acids were associated with T2DM. ω-6 to ω-3 ratio intake was associated with a higher risk of T2D. Also found positive associations between the ratios of total fat to ω-3.	Findings indicate that diets with high cholesterol, monounsaturated, polyunsaturated, and ω-3 fatty acids are associated with a lower risk of T2DM. Moreover, the ratios of ω-6/ω-3 and total fat/ω-3 were positively associated with T2DM.	Moderate
Nanri A. et al., 2011 [75]	Cohort prospective study	22,921 men and 29,759 women aged 45–75 y.	Participants completed a questionnaire of the second survey for the Japan Public Health Center-based Prospective Study.	5-year period	Prospectively investigated the association between fish intake and type 2 diabetes risk in Japanese adults.	971 new cases (572 men and 399 women) of type 2 diabetes were self-reported. In men, fish intake was significantly associated with a decreased risk of type 2 diabetes; In women, fish intake was not appreciably associated with type 2 diabetes risk.	In a population with high fish and seafood intake, fish consumption was associated with a lower risk of type 2 diabetes in men but not in women.	Moderate
Ibsen D.B. et al., 2019 [76]	Cohort study	A cohort of 53,163 participants from the Danish Diet, Cancer and Health study were followed for incident type 2 diabetes.	Diet was assessed by a validated 192-item food-frequency questionnaire at baseline.	Median follow-up time 15.4 years.	The associations between substitution of red meat (total, processed and unprocessed, low fat and high fat) with poultry or fish and substitution of processed red meat with unprocessed red meat and the risk of type 2 diabetes was examined.	6879 cases. Replacing total red meat with fish was associated with a lower risk of type 2 diabetes, as was replacement of processed red meat with poultry or fish. Replacing low fat red meat or high fat red meat with fish was associated with a lower risk of type 2 diabetes, whereas similar substitutions, with poultry, were not. Replacing processed red meat with unprocessed red meat was also associated with a lower risk of type 2 diabetes.	Replacing processed red meat with poultry, replacing total or processed red meat with fish, and replacing processed red meat with unprocessed red meat were all associated with a lower risk of type 2 diabetes.	Moderate
Chen G.C. et al., 2021 [77]	Prospective cohort study	392,287 middle-aged and older participants (55.0% women) in the UK Biobank who were free of diabetes, major cardiovascular disease, and cancer.	163,706 participated in one to five rounds of 24 h dietary recalls during 2009–2012.	Median 10.1 years of follow-up.	Evaluate associations of oily and nonoily fish consumption and fish oil supplements with incident type 2 diabetes.	7262 incident cases of T2D were identified.	Consumption of oily fish but not nonoily fish was associated with a lower risk of T2D. Use of fish oil supplements, especially constant use over time, was also associated with a lower risk of T2D.	Moderate
**(C) Lipid-Containing Foods and Prediabetes Review and Meta-Analysis**								
**Authors**	**Type of Study**	**Number of Studies**	**Subjects (Total)**	**End Point**	**Result**	**Conclusion**		**Strenght of Evidence**
Xun P. et al., 2012 [78]	Meta-analysis	Nine eligible studies	12 independent cohorts—438,214 individuals.	Assess the literature and determine the association between fish consumption and diabetes risk quantitatively.	Compared with those who never consumed fish or ate fish less than once per month, the pooled RR of incident diabetes was 0.99 (95% CI 0.85–1.16) for individuals who ate fish five or more times per week (*p*-trend = 0.80). Similar results were found for long-chain *n*-3 polyunsaturated fatty acid intake.	Evidence generated from this meta-analysis does not support an overall inverse association of fish or fish oil intake with incidence of diabetes.		High
Schwingshackl, L. et al., 2017 [79]	Systematic review and meta-analysis	Four cohort studies. 29 trials included in the metanalysis.	15,784 T2D	Illustrate the impact of olive oil (OO) on type 2 diabetes (T2D) by investigating the association between OO intake and risk of T2D, and the effect of OO intake in the management of T2D.	The highest OO intake category showed a 16% reduced risk of T2D. In T2D patients, OO supplementation resulted in a significantly more pronounced reduction in HbA1c.	The intake of OO could be beneficial for the prevention and management of T2D.		High

**Table 5:** (**A**) Vitamins and prediabetes interventional studies; (**B**) vitamins and prediabetes observational studies; and (**C**) vitamins and prediabetes review and meta-analysis.

**(A) Vitamins and Prediabetes Interventional Studies**								
**Authors**	**Type of Study**	**Population Characteristics**	**Type of Intervention**	**Duration**	**End Point**	**Results**	**Conclusion**	**Strength of Evidence**
Rasouli et al., 2022 [80]	RCT	1774 subjects with a mean age of 60.5 ± 9.8 years and a mean BMI of 31.9 ± 4.4 kg/m^2^ (44% female and 69% white). Participants had to meet at least two of three glycemic criteria for prediabetes defined by the 2010 American Diabetes Association guidelines.	Overweight/obese adults at high risk for type 2 diabetes (prediabetes) were randomly treated with vitamin D3 4000 IU or matching placebo daily. Disposition index (DI), as an estimate of β-cell function, was calculated as the product of HOMA2%Scpep and C-peptide response during the first 30 min of a 75 g oral glucose tolerance test (OGTT).	24 months	Investigate the effects of vitamin D3 supplementation on insulin sensitivity and β-cell function in adults at high risk for type 2 diabetes.	In the entire cohort, there were no significant differences in changes in DI, HOMA2%Scpep, or C-peptide response between the two groups. Vitamin D improved β-cell function among those who had baseline 25(OH)D levels below 12 ng/mL.	Supplementation with vitamin D3 for 24 months did not improve an OGTT-derived index of β-cell function in people with prediabetes not selected based on baseline vitamin D status; however, there was benefit among those with very low baseline vitamin D status.	High
**(B) Vitamins and Prediabetes Observational Studies**								
**Authors**	**Type of Study**	**Population Characteristics**	**Type of Intervention**	**Duration**	**End Point**	**Results**	**Conclusion**	**Strength of Evidence**
Banerjee et al., 2017 [81]	Cross-sectional study	202 subjects: 77 subjects with type 2 diabetes (mean age 48.09 ± 6.8), 73 with prediabetes (mean age 49.96 ± 7.6), and 52 healthy subjects constituting the control group (mean age 50.08 ± 7.1).	All subjects were matched for age, gender, and BMI within study groups. In all study groups, fasting serum levels of adiponectin, insulin and 25(OH)D were measured and routine biochemical parameters were analyzed.		Investigate the roles of deficient or deranged insulin, adiponectin, and 25 hydroxy vitamin D (25[OH]D) levels and to establish their interrelationship.	A statistically significant lower level of serum adiponectin and serum 25(OH)D and higher serum insulin levels in prediabetes or type 2 diabetes. The changes in the serum adiponectin or serum 25(OH)D in prediabetes and type 2 diabetes were inversely correlated with the serum levels of insulin.	The association of these hormones might act as a significant predictor of progression of prediabetes to type 2 diabetes.	Moderate
Jie Zhu et al., 2020 [82]	Prospective cohort study	4704 American adults (52% female and 51% black) between 18 and 30 years of age (mean age, 24.9 ± 3.6 years) and without diabetes enrolled in 1985–1986 and monitored through 2015–2016 in the Coronary Artery Risk Development in Young Adults (CARDIA) study.	Dietary assessment conducted by means of a validated anamnestic questionnaire at baseline, year 7, and year 20.	30 years	Prospectively examine intakes of folate, vitamin B6, and vitamin B12 in relation to diabetes incidence in a large U.S. cohort.	During 30 years of follow-up, 655 incident cases of diabetes occurred. Intake of folate was inversely associated with diabetes incidence after adjustment for potential confounders. Higher folate intake was also associated with lower plasma homocysteine (*p*-trend < 0.01) and insulin *p*-trend < 0.01).	Intake of folate in young adulthood was inversely associated with diabetes incidence in midlife among Americans.	Moderate
Jin G. et al., 2021 [83]	Cross-sectional study	22,041 participants (10,672 men and 11,369 women) over the age of 20.	Five diagnostic criteria (three based on laboratory data and two on questionnaires) were applied to define the condition of diabetes or prediabetes. A 24 h recall of two different days was used.		Define the association between folate, B12, and B6 obtained from diet and supplementation, and diabetes and prediabetes in American adults.	Of the 22,041 participants, 18.3% had diabetes and most were over 60 years old. Dietary folate and B6 were associated with a lower risk of diabetes, and, after adjusting for confounders, folate, B6, and B12 levels were inversely associated with diabetes. Dietary folate and B6 intakes were negatively associated with new diabetes diagnoses, and B12 and B6 were inversely associated with prediabetes.	There is an association between low values of B vitamins and the risk of diabetes, especially in the population over 60 years of age.	Moderate
Wilson et al., 2017 [84]	Cross-sectional study	89 participants, including individuals with normal glucose tolerance (*n* = 35), prediabetes (*n* = 25), and DM2 managed with diet alone or a metformin-only regimen (*n* = 29).	Participants completed a four-day food diary.		Analyzing plasma vitamin C concentrations across the glycemic spectrum and investigating the correlation with indices of metabolic health in adults with glycemic conditions ranging from normal glucose tolerance (NGT) to those with DM2.	Vitamin C plasma concentrations were significantly lower in subjects with DM2 than in those with NGT (41.2 µmol/L versus 57.4 µmol/L, *p* < 0.05), and a higher rate of deficiency was observed of vitamin C (i.e., <11.0 µmol/L) in both the prediabetic and DM2 groups. Fasting glucose, BMI, smoking history and dietary vitamin C intake are predictor-significant independents of plasma vitamin C concentrations.	These results suggest that adults with a history of smoking, prediabetes or DM2, and/or obesity have higher vitamin C requirements.	Moderate
**(C) Vitamins and Prediabetes Review and Meta-Analysis**								
**Authors**	**Type of Study**	**Number of Studies**	**Subjects (Total)**	**End Point**	**Result**	**Conclusion**		**Strenght of Evidence**
Zhang Y. et al., 2020 [85]	Systematic review and meta-analysis	Eight eligible studies	Total of 4896 subjects	Evaluate whether vitamin D supplementation reduces the risk of developing type 2 diabetes in patients with prediabetes.	All eight studies reported the development of new-onset diabetes, particularly in 1022 (20.9%) of 4896 participants. Combining data from all eight studies, vitamin D supplementation reduced the incidence of new-onset diabetes by 11%. Reversion of prediabetes to normoglycemia occurred in 116 of 548 (21.2%) participants in the vitamin D group and 75 of 532 (14.1%) participants in the control group.	In people with prediabetes, vitamin D supplementation reduces the risk of T2DM and increases the rate of reversion of prediabetes to normoglycemia.		High
Lind M.V. and al, 2019 [86]	Meta-analysis of RCTs	29 studies (4 with crossover design and 25 with parallel design).	Total of 22,250 participants.	Investigate the effects of folate supplementation on the outcome of insulin resistance and diabetes, evaluating the effect of placebo-controlled folate supplementation, alone or in combination with other B vitamins, on fasting glucose, insulin, HOMA-IR, HbA1c, or risk of type 2 diabetes.	Compared with placebo, folate supplementation lowered fasting insulin and HOMA-IR, but no overall effects were observed for fasting glucose or HbA1c. Changes in homocysteine after folate supplementation correlated with changes in fasting glucose and HbA1c.	Folate supplementation might be beneficial for glucose homeostasis and lowering IR, but, at present, there are insufficient data to conclusively determine the effect on development of T2D.		High
Ashor A.W. and al, 2017 [87]	Systematic review and meta-analysis of RCTs	22 studies (16 with parallel design and 6 with crossover design).	Total of 937 participants.	Test the effect of vitamin C administration on glucose, HbA1c, and insulin concentrations and on insulin sensitivity.	Vitamin C did not change glucose, insulin, and HbA1c concentrations. Subgroup analyses showed that vitamin C significantly decreased glucose concentrations in patients with DM2. The analyses showed a better benefit of vitamin C administration on glycemia in those with a higher baseline BMI and glucose concentration, with the greatest effects in the longer duration studies. Positive effects were observed on fasting insulin concentration values.	The greatest reduction in glucose concentrations was observed in patients with diabetes, older individuals, and with more prolonged supplementation.		High
Xu R. et al., 2014 [88]	Meta-analysis of RCTs	14 RCTs (12 evaluated the effects on HbA1c, 12 on fasting glucose, 6 on fasting insulin).	714 subjects (363 for the vitamin E group and 351 for the control group).	Characterize vitamin E impact (range of vitamin E administered 200–1600 IU/day, taken from 6 to 27 weeks) on HbA1c, fasting glucose, and fasting insulin.	Vitamin E supplementation did not lead to significant benefits in glycemic control, but subgroup analyses revealed significant reductions in HbA1c and fasting insulin versus controls in patients with low baseline vitamin E status.	There is insufficient evidence supporting a potential beneficial effect of vitamin E supplementation on improving HbA1c and fasting glucose and insulin concentrations in subjects with prediabetes.		High

**Table 6:** (**A**) Minerals and prediabetes interventional studies; (**B**) minerals and prediabetes observational studies; and (**C**) minerals and prediabetes reviews and meta-analysis.

**(A) Minerals and Prediabetes Interventional Studies**								
**Authors**	**Type of Study**	**Population Characteristics**	**Type of Intervention**	**Duration**	**End Point**	**Results**	**Conclusion**	**Strength of Evidence**
Pittas, A.G. et al., 2007 [89]	Double-blinded randomized controlled trial	314 Caucasian adults	Received, either 500 mg calcium citrate and 700 IU vitamin D(3), or placebos daily for 3 years	3 years	Compare the effects of combined calcium and vitamin D supplementation versus placebo on blood glucose and markers of inflammation in nondiabetic adults aged > or =65 years.	Effects of combined calcium–vitamin D supplementation on 3-year change in FPG depended on baseline FPG (*p* = 0.02 for interaction). Among participants with IFG at baseline, those who took combined calcium–vitamin D supplements had a lower rise in FPG at 3 years compared with those on placebo (0.02 mmol/L [0.4 mg/dL] vs. 0.34 mmol/L [6.1 mg/dL], respectively, *p* = 0.042) and a lower increase in HOMA-IR (0.05 vs. 0.91, *p* = 0.031).	In healthy, older adults with IFG, supplementation with calcium and vitamin D may attenuate increases in glycemia and insulin resistance that occur over time.	High
Ali, A. et. al., 2011 [90]	Double-blind randomized controlled trial	59 adult with IFG	Randomized, double-blind, placebo-controlled, modified cross-over clinical trial. Participants received six-month sequences of chromium picolinate or placebo at one of two dosages (500 or 1000 mcg daily).	Six months	To investigate the effects of daily chromium picolinate supplementation on serum measures of glucose tolerance and insulin sensitivity in patients at high risk for type 2 diabetes mellitus.	No changes were seen in glucose level, insulin level, or HOMA-IR (all *p* > 0.05) after six months of chromium at either dosage level (500 mcg or 1000 mcg daily) when compared with placebo.	Chromium supplementation does not appear to ameliorate insulin resistance or impaired glucose metabolism in patients at risk for type 2 diabetes and, thus, is unlikely to attenuate diabetes risk.	High
**(B) Minerals and Prediabetes Observational Studies**								
**Authors**	**Type of Study**	**Population Characteristics**	**Type of Intervention**	**Duration**	**End Point**	**Results**	**Conclusion**	**Strength of Evidence**
Zhou, Q. et al., 2019 [91]	Cross-sectional Study	Patients with IFG (*n* = 12), IGT (*n* = 15), T1D (*n* = 25), T2D (*n* = 137), and healthy controls (*n* = 50)	Se was detected using inductively coupled plasma spectrometer.	/	This study investigated serum and urinary Se levels in patients with impaired fasting glucose (IFG), impaired glucose tolerance (IGT), type 1 diabetes (T1D), and type 2 diabetes (T2D) in northeast Chinese populations.	The serum Se level was dramatically lower in patients with T1D and was significantly higher in IFG subjects, and the urinary Se concentration was markedly lower in IGT and T2D groups. The serum Se levels were positively correlated with serum zinc (Zn) in both IFG and IGT groups, while urinary Se were positively associated with urinary Zn and copper (Cu) in IGT group.	The serum Se levels were positively correlated with serum Cu in both T1D and T2D groups, and urinary levels of Se were positively associated with serum zinc and urinary Cu, Zn, calcium (Ca), and magnesium (Mg) and negatively correlated with serum Ca and Mg in T2D group, while the urinary levels of Se were positively correlated with urinary Zn and Mg both in peripheral neuropathy (DPN) and retinopathy (DR) groups.	Moderate
Xu, J. et al., 2013 [92]	Cross-sectional study	Patients with type 1 diabetes (T1D, *n* = 25), type 2 diabetes (T2D, *n* = 137), impaired fasting glucose (IFG, *n* = 12) or impaired glucose tolerance (IGT, *n* = 15), and age/gender matched controls (*n* = 50)		/	The association of copper and zinc levels in the serum or urine of patients living in northeast China, with either prediabetes or diabetes	Serum copper levels were significantly higher in IFG, IGT, and T2D groups. Serum zinc level was dramatically lower, and urinary zinc level was significantly higher in both T1D and T2D subjects compared with controls.	Simvastatin treatment in T2D patients had no significant effect on serum and urinary copper and zinc. These results suggest the need for further studies of the potential impact of the imbalanced serum copper and zinc levels on metabolic syndrome, diabetes, and diabetic complications.	Moderate
Hruby, A. et al., 2014 [93]	Prospective cohort study	2582 community-dwelling participants 26–81 years old at baseline		7 years	To assess 7-year associations between magnesium intake and incident prediabetes and/or insulin resistance (IR), and progression from these states to type 2 diabetes	Higher magnesium intake tended to be associated with lower follow-up FG and IR, but not fasting insulin, postload values, or insulin sensitivity.	Magnesium intake may be particularly beneficial in offsetting risk of developing diabetes among those at high risk. Magnesium’s long-term associations with non-steady-state (dynamic) measures deserve further research.	Moderate
Kieboom, B.C.T. et al., 2017 [94]	Retrospective cohort study	8555 participants (mean age, 64.7 years; median follow-up, 5.7 years) with normal glucose levels (mean ± SD: 5.46 ± 0.58 mmol/L) at baseline			Study the directionality of the association between serum magnesium levels and diabetes	A 0.1 mmol/L decrease in serum magnesium level was associated with an increase in diabetes risk (HR 1.18 [95% CI 1.04, 1.33]); similar association was found between serum magnesium levels and prediabetes risk (HR 1.12 [95% CI 1.01, 1.25])	Low serum magnesium levels are associated with an increased risk of prediabetes and this increased risk is similar to that of diabetes.	Moderate
Guerrero-Romero, F. et al., 2008 [95]	Prospective cohort study	1122 individuals (20–65 years of age) were enrolled between 1996 and 1997, and 817 individuals re-examined about 10 years later	New-onset IFG (5.6–7.0 mmol L^−1^ fasting glucose), IGT (7.8–11.1 mmol L^−1^ glucose 2-h postload), and type 2 diabetes were determined from the number of subjects who had these conditions at the second examination without evidence that they were present at the first one.	10 years	To examine the association between serum magnesium levels and the risk for developing IFG, IGT, and type 2 diabetes	New-onset IFG and IGT was identified in 276 (33.8%) individuals. The relative risk for IFG, IGT, and IFG + IGT was 1.11 (95% confidence interval, 0.5–5.1), 1.38 (95% confidence interval, 1.1–6.3), and 1.49 (95% confidence interval, 1.1–4.9), respectively. New-onset diabetes was identified in 78 (9.5%) individuals (relative risk 2.54; 95% confidence interval, 1.1–4.1).	Hypomagnesaemia is independently associated with the development of IGT, IFG + IGT, and type 2 diabetes, but not with the development of IFG	Moderate
Wu, F. et al., 2019 [96]	Prospective cohort study	1134 subjects; age 3 to 18 years at baseline	Dietary calcium intake was assessed at baseline (1980) and adult follow-up visits (2001, 2007, and 2011). Long-term (mean between youth and adulthood) dietary calcium intake was calculated.	31 years	Examine whether youth and long-term (between youth and adulthood) dietary calcium intake is associated with adult impaired glucose metabolism and type 2 diabetes (T2D).	No evidence for nonlinear associations between calcium intake and IFG or T2D among females and males (all *p* for nonlinearity > 0.05). Higher youth and long-term dietary calcium intake was not associated with the risk of IFG or T2D among females or males	Youth or long-term dietary calcium intake is not associated with adult risk of developing impaired glucose metabolism or T2D	Moderate
Alissa, E.M. et al., 2009 [97]	Cross-sectional study	130 Saudi men with an established history of myocardial infarction and 130 age-matched controls without established CVD	Measured serum and urine chromium concentrations, fasted lipid profile, plasma glucose, and serum lipid peroxide		To investigate chromium status among Saudi men with and without established cardiovascular disease (CVD) and its relationship to glucose tolerance, lipid profile, and other established CVD risk factors	Patients with established CVD had higher serum triglycerides (*p* < 0.05) and plasma glucose (*p* < 0.0001) and lower serum and urinary chromium concentrations (*p* < 0.0001) than controls. Serum chromium was inversely correlated with plasma glucose among cases and controls (r = −0.189, *p* < 0.05 and r = −0.354, *p* < 0.00001, respectively).	While chromium metabolism appears to be altered in individuals with CVD, it is unclear whether chromium supplementation would be effective in CVD prevention among patients with IGT.	Moderate
**(C) Minerals and Prediabetes Reviews and Meta-Analysis**								
**Authors**	**Type of Study**	**Population Characteristics**	**Type of Intervention**	**End Point**	**Results**	**Conclusion**		**Strength of Evidence**
Capdor, J. et al., 2013 [98]	Meta-analysis	3978 subjects were included in the meta-analysis.	A systematic review and meta-analysis of randomized placebo-controlled trials was conducted.	To determine the effect of zinc supplementation on fasting blood glucose, HbA1c, serum insulin, and serum zinc concentrations.	A small but statistically significant reduction in fasting glucose concentrations was observed (−0.19 ± 0.08 mmol/L, *p* = 0.013) after zinc supplementation. HbA1c tended to decrease in zinc-supplemented individuals (−0.64 ± 0.36%, *p* = 0.072). No significant effect was observed for serum insulin concentrations.	The significant, albeit modest, reduction in glucose concentrations and tendency for a decrease in HbA1c following zinc supplementation suggest that zinc may contribute to the management of hyperglycemia in individuals with chronic metabolic disease.		High
Wang X. et al., 2019 [99]	Meta-analysis	Thirty-two placebo-controlled interventions were extracted from 36 publications, involving a total of 1700 participants in 14 countries.	Zinc supplementation.	To assess the effects of zinc supplementation in preventing and managing diabetes.	The subjects in the zinc-supplementation group had a statistically significant reduction in fasting glucose [FG, weighted mean difference (WMD): −14.15 mg/dL; 95% CI: −17.36, −10.93 mg/dL], 2 h postprandial glucose (WMD: −36.85 mg/dL; 95% CI: −62.05, −11.65 mg/dL), fasting insulin (WMD: −1.82 mU/L; 95% CI: −3.10, −0.54 mU/L), homeostasis model assessment for insulin resistance (WMD: −0.73; 95% CI: −1.22, −0.24), glycated hemoglobin (WMD: −0.55%; 95% CI: −0.84, −0.27%), and high-sensitivity C-reactive protein (WMD: −1.31 mg/L; 95% CI: −2.05, −0.56 mg/L) concentrations.	Several key glycemic indicators are significantly reduced by zinc supplementation, particularly, the FG in subjects with diabetes and in subjects who received an inorganic zinc supplement.		High
Althuis, M.D. et al., 2002 [100]	Meta-analysis	This review summarizes data on 618 participants from the 15 trials.		To determine the effect of chromium on glucose and insulin responses in healthy subjects and in individuals with glucose intolerance or type 2 diabetes.	The meta-analysis showed no association between chromium and glucose or insulin concentrations among nondiabetic subjects. Three trials reported data on Hb A(1c): one study each of persons with type 2 diabetes, persons with impaired glucose tolerance, and healthy subjects.	Data from RCTs show no effect of chromium on glucose or insulin concentrations in nondiabetic subjects. The data for persons with diabetes are inconclusive.		High
Wang, Z.Q. et al., 2010 [101]	Narrative review			Chromium supplementation in type 2 diabetes and insulin resistance.	A consistent significant and beneficial effect of chromium may not be observed. Specifically, recent data fail to demonstrate significant improvement in carbohydrate metabolism in individuals with metabolic syndrome, impaired glucose tolerance, or consistently in individuals with type 2 diabetes.			Low

**Table 7:** Osteoporosis and prediabetes studies.

Authors	Type of Study	Population Characteristics	Type of Intervention	Duration	End Point	Results	Conclusion	Strength of Evidence
Liu Y et al., 2023 [102]	Multistage cross-sectional study	A total of 23,825 subjects (7427 with prediabetes).	Data from the U.S. National Health and Nutrition Examination Surveys. Bone mineral density and the skeletal muscle mass index (SMI) were measured with dual-energy X-ray absorptiometry (DXA).	Data from 2009 to 2018	Investigate the effect of sarcopenia, osteoporosis and osteosarcopenia on spine fracture in patients with prediabetes.	Regarding bone health, the lumbar and spinal bone mineral density of the prediabetes group was lower than in the healthy group, while there were no differences between diabetic and healthy subjects. The prevalence of osteoporosis was higher in diabetics than in prediabetics, and higher in the latter than in healthy subjects.	Osteoporosis is a risk factor for spine fracture in prediabetic adults and the combination of sarcopenia and osteoporosis further increases the prevalence of spine fracture.	Moderate

**Table 8:** (**A**) Fruits and vegetables and prediabetes observational studies; and (**B**) fruits and vegetables and prediabetes review and meta-analysis.

**(A) Fruits and Vegetables and Prediabetes Observational Studies**								
**Authors**	**Type of Study**	**Population Characteristics**	**Type of Intervention**	**Duration**	**End Point**	**Results**	**Conclusion**	**Strength of Evidence**
Barouti et al., 2022 [103]	Cohort prospective study	6961 men and women, aged between 35 and 56	Questionnaire for diet assessment	20 years	To establish a relationship between consumption of fruit and vegetables and the development of dysglycemia.	An inverse relationship was seen between fruit and vegetable consumption and the development of dysglycemia.	Increasing fruits and vegetables in the diet reduces the risk of dysglicemia (HR: 0.86)	High
Li et al., 2023 [104]	Cohort prospective study	79,922 patients aged over 40 years	Questionnaire for diet assessment	Four years	To establish a relationship between consumption of fruit and vegetables and the development of dysglycemia.	The risk of development of dysglycemia decreased especially in normoglycemic patients in whom the consumption of more than seven portions of fruit per week decreased the risk of developing diabetes by 48%.	Increasing fruits and vegetables in the diet reduces the risk of dysglycemia	High
Wu et al., 2021 [105]	Cross-sectional study	6802 participants between 18 and 65 years old	Questionnaire for diet assessment		To establish a relationship between diet and the development of DM2 or IFG.	A reduction in the risk of prediabetes was seen with a consumption of fruit and vegetables corresponding to the third and fourth quartile, i.e., between 320 and 530 g (vegetables + fruit/day).	Increasing fruits and vegetables in the diet reduces the risk of dysglycemia	High
Zhang et al., 2022 [106]	Cohort prospective study	18,085 participants who at baseline had neither diabetes nor prediabetes or other CVD nor cancer	Questionnaire for diet assesment	63,175 person-years	To establish a relationship between the consumption of fiber and DM2 or IFG.	4139 cases of diabetes occurred.	Fiber intake was inversely related to the incidence of prediabetes. The type of fiber that was found to be most effective in preventing prediabetes is soluble fiber, mostly found in fruit and vegetables.	High
Lopez Ridarura et al., 2004 [107]	Cohort prospective study	85,060 women and 42,872 men	Magnesium intake was evaluated using a validated food-frequency questionnaire every 2–4 years	18 years	To establish if magnesium intake could reduce the risk of developing diabetes mellitus.	Magnesium reduces the risk of diabetes.	The results of this study confirmed the inverse association between magnesium intake and diabetes risk	High
**(B) Fruits and Vegetables and Prediabetes Review and Meta-Analysis**								
**Authors**	**Type of Study**	**Number of Studies**	**Subjects (Total)**	**End Point**	**Result**	**Conclusion**		**Strenght of Evidence**
Min Li et al., 2014 [108]	Meta-analysis and systematic review of prospective cohort studies	A total of 10 articles including 13 comparisons with 24,013 cases of type 2 diabetes	434,342 participants	To assess the relationship between fruit and vegetable consumption and the risk of developing diabetes	Evidence of curve linear associations was seen between fruit and green leafy vegetables consumption and risk of type 2 diabetes.	Higher fruit or green leafy vegetables intake is associated with a significantly reduced risk of type 2 diabetes.		High

**Table 9:** Nuts and prediabetes interventional studies.

Authors	Type of Study	Population Characteristics	Type of Intervention	Duration	End Point	Results	Conclusion	Strength of Evidence
Gulati et al., 2022 [109]	RCT with two parallel groups	66 participants with prediabetes in the range of 18–60 years.	The effects of a dose of 20 g of almonds before lunch and dinner was evaluated.	Three months	To evaluate the risk of prediabetes and DM2.	The intake of almonds before main meals would therefore seem to help in the prevention of the evolution from prediabetes to frank diabetes.	Eating almonds before meals can help reduce the risk of DM2 and IFG.	High
Casas-Agustench P. et al., 2011 [110]	Randomized parallel-group study	50 patients with metabolic syndrome were instructed to consume 30 g of dried fruit per day.	People were instructed to consume 30 g of dried fruit per day (15 g walnuts, 7.5 g almonds, 7.5 g hazelnuts).	12 weeks	To evaluate the effects of nuts in inflammatory markers and HOMA index.	The nut group reduced fasting insulin by 2.60 μU/mL (95% CI, −4.62 to −0.59) and HOMA by 0.72 (−1.28 to −0.16) (*p* < 0.05). Among inflammatory markers, IL6 levels decreased by 1.1 ng/mL.	Eating nuts seems to reduce HOMA index and inflammatory markers.	High
Hou Y. et al., 2018 [111]	RCT	32 patients with DM2.	60 g/day peanuts for men and 50 g/day for women in the Peanut group, and 55 g/day almonds for men and 45 g/day for women in the Almond group.	Three months	To evaluate the relationship with peanuts and almonds in the diet and BMI and glycometabolic patterns.	There were significant pre-post changes in fasting and postprandial blood glucose (*p* < 0.05). The glycated value was reduced in the almond group (*p* < 0.05).	Introducing in the diet of peanuts and almonds can improve glycometabolic patterns.	High
Wien M. et al., 2010 [112]	Randomized parallel-group study	65 people with prediabetes.	Almond intake was approximately 20% of the daily caloric intake, approximately 60 g/day.	16 weeks	To evaluate the relationship with almonds in the diet and BMI and glycometabolic patterns.	The intervention group showed both a reduction in insulin HOMA IR and HOMA 2B compared with the control group without almonds.	Introducing in the diet of almonds can improve glycometabolic patterns and can help reduce insulin resistance.	High
Lu et al., 2021 [113]	Randomized crossover study	Ten overweight Chinese men with a mean age of 47.9 years.	Ingestion of a nut-based, high-protein snack bar or an isocaloric bar contain more carbohydrates. The bar used contained a caloric quantity of about 1000 KJ.	0–120 min	Evaluate the glycemic response in a 2 h window with two different bars.	In the patients who received the bar rich in dried fruit, there was a reduction in blood sugar (*p* < 0⋅05) in the 30–120 min after ingestion of both the bar alone and the bar associated with 50 g of white bread (a food that normally raises blood sugar), with an area under the glycemic curve 10 times lower.	The nut bar can reduce postprandial glycemia.	High

**Table 10:** (**A**) Plain water and prediabetes interventional studies; (**B**) sugary beverages and prediabetes interventional studies; (**C**) sugary beverages and prediabetes observational studies; and (**D**) sugary beverages and prediabetes reviews, meta-analyses, and guidelines.

**(A) Plain Water and Prediabetes Interventional Studies**								
**Authors**	**Type of Study**	**Population Characteristics**	**Type of Intervention**	**Duration**	**End Point**	**Results**	**Conclusion**	**Strength of Evidence**
Carroll et al., 2015 [114]	Cross-sectional study	138 adult subjects	The risk of DM2, blood pressure, fruit and vegetable intake, and beverage intake were evaluated. The DM2 risk score was calculated using the Diabetes UK risk assessment tool validated for use in the UK. Dietary variables were assessed using an FFQ. Water was measured in beakers and one beaker equaled 240 mL.	The past seven days	Investigate the correlation between water intake and risk of developing type 2 diabetes mellitus in an adult population in the United Kingdom.	Water intake was negatively correlated with the risk of developing DM2. A trend of low water consumption emerged in groups classified as high-risk for DM2. The analyses showed that each 240 mL portion of water drunk per day was associated with a 0.72-point decrease in the risk of DM2. The mean risk reduction in each participant was 1.7 points.	A negative correlation was observed between water intake and risk score, suggesting that water intake may play a significant role in the development and prevention of DM2.	Moderate
Carroll et al., 2016 [115]	Cross-sectional study	Total of 1035 subjects (456 men and 579 women, with an average age of 44 years)	Data includes a 4-day food diary and HbA1c values from blood sampling. The analyses used linear and logistic regressions stratified by gender modeling the associations of glasses per day (240 mL) of still water with HbA1c and probabilities of HbA1c ≥ 5–5%, respectively. Sugary drinks, fruit juices, and artificially sweetened drinks have been transformed with modeling systems into still water.	From 2008 to 2012	Analyze the association between simple water intake and HbA1c trend in the National Diet and Nutrition Survey (2008–2012) rolling survey.	1 glass/day of water was found to be associated with a reduction in HbA1c of −0–4% in men and men had a 22% reduced chance of HbA1c ≥ 5–5%/day cup of plain water. Still water intake was associated with lower HbA1c in men but not in women.	Water can be specifically associated with a reduction in the risk of DM2 and that intake of simple water and the right hydration are very important in the prevention or influence on the evolution of prediabetes into diabetes.	Moderate
**(B) Sugary Beverages and Prediabetes Interventional Studies**								
**Authors**	**Type of Study**	**Population Characteristics**	**Type of Intervention**	**Duration**	**End Point**	**Results**	**Conclusion**	**Strength of Evidence**
Maersk M. et al., 2012 [116]	Randomized intervention study	Overweight subject (*n* = 47)	Subject randomly assigned to four different test drinks - (1 L/d for 6 mo): SSSD (regular cola); - isocaloric semiskim milk; - aspartame-sweetened diet cola; - water.	Six months	To compare the effects of sucrose-sweetened soft drinks (SSSDs) with those of isocaloric milk and a noncaloric soft drink on changes in total fat mass and ectopic fat deposition (in liver and muscle tissue).	The relative changes between baseline and the end of 6-month intervention were significantly higher in the regular cola group than in the three other groups for liver fat (132–143%, sex-adjusted mean; *p* < 0.01), skeletal muscle fat (117–221%; *p* < 0.05), visceral fat (24–31%; *p* < 0.05), blood triglycerides (32%; *p* < 0.01), and total cholesterol (11%; *p* < 0.01). Total fat mass was not significantly different between the four beverage groups.	Daily intake of SSSDs for 6 mo increases ectopic fat accumulation and lipids compared with milk, diet cola, and water. Thus, daily intake of SSSDs is likely to enhance the risk of cardiovascular and metabolic diseases.	High
Raben A. et al., 2002 [117]	Randomized controlled trial	Overweight men and women (sucrose group *n* = 21, mean BMI 28 kg/m^2^; sweeteners group *n* = 20, mean BMI 27.6 kg/m^2^)	Subjects consumed daily supplements of either sucrose or artificial sweeteners.	10 weeks	To investigate the effect of long-term supplementation with drinks and foods containing either sucrose or artificial sweeteners on ad libitum food intake and body weight in overweight subjects.	Body weight and fat mass increased in the sucrose group (by 1.6 and 1.3 kg, respectively) and decreased in the sweetener group (by 1.0 and 0.3 kg, respectively); the between-group differences were significant at *p* < 0.001 (body weight) and *p* < 0.01 (fat mass).	Overweight subjects who consumed fairly large amounts of sucrose (28% of energy), mostly as beverages, had increased energy intake, body weight, fat mass, and blood pressure after 10 wk. These effects were not observed in a similar group of subjects who consumed artificial sweeteners.	High
**(C) Sugary Beverages and Prediabetes Observational Studies**								
**Authors**	**Type of Study**	**Population Characteristics**	**Type of Intervention**	**Duration**	**End Point**	**Results**	**Conclusion**	**Strength of Evidence**
Sylvetsky A.C. et al., 2017 [118]	Cross-sectional study using National Health and Nutrition Examination Survey data from 2009 to 2012	The final sample size was 16,942	/	Three years	To describe low-calorie sweetener (LCSs) consumption in the United States and to characterize consumption by sociodemographic subgroups, source, frequency, eating occasion, and location.	LCS consumption was higher in females compared with males among adults. Individuals of non-Hispanic white race/ethnicity also had higher prevalence of consumption compared with non-Hispanic blacks and Hispanics and those in the highest tertile of income had higher LCS consumption compared with individuals of middle or low income across LCS product categories in adults, and for LCS beverages and LCS foods in children.	LCS consumption is highly prevalent in the United States, among both children and adults. Well-controlled, prospective trials are required to understand the health impact of this widespread LCS exposure.	Medium
**(D) Sugary Beverages and Prediabetes Reviews, Meta-Analyses, and Guidelines**								
**Authors**	**Type of Study**	**Number of Studies**	**Subjects (Total)**	**End Point**	**Result**	**Conclusion**		**Strenght of Evidence**
Drouin-Chartier et al., 2019 [119]	Meta-analysis.	Three large prospective cohort studies.	76,531 women in the Nurses’ Health Study (1986–2012); 81,597 women in the Nurses’ Health Study II (1991–2013); 34,224 men in the Health Professionals’ Follow-up Study (1986–2012).	Investigate the association between long-term changes due to the consumption of sugary drinks (including 100% fruit juices) and drinks containing artificial sweeteners (ASBs) and the consequent risk of DM2.	Over 2,783,210 person-years of follow-up, 11,906 incident cases of DM2 are documented. Increase in total consumption of sugar-sweetened beverages by >0.5 servings per day over four years was associated with a 16% higher risk of DM2 in the following four years. Increasing ASB consumption by > 0.5 servings per day was associated with an 18% higher risk of DM2. Replacing a daily serving of sugary drink with water, coffee or tea was associated with a 2–10% lower risk of DM2.	Increased consumption of sugary drinks or ASB was associated with a higher risk of DM2, although the latter association may be influenced by reverse causality and surveillance biases.		High
Toews I. et al., 2018 [120]	Systematic review and meta-analysis of randomized and non-randomized CT and observational studies	56 studies included (21 RCT, 35 observational studies	/	To assess the association between intake of non-sugar sweeteners (NSSs) and important health outcomes in generally healthy or overweight/obese adults and children.	In adults, evidence of very low and low certainty from a limited number of small studies indicated a small beneficial effect of NSSs on fasting blood glucose (−0.16 mmol/L, −0.26 to −0.06; two, *n* = 52).	Most health outcomes did not seem to have differences between the NSS exposed and unexposed groups. Of the few studies identified for each outcome, most had few participants and were of short duration, and their methodological and reporting quality was limited; therefore, confidence in the reported results is limited.		High
WHO [121]	Guideline from WHO	/	/	Evidence-informed guidance on the use of non-sugar sweeteners to reduce the risk of unhealthy weight gain and diet-related noncommunicable diseases in adults and children.	/	This guideline includes a recommendation on the use of non-sugar sweeteners which can be used by policy-makers and programme managers to address non-sugar sweetener use in their populations through a range of policy actions and public health interventions.		High
ElSayed N.A. et al., 2023 [122]	ADA Standards of Care in Diabetes	/	/	Provide the components of diabetes care, general treatment goals and guidelines, and tools to evaluate quality of care.	/	/		High
Franz J.M. et al., 2017 [123]	Systematic review	60 studies met the inclusion criteria (18 RCTs, 1 nonrandomized clinical study, 3 cohort studies	/	Evidence for medical nutrition therapy effectiveness and recommendations for integration into the nutrition care process.	The systematic review for the Academy’s Nutrition Practice Guideline for Diabetes Type 1 and Type 2 in Adults reviewed 13 subtopics with 19 questions.	Strong evidence supports the effectiveness of medical nutrition therapy/MNT) provided by registered dietitian nutritionists (RDNs) on HbA1c with decreases up to 2.0% at 3 months, and with ongoing MNT support, decreases were maintained or improved long-term.		High
Hedrick V.E. et al., 2023 [124]	Narrative review	/	/	Current evidence on non-sugar sweetener intake is inadequate, and further research is needed to determine the health effects of individual non-sugar sweeteners, especially in specific population subgroups.		Research examining non-sugar sweeteners as a single entity provides unclear findings related to their health effects, especially for obesity, weight management, glycemic control, and other cardiometabolic disease risk factors.		Low

**Table 11:** (**A**) Alcohol and prediabetes interventional studies; (**B**) alcohol and prediabetes observational studies; and (**C**) alcohol and prediabetes review and meta-analysis.

**(A) Alcohol and Prediabetes Interventional Studies**								
**Authors**	**Type of Study**	**Population Characteristics**	**Type of Intervention**	**Duration**	**End Point**	**Results**	**Conclusion**	**Strength of Evidence**
Gepner et al., 2015 [125]	Randomized controlled trial	224 subjects	Three groups: one group to drink 150 mL of mineral water at dinner, another 150 mL of white wine, and, finally, 150 mL of red wine.	Two years	To establish any effects of wine on metabolic and diabetes blood markers.	No significant differences among groups, except for red wine which can reduce the risk of metabolic syndrome.	Red wine in moderate consumption can reduce the risk of metabolic syndrome.	High
**(B) Alcohol and Prediabetes Obervational Studies**								
**Authors**	**Type of Study**	**Population Characteristics**	**Type of Intervention**	**Duration**	**End Point**	**Results**	**Conclusion**	**Strength of Evidence**
Luz P et al., 2014 [126]	Case–control study	T205 people healthy males between the ages of 50 and 75.	Two groups of people were retrospectively compared, 101 people drinking red wine and 104 with complete abstinence from alcohol.	Five years	To evaluate the effects of red wine on glycometabolic patterns.	Drinking one glass of red wine a day has led to better glycometabolic parameters.	Drinking one glass of red wine is beneficial in preventing T2DM.	Moderate
Blomster et al., 2014 [127]	Prospective cohort study	11,140 people with type 2 diabetes mellitus	To define groups based on their alcohol consumption.	Five years	To establish the risk of complications in people with different levels of alcohol consumption.	Those who consumed moderate alcohol saw a reduction in cardiovascular events, fewer micro-vascular complications, and reduced all-cause mortality.	Moderate alcohol consumption is beneficial for all-cause mortality.	Moderate
Li et al., 2022 [128]	Cohort study	15,726 participants	Groups based on daily alcohol consumption.	Four years	To assess the association between the average consumption of alcohol with the incidence of type 2 diabetes mellitus.	Occasional or moderate alcohol use was not associated with risk of hyperglycemia, while high consumption increased the incidence of dysglycemia.	High alcohol consumption is detrimental, while moderate consumption is not associated with risk of dysglycemia.	Moderate
Cullman et al., 2011 [129]	Cohort study	2070 men and 3058 women with normoglycemia and 70 men and 41 women with prediabetes	Groups based on alcohol consumption in quantity and quality.	8–10 years of follow-up	To evaluate the effect on glycometabolic patterns of different types and dosages of alcoholic beverages.	Both women and men had higher risk with heavy alcohol consumption.	Heavy alcohol consumption is detrimental for both sexes.	Moderate
Suebsamran et al., 2016 [130]	Cross-sectional analytical study	383,442 patients	Enrolled were divided into six different groups based on the amount of alcohol consumed.		Relationship between alcohol consumption and prediabetes.	After adjustment for other risk factors, alcohol consumption was independently associated with prediabetes, with dose–response relationship.	The more the alcohol consumption, the more the risk of prediabetes.	Moderate
**(C) Alcohol and Prediabetes Review and Meta-Analysis**								
**Authors**	**Type of Study**	**Number of Studies**	**Subjects (Total)**	**End Point**	**Result**	**Conclusion**		**Strenght of Evidence**
Baliunas et al., 2009 [131]	Meta-analysis	20 cohort studies	477,200 individuals	To establish a relationship between alcohol consumption and diabetes type 2.	Our analysis confirms previous research findings that moderate alcohol consumption is protective for type 2 diabetes in men and women.	Moderate alcohol consumption is beneficial for preventing the risk of diabetes.		High

**Table 12:** (**A**) Coffee and prediabetes interventional and in vitro studies; (**B**) coffee and prediabetes observational studies; and (**C**) coffee and prediabetes reviews and meta-analysis.

**(A) Coffee and Prediabetes Interventional and in Vitro Studies**								
**Authors**	**Type of study**	**Population Characteristics**	**Type of Intervention**	**Duration**	**End Point**	**Results**	**Conclusion**	**Strength of Evidence**
Arion, W.J. et al., 1997 [132]	In vitro study				The interactions of chlorogenic acid (CHL) and 2-hydroxy-5-nitrobenzaldehyde (HNB) with the components of the rat hepatic glucose 6-phosphatase (Glc-6-Pase) system	CHL is without effect on the enzyme of fully disrupted microsomes or the system inorganic pyrophosphatase (PPiase) activity. HNB is a potent competitive inhibitor of the system PPiase activity (Ki = 0.56 mm) and a somewhat weaker noncompetitive inhibitor of enzyme activity (Ki = 2.1 mm).	The presence of CHL effectively protects T1, but not T2, against the irreversible inhibition by HNB. In contrast, PPi and Pi are effective in protecting T2, but not T1.	Low
Yanagimoto, A. et al., 2023 [133]	RCT randomized, double-blind, placebo-controlled crossover study		Study 1 (*n* = 18) evaluated two doses of GTC (270 or 540 mg) containing a fixed dose of CCA (270 mg) with 113 mg of caffeine and a placebo (0 mg GTC and 0 mg CCA) with 112 mg of caffeine. Study 2 (*n* = 18) evaluated two doses of CCA (150 or 300 mg) containing a fixed dose of GTC (540 mg) and a placebo with 99 mg of caffeine.	One year	Examined the effective dose of GTC and CCA on postprandial glucose, insulin, and incretin responses to a high-fat and high-carbohydrate cookie meal containing 75 g of glucose in healthy men.	The single combined ingestion of GTC and CCA significantly altered the incretin response and suppressed glucose and insulin levels.	These findings suggest that the effective minimum dose is 540 mg of GTC and 150 mg of CCA	High
**(B) Coffee and Prediabetes Observational Studies**								
**Authors**	**Type of Study**	**Population Characteristics**	**Type of Intervention**	**Duration**	**End Point**	**Results**	**Conclusion**	**Strength of Evidence**
Sakhi A. et al., 2004 [134]	Cross-sectional study	A group of 61 adults with corresponding plasma samples and data from a nationwide survey of 2672 Norwegian adults based on an extensive FFQ.			To determine the contribution of various food groups to total antioxidant intake, and to assess the correlations of the total antioxidant intake from various food groups with plasma antioxidants.	The total intake of antioxidants was approximately 17 mmol/d with beta-carotene, alpha-tocopherol, and vitamin C contributing <10%. The intake of coffee contributed approximately 11.1 mmol.	Dietary antioxidants other than the well-known antioxidants contribute to our antioxidant defense. Surprisingly, the single greatest contributor to the total antioxidant intake was coffee.	Moderate
**(C) Coffee and Prediabetes Reviews and Meta-Anaylisis**								
**Authors**	**Type of Study**	**Population Characteristics**	**Type of Intervention**	**Duration**	**End Point**	**Results**	**Conclusion**	**Strength of Evidence**
Poole, R. et al., 2017 [135]	Meta-analysis	201 meta-analyses of observational research with 67 unique health outcomes and 17 meta-analyses of interventional research with nine unique outcomes.	Umbrella review of the evidence across meta-analyses of observational and interventional studies of coffee consumption and any health outcome.		To evaluate the existing evidence for associations between coffee consumption and multiple health outcomes.	There was evidence of a non-linear association between consumption and some outcomes, with summary estimates indicating largest relative risk reduction at intakes of three to four cups a day versus none, including all-cause mortality (relative risk 0.83 (95% confidence interval 0.79 to 0.88), cardiovascular mortality (0.81, 0.72 to 0.90), and cardiovascular disease (0.85, 0.80 to 0.90).	Coffee consumption seems generally safe within usual levels of intake, with summary estimates indicating largest risk reduction for various health outcomes at three to four cups a day, and is more likely to benefit health than harm.	High
Ding, M. et al., 2014 [136]	Dose–response meta-analysis	Twenty-eight prospective studies were included in the analysis, with 1,109,272 study participants and 45,335 cases of type 2 diabetes.		The follow-up duration ranged from 10 months to 20 years.	Association of coffee consumption with the risk of type 2 diabetes.	Compared with no or rare coffee consumption, the relative risk (RR; 95% CI) for diabetes was 0.92 (0.90–0.94), 0.85 (0.82–0.88), 0.79 (0.75–0.83), 0.75 (0.71–0.80), 0.71 (0.65–0.76), and 0.67 (0.61–0.74) for 1–6 cups/day, respectively. The RR of diabetes for a 1 cup/day increase was 0.91 (0.89–0.94) for caffeinated coffee consumption and 0.94 (0.91–0.98) for decaffeinated coffee consumption (*p* for difference = 0.17).	Coffee consumption was inversely associated with the risk of type 2 diabetes in a dose–response manner. Both caffeinated and decaffeinated coffee was associated with reduced diabetes risk.	High
Van Dam, R.M., 2008 [137]	Narrative review	/	/	/	This paper briefly reviews the evidence for a relation between coffee consumption and these conditions, with particular attention to methodological issues.	Several early studies suggested that coffee consumption could result in a marked increase in risk of coronary heart disease and several types of cancer. High consumption of unfiltered types of coffee, such as French press and boiled coffee, has been shown to increase low-density-lipoprotein-cholesterol concentrations. In addition, limiting caffeinated coffee intake during pregnancy seems a prudent choice.	In sum, the currently available evidence on coffee and risk of cardiovascular diseases and cancer is largely reassuring, and suggests that, for the general population, addressing other health-related behaviors has priority for the prevention of chronic diseases.	Low
Meng, S. et al., 2013 [138]	Narrative review				Roles of chlorogenic acid on regulating glucose and lipids metabolism.	Chlorogenic acid (CGA) exerts many biological properties, including antibacterial, antioxidant, and anticarcinogenic activities.	The mechanism on glucose and lipid metabolism has not yet been conclusively elucidated.	Low

**Table 13:** (**A**) Physical activity and prediabetes interventional studies; and (**B**) physical activity and prediabetes, review, and meta-analysis.

**(A) Physical Activity and Prediabetes Interventional Studies**								
**Authors**	**Type of Study**	**Population Characteristic**	**Type of Intervention**	**Duration**	**End Point**	**Result**	**Conclusion**	**Strength of Evidence**
Jung M.E. et al., 2015 [139]	RCT	32 inactive individuals with prediabetes between the ages of 30 and 60 years.	15 individuals received HIIT and 17 individuals received MICT one-month follow-up testing.	After completing 10 sessions of supervised training participants were asked to perform HIIT or MICT three times per week for four weeks.	To compare self-report and objective measures of physical activity after one month of independent exercise in individuals with prediabetes who were randomized to HIIT (*n* = 15) or traditional moderate-intensity continuous training (MICT, *n* = 17).	Individuals in HIIT (89 ± 11%) adhered to their prescribed protocol to a greater extent than individuals in MICT (71 ± 31%). Minutes spent in vigorous physical activity per week measured by accelerometer were higher in HIIT (24 ± 18) as compared to MICT (11 ± 10) at one-month follow-up (*p* = 0.049, Cohen’s d = 0.92). Cardiorespiratory fitness and systolic blood pressure assessed at one-month follow-up were equally improved (*p* < 0.05).	This study provides preliminary evidence that individuals with prediabetes can adhere to HIIT over the short term and do so at a level that is greater than MICT.	High
**(B) Physical Activity and Prediabetes, Review, and Meta-Analysis**								
**Authors**	**Type of Study**	**Number of Studies**	**Subjects**	**End Point**	**Results**	**Conclusion**		**Strength of Evidence**
Snowling N.J. et al., 2006 [140]	Meta-analysis	27 eligible studies	1003 type 2 diabetic patients (age 55 +/− 7 years) over 5–104 weeks.	Meta-analyze the effects of different modes of exercise training on measures of glucose control and other risk factors for complications of diabetes.	Differences among the effects of aerobic, resistance, and combined training on HbA(1c) (A1C) were trivial; for training lasting ≥ 12 weeks, the overall effect was a small beneficial reduction (A1C 0.8 +/− 0.3% [mean +/− 90% confidence limit]). There were generally small to moderate benefits for other measures of glucose control. Effects of covariates were generally trivial or unclear, but there were small additional benefits of exercise on glucose control with increased disease severity.	All forms of exercise training produce small benefits in the main measure of glucose control: A1C. The effects are similar to those of dietary, drug, and insulin treatments. The clinical importance of combining these treatments needs further research.		High
De Nardi A.T. et al., 2018 [141]	Meta-analysis	818 eligible studies	Seven studies were included in systematic review (64 prediabetes and 120 T2D patients) and five with T2D were meta-analyzed.	To compare the effects of high-intensity interval training (HIIT) versus moderate-intensity continuous training (MICT) on functional capacity and cardiometabolic markers.	No differences were found between two modalities of exercises considering the outcomes HbA1c, systolic and diastolic blood pressure, total cholesterol, HDL and LDL cholesterol, triglycerides, BMI, and waist-to-hip ratio. Most of the studies presented unclear risk of bias, and low and very low quality of evidence.	HIIT induces cardiometabolic adaptations similar to those of MICT in prediabetes and T2D, and provides greater benefits to functional capacity in patients with T2D.		High
Jadhav R.A. et al., 2021 [142]	Systematic review and meta-analysis of randomized controlled trials	1688 citations, 31 full-text articles assessed for eligibility of inclusion. Nine studies satisfied the pre-specified criteria for inclusion.	1906 participants with the age group ranging from 20 to 70 years. All participants were diagnosed by either WHO or ADA.	Strengthen the evidence on the impact of physical activity promotion on inflammatory markers in prediabetes considering that the inflammatory stage in prediabetes is associated with increased levels of adipokines and pro-inflammatory cytokines.	Meta-analysis found that physical activity with or without dietary or lifestyle modification reduces level of leptin (MD −2.11 ng/mL, 95% CI −3.81–−0.42) and interleukin-6 (MD −0.15 pg/mL, 95% CI −0.25–−0.04). It has no effect on level of adiponectin (MD 0.26 µg/mL, 95% CI −0.42–0.93), C-reactive protein (MD −0.05 mg/L, 95% CI −0.33–0.23), and tumour necrosis factor-α (MD 0.67 pg/mL, 95% CI −2.56–3.89).	Physical activity promotion with dietary and lifestyle modification may reduce the level of leptin and interleukin-6, but we are uncertain if there is any effect on levels of adiponectin, C-reactive protein, and tumour necrosis factor-α in the individuals with prediabetes.		High

**Table 14:** (**A**) Dietary supplementation with probiotics and prediabetes observational studies; and (**B**) dietary supplementation with probiotics and prediabetes reviews and meta-analyses.

**(A) Dietary Supplementation With Probiotics and Prediabetes Observational Studies**								
**Authors**	**Type of Study**	**Population Characteristics**	**Type of Intervention**	**Duration**	**End Point**	**Results**	**Conclusion**	**Strength of Evidence**
Qin J. et al., 2012 [143]	Case–control study	123 patients with metabolic syndrome (MetS) and 304 controls	Whole-genome shotgun sequencing was used and approximately 60,000 type-2-diabetes-associated markers was identified		To determine whether the gut microbiome plays a role in MetS development and progression	Gut microbiomes of MetS patients possess significantly lower gut microbiome diversity. Microbiome changes in MetS patients may aggravate inflammation and contribute to MetS diseases by inhibiting the production of short-chain fatty acids (SCFAs).	Potential utility of beneficial gut microbiota as a potential therapeutic to alleviate MetS.	Moderate
Larsen N. et al., 2010 [144]	Case–control study	36 male adults with different ages and body-mass indices (BMIs), including 18 subjects with diabetes type 2 (DT2)	The fecal bacterial composition was investigated by real-time quantitative PCR (qPCR) and in a subgroup of subjects (*n* = 20) by tag-encoded amplicon pyrosequencing of the V4 region of the 16S rRNA gene.		To assess the differences between the composition of the intestinal microbiota in humans with type 2 diabetes and non-diabetic persons	The proportions of phylum Firmicutes and class Clostridia were significantly reduced in the diabetic group compared to control group. Bacteroidetes/Firmicutes ratios, as well as Bacteroides/Prevotella ratios, correlated positively and significantly with plasma glucose concentration but not with BMIs. Class Betaproteobacteria was highly enriched in diabetic compared to non-diabetic persons (*p* = 0.02)	DT2 in humans is associated with compositional changes in intestinal microbiota.	Moderate
Tang W.H.W. et al., 2017 [145]	Prospective cohort study	Two cohorts: the first cohort comprises 1216 patients with T2DM, second cohort comprises 300 apparently healthy individuals ≥21 years old	Examination of relationship between fasting TMAO and two of its nutrient precursors, choline and betaine, vs. three-year major adverse cardiac events and five-year mortality	Five-year period	To study prognostic value of TMAO concentrations that are increased in T2DM patients and their relation to glycemic control.	TMAO and choline concentrations were higher in individuals with T2DM vs. healthy controls. Within T2DM patients, higher plasma TMAO was associated with a significant 3.0-fold increased three-year major adverse cardiac event risk and a 3.6-fold increased five-year mortality risk.	Fasting plasma concentrations of the proatherogenic-gut-microbe-generated metabolite TMAO are higher in T2DM patients and are independent risk factor for major adverse cardiac events and mortality risks.	Moderate
Li Y. et al., 2015 [146]	Prospective cohort study	203,308 men and women extracted from three cohorts: the Nurses’ Health Study (NHS), NHS II, and the Health Professionals Follow-Up Study (HPFS).	Dietary phosphatidylcholine was estimated by a food-frequency questionnaire	Four years	To study the association between dietary phosphatidylcholine and risk of type 2 diabetes (T2D).	7063, 4465, and 3531 cases of T2D (during NHS, NHS II, and HPFS, respectively) were documented. Compared with people in the lowest quintiles of dietary phosphatidylcholine intakes, the RR of T2D for those in the highest quintiles was 1.36 in NHS, 1.35 in NHS II, 1.28 in HPFS, and 1.34 in the pooled analysis. The association was 1.24 after further adjustment for the three major food sources (red meat, eggs, and seafoods) and 1.27 with all choline-containing components and betaine mutually adjusted. With an increase of 100 mg choline from phosphatidylcholine, the risk of T2D increased by 17%.	Dietary intakes of phosphatidylcholine are associated with incident T2D risk.	Moderate
Roy S. et al., 2020 [147]	Longitudinal cohort study	300 diabetes-free men and women (77%) aged 20–55 years (mean = 34 ± 10)	Multivariable generalized linear models regressed; (i) FPG change (year 2 minus baseline) on baseline TMAO tertiles; and (ii) HOMA-IR and HbA1c on TMAO tertiles. Multivariable relative risk regressions modeled prevalent prediabetes across TMAO tertiles.	2 years	To investigate the association between TMAO and biomarkers of diabetes risk.	Mean values of two-year longitudinal FPG ± SE across tertiles of TMAO were 86.6 ± 0.9, 86.7 ± 0.9, and 86.4 ± 0.9 (*p* = 0.98). Trends were null for FPG, HbA1c, and HOMA-IR, cross-sectionally. The prevalence ratio of prediabetes among participants in second and third TMAO tertiles (vs. the 1st) were 1.94 [95% CI 1.09–3.48] and 1.41 [95% CI: 0.76–2.61].	TMAO levels are associated with increased prevalence of prediabetes in a nonlinear fashion but not with insulin resistance or longitudinal FPG change.	Moderate
Allin K.H. et al., 2018 [148]	Case–control study	134 Danish adults with prediabetes, overweight, insulin resistance, dyslipidemia, and low-grade inflammation and 134 individuals with normal glucose regulation.	Comparison of biochemical markers, anthropometric parameters, and gut microbiota in control and case groups.	12 weeks	To test whether specific gut microbiota profiles are associated with prediabetes.	Five bacterial genera and 36 operational taxonomic units (OTUs) were differentially abundant between individuals with prediabetes and those with normal glucose regulation. At the genus level, the abundance of Clostridium was decreased, whereas the abundances of Dorea, Sutterella and Streptococcus were increased. The two OTUs that differed the most were members of the order Clostridiales and Akkermansia muciniphila, which both displayed lower abundance among individuals with prediabetes.	Individuals with prediabetes have intestinal microbiota characterized by a decreased of the genus Clostridium and the mucin-degrading bacterium A. muciniphila. These findings are comparable to observations in overt chronic diseases characterized by low-grade inflammation.	Moderate
Zhong H. et al., 2019 [149]	Cohort study	77 type 2 diabetic individuals who are treatment-naïve, 80 pre-diabetic individuals, and 97 normal glucose-tolerant individuals.	Combination of in-depth metagenomics and metaproteomics analyses on fecal samples		To investigate compositional and functional changes of the gut microbiota and the fecal content of microbial and host proteins in preDM and treatment-naïve T2D individuals	Distinct differences characterizing the gut microbiota of the three groups were observed. The content of several human antimicrobial peptides and pancreatic enzymes differed in fecal samples between three groups.	There is a complex, disease-stage-dependent interplay between the gut microbiota and the host.	Moderate
**(B) Dietary Supplementation with Probiotics and Prediabetes Reviews and Meta-Analyses**								
**Authors**	**Type of Study**	**Number of Studies**	**Subjects (Total)**		**End Point**	**Result**	**Conclusion**	**Strenght of Evidence**
Letchumanan G. et al., 2022 [150]	Systematic Review of Observational Studies	18 eligible studies	5489 participants		To summarize the existing evidence related to microbiota composition and diversity in individuals with prediabetes (preDM) and individuals newly diagnosed with T2DM (newDM) in comparison to individuals with normal glucose tolerance (nonDM).	4 out of the 18 studies found increased abundance of phylum Firmicutes along with decreased abundance of Bacteroidetes in newDM. At the genus/species levels, decreased abundance of *Faecalibacterium prausnitzii*, *Roseburia*, *Dialister*, *Flavonifractor*, *Alistipes*, *Haemophilus,* and *Akkermansia muciniphila* and increased abundance of *Lactobacillus*, *Streptococcus*, *Escherichia*, *Veillonella,* and *Collinsella* were observed in the disease groups in at least two studies. Lactobacillus was also found to positively correlate with fasting plasma glucose (FPG), HbA1c, and/or homeostatic assessment of insulin resistance (HOMA-IR) in four studies.	There is a need for further investigations on the species/strain-specific role of endogenously present Lactobacillus in the glucose regulation mechanism and T2DM disease progression and more studies are needed to establish more consistent associations, between clinical biomarkers or dietary intake and specific gut bacterial composition in prediabetes and early T2DM.	High
Gurung M. et al., 2020 [151]	Systematic review	42 human studies	Not available		To study the potential role of different bacterial taxa affecting diabetes and to discuss potential molecular mechanisms of microbiota effects in the onset and progression of T2D.	The genera of *Bifidobacterium*, *Bacteroides*, *Faecalibacterium*, *Akkermansia,* and *Roseburia* were negatively associated with T2D, while the genera of *Ruminococcus*, *Fusobacterium*, and *Blautia* were positively associated with T2D.	Some microbial taxa and related molecular mechanisms may be involved in glucose metabolism related to T2D. However, the heterogeneity of T2D and redundancy of gut microbiota do not promise simple interpretations (e.g., low diversity) and easy solutions (such as fecal transplant from non-diabetic/non-obese donor).	High
Barengolts E., 2016 [152]	Review of randomized controlled trials	Not available	Not available		To review the data from randomized controlled trials (RCTs) for the roles of microbiota, pre-, pro-, and synbiotics in metabolic conditions (obesity, prediabetes, and diabetes mellitus type 2 [DM2]).	Results of RCTs of prebiotics suggested a neutral effect on body weight, decreased fasting and postprandial glucose, and improved insulin sensitivity and lipid profile. Some inflammation markers were reduced, sometimes substantially (20–30%). The effect was seen mostly with fermented milk or yogurt compared to capsule form, consumption for at least eight weeks, and use of multiple rather than a single bacterial strain. Changes in microbiota were seen at times with both pre- and probiotics. Pickled and fermented foods, particularly vegetables and beans, could serve as a dietary source of pre-, pro-, and synbiotics.	Pre-, pro-, and synbiotics could prove useful, but further research is needed to clarify their clinical relevance for the prevention and management of metabolic disease.	High
Wang X. et al., 2021 [153]	Systematic review of randomized controlled trials	Eight randomized controlled trials.	391 Participants		To identify evidence for microbiota’s role and use of probiotics, pre-biotics, or synbiotics in prediabetes.	Probiotics can decrease glycated hemoglobin (HbA1c) and have the potential to improve post-load glucose levels. Pre-biotics failed to show an evident improvement in glycemic control, but their use caused the changes in the composition of gut microbiota. A combination of probiotics and prebiotics in the synbiotic supplementation is more effective than probiotics alone in glycemic control.	Using probiotics, pre-biotics, or synbiotics for the treatment of prediabetes, the benefits of modulating the abundance of gut microbiota were partially demonstrated. However, there is insufficient evidence to show significant benefits on glucose metabolism.	High
Li Y. et al., 2022 [154]	Systematic review and meta-analysis	Seven publications	460 patients		To examine the effects of probiotics on eight factors in the prediabetic population.	Probiotics were able to significantly decrease the levels of glycated hemoglobin A1c (HbA1c), quantitative insulin sensitivity check index (QUICKI), total cholesterol (TC), triglyceride (TG), and low-density lipoprotein cholesterol (LDL-C) compared to levels in the placebo group.	Probiotics may fulfil an important role in the regulation of HbA1c, QUICKI, TC, TG, and LDL-C in patients with prediabetes. In addition, probiotics may regulate blood glucose homeostasis in a variety of ways.	High
Zeighamy Alamdary S. et al., 2022 [155]	Systematic review of randomized controlled trials	15 articles	1295 patients		To compile the results of clinical trials investigating the effects of pro-/pre-/synbiotics on prediabetes subjects from 2010 to 2020.	Different probiotics compositions have shown beneficial and noticeable effects on glucose homeostasis, lipid profiles, BMI, and inflammatory markers in subjects with prediabetes, metabolic syndrome, and healthy individuals.	Administration of probiotics may provide beneficial and healthful effects in the clinical management of patients with prediabetes and metabolic syndrome and could be advantageous in recomposing the gut microbiota back into the normal state during the prediabetic state.	High
Bock P.M. et al., 2021 [156]	Systematic review and meta-analysis of randomized controlled clinical trials	130 articles for review, 38 of which included in the meta-analysis	2086 participants		To assess the effect of probiotic, prebiotic, or synbiotic supplementation on gut microbiota and glucose control and lipid levels in individuals with diabetes.	The use of prebiotics, probiotics, or synbiotics reduced HbA1c levels, but did not reach the threshold for significance and had no effect on LDL-cholesterol levels. However, their consumption decreased levels of fasting blood glucose, total cholesterol, triacylglycerols, and insulinemia, and increased HDL-cholesterol levels.	In individuals with diabetes mellitus, supplementation with probiotics, prebiotics, or synbiotics improved metabolic variables, although the magnitude of this effect is low.	High
Zhang Q. et al., 2015 [157]	Meta-analysis of randomized controlled trials	Seven trials	386 participants		To investigate the effects of probiotics on glucose metabolism in patients with type 2 diabetes mellitus.	Probiotic consumption significantly changed FPG by −15.92 mg/dL and HbA1cby −0.54% compared with control groups. Subgroup analysis was conducted in to trials with non-yogurts control. Meta-analysis of trials with multiple species of probiotics found a significant reduction in FPG. The duration of intervention for ≥8 weeks resulted in a significant reduction in FPG. The results also showed that probiotic therapy significantly decreased homeostasis model assessment of insulin resistance (HOMA-IR) and insulin concentration.	Consuming probiotics may improve glucose metabolism by a modest degree, with a potentially greater effect when the duration of intervention is ≥8 weeks, or multiple species of probiotics are consumed.	High

**Table 15:** (**A**) Monitoring glucose levels and prediabetes interventional studies; (**B**) monitoring glucose levels and prediabetes observational studies; and (**C**) monitoring glucose levels and prediabetes reviews and meta-analyses.

**(A) Monitoring Glucose Levels and Prediabetes Interventional Studies**								
**Authors**	**Type of Study**	**Population Characteristics**	**Type of Intervention/Exposure**	**Duration**	**End Point**	**Results**	**Conclusion**	**Strength of Evidence**
Pierre K.B. et al. [158]	Randomized controlled trial	1548 patients receiving mechanical ventilation admitted to the surgical intensive care.	A total of 1548 critically ill surgical intensive care unit patients were randomly assigned to receive either intensive insulin therapy (BG 80–110 mg/dL) or conventional treatment (initiation of insulin infusion for BG greater than 215 mg/dL and maintenance of BG between 180 and 200 mg/dL).	2 February 2000 and 18 January 2001	To determine whether intensive insulin therapy to normalize blood glucose levels (BG) reduces mortality and morbidity among critically ill patients.	In the intensive insulin therapy group, BG was maintained between 80 and 110 mg/dL. Overall, 98.7% of the intensive treatment group required insulin based on this algorithm. Of the conventional treatment group, 39.2% of patients received insulin therapy with maintenance of BG at a mean of 173 ± 33 mg/dL.	Maintenance of normoglycemia (80–110 mg/dL) with intensive insulin therapy reduces both mortality and morbidity associated with critical illness.	High
Zhang Z. et al., 2019 [159]	Experimental study	12 adults (10 completed).	The photos of the patches were used for the determination of sweat loss and pH level. The sweat patches were then placed in Petri dishes and air-dried before a second photograph was taken to capture the color intensity on the detection zones.	/	They introduce a versatile, cost-effective, flexible, and wearable POC biomarker patch for effective sweat collection and health monitoring.	It was observed glucose concentrations between 4.7 and 98.4 μM for eight of the subjects, which is in the normal range of sweat glucose (<120 μM). Subject 2 and 10 have a relatively high sweat glucose concentration of 163.5 μM and 336.1 μM, respectively. The high glucose readout in subject 10, corrected by the sweat volume, could potentially indicate a prediabetic or diabetic condition for the subject.	The device can detect sweat loss, pH, glucose concentrations, and lactate concentrations in sweat, with the ability to detect glucose levels in the physiological range of 50–300 μM.	Low
Cui Y. et al., 2021 [160]	Randomized controlled trial	A total amount of six saliva collection methods were employed in 80 healthy participants in the morning.	/	/	To identify the ideal saliva collection method	The improved method obtained absorbance at the wavelength of 520 nm, and the optimized parameter combination was pH 6.5 and 5 mg/dL NaCl. The lower limit of glucose detection was 0.1 mg/dL. Unstimulated saliva glucose concentration was higher than stimulated saliva glucose concentration. Unstimulated parotid saliva glucose concentration was the highest. Moreover, unstimulated saliva glucose has a better normal distribution effect. Meantime, it was found that unstimulated parotid saliva was the most highly correlated with blood glucose (R2 = 0.707).	The saliva collection method was an important factor that affected saliva glucose concentration. Unstimulated parotid saliva was the most highly correlated with blood glucose, which provided a reference for the prediction of diabetes mellitus.	High
Cui Y. et al., 2022 [161]	Randomized controlled trial	40 age-matched DM patients and 40 healthy controls.	The correlations between salivary glucose and blood glucose before and after breakfast were analyzed.	/	This study aims to identify an ideal saliva collection method and to use this method to determine the population and individual correlations between salivary glucose and blood glucose levels in DM patients and healthy controls.	Compared with unstimulated saliva, stimulated saliva had decreased glucose level and increased salivary flow. In addition, unstimulated parotid salivary glucose was most correlated with blood glucose level (R2 = 0.9153), and the ROC curve area was 0.9316, which could accurately distinguish DM patients.	The unstimulated parotid salivary glucose before breakfast presents an ideal saliva collecting method with which to replace blood-glucose use to detect DM, which provides a reference for the prediction of DM.	High
**(B) Monitoring Glucose Levels and Prediabetes Observational Studies**								
**Authors**	**Type of Study**	**Population Characteristics**	**Type of Intervention/Exposure**	**Duration**	**End Point**	**Results**	**Conclusion**	**Strength of Evidence**
Schaffzin J.K. et al. 2012 [162]	Retrospective cohort study	Any resident with diabetes living in the assisted-living facilities between August 2006 and January 2007 (34 residents with diabetes and 12 epidemiologically linked residents). A case was defined as any member of the cohort with serologic evidence of current or previous HBV infection. Diabetes was identified based on notation in the medical record.	/	/	HBV transmission in patient during assisted monitoring of blood glucose (AMBG) and implemented preventive measures.	Serologic testing detected six residents with diabetes with current HBVI and four residents with diabetes and one epidemiologically linked resident with previous HBVI.	AMBG was significantly associated with HBVI in ALF residents with diabetes.	Medium
Han B. et al. 2021 [163]	Cross-sectional comparative study	408 patients with diabetes and 408 people without diabetes randomly matched 1:1.	Venous blood was collected for HBV serological testing and blood glucose testing.	/	Comparison of hepatitis B surface antigen (HBsAg) positive rates between the two groups.	HBsAg positive rate in people without diabetes was 2.0% and in those with diabetes was 4.2%. Whether in people without diabetes or patients with diabetes, higher frequency of SMBG was associated with higher HBsAg positive rate. Increases in the duration of diabetes were correlated with increasing rates of HBsAg.	Routine blood glucose monitoring at home was associated with HBV infection, which meant people with diabetes may be at high risk of HBV infection.	Medium
Finney S.J. et al. 2003 [164]	Single-center, prospective, observational study	531 patients (median age, 64 years).	/	Median lengths of stay were 1.8 (interquartile range, 0.9–3.7) days and 6 (interquartile range, 4.5–8.3) days, respectively.	Intensive care unit (ICU) mortality.	Of 531 patients admitted to the ICU, 523 underwent analysis of their glycemic control. Twenty-four–hour control of blood glucose levels was variable. Rates of ICU and hospital mortality were 5.2% and 5.7%, respectively.	Increased insulin administration is positively associated with death in the ICU regardless of the prevailing blood glucose level. Thus, control of glucose levels rather than of absolute levels of exogenous insulin appear to account for the mortality benefit associated with intensive insulin therapy demonstrated by others.	Medium
Evans J.M. et al. 1999 [165]	Retrospective study	Patients resident in Tayside in 1993–1995 who were using insulin and were registered on the database and diagnosed with insulin-dependent (type 1) or non-insulin-dependent (type 2) diabetes before 1993.			Investigate patterns of self-monitoring of blood glucose concentration in diabetic patients who use insulin and to determine whether frequency of self-monitoring is related to glycemic control.	Among 807 patients with type 1 diabetes, 128 (16%) did not redeem any prescriptions for glucose-monitoring reagent strips in the three-year study period. Only 161 (20%) redeemed prescriptions for enough reagent strips to test glucose daily. The corresponding figures for the 790 patients with type 2 diabetes who used insulin were 162 (21%; no strips) and 131 (17%; daily tests).	Self-monitoring of blood glucose concentration is associated with improved glycemic control in patients with type 1 diabetes. Regular self-monitoring in patients with type 1 and type 2 diabetes is uncommon.	Medium
Berghe G. et al. 2003 [166]	Prospective randomized controlled trial	1548 patients	Subjects randomly assigned to either strict normalization of blood glucose (80–110 mg/dL) with insulin infusion or the conventional approach, in which insulin is only given to maintain blood glucose levels at 180–200 mg/dL.	12 days	Report the factors determining insulin requirements and the impact of insulin dose vs. blood glucose control on the observed outcome benefits.	The lowered blood glucose level rather than the insulin dose was related to reduced mortality (*p* < 0.0001), critical illness polyneuropathy (*p* < 0.0001), bacteremia (*p* = 0.02), and inflammation (*p* = 0.0006) but not to prevention of acute renal failure.	Normoglycemia was safely reached within 24 hrs and maintained during intensive care by using insulin titration guidelines.	High
Sørgård, B. et al. 2019 [167]	Cross-sectional study	23 adults with type 1 diabetes, including current and former users of continuous glucose monitoring from four different outpatient clinics.	/	/	To describe positively and negatively perceived situations experienced by adults with type 1 diabetes using continuous glucose monitoring and the actions they take to deal with these situations.	The participants described that they felt that the use of continuous glucose monitoring was a balance between benefits and barriers, and how, through their actions, they tried to adapt their use of continuous glucose monitoring to fit their lifestyles.	Continuous glucose monitoring is perceived as an effective and important tool in the self-management of diabetes type 1. It enables a better everyday life and increased satisfaction with treatment.	Medium
Hilliard M.E. et al. 2019 [168]	Cross-sectional study	Transcripts from semi-structured qualitative interviews with 55 parents of children aged 1 to <8 years, with T1D duration ≥6 months, and whose child currently or previously used continuous glucose monitoring (CGM).	/	/		Parents described benefits of CGM use: decreased worry about glucose excursions, improved sleep, increased sense of safety with children who cannot recognize or express symptoms of hypo- or hyperglycemia, and greater comfort with other caregivers.	CGM may address unique challenges of T1D in young children and increase parental comfort with diabetes management, yet there are multiple barriers to initiating or maintaining CGM use.	Medium
Engler R. et al. 2018 [169]	Cross-sectional study	People with diabetes and the parents of children with diabetes (*n* = 1348).	Results from two surveys regarding continuous glucose monitoring (CGM) devices.	/	The importance of the concept of “user burden” in patients’ and caregivers’ evaluations of the acceptability of available devices.	/	Minimizing system obtrusiveness will likely be of significant value in reducing hurdles to CGM device use and adherence.	Medium
Diabetes Care 2022 [2]	Position statement						SMBG is recommended for all patients who use insulin. Recommendations for testing urine for glucose and ketones as part of diabetes management are described here.	Low
Hayford J.T. et al., 1983 [170]	Cross-sectional study	24 diabetic subjects (15 female and 9 male); all subjects had type I diabetes, with the exception of one subject (insulin-treated type II diabetic patient).	Subjects were maintained on their previous insulin regimens with no attempt to optimize blood glucose control.	/	Correlation between mean plasma glucose concentration with simultaneous urine glucose concentration on excretion rate.	Both the urine glucose concentration and urine glucose excretion rate are significantly correlated (*p* < 0.0001).	Observations on the correlation between mean plasma glucose concentration with simultaneous urine glucose concentration or excretion rate re-emphasize the limitations of this approach.	Medium
Morris L.R., 1981 [171]	Cross-sectional study	246 adult diabetics	Reported levels from 400 s-voided urines was compared to simultaneous plasma determinations.	/	Determine whether semi-quantitative glucose measurements of spot urine specimens accurately reflect prevailing plasma glucose levels.	Quantitative urine levels and plasma glucose levels correlated. However, when semi-quantitative urinary determinations were compared to plasma glucose stratified into 0 to 149, 150 to 199, and greater than 200 mg/dL, 75% of the urine samples associated with plasma levels from 150 to 199 mg/dL were negative by Diastix, and 16.5% of samples negative by Diastix were in the 200+ mg/dL plasma range.	Except for the detection of marked hyperglycemia, spot urine glucose determinations are inadequate as the sole means of clinical assessment for the management of diabetic patients.	Medium
**(C) Monitoring Glucose Levels and Prediabetes Reviews and Meta-Analyses**								
**Authors**	**Type of Study**	**Numbers of Studies**	**Population Characteristics**	**Type of Intervention/Exposure**	**End Point**	**Results**	**Conclusions**	**Strenght of Evidence**
Malanda U.L. et al., 2012 [172]	Systematic review	Twelve randomized controlled trials were included and evaluated outcomes in 3259 randomized patients.	Nine trials compared SMBG with usual care without monitoring, one study compared SMBG with SMUG, one study was a three-armed trial comparing SMBG and SMUG with usual care, and one study was a three-armed trial comparing less intensive SMBG and more intensive SMBG with a control group. Seven out of eleven studies had a low risk of bias for most indicators.	Intervention duration ranged from 6 months (26 weeks) to 12 months (52 weeks).	To assess the effects of SMBG in patients with type 2 diabetes mellitus who are not using insulin.	/	When diabetes duration is over one year, the overall effect of self-monitoring of blood glucose on glycemic control in patients with type 2 diabetes who are not using insulin is small up to 6 months after initiation and subsides after 12 months.	High
Ilea A. et al., 2019 [173]	Systematic review	49 papers selected	/	/	To present the development in the biosensors research and their utility using salivary assessment.	The 49 papers selected for the present review focused on assessing the salivary biomarkers used in general diseases, oral pathologies, and pharmacology. The biosensors proved to be reliable tools for measuring the salivary levels of biochemical metabolic compounds such as glucose, proteinases and proteins, heavy metals and various chemical compounds, micro-organisms, oncology markers, drugs, and neurotransmitters.	Saliva is a biofluid with a significant clinical applicability for the evaluation and monitoring of a patient’s general health. Biosensors designed for assessing a wide range of salivary biomarkers are emerging as promising diagnostic or screening tools for improving the patients’ quality of life.	High

## 4. Discussion

### 4.1. Carbohydrate-Containing Foods

Carbohydrates are an easily used source of energy and represent the main dietary factor influencing blood sugar, especially postprandial. Carbohydrate-containing foods, with varying proportions of sugars, starches, and fibers, have a wide range of effects on the glycemic response. Some cause a sustained rise and slow fall in blood glucose concentrations, while others cause a rapid rise followed by a rapid fall. A recent consensus report identified carbohydrate foods that are rich in dietary fiber, vitamins, and minerals, and low in added sugar, fat, and sodium as ideal [19].

Whole-grain carbohydrates with a low glycemic index are, therefore, the ideal foods to recommend for people with prediabetes. Moreover, these foods are the carbohydrates present in the Mediterranean diet which, as reported in numerous studies, represents a dietary pattern that can be considered a reference for the treatment of prediabetes. Indeed, the Mediterranean diet, in a prospective cohort study in the Spanish primary care setting that examined a total of 1184 participants with prediabetes for an average of 4.2 years, demonstrated that a high adherence to a Mediterranean diet compared to a low/medium adherence would reduce the incidence of T2DM [28].

A previous prospective study conducted in Greece showed similar results [29].

Considering potatoes among carbohydrates, potatoes eaten in their skins can represent a valid food to be included in the group of carbohydrates for nutrition in subjects with prediabetes. Potatoes are a rich and bio-available source of potassium, dietary fiber, and other key nutrients, such as magnesium, that may benefit cardiometabolic health [174].

Considering meal frequency, there are no specific studies in subjects with prediabetes, but the consensus statement by the American Association of Clinical Endocrinologists and American College of Endocrinology on the Comprehensive Type 2 Diabetes Management Algorithm—2020 Executive Summary reports the importance of consuming five meals a day (breakfast, lunch, and dinner with two snacks) in order to avoid hypo- and hyperglycemia [175].

In conclusion, three portions per day of whole-meal carbohydrates with a low glycemic index or potatoes with their skins, therefore, represent the ideal foods to recommend for subjects with prediabetes.

#### Low-Carbohydrate Diets for the Treatment of Obese Patients with Prediabetes

The consensus report “Therapy for Adults with Diabetes or Prediabetes”, published in 2019, highlighted that individuals with prediabetes should be referred for an intensive behavioral lifestyle intervention program modelled on the DPP (diabetes prevention program) and/or individualized MNT (medical–nutritional therapy) typically provided by a professional, with the aim of improving eating habits, increasing moderate-intensity physical activity to at least 150 min per week, and achieving and maintaining a 7–10% initial body weight loss if necessary [19].

With regard to weight loss in subjects with obese prediabetes, the evidence of scientific research shows that, in this situation, it may be useful to consider a low-carbohydrate diet.

Results from a randomized (1:1) parallel group study suggest that adults with prediabetes or noninsulin-dependent T2DM and obesity may be able to improve glycemic control with fewer medications by following a ketogenic diet at low-carbohydrate libitum (LCK) versus a low-fat, low-calorie, moderate-carb (MCCR) diet.

There were 34 patients involved, 16 who followed the LCK and 18 who followed the MCCR; at 12 months, the participants in the LCK group lost more weight than the participants in the MCCR group. On average, at 12 months, participants in the LCK group lost 8.3% of body weight, while the MCCR group lost 3.8%. Moreover, at 12 months, participants in the LCK group reduced their HbA1c by greater levels than participants in the MCCR group; the largest reductions in HbA1c in the LCK group occurred despite a greater reduction in the use of glucose-lowering medications [25].

A further randomized and balanced study divided the participants into two groups: the first (consisting of 18 participants) followed a low-calorie, medium-carbohydrate, low-fat, carbohydrate-count (MCCR) diet consistent with the guidelines of the American Diabetes Association. The second group (consisting of 16 participants) instead followed a low-carbohydrate, high-fat normocaloric diet, the goal of which was to induce nutritional ketosis (LCK). Participants were all at least 18 years old, diagnosed with T2DM (HbA1c ≥ 6.5%) or prediabetes with an HbA1c greater than 6.0% and a body mass index (BMI) of at least 25 kg/m^2^; the study lasted three months.

A key finding of this study was that a low-carbohydrate diet was more effective than a standard and moderate carbohydrate diet in reducing HbA1c at three months. All individuals in the LCK group showed a decrease in HbA1c, and this was a statistically significant advantage over the percentage of people in the MCCR group whose HbA1c decreased. The proportion of people who achieved a decrease in HbA1c of at least 0.5% was more than double in the LCK (56% vs. 22%), which, again, was statistically significant [26].

Moreover, another randomized cross-over and interventional study also compared two low-carbohydrate diets, both involving the consumption of non-starchy vegetables, the avoidance of added sugars, and the use of refined grains. The main differences between the two diets concerned legumes, whole grains, and fruit; in fact, the diet called WFKD (well-formulated ketogenic diet) avoided the use of legumes, fruit, and whole grains, while the diet named Med-Plus (Mediterranean-plus diet) encouraged their use. Legumes, fruits, and whole grains are consistently recommended by national and international public health organizations based on extensive evidence of the cardiovascular benefits of fiber, antioxidants, and vitamins and minerals characteristic of these food groups.

There were 40 enrolled participants, all over 18 years old, with a diagnosis of prediabetes (HbA1c 5.7–6.4% or fasting glucose 100–125 mg/dL) or T2DM (HbA1c ≥ 6.5% or fasting glucose ≥126 mg/dL).

The two types of diet were randomly assigned, and both were followed for 12 weeks each by the enrolled patients.

The primary outcome of the study was to investigate the percent change in HbA1c after 12 weeks of each diet. Secondary and exploratory outcomes included percentage changes in body weight, fasting insulin, blood glucose, and lipids; average glucose from continuous glucose monitoring (CGM); and nutrient intake.

The primary analysis was for 33 participants with complete data. HbA1c values did not differ between diets at 12 weeks. Triglycerides decreased the most for WFKD [percentage changes, −16% vs. −5% for Med-Plus; *p* = 0.02] and LDL cholesterol was higher for WFKD [percentage changes, +10% versus −5% for Med-Plus; *p* = 0.01]. Weight decreased 8% versus 7% and HDL cholesterol increased 11% versus 7% for WFKD versus Med-Plus, respectively. However, there was a significant diet order interaction for both. Participants had lower intakes of fiber and three nutrients on WFKD compared to Med-Plus. The twelve-week follow-up data suggest that Med-Plus is more sustainable.

This study confirmed that HbA1c levels can be significantly reduced with both a ketogenic diet and a Mediterranean diet, as long as the diet is limited in added sugars and refined carbohydrates. The study’s primary question was whether people with an impaired glucose metabolism experienced greater metabolic benefits or harm when limiting whole and intact legumes, fruits, and grains. in addition to avoiding added sugars and refined grains.

Although carbohydrate reduction is often recommended as a strategy to control blood glucose in patients with prediabetes and T2DM, the metabolic benefits and risks of extreme versus moderate carbohydrate restriction are unclear. The authors found that both WFKD and Med-Plus were associated with improved blood sugar and reduced body weight due to a reduction in added sugars and refined sugars. Although WFKD was associated with better glucose control, differences in HbA1c values were modest despite the 50% lower carbohydrate intake on WFKD compared to Med-Plus (<20% vs. <40% total calories from carbohydrates, respectively). Additionally, increased LDL cholesterol, decreased fiber intake, and increased potential for nutrient deficiencies in WFKD may be concerning. Collectively, these comparative results do not support a benefit sufficient to justify avoiding legumes, fruits, and grains to achieve the metabolic state of ketosis. In a clinical setting, patients need to be supported in choosing a dietary pattern that fits their needs and preferences. There should, therefore, be less focus on promoting a particular dietary approach; clinicians should enable patients to make an informed choice to help them determine which approach is best for them. Finally, regardless of the benefits, diets must be sustainable, and this study suggests that it was difficult for participants to follow through on WFKD [27].

Finally, a very recent study that included 9793 adults with prediabetes, taken from the 1999–2014 NHANES survey, investigated the association between different types of low-carbohydrate diets (LCDs) and low-fat diets (LFDs) with mortality. The results demonstrate that subjects with prediabetes who mostly adhered to LCDs and LFDs showed a significantly lower all-cause mortality compared to subjects with less adherence to this type of diet, who, on the contrary, showed higher all-cause mortality [30].

In conclusion, with regard to weight loss in subjects with obese prediabetes, the evidence from scientific research shows that, in this situation, it may be useful to consider low-carbohydrate diets.

### 4.2. Protein Foods

Proteins are essential in a balanced diet and some evidence in the literature focuses on their important role in the prevention and treatment of the onset of prediabetes.

Foods rich in protein appear to be characterized by a low glycemic index (GI) and the intake of proteins induces a release of insulin in any case lower than that generated by the ingestion of carbohydrates [176,177,178].

This suggests that high-protein (HP) diets may help preserve β-cells by increasing insulin sensitivity and decreasing the insulin load per meal [179].

Furthermore, as can be seen from studies on human models by Ferrarini et al. and DeFronzo and Tripathy, the higher protein content in the diet can also contribute to maintaining or increasing lean body mass and, consequently, to favoring the uptake of glucose at the muscle level: the skeletal muscle is, in fact, essential for the clearance of glucose being substantially responsible for the postprandial uptake of about 80% of the glucose taken [9,180].

The current international dietary guidelines regarding the daily protein requirement in the diet range from 0.8 g/kg/day to 1–1.5 g/kg/day depending on the age, sex, and concomitant pathologies of the subjects under examination. However, despite advances in understanding the ideal protein requirement for many states of the disease, there is still a debate about the ideal intake for patients with IR and prediabetes [19]. There are several studies in the literature that highlight the usefulness of protein intake up to 30% of total daily calories [181,182].

#### 4.2.1. Milk and Dairy Products

There are two different types of proteins in milk: caseins and whey proteins. The proportion between the two protein components varies in the milk of various mammalian species. Specifically, in cow’s milk, the most consumed in the world, the whey protein/casein ratio is 1:4 [41]. Caseins and whey proteins are biologically very similar and are both an excellent source of essential amino acids with a super-imposable PDCAAS (protein-digestibility-corrected amino acid score) equal to 1.0 but whey proteins are generally considered as nutritionally superior as they are characterized by a higher biological value; better NPU (net protein utilization), i.e., the proportion of amino acids converted into muscle mass compared to the amount of amino acids supplemented; higher quantity of BCCAAs; and faster absorption [33].

In general, the benefits of milk derivatives are extensively investigated in the literature, also with regard to the relationship with the risk or therapy for prediabetes.

In the recent scoping review by Yau JW et al. concerning different dietary patterns and their efficacy on prediabetes, 19 studies on the impact of protein-rich meals and nuts on prediabetes were considered: with three trials each, protein from milk and soy were the supplementation interventions; substantial improvements in glucose metabolism, especially postprandial glucose, HbA1c, and indicators of oxidative stress were observed in these subjects [42].

One of the first studies that thoroughly investigated this relationship is the 2013 study by Rideout TC et al. Twenty-three healthy subjects completed a randomized, crossover trial of 12 months. Participants consumed their habitual diets and were randomly assigned to one of two treatment groups: a high-dairy-supplement group instructed to consume four servings of dairy per day (HD); or a low-dairy-supplement group limited to no more than two servings of dairy per day (LD).

The results demonstrated that the consumption of four portions of low-fat dairy products (skimmed milk 250 mL or low-fat yogurt 100 g) per day for six months was able to improve the HOMA index (*p* < 0.05) and the blood insulin (*p* < 0.05) [31].

A recent population-based study analyzed the possible relationship between the consumption of dairy products and the incidence of prediabetes in a sample of 2262 participants with no alterations in glucose metabolism at enrolment (mean age 56 ± 7.3 years; 50% male). Having defined a portion of liquid dairy products as a quantity equal to 200 mL and a portion of cheese as a quantity equal to 20 g, after an average follow-up period of 6.4 ± 0.7 years, it emerged that the intake of fermented dairy products, high-fat cheese and full-fat cheeses are associated with a reduction in the risk of prediabetes between the upper quartile (3.9–15.4 servings/day) and lower quartile (0–1.8 servings/day) by 17%, respectively (RR 0.83, 95% CI 0.69–0.99, *p*-trend = 0.04), 14% (RR 0.86, 95% CI 0.73–1.02, *p*-trend = 0.04), and 21% (RR 0.79, 95% CI 0.66–0.94, *p*-trend = 0.01). No association was found with total dairy intake and with other dairy subtypes, suggesting that cheese intake may be related to a lower risk of T2DM, despite the high saturated fat and sodium content [34]. Morever, the same year included an analysis conducted on 6770 subjects (mean age 62 ± 4 years, 59% female), which showed a lower risk of prediabetes in the event of a higher intake of whole yoghurt (HRQ4 vs. Q1 0.70, 95% CI 0.54–0.91 and HRserving/day 0.67, 0.51–0.89) and whole milk (HRQ4 vs. Q1 0.81, 0.67–0.97, HRserving/day 0.88, 0.79–0.99) [35].

In 2017, a study based on the evaluation, using food-frequency questionnaires, of the total intake of dairy and milk-based products on 2809 participants (mean age ± SD: 54.0 ± 9.7 years; body mass index (in kg/m^2^): 27.1 ± 4.7; 54% females), with an average follow-up of 12 years, had already observed, in 1867 subjects without prediabetes at baseline, a lower risk of developing prediabetes in association with consumption (≥14 compared to <4 portions/week) of total, high-, and low-fat dairy products (39%, 32%, and 25% reduction, respectively; servings in grams equal to 245 g for skimmed and whole milk; 15 g for cream and 12 g for sour cream; 96 g for sorbets and iced milk; 66 g for ice cream; 227 g for yogurt; 105 g for cottage cheese and ricotta; 28 g for cream cheese and other cheeses; 5 g for butter). Intake of whole, low-fat, and skimmed milk, as well as whole milk and yogurt, also showed a nonlinear association with the incidence of prediabetes [36].

A longitudinal population study published in 2023, conducted on 4891 healthy Australian subjects, with normal glucose tolerance (age 49.0 ± 12.3 years, 57% females), investigated the intake of dairy products at baseline using the food-frequency questionnaire and the possible occurrence of prediabetes at 5 and 12 years of follow-up. Higher intakes of dairy products and full-fat milk, and whole cheeses were associated with a lower risk of prediabetes [37].

A 2018 study had analyzed in great detail the relationship between the consumption of individual subgroups of dairy products and the risk of developing prediabetes (defined on the basis of fasting blood glucose or Hb1Ac values). Overall, semi-quantitative food-frequency questionnaires and blood samples from 112,086 healthy adult participants were analyzed; an inverse association of skimmed and fermented dairy products and buttermilk with prediabetes had emerged [38].

More generally, however, the cross-sectional population study by Pestoni et al. of 2021, conducted on 1305 participants, showed that a dietary pattern characterized by a high consumption of vegetables, fruit, whole grains, and dairy products (called the Prudent pattern) was associated with a significantly lower probability of developing prediabetes (diagnosed by the oral glucose tolerance test, OGTT) compared to the Western pattern, characterized by the consumption of high quantities of red and processed meat, alcoholic and sugary drinks, and refined cereals [39].

The scientific literature also reports studies concerning the relationship between milk protein supplements, in particular, whey protein, and glycemic control.

Akhavan et al. [32] investigated how premeal supplements of different doses of whey proteins (5, 10, 20, or 40 g) affected insulin release and postprandial glucose: the supplementation of 20–40 g of whey proved able to reduce the subsequent food intake (*p* < 0.0001) and those 10, 20, and 40 g were able to reduce postprandial glycemia and AUC (*p* < 0.05); there is no evidence for supplements of just 5 g of whey protein.

Finally, a recent review in 2019 [40] analyzed the possible advantages of milk proteins in improving glycemic control and, therefore, in delaying the onset of T2DM: the review specifically examined 45 studies concerning the evidence of an association between milk proteins and glycemic control, however, not elaborating a meta-analysis in consideration of the excessive heterogeneity of the studies examined; the doses of whey protein between the various studies examined ranged from supplements of 4.5 g to 90 g/day and that of casein from 12 to 60 g. The study concludes that there is sufficient evidence to state that acute milk protein supplementation improves insulin secretion by positively influencing postprandial glycemic values (but, at the moment, there is no long-term evidence) and that milk proteins can be a valid ally in the treatment of prediabetes and T2DM as they include amino acids and bioactive peptides capable of directly influencing gastric emptying, the secretion of incretin hormones, and the insulin response itself.

In conclusion, scientific literature concurs in demonstrating that a higher intake of milk and cheese, both low-fat and fat-free (three portions of low-fat dairy products (skimmed milk or low-fat yogurt: 300–400 g/day)), is associated with better glycemic control.

Whey protein supplementation (10–40 g of whey protein taken before a meal) may also be helpful in glycemic control, particularly in sarcopenic prediabetic patients.

#### 4.2.2. Eggs

Eggs are a rich source of nutrients, including unsaturated fatty acids (omega-3, EPA, and DHA), choline, essential amino acids, minerals, carotenoids, lecithin, folate, and vitamins (A, D, and B). With about 200 mg of cholesterol per egg, eggs are among the foods with the highest cholesterol content [183]; however, the most recent American guidelines [184] no longer support the association between dietary cholesterol and serum cholesterol (and, consequently, the risk of coronary heart disease). Therefore, these guidelines no longer provided a recommended upper limit for dietary cholesterol intake to avoid the effect on blood cholesterol concentrations.

Some studies are now examining the relationship between egg consumption and the risk of developing prediabetes and T2DM.

In the 12-week randomized controlled trial by Pourafshar S. et al. [43], the correlation between egg consumption and improvement in glycemic control and insulin sensitivity was evaluated. We included 42 overweight or obese subjects aged 40 to 75 years with prediabetes and T2DM. Participants were randomly assigned to receive one large egg per day or an equivalent amount of egg substitute per day for 12 weeks. Blood samples were taken to analyze the lipid profile and biomarkers related to glycemic control.

Regular egg consumption has been shown to lead to an improvement in fasting blood sugar and IR levels (HOMA-IR). The lipid profile was then evaluated: there were no significant changes in the levels of total cholesterol and LDL cholesterol.

A 2017 systematic review [44] evaluated the link between egg consumption and the main cardiovascular risk factors in subjects with T2DM or at risk of the disease (prediabetes, IR, or metabolic syndrome). The inclusion criteria were randomized, controlled trials in which the number of eggs consumed was pitted against an egg-deficient (two eggs per week) or egg-free diet control group.

From 10 articles, it was found that the consumption of 6–12 eggs per week had no negative effects on the plasma concentrations of total cholesterol, LDL, triglycerides, fasting glucose, insulin, and C-reactive protein, in subjects at risk of developing T2DM.

A 2020 systematic review [45] considered three American cohort studies to analyze the correlation between long-term egg consumption and the risk of developing T2DM.

We included 82,750 women from the Nurses’ Health Study (NHS; 1980–2012), 89,636 women from NHS II (1991–2017), and 41,412 men from the Health Professionals Follow-Up Study (HPFS; 1986–2016) with no T2DM, cardiovascular disease, or baseline cancer.

Over a 32-year follow-up period, comprehensive data on egg intake and diet were collected every two to four years. The meta-analysis results indicate that there is no consistent relationship between T2DM risk and moderate egg consumption. However, the various geographic areas have been shown to differ significantly from one another. In fact, in US studies, the risk of T2DM increased by 18% for each egg consumed daily; this did not happen in the European or Asian studies. In the United States, however, egg consumption reflects an adherence to a Western dietary pattern, as eggs are often eaten alongside red or processed meats, refined grains, and sugary drinks.

In conclusion, the intake of 2–4 eggs per week should be recommended in patients with prediabetes.

#### 4.2.3. Meat

Recent studies have investigated the association between the consumption of red and processed meat and the risk of developing prediabetes and T2DM.

In a 2022 cross-sectional study, Nguyen et al. [46] examined the relationship between the consumption of red and processed meat and the risk of prediabetes and T2DM in a cohort of 3000 participants, aged 40 to 60 years old, living in a municipality in Vietnam. Compared to subjects who ate less than 100 g per day, it was found that those who consumed more than 200 g of red meat per day had a 1.67 times higher risk of developing prediabetes and a 1.8 times higher risk of developing prediabetes and T2DM. There was no significant evidence regarding the consumption of white meat.

A further meta-analysis, combined with three cohort studies [48], examined the association between the consumption of red meat, both processed and unprocessed, and the incidence of developing prediabetes and T2DM. Both processed and unprocessed red meat were associated with an increased risk of T2DM in each of three cohort studies, which included 37,083 men from the Health Professionals Follow-Up Study (1986–2006), 79,570 women from the Nurses’ Health Study I (1980–2008), and 87,504 women in Nurses’ Health Study II (1991–2005). A meta-analysis of 442,101 participants supported the findings, which showed a 19% and 51% increase in relative risk for intakes of 100 and 50 g of processed red meat per day, respectively.

Another study including the analysis of three prospective cohort studies [49] investigated the relationship between changes in red meat consumption over a four-year period and the related changes in the risk of developing T2DM and prediabetes. Included in this study were 74,077 females from the Nurses’ Health Study II (1991–2007), 48,709 females from the Nurses’ Health Study (1986–2006), and 26,357 males from the Health Professionals Follow-Up Study (1986–2006). It was found that eating more than 0.50 servings of red meat per day was linked to a 48% greater chance of developing prediabetes and T2DM over the next four years. Meanwhile, reducing red meat consumption by more than 0.50 servings per day from baseline to the first four years of follow-up was associated with a 14% lower risk during the entire subsequent follow-up.

A prospective study conducted by Song et al. [47] established a direct link between the consumption of red and processed meat and the increased risk of T2DM: 37,309 women participated in the Women’s Health Study of age 45 or older, free from cancer, cardiovascular disease, and/or T2DM. Specifically, when comparing women in the top quintile with those in the bottom quintile, the relative risks of T2DM were 1.28 for red meat and 1.23 for processed meat intake.

However, a systematic review with meta-analysis conducted in 2023 on 21 randomized controlled trials [50] evaluated the relationship between the consumption of red meat and the risk of prediabetes and T2DM. The results highlighted that, compared to diets with reduced or no red meat intake, there were no significant differences of red meat intake in terms of insulin sensitivity, IR, blood glucose and fasting insulin, HbA1c, and pancreatic beta cell function. When comparing meals with those with little or no red meat intake, red meat consumption only slightly reduced postprandial glucose.

In conclusion, the intake of red and processed meat must be occasional, a portion of 100 g no more than one day a week.

#### 4.2.4. Plant-Based Foods

Vegetable protein sources, such as legumes, have the great advantage of being low in saturated fats and rich in fiber, phytonutrients, and antioxidants, which, on the contrary, are scarce in foods of animal origin [56]. Antioxidants inhibit glucose uptake, stimulate insulin secretion, reduce hepatic glucose production, and stimulate glucose uptake, as reported in the review by Kim [57]. In fact, several in vitro and in vivo studies on animals have revealed that polyphenols are able to inhibit the action of carbohydrate digestive enzymes (alpha-amylase and alpha-glucosidase) [185,186,187,188] and improve insulin-mediated glucose uptake by activating AMPK in skeletal muscle and upregulating glut-4 expression [189]. The fibers modulate the postprandial glucidic response and stimulate the production of short-chain fatty acids, which, in turn, improve the glucidic response and insulin sensitivity [190]. Iron, zinc, potassium, magnesium, niacin, dietary fiber, and a particularly high intake of environmentally friendly proteins make legumes a one-of-a-kind food with a nutrient-rich profile. Legumes are considered low-glycemic-index foods (GI < 55%) because they are excellent in reducing the postprandial response to glucose and insulin [191]. Soybeans are the legumes with the lowest glycemic index among legumes (GI 16 ± 1%), followed in ascending order by beans (GI 24 ± 4%), chickpeas (GI 28 ± 9%), and lentils (GI 32 ± 5%) [192].

Legume consumption was related to better metabolic health, as reported in the systematic review with meta-analysis by Schwingshackl [58]; this study evaluated the correlation of certain metabolic parameters (systolic blood pressure, glycemic parameters, and lipid profile) and consumption of specific food groups: 66 studies in the literature considered “reliable” were analyzed in detail (out of a total of more than 300, dated between 1979 and 2018); the random effect model was used for the statistical evaluation. Legume consumption was associated with a reduction in LDL cholesterol (−0.30 to −0.12 mmol/L), HOMA-index, total cholesterol, HbA1c, and both systolic and diastolic blood pressure. In the general analysis, legumes were second only to the consumption of dried fruit in terms of benefit for all the outcomes analyzed.

The same working group also investigated the association between the consumption of foods from the main food groups and all-cause mortality [59].

In this review with meta-analysis, 103 prospective studies were considered for the analysis (17 treated legumes as a food group): an inverse association with all-cause mortality was highlighted in the comparison between the category with the highest consumption of legumes in the diet (total range: 6–166 g/day); i.e., those who consume the highest amount are at a lower risk of mortality from all causes than those who consume a smaller amount (RR: 0.96; 95% CI: 0.93, 1.00; *I*^2^ = 48%; *p*-heterogeneity = 0.01); it also highlighted that the risk of mortality from all causes decreased by 16% with the increase in the intake of legumes of up to 150 g/day.

From the observational study by Becerra-Tomás N et al. on a large sample of 3349 non-diabetic patients at baseline (from the PREDIMED epidemiological study), it was possible to observe that, during an average prospective follow-up period of 4.3 years, the individuals who consumed higher amounts of legumes in their ordinary diet (the highest quartile) were exposed to a significantly reduced risk of developing T2DM compared to the lowest quartile (HR: 0.65; 95% CI: 0.43, 0.96; *p*-trend = 0.04). The quartiles were made up as follows: Q1 9.64 ± 3.55 g/day, Q2 16.09 ± 1.41 g/day, Q3 21.57 ± 1.89 g/day, and Q4 34.60 ± 17.24 g/day. In practice, the subjects of Q4 consume about 120–360 g of legumes per week (a range that goes from just under a standard portion of legumes, which turns out to be 150 g, to more than two portions) and are exposed to a significantly higher risk but are less likely to develop T2DM than those who consume less and occasionally [53].

In 2008, H. Shams et al. [54] conducted a case–control study to evaluate the impact of legume supplementation in improving glycemic indices in a sample of 30 patients with T2DM: the patients were randomly divided into two groups, one control group with a standard diet and a treatment group that would consume 50 g of cooked lentils per day (instead of 30 g of bread in the control diet); after a six-week period, total cholesterol and fasting blood glucose were significantly reduced (*p* < 0.05).

From the recent 2020 meta-analysis by Tang J regarding the intake of plant-derived proteins (legumes in general and soybeans and derivatives), it was possible to identify with a moderate degree of evidence that there is a dose-dependent inverse correlation between the consumption of soy (tofu, soy protein, and soy isoflavones) and incidence of T2DM (all *p*-values < 0.05) [60]. Specifically, a daily increase of one serving (124 g) of tofu, 10 g of soy protein, and 10 mg of soy isoflavones was associated with lower risks of T2DM by 32%, respectively (RR: 0.68; 95% CI: 0.50, 0.93), 9% (RR: 0.91; 95% CI: 0.84, 0.99), and 4% (RR: 0.96; 95% CI: 0, 92, 0.99). These data are also confirmed in the previous review (2018) by Li W. et al. [61].

A 2020 Korean study investigated the effect of soy protein supplementation (specifically, black soy peptides, a less common variant of green or yellow soy, typically found in eastern countries and used in Chinese medicine) on glycemic outcomes in patients with impaired glycemia (prediabetics and newly diagnosed T2DM): 55 subjects were recruited and randomly assigned, either to the treatment group or to the control group; subjects in the treatment group were administered 4.5 g/day of black soy peptides for 12 weeks, and the control group a placebo for the same timeframe. It was possible to observe an improvement in the fasting blood glucose of the treated patients compared to the control patients (*p* < 0.05), as well as for the blood glucose 2 h after the meal (*p* < 0.05), while no significant differences were found for parameter HbA1c [51].

Peanuts are also taxonomically classified as legumes. There are few studies in the literature on the effect of peanuts on T2DM and IFG: the prospective study conducted by Rui Jiang et al. in 2002 on a cohort of 83 818 female subjects, aged between 34 and 59 years, without T2DM at baseline, and during an average follow-up of 16 years, showed that peanut butter consumption was inversely associated with the onset of T2DM with an RR of 0.79 (95% CI, 0.68–0.91; *p* for trend <0.001) with an average weekly consumption of five portions (equivalent, according to the authors, to > 140 g/week) compared to those who did not consume it [55].

Instead, from the administration study by C.E.G. Reis et al., it was possible to evaluate the postprandial glucidic response to the intake of peanuts: in a sample of 13 subjects (nine female and four male, healthy, average age 28.5 years and with mean BMI of 22.7), a meal of raw peanuts with skin (RPS) (63 g), roasted peanuts without skin (RPWS) (63 g), and ground-roasted peanuts without skin was randomly administered after 12 h of overnight fasting (GRPWS) (63 g), or an isocaloric, isoglucose, isolipid, and isoprotein control meal; as a consequence, postprandial capillary blood glucose levels were measured at 30, 45, 60, 90, and 120 min and each subject ate all four test meals at different times and in a random sequence (one test every two days, taking one of the randomly chosen from those not previously tested). The glycemic index of peanuts was found to be 14.33. The area under the glycemic response curve (AUC) after GRPWS intake was lower (*p* = 0.02) than that obtained with RPS and overall blood glucose at 15 min and 30 min of roasted peanut meals were hypoglycemic compared to the control meal. There was no treatment effect on energy, macronutrient, and fiber intake after the test meal. The authors argue that the amount of fat released and absorbed in the digestive system depends on the degree of maceration and rupture of the peanut cell wall; therefore, their grinding influences the glycemic and insulinemic responses [52].

In conclusion, from the evidence in the literature, it is possible to deduce that a consumption of vegetable proteins is recommended for the purpose of improving glycemic control and IR: a good amount of legumes, between 150 g fresh, corresponding to 50 g dried (standard portion), three times a week, up to a maximum of 150 mg/day, is recommended. Furthermore, soy and its derivatives, such as tofu, and peanuts, preferably with skins and also in the form of peanut butter, are a valid addition to the diet as they can all contribute to preventing the progression of IR, typical of prediabetes, in overt T2DM.

### 4.3. Sarcopenia

There are still few studies aimed at investigating a possible specific association between sarcopenia and prediabetes. Among the most recent is the study by Xu J. et al. of 2023, conducted on 16,116 adults aged between 20 and 59 years. Sarcopenia, defined by a DXA assessment of appendicular skeletal muscle mass (ASM), was found to be closely associated with an increased risk of prediabetes (particularly of IGT), independent of obesity, triglycerides, and LDL cholesterol levels. In particular, the greatest risk was observed in subjects with sarcopenia and, at the same time, obesity, especially if centrally obese [193]. In another cross-sectional study of 1629 elderly (mean age 73.1 ± 5.4 years) living in Japan, logistic regression demonstrated that prediabetes represents an independent risk factor for sarcopenia (assessed by appendicular skeletal muscle mass) for male subjects, but not female subjects [63].

Considering how a reduced metabolism and muscle mass are associated with an increase in IR and the appearance of T2DM, the causal relationship between sarcopenia and impaired glucose metabolism could, in fact, be bidirectional; in this regard, a study conducted on 22,482 adults aged ≥ 20 years, defining sarcopenia as the ratio of appendicular skeletal muscle mass/body mass index (ASMBMI) < 0.789 for men and <0.512 for women (based on the established by the Foundation for the National Institutes of Health (FNIH) Sarcopenia Project), showed that prediabetes is associated with an increased risk of sarcopenia [OR (95% CI) = 1.230 (1.057, 1.431), *p* = 0.008]. In particular, an HbA1c > 5.2% was found to be an independent risk factor for the loss of appendicular skeletal muscle mass and sarcopenia in male participants without T2DM [64].

Already, in 2021, a meta-analysis performed on 16 observational studies to investigate the association between the presence of sarcopenia and HbA1c values, prediabetes, T2DM, and its complications, had shown that subjects with prediabetes had reduced mass, strength, and muscle performance compared to non-diabetics [65].

Therefore, further studies are needed in order to confirm not only the existence of an association between sarcopenia and prediabetes, but also to better define, possibly, the causal relationship between the two conditions. In any case, the assessment and monitoring of body composition over time must be part of the multidimensional assessment of the patient with prediabetes.

In conclusion, given that the studies that have been carried out up to now investigating the correlation between prediabetes and sarcopenia concur in demonstrating that subjects with prediabetes had reduced mass, strength, and muscle performance compared to non-diabetics, it is important that patients with prediabetes be screened in order to evaluate the possible presence of this pathology. Finally, given this background, it is equally important that protein intake is taken adequately by patients with prediabetes.

### 4.4. Lipid-Containing Foods

An impaired fatty acid (FA) metabolism plays a pivotal role in the development of an impaired glucose metabolism and, consequently, in the onset of T2DM [194]. An interesting review by Stinkens provides an overview of the pathways related to the metabolism of fatty acids in the adipose tissue, liver, and muscle. Adipose tissue dysfunction can result in excess lipids, systemic inflammation, and the excessive accumulation of lipids in non-adipose tissue such as the liver, skeletal muscle, and pancreas. All of these alterations favor the development of an impaired glucose metabolism, IR, and T2DM. The review suggests that improving the lipid storage capacity of adipose tissue prevents excess lipids in circulation and the consequent ectopic fat deposition, and, thus, has a high potential to improve glucose tolerance and insulin sensitivity.

The improvement of the mitochondrial function of adipose tissue can be achieved by nutritional strategies such as supplementation with specific polyphenols (or a combination of polyphenols) and modification of the FA composition in the diet. The quality of dietary fat not only modulates lipid metabolism but may also affect low-grade inflammation which, in turn, may reduce the risk of developing IR and T2D. Targeting skeletal muscle lipid turnover and the balance between lipolysis, FA uptake, and mitochondrial function/fat oxidation may be an attractive strategy with which to improve insulin sensitivity in obesity and T2D.

The most promising dietary intervention to reduce FA uptake is the improvement of the lipid-buffering capacity of adipose tissue. The increased activation of SIRT-1 and PGC-1 may result in an improved skeletal muscle mitochondrial biogenesis function, fat oxidation, and metabolic flexibility. The modulation of dietary polyphenol content or modulation of dietary fat quality may also have positive effects on skeletal muscle lipid metabolism and insulin sensitivity. Finally, the prevention of β-cell apoptosis and modulation of β-cell proliferation by bioactive compounds (including polyphenols, vitamins, and carotenoids) may maintain insulin secretory capacity and, as such, could be a potential target to reduce the incidence of T2D.

In conclusion, the manipulation of the quantity and quality of dietary fat is of particular interest for improving adipose tissue function, skeletal muscle lipid turnover, and mitochondrial function. Further human intervention studies targeting specific AF-related pathways are needed to translate the indications above into relevance for the control of glucose homeostasis and insulin sensitivity.

A case–control study aimed at determining the association between dietary fat quality (DFQ) indices and fatty acid intake with prediabetes was carried out.

In the study, 150 subjects with normal fasting glucose (FBG) and 147 prediabetic subjects were included. (The inclusion criteria for prediabetic subjects were as follows: age between 35 and 65 years, fasting glucose of 100–125 mg/dL after an 8–12 h fast, or OGTT of 140–199 mg/dL after 2 h and with a 75 g glucose load, diagnosed no more than three months before the interview.) Participants were interviewed to complete a valid semi-quantitative FFQ with 168 food items. The Healthy Eating Index (HEI) score was calculated, which summarizes the consumption of 13 foods or nutrients (including consumption of total fruits, whole fruits, total vegetables, greens and beans, whole grains, dairy products, total protein foods, seafood and plant proteins, fatty acids, refined grains, sodium, added sugars, and saturated fats). Each component was rated on a scale of 0 to 10. In the Dietary Fat Quality Survey, a positive association was found between total saturated fatty acid (SFA), myristic and palmitic acid intakes, and prediabetes. Furthermore, an inverse association was observed between the intake of *n*-3 polyunsaturated fatty acids, eicosapentaenoic acid, docosahexaenoic acid, arachidonic acid, and prediabetes (*p*-trend < 0.05) [73].

Another 2018 study wanted to demonstrate the association between the quality and quantity of lipids in the diet and the risk of onset of T2DM in adults. For the study, 2139 adult subjects between 20 and 70 years of age, without T2DM, were taken into consideration, and followed for more than five years with the assessment of dietary intake using validated questionnaires. During the follow-up, in which 143 new cases of T2DM were found, high intakes of monounsaturated, polyunsaturated, omega-3 fatty acids, and cholesterol were associated with a lower risk of T2DM. Additionally, omega-6/omega-3 and total fat/omega-3 ratios were also found to be positively associated with T2DM [74].

Fatty acids (FAs) influence the translocation of glucose transporters, as well as insulin receptor binding and signalling, as well as contribute to cell membrane fluidity and permeability. A 2021 review details the role of FAs in the pathogenesis of T2DM, which, to date, has not been fully shown.

Through lipidomic techniques (a subcategory of metabolomics), it has been shown that specific combinations of FAs within phospholipids and triglycerides have a stronger association with the risk of T2DM, particularly shorter-chain saturated fatty acids (SFAs).

Elevated TG levels increase FFA, resulting in the accumulation of DAG (diacylglycerol) and fatty acyl Co-A and increased ROS. All this together activates PKC (protein kinase C), which disrupts the insulin signalling pathway [195].

As explained in the review, the increase in palmitic acid is due to an enhancement of the de novo synthesis of fatty acids, as well as the alteration of cellular organelles and the promotion of a pro-inflammatory state. Furthermore, the increase in intracellular levels of palmitic acid depends on the plasma content of non-esterified fatty acids (NEFAs), which promote lipotoxicity and lipoapoptosis.

When palmitic acid levels rise beyond mitochondrial oxidation limits, this causes excess fatty acids to be converted into harmful lipids such as diacylglycerol and ceramide which, if present in high amounts, can activate protein kinase C (PKC) isoforms, which, in turn, phosphorylate the Serine 1 residues of insulin, thus attenuating its signalling pathway and, consequently, determining IR. Furthermore, PKC isoforms inhibit the pro-inflammatory signalling cascade and promote ceramide synthesis, the latter being involved in the inhibition of FA mitochondrial β-oxidation.

Collectively, these changes cause metabolic dysregulation and promote the onset of T2DM.

On the other hand, oleic acid produces beneficial effects on insulin sensitivity, such as the production of anti-inflammatory effects (due to the activation of IL-10), the increase in the production of adiponectin, and the reduction of pro-inflammatory cytokines (IL-6 and TNF-α).

As mentioned earlier, omega-3s may improve insulin signalling and a possible mechanism is explained in the review. In fact, PUFAs can reduce endoplasmic reticulum stress and improve beta-oxidation at the mitochondrial level, thus determining a reduction in the accumulation of lipids, as well as reactive oxygen species (ROS).

EPA and DHA regulate insulin sensitivity through the phosphorylation of Akt (protein kinase B), activation of AMPK (AMP-activated protein kinase), and activation of PPAR-γ (peroxisome proliferator-activated receptor-γ). They inhibit the expression of pro-inflammatory mediators from adipose tissue and promote the expression of adiponectin, thereby indirectly promoting insulin secretion from pancreatic β-cells, while having a direct effect on the same function by exerting a role on the membrane fluidity and structure of the lipid raft and binding to PPARs, GPR40, and GPR120, thereby promoting insulin secretion.

Conversely, omega-6s exert a pro-inflammatory effect, since the product of desaturation, arachidonic acid, produces inflammatory cytokines and eicosanoids.

Thus, maintaining an optimal balance of omega-6 and omega-3 FAs in the diet is critical to human health. The review proposes a ratio between 1:1 and 2:1 as a target ratio between omega-6 and omega-3. Therefore, FA dietary interventions specifically aimed at maintaining an optimal omega-6/omega-3 ratio may overcome the risk of developing T2DM [196].

#### 4.4.1. Fish

Numerous epidemiological studies have investigated the association between the intake of fish, the food richest in omega-3, and the risk of prediabetes.

A meta-analysis, creating a database of 12 independent cohorts followed in longitudinal studies for about 11.4 years, found an inverse relationship between the incidence of T2DM and fish consumption by combining studies on Eastern populations, while such a relationship did not exist among Western populations [78].

A Japanese longitudinal study evaluated the incidence of new cases of T2DM in a population of 22,921 men and 29,759 women. This incidence was found to be 971 new cases. In men, fish intake was found to be statistically significant and associated with a decreased risk of T2DM. However, this association was not found in the female population [75].

A case–control study carried out in 2021 evaluated how, in a pool of 152 people over the age of 65 and with fasting blood sugar between 100 and 124 mg/dL, those who followed a balanced diet enriched with two weekly portions of sardines showed, in one year, a greater decrease in blood pressure, an increase in the omega-3 index, an increase in circulating metabolites considered protective against T2DM, an increase in adiponectin, and an improvement in IR assessed by HOMA-IR [66].

Another study conducted by Rajabi-Naeeni et al., conducted on 168 women aged between 15–50 years with prediabetes, found a significant reduction in fasting insulin in those who took a supplement of 1 g per day of omega-3 or a combined supplementation with omega-3 and vitamin D [67].

Another 2017 study conducted in Indonesia administered a supplement of 3 g per day of omega-3 for 12 weeks to a pool of 45 women suffering from obesity: the supplementation led to a greater control of prediabetes compared to the group that took the placebo [68].

A 2018 study hypothesized that omega-3 fatty acids may have a preventive role in patients with prediabetes, also in light of the benefits that such supplementation can have against anxiety and sleep disorders, conditions that pose a greater risk for the development of metabolic syndrome [69].

A Danish study found, by carrying out a long follow-up on a middle-aged cohort, how a diet that favors white meat and fish in place of processed meats determines a lower risk of developing T2DM [76].

In 2021, a 10-year longitudinal study evaluated the incidence of new cases of T2DM and the consequent correlation with the diet of the participants (evaluated by 24 h recall). In a cohort of 392,287 people, 7262 new cases of T2DM were found. It emerged that the consumption of lean fish does not seem to correlate with the risk of developing T2DM; instead, a statistically significant inverse correlation (*p* < 0.001) was found between the consumption of fatty fish and fish oil and the risk of T2DM. Those who reported eating two or more servings of fatty fish per week or fish oil had a 22% lower risk of developing T2DM, even after adjusting for any confounders. Those who already consumed fish oil regularly at baseline had a 9% lower risk [77].

#### 4.4.2. Olive Oil

Extra-virgin olive oil, the food richest in polyunsaturated fatty acids, is probably one of the major components that distinguish the Mediterranean diet. It is, in fact, the most abundant oil in flavonoids, lignans, and bioactive compounds, in light of its low level of refining. The main polyphenols contained in olive oil are oleuropein and hydroxytyrosol, as reported in the Narrative Review of the Evidence by Guasch-Ferrè [197].

A 2017 meta-analysis highlights how olive oil intake can be beneficial for the prevention and treatment of T2DM. Four study cohorts were analyzed, a total of 15,784 subjects with T2DM. The highest intake of olive oil (approximately 15–20 g per day) showed a 16% reduction in the risk of onset of T2DM compared with a lower intake. In patients with T2DM, on the other hand, supplementation with olive oil showed a more marked reduction in HbA1c and fasting blood glucose than in the control group [79].

The literature has hypothesized that the effectiveness of taking EVO OIL on lipid metabolism is not only due to the lipid content but also to an action on intestinal permeability.

An Italian study from 2022 evaluated the role of EVO oil in 20 subjects with IFG, hypothesizing a role for the latter in effectively reducing intestinal permeability and the consequent metabolic endotoxemia. The addition of EVO oil to the meal showed, in subjects affected by IFG compared to healthy subjects, a decrease in LPS and zonulin values, suggesting a potential role in reducing intestinal permeability. At the same time, a reduction in postprandial glycemia at 2 h and an increase in insulin and GLP1 values were detected [72].

A double-blind randomized study found that, in a group of patients at risk for T2DM (as a result of suffering from prediabetes or metabolic syndrome), the intake of 50 mL of EVO oil significantly improved the endothelial function [70].

An Italian cross-over study found improvements in the blood sugar, insulin, and GLP-1 levels of 30 patients following the administration of 10 g of extra-virgin olive oil. An improvement in the activity of dipeptidyl peptidase 4 (DDP4) was also found in these patients [71].

In conclusion, there are numerous studies that have investigated the role of fatty acids in prediabetes, in particular, the beneficial role of the intake of foods rich in mono- and polyunsaturated fats. The protective role of an intake of at least 2–3 portions of fish weekly in T2DM is linked to the presence of omega-3 fatty acids EPA and DHA and their anti-inflammatory action, capable of increasing membrane fluidity, and the number and efficiency of insulin receptors. Furthermore, the benefits of fish could also be linked to the content in the latter of proteins and amino acids, capable of increasing the sense of satiety and, consequently, facilitating weight loss.

As regards monounsaturated fatty acids, the literature has shown that the daily intake of at least 10 mL per day (optimal 15–20 mL) of extra-virgin olive oil represents an important strategy in glucid control in prediabetes. The effectiveness is due, in particular, to the presence of high quantities of polyphenols in the EVO oil.

### 4.5. Vitamins

#### 4.5.1. Vitamin D

Recent meta-analyses have demonstrated the beneficial effects of vitamin D supplementation in delaying progression to T2DM (relative risk reduction of 11–12% compared to the placebo) and also in improving the regression to the normal regulation of glucose (relative benefit of 48% compared to the placebo) [85]. In the pathogenesis of prediabetes and T2DM, the role of deficient or altered levels of insulin, adiponectin, and 25 hydroxy vitamin D (25[OH]D) regulate food intake, energy metabolism, glucose and lipid metabolism, and body weight. Adiponectin is a hormone synthesized by adipocytes, which plays an important role in the regulation of glucose metabolism and which increases insulin sensitivity, exerting antidiabetic and anti-inflammatory activities.

Similarly, vitamin D appears to improve insulin sensitivity through various mechanisms, and recent studies have found a strong link between vitamin D deficiency, obesity, and metabolic syndrome. Since both low levels of vitamin D and adiponectin are associated with increased obesity, the association between vitamin D and adiponectin could provide an explanation for the development and natural course of T2DM.

As their relationship and etiology are still unknown, Banerjee et al. conducted a cross- sectional study published in 2017 with the aim of investigating the role of these parameters and establishing their interrelationship in patients with prediabetes and T2DM. The study included 202 subjects: 77 subjects with T2DM (mean age 48.09 ± 6.8), 73 with prediabetes (mean age 49.96 ± 7.6), and 52 healthy subjects constituting the control group (mean age 50.08 ± 7.1). All subjects were matched for age, gender, and BMI within study groups. In all study groups, fasting serum levels of adiponectin, insulin, and 25(OH)D were measured, and routine biochemical parameters were analyzed. The results show statistically lower serum adiponectin and 25(OH)D levels and higher serum insulin levels in subjects with prediabetes or T2DM compared to controls. Changes in serum adiponectin or serum 25(OH)D in people with prediabetes and T2DM were inversely related to serum insulin levels. Furthermore, a multiple linear regression analysis with 25(OH)D, insulin, and HOMA-IR as variables revealed that serum adiponectin levels could be an independent risk factor for the progression of prediabetes and T2DM. In conclusion, the authors state that the association of these hormones could act as a predictor of the progression of prediabetes to T2DM [81].

To evaluate whether vitamin D supplementation reduces the risk of developing T2DM in patients with prediabetes, Zhang Y. et al. conducted a systematic review and meta-analysis published in 2020, including randomized control trials evaluating vitamin D supplementation versus a placebo. The primary outcome was new-onset T2DM; the second was the reversibility of prediabetes.

The authors identified eight eligible studies with a total of 4896 subjects. The population consisted of adults with prediabetes (defined by the WHO and American Diabetes Association criteria) who were taking vitamin D, regardless of type, dose, duration, or route of administration. Specifically, five studies recruited participants with prediabetes, two studies recruited participants with prediabetes and vitamin D deficiency, and one study recruited participants with prediabetes, vitamin D deficiency, and obesity. The follow-up duration of the studies ranged from six months to five years.

All eight studies reported the development of new-onset T2DM, particularly in 1022 (20.9%) of the 4896 participants. Combining data from all eight studies that reported an RR, vitamin D supplementation reduced the incidence of new-onset T2DM by 11%. In particular, non-obese subjects benefitted more than obese subjects. The reversion of prediabetes to normoglycemia occurred in 116 of 548 (21.2%) participants in the vitamin D group and 75 of 532 (14.1%) participants in the control group.

In conclusion, in people with prediabetes, vitamin D supplementation reduces the risk of T2DM and increases the rate of reversion of prediabetes to normoglycemia [85].

Considering that vitamin D has a regulation on glucose homeostasis pathways, Rasouli et al. conducted a study published in 2022 with the aim of investigating the effects of vitamin D supplementation on β-cell function. This was a randomized, double-blind, placebo-controlled clinical trial conducted at 22 sites in the United States comparing vitamin D versus a placebo for T2DM prevention in adults at high risk for T2DM. To be eligible, participants had to meet at least two of three glycemic criteria for prediabetes defined by the 2010 American Diabetes Association guidelines: fasting plasma glucose (FPG) 100 to 125 mg/dL (5.6 to 6.9 mmol/L); plasma glucose 2 h after a 75 g oral glucose load 140 to 199 mg/dL (7.8 to 11.0 mmol/L); and HbA1c 5.7% to 6.4% (39–47 mmol/mol). The other inclusion criteria were age (>30 years) and BMI (between 24 and 42 kg/m^2^). In total, there were 1774 participants, with a mean age of 60.5 ± 9.8 years and a mean BMI of 31.9 ± 4.4 kg/m^2^, 44% were female, and 69% white.

Participants were randomized to either a single soft gel containing 4000 IU vitamin D3 (cholecalciferol) or a matching placebo once daily. Randomization was divided into blocks by site, body mass index (<30 or ≥30 kg/m^2^), and race (white or non-white). Participants were asked to refrain from the use of specific T2DM or weight-loss medications during the study and to limit the use of other vitamin-D-containing supplements or multivitamins. During the study, participants received information on T2DM prevention through information sheets and bi-annual group meetings.

The main outcomes included changes in insulin sensitivity, insulin secretion, and β-cell function in response to the study intervention (vitamin D vs. placebo) over the first two years of the study. The homeostasis assessment model (HOMA) was used to estimate steady-state insulin sensitivity (%S) and β-cell function (%B) as percentages of a normal reference population. The study involved measuring C-peptide and insulin concentrations at baseline, 12 months, and 24 months.

Approximately one-third of participants met all three prediabetic criteria (FPG, 2 h plasma glucose after a 75 g oral glucose load, and HbA1c). The mean baseline vitamin D level was 27.9 ng/mL and increased to 52.8 at 12 months and 54.9 mg/dL at 24 months in the vitamin D group, whereas it was unchanged in the placebo group (28.5 ng/mL at baseline, 27.9 ng/mL at 12 months, and 28.4 ng/mL at 24 months). Of the total of 1774 participants, 275 (15.5%) subjects had a diagnosis of T2DM between the 12th and 24th months of follow-up, 116 (13.1%) in the vitamin D group vs. 159 (18.0%) in the placebo group, showing a significantly lower incidence of T2DM onset in the vitamin D group.

The authors state that this study showed no difference or change in β-cell function with vitamin D supplementation versus placebo. However, vitamin D improved β-cell function among those who had baseline 25(OH)D levels below 12 ng/mL. The rate of incident T2DM over the two years of the study was significantly lower in the vitamin D group than in the placebo group, showing a real benefit from vitamin D supplementation in patients with prediabetes [80].

In conclusion, the evaluation of vitamin D blood levels is mandatory in patients with prediabetes in order to identify personalized supplementation of this vitamin.

#### 4.5.2. Group B Vitamins

As for the B vitamins, by regulating homocysteine synthesis, these vitamins could reduce the risk of T2DM, by reducing IR and oxidative stress.

Hyperhomocysteinemia has emerged as a risk factor for prediabetes and T2DM due to its association with IR (IR). Hyperhomocysteinemia can be caused by various factors, including dietary factors, and a low intake of folate, vitamin B6, and B12.

These B vitamins play a fundamental role in the degradation of homocysteine (Hcy) by acting as preliminary substrate donors (folate) or as essential coenzymes (vitamin B6 and B12). Furthermore, these vitamins are vital components of a one-carbon metabolism that contributes to DNA methylation, critical to the pathogenesis of T2DM [82].

To prospectively examine folate, vitamin B6, and B12 intakes in relation to T2DM incidence in a large US cohort, Jie Zhu et al. published the results of a 30-year follow-up of young adult patients in 2020.

A total of 4704 American adults (52% female and 51% black) between 18 and 30 years of age (mean age, 24.9 ± 3.6 years) and without T2DM were enrolled in this study in 1985–1986 and monitored through 2015–2016 in the Coronary Artery Risk Development in Young Adults (CARDIA) study. Dietary assessment was conducted by means of a validated anamnestic questionnaire three times: at baseline, in 1992–1993 (year 7), and in 2005–2006 (year 20). The cumulative mean intakes (from diet and supplementation) of folate, vitamin B6, and B12 were included in the analyses. Cases of incident T2DM were defined on the basis of plasma glucose levels (measured at baseline and then at the 7th, 10th, 15th, 20th, 25th, and 30th year), oral glucose tolerance tests (with 10th, 20th, 25th, and 30th year of OGTT), hemoglobin A1c concentrations (measured at 20th, 25th, and 30th year), and/or antidiabetic drugs.

During 30 years (mean of 20.5 ± 8.9) of follow-up, 655 cases of T2DM occurred. Folate intake, but not vitamin B6 or vitamin B12 intake, was inversely associated with the incidence of T2DM after adjustment for potential confounders, especially in older subjects and whites. To investigate the possible mechanisms behind the potential benefits of folates in diabetics, we studied the correlation between folate intake and plasma homocysteine blood glucose, insulin levels, HOMA-IR and HOMA-β function index, and inflammatory indices. It emerged that folate intake was inversely associated with homocysteine levels, so a higher intake of these was also associated with a reduction in plasma homocysteine (*p*-trend < 0.01) and insulin (*p*-trend < 0.01). Finally, among supplement users, folate intake was inversely associated with serum C-reactive protein levels (*p*-trend < 0.01).

In conclusion, this prospective study found that folate intake (both dietary and supplemental) in young adulthood is inversely associated with the incidence of T2DM (over a 30-year follow-up) among Americans. The observed association may be partially explained by mechanisms binding homocysteine, insulin sensitivity, and systemic inflammation [82].

These findings are supported by a previous 2012 case–control study which demonstrated that both dietary intake and serum folic acid level were lower in Omani patients with T2DM than in healthy control subjects [198] and from two prospective cohort studies (2017 and 2019) that found dietary folate consumption was inversely associated with incident T2DM in Korean or Japanese women [199,200]. However, the cases of T2DM in these two cohort studies were self-reported and were not validated by clinical measurements and data.

Lind MV and colleagues, in 2019, conducted a meta-analysis of the randomized controlled trials present in the literature, to investigate the effects of folate supplementation on the outcome of IR and T2DM. In this meta-analysis, 29 studies were analyzed (with a total of 22,250 participants) and they evaluated the effect of placebo-controlled folate supplementation, alone or in combination with other B vitamins, on fasting glucose, insulin, model evaluation of homeostasis for IR (HOMA-IR), HbA1c, or risk of T2DM. Of the 29 studies included (4 with a crossover design and 25 with a parallel design), the majority (*n* = 26) reported fasting blood glucose values. For fasting insulin and HOMA-IR, 11 studies were included, while, for HbA1c, 8 studies were included. Only two studies were included for the development of T2DM as an outcome, with a follow-up of 4 and 7.3 years, respectively. The duration of studies reporting glucose metabolism outcomes ranged from two weeks to two years, with the majority of studies ranging in duration from four to eight weeks. Of the 29 studies, 21 studies involved supplementation with folic acid alone, while 3 included vitamin B12, 1 included vitamin B6, and 4 included both vitamin B12 and vitamin B6. The folic acid dosages used ranged between 0.4 and 15 mg/day (the most used dosage was between 0.8 and 5 mg/day).

The results of all these studies showed a mean reduction in homocysteine ranging from 0.3 to 4.3 µmol/L compared to treatment with a placebo. As far as the study population is concerned, possible pathologies were also taken into account: one study analyzed the results on women with polycystic ovary syndrome; eight studies were on subjects with T2D; six on subjects with metabolic syndrome traits—overweight, obese, hypertensive, or with multiple metabolic syndrome variables; seven on people with heart disease; and five included apparently healthy participants. Most of the studies included both genders, but six involved only women and five only involved men. The T2DM developmental outcome studies had a total of 24,954 participants, with 916 new cases of T2DM identified during follow-up.

There were no significant effects of folate administration on fasting blood glucose. Only studies combining folate and B vitamins showed lower fasting blood sugar compared with placebos, while folate administration alone showed no difference between the treated and placebo groups. Instead, compared with the placebo, folate supplementation reduced fasting insulin (WMD: −13.47 pmol/L; 95% CI: −21.41, −5.53 pmol/L; *p* < 0.001) and HOMA-IR (WMD: −0.57 units; 95% CI: −0.76, −0.37 units; *p* < 0.0001), but no particular effects were observed for fasting glucose or the HbA1c. Changes in homocysteine following folate supplementation correlated with changes in fasting glucose (β = 0.07; 95% CI: 0.01, 0.14; *p* = 0.025) and HbA1c (β = 0.46; 95% CI: 0.06, 0.85; *p* = 0.02). Only two studies reported the development of new cases of T2DM in the follow-up period. Of these, one used folic acid alone and the other used folic acid, vitamin B12, and vitamin B6. There were no differences for folate supplementation on overall risk of T2DM (0.91; 95% CI: 0.80, 1.04; *p* = 0.16) and no heterogeneity was found in the results of the two studies (*I^2^* = 0%; *p* = 1.00). Therefore, the authors conclude the work stating that folate supplementation could be beneficial for glucose homeostasis and RI reduction, but, at present, there is insufficient data to definitively determine the effect on T2DM development [86].

The most recent study on this subject is one published by Jin G et al., who conducted and published in 2021 a cross-sectional study to define the association between folate, B12, and B6 obtained from diet, supplementation, and T2DM and prediabetes in American adults. Subjects from the general American population were chosen, reaching a total of 22,041 participants (10,672 men and 11,369 women), to select 29,201 candidates over the age of 20. To define their condition of T2DM or prediabetes, five diagnostic criteria were applied: three based on laboratory data and two on questionnaires. To calculate the intake of the vitamins concerned, 24 h recalls on two different days were used. The mean value of intake was obtained from the 24 h diary and from the supplementation. From the results, of the 22,041 participants, 18.3% had T2DM and most were over 60 years old. Healthy subjects ate more folate, vitamin B12, B6, and calories than diabetics. In binary logistic regression analysis, dietary folate and B6 were associated with a lower risk of T2DM, and, after adjusting for confounders, folate, B6, and B12 levels were inversely associated with T2DM. Dietary folate and B6 intakes were negatively associated with new T2DM diagnoses, and B12 and B6 were inversely associated with prediabetes. In conclusion, therefore, the authors state that there is an association between low values of B vitamins and the risk of T2DM, especially in the over-60 population [83].

Since it is possible with routine tests to carry out the evaluation of folate, vitamin B 12, and homocysteine in the blood, it is essential to carry out these analyses in subjects with prediabetes in order to supplement in case of folic acid and vitamin B12 deficiencies.

#### 4.5.3. Vitamin C

Research suggests that chronic low-grade inflammation and oxidative stress play a key role in the development of IR and T2DM, as well as related complications. Vitamin C is a micronutrient with potent antioxidant properties, essential to humans for many biological functions and as an enzyme cofactor, and recent studies suggest that people with T2DM have lower plasma vitamin C values than subjects with normal glycemic control.

With the aim of analyzing plasma vitamin C concentrations across the glycemic spectrum and investigating the correlation with metabolic health indices, Wilson et al. conducted a pilot cross-sectional observational study of adults with various glycemic conditions ranging from normal glucose tolerance (NGT) to those with T2DM.

Demographic and anthropometric data, and physical activity information were collected, and participants were asked to complete a four-day food diary. Venous blood samples were collected and glycemic indices, plasma vitamin C concentrations, hormone tests, lipid profiles, and high-sensitivity C-reactive protein (hs-CRP) were analyzed.

A total of 89 participants completed the study, including individuals with NGT (*n* = 35), prediabetes (*n* = 25), and T2DM managed with diet alone or a metformin-only regimen (*n* = 29). Vitamin C plasma concentrations were significantly lower in subjects with T2DM than in those with NGT (41.2 µmol/L versus 57.4 µmol/L, *p* <0.05), and a higher rate of vitamin C deficiency was observed (i.e., <11.0 µmol/L) in both the prediabetic and T2DM groups. Results showed that fasting glucose (*p* = 0.001), BMI (*p* = 0.001), smoking history (*p* = 0.003), and dietary vitamin C intake (*p* = 0.032) are significant independent predictors of vitamin C plasma concentrations.

In conclusion, these results suggest that adults with a history of smoking, prediabetes or T2DM, and/or obesity have higher vitamin C requirements [84].

Because existing randomized control trials had observed conflicting results of the effects of vitamin C on circulating biomarkers of glycemic and insulin regulation, Ashor AW and colleagues published in 2017 a systematic review and meta-analysis of RCTs testing the effect of vitamin C administration on glucose, HbA1c, and insulin concentrations and on insulin sensitivity. For this meta-analysis, four databases (PubMed, Embase, Scopus, and Cochrane Library) were used to retrieve the studies published up to April 2016 and which verified the effects of supplementation with vitamin C alone (dosage between 72 and 6000 mg per day, with an average of 1000 mg/day) in adults over the age of 18, regardless of their state of health, and/or phenotype. A total of 22 studies were included (16 with parallel design and 6 with crossover design) for a total of 937 participants, with an average age of 49 (range 22–60). Having identified two independent subgroups in two studies, the analysis was ultimately based on a total of 24 studies: 23 described the effects of vitamin C on blood glucose, 9 on insulin, and 10 on HbA1c concentration; 19 studies analyzed fasting glucose, and 4 studies analyzed postprandial glucose. Finally, 13 studies involved subjects with T2DM, 8 studies healthy subjects, 2 studies subjects with 1DM, and 1 study patients with coronary disease. The duration of the various studies ranged from 1 to 120 days (mean of 30 days).

The results showed that, overall, vitamin C did not change glucose, insulin, and HbA1c concentrations. However, a subgroup analysis showed that vitamin C significantly decreased glucose concentrations (−0.44 mmol/L, 95% CI: −0.81, −0.07, *p* = 0.01) in patients with T2DM, as well as in interventions longer than 30 days (−0.53%, 95% CI: −0.79, −0.10, *p* = 0.02). The analysis showed a better benefit of vitamin C administration on glycemia in those with a higher baseline BMI and glucose concentration, with the greatest effects in the longer duration studies. As regards insulin concentration, positive effects were observed on fasting values, but not on postprandial ones. The best response was observed in the elderly and in overweight and obese subjects.

In summary, the greatest reduction in glucose concentrations was observed in patients with T2DM, older individuals, and with more prolonged supplementation [87].

In conclusion, personalized vitamin C supplementation could represent a possible future strategy to increase the benefits and effectiveness of interventions. However, the results should be interpreted with caution due to the limitations of the primary studies analyzed.

#### 4.5.4. Vitamin E

The association between oxidative stress and T2DM has been recognized for some time and is based on the observation that hyperglycemia, hyperinsulinemia, and IR can increase the generation of free radicals and, thus, contribute to oxidative stress. Oxidative stress may, in turn, promote the glycation of hemoglobin and impair insulin signalling and secretion by β-cells. It is, therefore, reasonable to hypothesize that antioxidants, such as vitamin E, may have beneficial effects on glycemic control in T2DM.

Observational studies have revealed that higher serum vitamin E concentration, as well as increased intake and supplementation of this vitamin, is associated with beneficial effects on glycemic control in T2DM. However, it is still unclear whether the Vitamin E supplementation has a definite effect on glycemic control. The article published in 2014 by Xu R and colleagues provides a meta-analysis of randomized control trials (conducted in parallel or with a crossover design) on vitamin E with the aim of characterizing its impact on HbA1c values, fasting blood glucose, and fasting insulin. All relevant studies in existence up to April 2013 were reviewed within PubMed, EMBASE, and the Cochrane Library for the analysis. Inclusion criteria required that subjects be adults with T2DM and supplement vitamin E for at least 6 weeks and that this treatment was the only difference with the control groups. A total of 14 randomized control trials were thus selected, involving a total of 714 subjects (including 363 subjects for the vitamin E group and 351 for the control group); 12 studies evaluated the effects on HbA1c, 12 on fasting glucose, 6 on fasting insulin. The range of vitamin E administered was 200 to 1600 IU/day, taken from 6 to 27 weeks.

The meta-analysis results showed that increasing vitamin E supplementation did not lead to significant benefits in glycemic control, as measured by reductions in HbA1c, fasting glucose, and fasting insulin. A subgroup analysis revealed significant reductions in HbA1c (0.58%, 95% CI −0.83 to −0.34) and fasting insulin (−9.0 pmol/L, 95% CI −15.90 to −2.10) versus controls in patients with low baseline vitamin E status. The subgroup analysis also demonstrated that results may have been influenced by vitamin E dosage, study duration, ethnic group, serum HbA1c concentration, and fasting glucose control status [88].

In conclusion, based on the evidence analyzed from the randomized control trials, there is insufficient evidence supporting a potential beneficial effect of vitamin E supplementation on improving HbA1c, fasting glucose, and insulin concentrations in subjects with prediabetes.

### 4.6. Minerals

Scientific literature has evaluated the intake of some minerals, in particular, zinc, magnesium, selenium, and calcium, on glucose metabolism.

Since zinc is an element capable of influencing glucose homeostasis in animals and humans, a systematic review and meta-analysis of 14 randomized control trials (for a total of 3978 subjects, of which 3298 were healthy, 408 affected by T2DM, 48 with DM1, 48 with DM1 or T2DM, 120 with metabolic syndrome, and 56 obese subjects) evaluated the effect of zinc supplementation on fasting blood glucose, HbA1c, insulin, and blood zinc concentrations. Overall, zinc supplementation (3 to 240 mg/day, median 30 mg/day) was associated with a statistically significant reduction in fasting glucose [98].

A more recent review with a meta-analysis of 32 randomized controlled trials, with a total of 1700 patients from 14 different countries, showed, in the groups treated with zinc supplementation compared to the control groups, a statistically significant reduction in fasting glucose [FG, weight mean difference (WMD): −14.15 mg/dL; 95% CI: −17.36, −10.93 mg/dL], and a homeostasis model assessment for IR (WMD: −0.73; 95% CI: −1.22, −0.24) and HbA1c (WMD: −0.55%; 95% CI: −0.84, −0.27%). In particular, the greatest reductions were observed in subjects already diagnosed with T2DM and in those receiving supplementation with inorganic zinc [99]. The mean dose of elemental zinc used in the studies analyzed was 35 mg/day (range: 4–240 mg/day; mean: 30 mg/day), with a duration ranging from 1 to 12 months. Different anions have been used in the various preparations, including sulphate, gluconate, amino chelate, oxide, and acetate; in five studies, however, the type of anion used was not specified [99].

In conclusion, therefore, zinc-based supplementation could have a potential role as an adjunctive treatment in the prevention and management of prediabetes and T2DM, particularly in subjects who do not reach the RDA in zinc intake.

Moreover, the alterations of selenium homeostasis in the context of an altered glucose metabolism are arousing great interest in the scientific community. It represents a fundamental component of selenic proteins, which play an important role in redox homeostasis, in the metabolism of thyroid hormones, and in the protection from oxidative stress and inflammation. A 2019 study on a population sample from northeast China (189 cases, of which 12 subjects are with IFG, 15 subjects with IGT, 25 patients with DM1, and 137 with T2DM, and 51 healthy controls) highlighted the presence of significantly higher serum Se levels in the IFG group and markedly reduced urinary concentrations in the IGT group. Furthermore, blood Se levels were positively associated with serum Zn in subjects with IFG and IGT, and urinary Se with urinary Zn and Cu in the IGT group alone [91]. Furthermore, the same group of researchers had also investigated serum and urinary Zn and Cu in the same sample, finding significantly higher serum Cu levels in subjects with IFG and IGT, in agreement with the results of Serdar et al. [92].

In conclusion, the results obtained suggest the importance of the further investigation of the role of it in glucose metabolism.

In a 2014 clinical trial, lasting seven years, the association was investigated between the intake of magnesium, assessed using the semi-quantitative food-frequency questionnaire (FFQ), both through foods and through the use of supplements, and the risk of developing IFG (≥5.6 to <7.0 mmol/L), IGT (2 h post-load glucose ≥7.8 to <11.1 mmol/L), IR, or hyperinsulinemia (≥90th percentile of homeostasis model assessment of IR or fasting insulin, respectively) in 2582 participants (55% female), aged 26 to 81, with normal glucometabolic status at baseline. Subjects with the highest magnesium intake (upper quintile: 356–651 mg/day, mean 395 mg/day) showed a 37% lower risk of impaired glucose metabolism than those with the lowest intake (lower quintile range: 101–258 mg/day, mean 236 mg/day) [93]. In line with these results, Kieboom et al., in the context of the population-based Rotterdam Study (8555 participants, mean age 64.7 years; mean follow-up 5.7 years), found that a reduction in serum magnesium levels equal to 0.1 mmol/L is associated with an increased risk of T2DM and prediabetes (understood as IFG) (HR 1.12 [95% CI 1.01, 1.25]), with only 13.4% of the effect of serum magnesium levels mediated by IR [94]. Similar results had already emerged, in 2008, from the study by Guerrero-Romero et al. on 1122 subjects (age 20–65), of which 817 were re-examined after 10 years, in which hypomagnesemia was associated with the development of IGT (relative risk 1.38, 95% confidence interval, 1.1–6.3) and IGT + IFG (1.49, 95% confidence interval, 1.1–4.9), but not with the development of IFG alone [95].

In conclusion, an adequate intake of magnesium can play an important role in the management of glucose metabolism. In case of insufficient dietary intake, in the presence of prediabetes, magnesium supplementation may be useful.

Regarding calcium intake during juvenile-adulthood, a recent 2019 cohort study evaluated its association with the development of IGF (*n* = 1134, M and F; baseline age between 3 and 18). Baseline dietary calcium intake (assessed by 48 h FFQ), 1019 ± 366 mg/day for females and 1270 ± 514 mg/day for males, as well as long-term mean intake (1181 ± 340 mg/day for females and 1398 ± 424 mg/day for males) were not correlated with the risk of developing impaired glucose metabolism in adulthood [96]. In a double-blind, randomized, controlled clinical trial of 314 healthy adults, however, daily supplementation with 500 mg of calcium citrate and 700 I.U. of vitamin D for three years was associated with a smaller increase in fasting glucose than placebo (0.02 mmol/L [0.4 mg/dL] vs. 0.34 mmol/L [6.1 mg/dL], respectively, *p* = 0.042) and HOMA-IR (0.05 vs. 0.91, *p* = 0.031) in participants with baseline IFG (*n* = 92) [89]. In conclusion, the results of this study demonstrated that, in healthy, older adults with IFG, supplementation with calcium and vitamin D (500 mg of calcium citrate and 700 IU of vitamin D) may attenuate increases in glycemia and IR that occur over time.

In conclusion, if dietary calcium intake is not adequate, it is useful to suggest calcium supplementation (500 mg/day) to subjects with prediabetes.

Another mineral, whose defect appears to be associated with IFG and dyslipidemia, is chromium: in 130 male patients with a history of myocardial infarction and 130 controls, serum chromium was inversely correlated with blood glucose values in both cases and in controls (r = −0.189, *p* < 0.05 and r = −0.354, *p* < 0.00001, respectively) [97]. However, a 2002 systematic review and meta-analysis involving 15 randomized clinical trials involving a total of 618 participants, 193 of whom had T2DM and 425 were healthy or found to have IGT, found no relationship between the concentration of chromium and that of glucose and insulin in non-diabetic subjects [100]. On the other hand, with reference to the effects of a daily supplementation with chromium picolinate, a randomized, controlled, double-blind, cross-over clinical trial examined the effects of a six-month supplementation with chromium picolinate (500 or 1000 mcg/day) versus placebo in 59 subjects with IFG, IGT, or metabolic syndrome. The authors found no changes in fasting and 2 h oral glucose levels, insulin levels, and HOMA-IR [90]. A 2010 review did not reveal any significant improvement in glucose metabolism either in patients with metabolic syndrome or in subjects with IGT [101].

In conclusion, there are inadequate data to recommend chromium supplementation in patients with prediabetes.

### 4.7. Osteoporosis

The scientific literature has been studying the correlation between T2DM and osteoporosis for some time.

Paschou and colleagues aimed at reviewing the updated information on osteoporosis and fracture risk in patients with T2DM. They discussed the effects of T2DM treatment on bone metabolism, as well as the effect of anti-osteoporotic drugs on T2DM incidence and control, while providing personalized guidance for the optimal management of these pathologies. Their “guide to optimal management” was published in 2017. To this purpose, the scientific literature (human studies) existing up to 2017 was reviewed, and a classification system as defined by the American College of Physicians was adopted for the recommendations.

In summary, the results showed that both a healthy diet and physical exercise are very important for the prevention and treatment of both pathologies [201].

As Ferrari and colleagues also state in a review published in 2018, fragility fractures are increasingly recognized as a complication of both type 1 and T2DM, with the risk of fracture increasing with disease duration and poor glycemic control. Since the identification and management of fracture risk in these patients is challenging, the review investigated the clinical features of bone fragility in adults with T2DM to find evidence in the literature and studies evaluating parameters such as bone mineral density (BMD), bone microstructure and material properties, biochemical markers, and fracture prediction algorithms (e.g., FRAX) in these patients. Furthermore, the impact of T2DM drugs on bone and the efficacy of osteoporosis treatments in this population were examined and, eventually, an algorithm was proposed by the authors for the identification and management of diabetic patients at an increased risk of fracture.

With regard to T2DM, an increased risk of fracture was reported in some of the studies analyzed in the review, but not in others. In a middle-aged population of 33,000 people, T2DM was the strongest predictor of low-energy fracture in both men and women, with relative risks of 2.38 and 1.87, respectively. In a meta-analysis of patients with T2DM, the relative risk of hip fractures in men was 2.8 and, in women, it was 2.1.

With an OR of approximately 1.5 for osteoporotic fractures in patients with T2DM, only approximately 4% of the overall osteoporotic fracture burden is statistically attributable to T2DM. However, considering the increasing prevalence of T2DM and the fact that the condition itself may also be associated with an increased risk of falls (with damage), fragility fractures increasingly appear as a serious but overlooked complication of this disease. Nonetheless, the link between T2DM and skeletal health receives only cursory attention in osteoporosis guidelines and even less in clinical T2DM guidelines [202].

Finally, this year, Liu Y. and colleagues published a multistage cross-sectional study to investigate the effects of sarcopenia, osteoporosis, and osteopenia on spine fractures in prediabetic patients by collecting and analyzing data from the U.S. National Health and Nutrition Examination Surveys from 2009 to 2018. A total of 23,825 subjects were included in the study, including 7427 with prediabetic youth. Bone mineral density and skeletal muscle mass index (SMI) information were measured with DXA. The diagnosis of vertebral fracture was based on DXA results and history.

As for bone health, the results showed that the lumbar and spinal bone mineral density of the prediabetes group was lower than in the healthy group, while there were no differences between diabetic and healthy subjects (this is probably explained by the fact that the diabetic group was overweight). The prevalence of osteoporosis was higher in diabetics than in prediabetics, and higher in the latter than in healthy subjects. The same was true for the prevalence of spinal fractures among the three groups. The SMI trend was also higher in the diabetic group than in the prediabetic group and in the prediabetic group than in the healthy group, probably again due to the excessive weight of the subjects of these two groups. By adding data related to some variables, namely, age, sex, race, BMI, etc., it can be stated that prediabetes is not an independent risk factor for osteoporosis since there was no significant increase in osteoporosis among subjects with prediabetes (OR 0.91, 95% CI 0.78–1.05). For fracture risk, the results showed that sarcopenia is not an independent risk factor for vertebral fractures in the prediabetic population, whereas osteoporosis and osteopenia are. But, adjusting for variables, both sarcopenia and osteoporosis were positively associated with vertebral fractures in all subject groups (OR 4.44, 95% CI 1.76–11.21, and OR 2.90, 95% CI 1.85–4.56, respectively), with a higher prevalence in osteosarcopenia sufferers (OR 6.63; 95% CI, 1.34–2.94) compared to those affected only by sarcopenia or osteoporosis. In conclusion and summary, both sarcopenia and osteoporosis are risk factors for spine fracture in prediabetic adults, and the combination of sarcopenia and osteoporosis further increases the prevalence of spine fracture [102].

In conclusion, from the various studies and scientific evidence, the link between prediabetes and osteoporosis has been known for years, understood as an increased risk of experiencing a decreased bone mineral density and, therefore, an increased risk of fracture in this population. The causes of this link are not entirely clear but there are many possible factors at play: from pathophysiological ones such as microangiopathy at the bone tissue level, discussed as a possible cause of diabetic osteopenia, to molecular ones (it has been demonstrated that insulin, IGF-1 and IGF-2, cytokines, and hormones have an influence on bone metabolism itself such as determining changes in the bone metabolism of subjects with glycemic imbalances). It is, therefore, evident how important the prevention of a chronic and progressive pathology such as osteoporosis is, also, and above all in this population of subjects. The latter have to undergo a regular monitoring of bone mineral density and patient management procedures that include the right tests and correct calcium and vitamin D supplements.

### 4.8. Fruits and Vegetables

The relationship between fruit and vegetable consumption and prediabetes has been investigated by numerous studies.

In a Swedish court study [103] in which 6961 men and women aged between 35 and 56 were enrolled, the relationship was studied between lifestyle, in particular, fruit and vegetable consumption, and the incidence of prediabetes and T2DM during the mean follow-up of 20 years. During the follow-up, 1024 patients developed T2DM and 870 a prediabetic condition. An inverse relationship was seen between fruit and vegetable consumption and the development of a dysglycemia condition with an HR of 0.76 (95% CI 0.58–1.00). In the analysis of the subtypes, an increased incidence of T2DM was noted in those who consume banana, cabbage, and tomato, while a reduction in the incidence was noted with the consumption of apples and pears.

In the study by Li et al. [104], the court in question consisted of 79,922 patients aged over 40 years and the information regarding fruit consumption was collected on the basis of administered questionnaires. Baseline and 2 h post-load glucose measurements were measured at baseline and during follow-up. During an average follow-up of about four years, 7.36% of the participants developed T2DM. Overall, a trend was noted whereby the risk of T2DM decreases (every 100 g more per day corresponds to 2.8% less risk) as the intake of fruit increases. This risk especially decreased in normoglycemic patients in whom the consumption of more than seven portions of fruit per week decreased the risk of developing T2DM by 48%. On the other hand, fruit consumption did not lead to a reduction in the risk of progression towards a frank condition of T2DM in patients already in a prediabetic situation.

Another Chinese cross-sectional study [105] involved 6802 participants between 18 and 65 years old. The correlation between fruit and vegetable consumption and the development of T2DM was seen to be positive when analyzing the female sample, while this relationship was not found in the male sample. If the risk of prediabetes is assessed, however, this relationship is valid for both sexes, especially males. A reduction in the risk of prediabetes was seen with a consumption of fruit and vegetables corresponding to the third and fourth quartile, i.e., between 320 and 530 g (including the weight of vegetables + fruit/day).

Zhang’s study [106] looked at fiber intake and the risk of prediabetes among Chinese adults. The prospective study involved 18,085 participants who—at baseline—had neither T2DM nor prediabetes or other CVDs nor cancer. In the follow-up period, 63,175 person/years, 4139 cases of T2DM occurred. The amount of fiber was quantified in the study as weight per 1000 Kcal introduced. The population under examination was divided into quartiles, in which the third and fourth quartiles introduced 12.7 g/1000 Kcal and 19.2 g/1000 Kcal of fibers, respectively. The results were that fiber intake was inversely related to the incidence of prediabetes. The study also demonstrated that the type of fiber that was found to be most effective in preventing prediabetes is soluble fiber, mostly found in fruit and vegetables.

In addition to fibers, the literature has shown that the polyphenols contained in fruit and vegetables can play a fundamental role in glycemic control.

A 2017 narrative review summarized the evidence from clinical and prospective observational studies linking dietary polyphenols to prediabetes and T2DM, with particular attention to foods rich in polyphenols, characteristic of the Mediterranean diet. In addition, metabolic biomarkers related to polyphenol intake and genotype–polyphenol interactions that modulate the effects on T2DM have been described. Dietary polyphenols come primarily from plant-based foods including fruits, vegetables, whole grains, coffee, tea, and nuts. Polyphenols may affect blood sugar and T2DM through several mechanisms, such as promoting glucose uptake into tissues and thereby improving insulin sensitivity. The intake of polyphenols, particularly flavan-3-ols, and their dietary sources have demonstrated beneficial effects on IR and other cardiometabolic risk factors. Several prospective studies have shown inverse associations between polyphenol intake and T2DM. The Mediterranean diet and its key components—olive oil, nuts, and red wine—have been inversely associated with IR and T2DM. To some extent, these associations can be attributed to the high amount of polyphenols and bioactive compounds in typical foods conforming to this traditional dietary pattern. Few studies have suggested that genetic predisposition may modulate the relationship between polyphenols and T2DM risk. In conclusion, polyphenol intake may be beneficial for both IR and T2DM risk [197].

Even the intake of magnesium, which fruits and vegetables are rich in, especially green leafy vegetables, can play an important role in controlling glucose metabolism. Animal studies have shown that a low-magnesium diet can lead to impaired insulin secretion and action [203].

Moreover, magnesium is an essential cofactor for multiple enzymes involved in glucose metabolism and has been discovered to play a role in the development of T2DM [108,204].

Studies in humans also suggest a significant inverse association between magnesium intake and T2DM risk and supports the dietary recommendation to increase consumption of major food sources of magnesium, such as whole grains, nuts, and green leafy vegetables.

In a study that followed 85,060 women and 42,872 men, magnesium intake was evaluated using a validated food-frequency questionnaire every 2–4 years. After 18 years of follow-up in women and 12 years in men, the study documented 4085 and 1333 incident cases of T2DM, respectively. After adjusting for age, BMI, physical activity, family history of T2DM, smoking, alcohol consumption, and history of hypertension and hypercholesterolemia at baseline, the relative risk (RR) of T2DM was 0.66 (95% CI 0.60–0.73; *p* for trend < 0.001) in women and 0.67 (0.56–0.80; *p* for trend < 0.001) in men, comparing the highest with the lowest quintile of total magnesium intake. The results of this study confirmed the inverse association between magnesium intake and T2DM risk [107].

Moreover, as regards green leafy vegetables, a meta-analysis demonstrated that, for green leafy vegetables, the summary relative risk of T2DM for an increase of 0.2 serving consumed/day was 0.87 (95% CI 0.81 to 0.93) without heterogeneity among studies (*p* = 0.496, *I*^2^ = 0%), confirming that high green leafy vegetable intake is associated with a significantly reduced risk of T2DM [108].

Moreover, in the systematic review by Banaszak [205], it emerges that people who adopt a vegetarian diet have better blood parameters: glucose, insulin, total and LDL cholesterol, and the resulting HOMA IR. These subjects have a higher consumption of fruit and vegetables on average than omnivores.

In conclusion, all the studies found that a consumption of fruit (particularly pears and apples) and vegetables equal to an average of 500 g per day led to a reduction in the development of prediabetes and T2DM. This result can be explained both by the supply of fibers, especially soluble, and water, and by the presence of micronutrients such as vitamins and minerals, especially magnesium, and polyphenols, present in fruit and vegetables.

### 4.9. Nuts

Numerous studies have evaluated the correlation between the intake of nuts (rich in unsaturated fatty acids, fibers, antioxidant vitamins, minerals, and other bioactive compounds), and prediabetes.

The randomized clinical study with two parallel arms by Gulati et al. [109] in which the effects of a dose of 20 g of almonds before lunch and dinner was evaluated over three months. Sixty-six participants with prediabetes in the range of 18–60 years were enrolled; thirty patients per arm completed the study. In the blood parameters in the group with almonds, there was a reduction in the HOMA IR index, total cholesterol, and LDL cholesterol. Above all, a reduction in the normoglycemic state was seen in 23.3% of participants, a reduction comparable to the efficacy with a treatment with acarbose. The intake of almonds before main meals would, therefore, seem to help in the prevention of the evolution from prediabetes to frank T2DM.

In the randomized parallel group study by Cascas et al. [110], 50 patients with metabolic syndrome were instructed to consume 30 g of dried fruit per day (15 g walnuts, 7.5 g almonds, and 7.5 g hazelnuts). The nut group reduced fasting insulin by 2.60 μU/mL (95% CI, −4.62 to −0.59) and HOMA by 0.72 (−1.28 to −0.16) (*p* < 0.05). Among inflammatory markers, IL6 levels decreased by 1.1 ng/mL (−2.7 to −0.1; *p* = 0.035), although, adjusting for weight loss, the correlation was attenuated.

In the study by Hou et al. [111], the effect of peanuts and almonds in the context of a low-carbohydrate diet on cardiometabolic and inflammatory parameters in patients with T2DM was compared. A total of 32 patients were recruited, of which 17 were assigned to the peanut group and 15 to the almond group in a parallel study. Over the three-month follow-up period, there were significant pre–post changes in fasting and postprandial blood glucose (*p* < 0.05). The HbA1c value was reduced in the almond group (*p* < 0.05). Consumption of peanuts and almonds did not increase BMI and had no effect on plasma lipid levels or interleukin-6.

In the randomized parallel group study by Wien et al. [112], 65 people with prediabetes were recruited. The dietary intervention lasted 16 weeks in which the almond intake was approximately 20% of the daily caloric intake, approximately 60 g/day.

The intervention group showed both a reduction in insulin (−1.78 µU/mL vs. +1.47 µU/mL, *p* = 0.002), HOMA IR (−0.48 vs. +0.30, *p* = 0.007), and HOMA 2B (−13.2 vs. +22.3, *p* = 0.001) compared with the control group without almonds. LDL decreased (−12.4 mg/dL vs. −0.4 mg/dL). There were no changes in BMI (−0.4 vs. −0.7 kg/m^2^, *p* = 0.191), systolic blood pressure (−4.4 mm Hg vs. −3.5 mm Hg, *p* = 0.773), or other cardiovascular risks.

Studies have also been conducted in which food products with nuts, such as bars, were used. For example, in the randomized crossover study by Lu et al. [113], the postprandial glycemic response was evaluated from 0 to 120 min after the ingestion of a nut-based, high-protein snack bar or an isocaloric bar containing more carbohydrates. The bar used contained a caloric quantity of about 1000 KJ. Ten overweight Chinese men with a mean age of 47.9 years and a mean BMI of 25.5 with the presence of abundant pancreatic and hepatic ectopic fat tissue assessed using DEXA and MRI were enrolled in the study. In the patients who received the bar rich in dried fruit, there was a reduction in blood sugar (*p* < 0⋅05) in the 30–120 min after ingestion of both the bar alone and the bar associated with 50 g of white bread (a food that normally raises blood sugar), with an area under the glycemic curve 10 times lower.

It has been hypothesized that some polyphenols contained in dried fruit, in particular, ellagic acid, may be the substances that counterbalance lipoinflammation and are involved in glucose control. In fact, as shown by the review by Guasch Ferrè et al., polyphenols promote glucose uptake in the tissues and increase insulin sensitivity [197].

In the review by Kang et al. [206], the mechanisms involved in the metabolic benefit of ellagic acid are discussed in detail, in particular, its role as an epigenetic and intestinal microbiota regulator. It is mainly contained in raspberries, strawberries, apples, walnuts, and pecans. Ellagic acid may be a promising strategy to ameliorate obesity-related metabolic complications, independent of weight loss.

In conclusion, the consumption of dried fruit—in the average dose of 30 g per day—was protective against IR, IFG, and HbA1c. Some studies show a reduction in inflammatory markers, but more studies are needed to establish the correlation between nuts and levels of pro-inflammatory cytokines, such as IL6. It has been hypothesized that some polyphenols contained in dried fruit, such as ellagic acid, may be the substances that counterbalance lipoinflammation and are involved in glucose control.

### 4.10. Hydration

#### 4.10.1. Plain Water

Proper hydration plays a crucial role in preventing or influencing the evolution of prediabetes into T2DM.

In 2015, Carroll and colleagues published a cross-sectional study with the aim of investigating the correlation between water intake and risk of developing T2DM in an adult population in the United Kingdom. The authors hypothesized that a higher water intake could be associated with a lower risk of T2DM.

The survey evaluated—in 138 adult subjects—the risk of T2DM, blood pressure, fruit and vegetable intake, and beverage intake. The T2DM risk score was calculated using the Diabetes UK risk assessment tool validated for use in the UK. This tool measures seven key risk factors for T2DM: age, gender, body mass index (BMI), waist circumference, ethnicity, family history of first-degree T2DM, and use of antihypertensive medications/history of hypertension.

Dietary variables were assessed using a food-frequency questionnaire (FFQ). The FFQ investigated the consumption trends over the previous seven days and only fruit and vegetables were measured in terms of diet. From a list of 38 fruits and vegetables, participants were asked to indicate their average intake (“never”, “once”, “2–4 times”, “5–6 times”, “once a day”, “2–3 a day”, “4–5 a day”, or “6+ times a day”). Since fizzy water was not included in the original FFQ, it was added to the survey in the same format as other beverages. Water was measured in beakers and an information card indicated that 1 beaker equaled 240 mL (standard measure). Participants were asked to report their average intake of a variety of beverages (including, but not limited to, alcoholic, caffeinated, sweetened, and artificially sweetened beverages) in the previous seven days. The FFQ, therefore, made it possible to evaluate the average daily intake of still water and the total average intake of drinks and fruit and vegetables (portions/day).

The results, confirming the authors’ hypothesis, showed that water intake was negatively correlated with the risk of developing T2DM, since those who consumed greater quantities of plain water showed a lower risk. A clear trend of low water consumption emerged in groups classified as being at a high risk for T2DM. Total beverage intake was not associated with risk of T2DM, and no significant differences in total intake were observed between risk groups. Finally, the analyses showed that each 240 mL portion of water drunk per day was associated with a 0.72-point decrease in the risk of T2DM. With the mean daily water intake of 567 mL, the mean risk reduction in each participant was 1.7 points.

In conclusion, although no significant differences were found in water intake between the various risk groups, a negative correlation was observed between water intake and risk score: an increased risk of T2DM was, in fact, associated with a lower water intake, confirming the hypothesis of Harriet A. Carroll and colleagues and suggesting that water intake may play a significant role in the development and prevention of T2DM [114].

In 2016, Carroll and colleagues also published another cross-sectional study, conducted from 2008 to 2012, on a total of 456 men and 579 women with an average age of 44 years. The aim of the study was to analyze the association between simple water intake and the HbA1c trend in the National Diet and Nutrition Survey (2008–2012) rolling survey. The data collected include a four-day food diary and HbA1c values from blood sampling. The analyses used linear and logistic regressions stratified by gender while modelling the association of still water intake per day (240 mL) with HbA1c and probabilities of HbA1c ≥ 5–5%, respectively. Sugary drinks, fruit juices, and artificially sweetened drinks have been transformed with modelling systems into still water.

After adjustment, 1 glass/day of water was found to be associated with a reduction in HbA1c of 0–4% (95% CI 0–7, 0–2) in men, and, in logistic regression, men had a 22% (95% CI 10.32%) reduced chance of HbA1c ≥ 5–5%/day cup of plain water. There was no evidence of an association with HbA1c or odds of HbA1c ≥ 5–5% in women. None of the substitution models was associated with a change in HbA1c probabilities ≥ 5–5%. Still water intake was associated with lower HbA1c in men but not in women. Replacing water with specific beverages was not associated with a reduction in the odds of HbA1c ≥ 5–5%, suggesting that adding water is more relevant [115].

The mechanisms that potentially associate a greater intake of water with better glycemic control can be summarized as follows: surely, the consumption of water represents an indicator of a healthy lifestyle, especially if it is associated with physical activity and a diet including a reduced intake of sugars and more healthy food. Furthermore, still water intake is arguably linked to an increase in satiety, which leads to a potential reduction in energy expenditure. These factors contribute to improving blood sugar both indirectly (through weight stability) and directly (through lower and/or lower blood glucose concentration peaks). The ingestion of water also contributes to hydration and, thus, to the reduction of arginine vasopressin secretion (the hormone that regulates blood pressure and which plays an important role in glycemic control) and to the increase in plasma volume (consequently, decreasing the concentration plasma glucose), both of which influence glucose homeostasis.

In summary, in the first study published by Harriet A. Carroll and colleagues in 2015, an increased risk of T2DM was observed where there was a lower water intake, suggesting the potential positive impact of a good water intake on the risk of developing T2DM. In the second study of 2016 it was observed that, in the male population examined, 1 glass/day of water was associated with a reduction in HbA1c of 0–4% and, in the logistic regression, men always had a reduced probability of 22% (95% CI 10.32%) HbA1c ≥ 5–5% for each daily cup of plain water consumed.

In conclusion, following what has been reported in the literature, it can be stated that not only does water appear to be an essential element for normal metabolism, but it can also be specifically associated with a reduction in the risk of T2DM and, therefore, make it clear how important the intake of simple water and the right hydration are in both the prevention and influence on the evolution of prediabetes into T2DM.

Given what is reported on calcium intake in the above chapter on minerals and on the relationship between osteoporosis and T2DM, it could be interesting to advise people with prediabetes to drink mineral waters rich in calcium.

The consumption of 2 L of calcium-rich mineral water (when the calcium content is higher than 150 mg/L) is useful for reaching the calcium daily requirements because it is a valuable source of highly bio-available calcium.

#### 4.10.2. Sugary Beverages

As regards the correlation between the consumption of sugary beverages (SSB) and the onset of T2DM, there are several studies in the literature concerning both young people and adults.

Duke published a cohort study in 2021 to examine the relationships between sugary beverage (SSB) intake and reported prediabetes in US adolescents. Sugar-sweetened drinks are marketed in different forms, including nondiet pop or soda, flavored juice drinks, sports drinks, sweetened coffee and tea, coffee drinks, energy drinks, and electrolyte-replacement solutions. A variety of sugars may be added to sweeten these beverages as they are processed (e.g., brown sugar, corn sweetener, corn syrup, fructose, high fructose corn syrup, honey, molasses, raw sugar, and sucrose) [207].

The Duke cohort study analyzed data coming from a 2019 Minnesota student survey (N = 125,375). We used logistic regression to examine the relationships between SSB intake frequencies and youth-reported prediabetes in analytical models while adjusting for demographic and other cardiometabolic indicators. Further analyses examined the relationships between fruit juice, milk, and water consumption, and prediabetes.

The results showed that 1 in 4 adolescents reported consuming at least one SHW per day. In adjusted models, a range of SSB intake frequencies were significantly associated with increased odds of prediabetes. However, all frequencies of water intake were associated with a reduction in the odds of prediabetes.

We therefore conclude that efforts to reduce SSB intake among adolescents are warranted in order to support cardiometabolic health. The results of the study are consistent with the current guidelines that indicate water as the preferred drink for adolescents’ hydration needs, regardless of whether or not they are at risk of developing metabolic problems [207].

The risk of T2DM associated with SSB intake was also quantified among adults. Using the results from three large US prospective cohorts of women and men, Drouin-Chartier et al. found that an increase of sugary beverage intake by more than 0.5 servings per day was associated with a 16% higher risk of T2DM over a subsequent four-year period. Additionally, replacing a daily serving of SHW with water, coffee/tea (the amount of sugar and dairy was not measured), or skim milk (0–2%) was associated with a reduction in the risk of T2DM by 2–10%.

In the study published in 2019 by Drouin-Chartier et al., we investigated the association between long-term changes due to the consumption of sugary drinks (including 100% fruit juices) and drinks containing artificial sweeteners (ASBs), together with the consequent risk of T2DM.

The three large prospective studies analyzed by the authors followed 76,531 women in the Nurses’ Health Study (1986–2012), 81,597 women in the Nurses’ Health Study II (1991–2013), and 34,224 men in the Health Professionals’ Follow-up Study (1986–2012). Changes in beverage consumption (i.e., eight-ounce servings per day) were calculated via food-frequency questionnaires administered to subjects every four years. Multivariable Cox proportional regression models were used to calculate hazard ratios for T2DM associated with changes in beverage consumption. The results of the three cohorts were pooled using a meta-analysis.

During 2,783,210 person-years of follow-up, the authors documented 11,906 incident cases of T2DM. After adjusting for BMI and initial and final covariates of dietary and lifestyle changes, an increase in the total consumption of sugar-sweetened beverages (including both sugar-sweetened beverages and 100% fruit juices) by >0.5 servings per day over four years was associated with a 16% (95% CI 1%, 34%) higher risk of T2DM in the following four years. An increase in ASB consumption by >0.5 servings per day was associated with an 18% (2%, 36%) higher risk of T2DM. Replacing a daily serving of sugary drinks with water, coffee, or tea—no ASBs—was associated with a 2–10% lower risk of T2DM.

The conclusions of the study are that an increased consumption of sugary drinks or ASBs was associated with a higher risk of T2DM, although the latter association may be influenced by reverse causality and surveillance biases [119].

Another interesting topic is considering non-sugar sweeteners (NSSs), such as acesulfame potassium, aspartame, saccharin, sucralose, steviol glycosides, cyclamate, neotame, advantame, and luo han guo, that are widely used to replace added sugars in the diet; the studies on the association between their use and blood glucose control in patients with alterations of glucose metabolism are inconclusive. Moreover, all the studies were conducted on diabetic subjects and the position papers are also dedicated exclusively to diabetic subjects and not to subjects with prediabetes. In two RCTs, conducted in 52 subjects, levels of fasting blood glucose were 0.16 mmol/L lower in the groups receiving aspartame or a combination of NSSs than in groups receiving sugar (95% confidence interval −0.26 to −0.06); no differences were observed in plasma insulin levels or in insulin resistance and β-cell function as measured by the homoeostatic model assessment of insulin resistance [116,117]. On the contrary, as reported by a systematic review and meta-analyses, observational studies showed that consuming NSS had long-term negative effects, such as an increased risk of overweight, and type 2 diabetes [120].

Recently, a new guideline from the World Health Organization (WHO) advises against using NSSs to regulate weight or prevent non-communicable diseases [121]. Unfortunately, individuals with diabetes, who frequently utilize NSSs as a means of preserving glycemic control, were not given any advice by the WHO guideline [118]. According to the American Diabetes Association’s (ADA) 2023 standards of care for people with diabetes, when consumed in moderation, NSSs may be a suitable substitute for sugar-sweetened products. The standards also suggest that NSSs do not appear to have a significant impact on glycemic control, though it is unclear how they will affect weight management. But the ADA’s recommendations are also based on studies that did not look at every kind of non-sugar sweetener (acesulfame potassium, which is commonly present in foods and beverages, was not studied) [122]. The Academy of Nutrition and Dietetics practice guidelines for adults with type 1 and type 2 diabetes examined individual types of NSSs for managing glycemia in adults with diabetes, despite the lack of long-term studies; the report concluded that adults with diabetes should be informed that intake of aspartame, sucralose, and steviol glycosides, within the acceptable daily intake (ADI) levels, will not have a significant influence on glycemic control; however, studies that looked at the effects of saccharin, acesulfame potassium, and neotame intake on glycemic outcomes in this population were not included [123].

In conclusion, as reported by Hedrick [124], considering the intake of non-sugar sweeteners, the lack of specificity in studying NSS, the discrepancies in the findings of observational versus randomized controlled trials, the fact that NSSs are heterogeneous compounds that may affect different metabolic pathways with various impacts on health, and other methodological aspects, make it difficult to draw firm conclusive recommendations for or against the use of NSSs in a specific population subgroup such as prediabetes subjects.

In conclusion, these studies agree in confirming the hypothesis that there is a close correlation—in both young people and adults—between the consumption of SSBs and the development of T2DM, while reiterating how higher frequencies of water intake have instead been associated with a reduction in the odds of prediabetes.

### 4.11. Alcohol

As far as red wine is concerned, there are studies showing that red wine consumption can be beneficial in certain quantities. Wine can be beneficial as it contains some bioactive compounds such as polyphenols (anthocyanins, flavones, alkyphenols, phenolic acids, lignans, and stilbenes) [208,209].

Polyphenols may influence glycemia and T2DM through different mechanisms, such as promoting the uptake of glucose in tissues, therefore improving insulin sensitivity, as reported in a narrative review by Guash-Ferre [197].

The meta-analysis by Bajunas et al. [131] included 20 cohort studies that compared the relative risk of developing T2DM for both men and women in response to the introduction of red wine every day. The study showed that both men and women had a lower relative risk of developing prediabetes than those who did not consume it and those who consumed more than 50 g in women and 60 g in men. The risk-lowering consumption is 22 g of ethanol in men and 24 g in women.

In the case–control study by Rochitte et al. [126], two groups of people were retrospectively compared, 101 people drinking red wine and 104 with complete abstinence from alcohol. The 205 people are healthy males between the ages of 50 and 75. The group that drank red wine consumed one glass a day for 4–5 days a week for at least 5 years before the analyses. FPG was lower among wine drinkers than non-drinkers (97.6 ± 18.2 vs. 118.4 ± 29.6 mg/dL; *p* < 0.02) and, thus, HDL cholesterol was higher in wine drinkers (46.9 ± 10.9 vs. 39.5 ± 9.0 mg/dL; *p* < 0.001). In retrospect, it is, therefore, possible to understand that the habit of drinking one glass of red wine a day has led to better glycometabolic parameters.

In the prospective cohort study by Blomster et al. [127], during an average follow-up of 5 years, a cohort of 11,140 people with T2DM was studied and 3389 of these took alcohol. Participants were determined to be eligible for the study with an age over 55 years, if they were diagnosed with T2DM after 30 years of age, and had a history of major macro- or micro-vascular complication. The women enrolled who consumed alcohol were 593 or 17% of the drinking group. Those who consumed moderate alcohol (less than 21 units of alcohol per week in men and less than 14 units of alcohol per week for women) saw a reduction in cardiovascular events (adjusted hazard ratio [aHR] 0.83; 95% CI 0.72 −0.95; *p* = 0.008), fewer micro-vascular complications (aHR 0.85; 95% CI 0.73–0.99; *p* = 0.03), and a reduced all-cause mortality (aHR 0.87; 96% CI 0.75–1.00; *p* = 0.05). These benefits were seen in people who drank mostly red wine.

In the two-year randomized clinical trial by Gepner et al. [125], 224 subjects were assigned to one of three groups: the first group was assigned to drink 150 mL of mineral water at dinner, the second group was assigned 150 mL of white wine, and the third group was assigned 150 mL of red wine for two years. Calorie and carbohydrate consumption between the various groups was comparable. The red wine group increased (HDL-C) by 2.0 mg/dL (95% CI, 0.04 to 0.06 mmol/L [1.6 to 2.2 mg/dL]; *p* < 0.001), apolipoprotein A1 by 0.03 g/L (CI, 0.01 to 0.06 g/L; *p* = 0.05), and decreased the ratio of total cholesterol to HDL by 0.27 (CI, −0.52 to −0.01; *p* = 0.039). No differences were seen in blood pressure, adiposity, liver function, drug therapy, symptoms, or quality of life in the three groups. Sleep quality improved in the wine drinking groups. Overall, the group consuming one glass of red wine per day reduced the number of positive parameters for diagnosing metabolic syndrome by 0.34 (CI, −0.68 to −0.001; *p* = 0.049).

Conversely, alcohol abuse led to an increased risk of prediabetes.

In the study by Li et al. [128], the aim was to examine the association between the average consumption of alcohol with the incidence of T2DM or with a condition of dysglycemia and to note associations with the polymorphism of the genes coding for aldehyde dehydrogenase (ALDH2) and alcohol dehydrogenase (ADH1B). The participating patients were all older than 50 years: 15,726 participants were without T2DM; 11,232 were without T2DM and without IFG. Of these, 1624 patients corresponding to 10.33% developed T2DM, and 1004 (8.94%) developed IFG during the four-year follow-up. Adjusting for other confounders, occasional or moderate alcohol use was not associated with the risk of hyperglycemia (IFG + T2DM) (odds ratio (OR) = 1.10, 95% confidence interval (CI) 0.95–1.27, and 0.90 (0.69–1.18), respectively), while high consumption increased the incidence of dysglycemia (T2DM + IFG) (OR = 1.82, 95% CI 1.24–2.68). No interactions of sex, BMI, and ADH1B/ALDH2 genetic polymorphisms were seen. In the 2011 study by Cullman et al. [129] a different behavior of alcohol emerged in the two sexes. This cohort study analyzed 2070 men and 3058 women with normoglycemia and 70 men and 41 women with prediabetes. Total alcohol consumption and binge drinking increased the risk of prediabetes and T2DM in men (OR 1.42, 95% CI 1.00–2.03 and OR 1.67, 95% CI 1.11–2.50, respectively), while low consumption reduced the risk of T2DM in women (OR 0.41, 95% CI 0.22–0.79). Men had a higher risk of prediabetes with a high beer consumption (OR 1.84, 95% CI 1.13–3.01) and T2DM with a high alcohol consumption (OR 2.03, 95% CI 1.27–3.24). Women showed a reduced risk of prediabetes, with a high wine consumption (OR 0.66, 95% CI 0.43–0.99), and T2DM, with medium consumption of both wine and spirits (OR 0.46, 95% CI 0.24–0.88 and OR 0.55, 95% CI 0.31–0.97, respectively), while heavy alcohol consumption increased the prediabetes risk (OR 2.41, 95% CI 1.47–3.96).

In the cross-sectional analytical study by Suebsamran et al. [130], a relationship was seen between alcohol consumption and prediabetes. The total 383,442 patients enrolled were divided into six different groups based on the amount of alcohol consumed. The prevalence of prediabetes was 10.5% (11.2% and 9.7% in men and women, respectively). After adjustment for other risk factors, alcohol consumption was independently associated with prediabetes, with a dose–response relationship (adjusted odds ratio (OR of 1.80, 95% CI 1.53–2.11, *p* < 0.001 and 1.47, 95% CI 1.28–1.68, *p* < 0.001) for those who drank alcohol daily and 3–4 times a week, compared with non-drinkers), but those who drank 1–2 times/month had a reduced risk of prediabetes (OR = 0.89, 95% CI, 0.82–0.97, *p* = 0.006). Similar results were seen for men. Women who drank occasionally had a significantly reduced risk of prediabetes, compared with women who did not drink (OR 0.95, 95% CI 0.91–0.99, *p* = 0.039). In the study, it is clear that alcohol consumption increases the risk of prediabetes in a dose-dependent manner, although sporadic consumption seems to decrease the incidence.

Based on the literature reviewed, it can be stated that there is no safe dose of alcohol. It has been seen that the condition of prediabetes acts synergistically with alcohol in increasing the risk of pancreatic and biliary tree tumors and stroke, especially in geriatric patients. Drinking alcohol in a condition of dysglycemia leads to a more rapid progression to a condition of frank T2DM in a dose-dependent manner. Occasional consumption (one or two times a month) has been shown to be safe in some studies.

In conclusion, the analysis of the literature shows that the consumption of 1 glass of red wine a day during a meal was found to be protective in prediabetes and reduce the risk of disease progression and macro-vascular and micro-vascular complications of diabetic disease. The risk-lowering consumption is 22 g of ethanol in men and 24 g in women. The beneficial effect would seem to be elicited by the various polyphenolic compounds present in red wine. Polyphenols may influence glycemia and T2DM through different mechanisms, such as promoting the uptake of glucose in tissues, and, therefore, improving insulin sensitivity. Conversely, alcohol abuse leads to an increased risk of prediabetes.

### 4.12. Coffee

Coffee is one of the most consumed beverages in the world. It is commonly taken in roasted coffee form and, as such, it represents a complex blend of over 1000 bioactive compounds, some with potentially therapeutic antioxidant, anti-inflammatory, or anticancer effects. The main active compounds include caffeine, chlorogenic acids, and diterpenes cafestol and kahweol [135].

A systematic review and a dose–response meta-analysis by Ding demonstrated that coffee consumption was consistently associated with a lower risk of T2DM [136].

Specifically, compared with no coffee consumption, the results of this systematic review and meta-analysis, based on 1,109,272 study participants and 45,335 T2DM cases, demonstrate that consuming six cups of coffee per day was associated with a 33% lower risk of T2DM. Consumption of caffeinated coffee and decaffeinated coffee was both associated with a lower risk of T2DM. The association between coffee consumption and T2DM risk was consistent for men and women and for European, US, and Asian populations. A total of 218 meta-analyses were considered, including 201 meta-analyses of observational studies and 17 meta-analyses of randomized controlled trials.

There are several plausible biological mechanisms that may contribute to the inverse association between coffee consumption and T2DM risk: chlorogenic acid, a phenolic compound, is a major component of coffee and has been shown to reduce blood glucose concentrations in animal experiments [137]. Chlorogenic acid (CGA) may reduce intestinal glucose absorption by competitively inhibiting glucose-6-phosphate translocase and by reducing sodium-dependent glucose transport into vesicles of the brush border membrane [132], by reducing oxidative stress due to its antioxidant properties, and by reducing hepatic glucose production [134].

Meng’s review, which reports the role of chlorogenic acid in the regulation of glycolipid metabolism, describes chlorogenic acid (CGA) as an insulin sensitizer that potentiates insulin action similarly to the therapeutic action of metformin. CGA was found to promote a significant reduction in peak plasma glucose in the oral glucose tolerance test, most likely by attenuating intestinal glucose absorption, indicating a possible role of CGA as a glycemic-index-lowering agent and highlighting it as a compound of interest for reducing the risk of developing T2DM.

CGA exerts its antidiabetic effects by stimulating glucose uptake in both insulin-sensitive and insulin-resistant adipocytes. Furthermore, CGA, unlike thiazolidinediones (TZDs) or insulin, does not induce obesity or other side effects [138].

Interesting is Yangimoto’s randomized, double-blind, placebo-controlled crossover study in which they examined the effective dose of green tea catechins (GTCs) and coffee chlorogenic acid (CCA) on postprandial glucose, insulin, and incretin responses to a high-fat, carbohydrate cookie meal containing 75 g of glucose in healthy men. Study 1 (*n* = 18) evaluated two doses of GTC (270 or 540 mg) containing a fixed dose of CCA (270 mg) with 113 mg caffeine and a placebo (0 mg GTC and 0 mg CCA) with 112 mg caffeine. Study 2 (*n* = 18) evaluated two doses of CCA (150 or 300 mg) containing a fixed dose of GTC (540 mg) and a placebo with 99 mg caffeine. The combined single ingestion of GTC and CCA significantly impaired the incretin response and suppressed the glucose and insulin levels. These results suggest that the minimal effective dose is 540 mg of GTC and 150 mg of CCA [133].

In conclusion, the scientific literature has shown that coffee consumption is significantly associated with a lower risk of T2DM. Specifically, when compared with no coffee consumption, consumption of six cups of coffee per day was associated with a 33% lower risk of T2DM. Chlorogenic acid, among the numerous bioactive compounds present in coffee, would be the main compound responsible for this positive activity.

### 4.13. Physical Activity

Exercise is increasingly recognized as a form of therapy in individuals with T2DM, as it is effective in improving fitness and reducing HbA1c and other cardiovascular risk factors, as well as the dosage/number of medications taken by hypoglycemics [140].

In “Nutrition Therapy for Adults with Diabetes or Prediabetes: A Consensus Report” for patients with T2DM, a moderate-intensity physical activity of at least 150 min per week is recommended [19].

As regards the studies that consider the efficacy of glycemic control in patients with prediabetes, these are less numerous, but nonetheless provide interesting indications.

The aim of the meta-analysis systematic review by Angélica Trevisan De Nardi et al. of 2018 was to compare the effects of high-intensity interval training (HIIT) versus moderate-intensity continuous training (MICT) on functional capacity and cardiometabolic markers in individuals with prediabetes, comparing previously published studies.

The duration of the studies ranged from two to four weeks for the prediabetes patient groups. The included studies used different HIIT and MICT protocols. Exercise types, session length, and intensity varied widely between studies. All exercises were performed after meals and were monitored by direct supervision or objective measurements using heart rate monitors or accelerometers. Additionally, subjects were instructed not to alter their dietary and medication intake habits during the study period.

Both modalities—HIIT and MICT—showed similar effects on systolic and diastolic blood pressure in individuals with prediabetes. High-intensity interval training, requiring a smaller time commitment, has the potential to be used as a treatment modality for people with prediabetes or T2D. This type of training induces cardiometabolic adaptations similar to those of MICT [141].

The study by Jung ME et al. of 2015 [139] compared high-intensity interval training (HIIT) with traditional moderate-intensity continuous training (MICT). In fact, according to the authors, HIIT leads to improvements in various cardio-metabolic health markers, but the adherence to HIIT following a supervised laboratory intervention has yet to be tested. The researchers compared self-report and objective measures of physical activity after one month of independent exercise in individuals with prediabetes who were randomized to HIIT (*n* = 15) or MICT (*n* = 17).

The individuals involved were all with prediabetes ranging in age from 30 to 60. After completing 10 supervised exercise sessions, the participants were asked to perform HIIT or MICT three times a week for four weeks.

Individuals randomized into the HIIT group (89 ± 11%) adhered to the prescribed protocol to a greater extent than individuals in MICT (71 ± 31%) as determined by exercise logs completed over the one-month follow-up (*p* = 0. 05, Cohen’s d = 0.75). Minutes spent in vigorous physical activity per week as measured by the accelerometer were higher in HIIT (24 ± 18) than in MICT (11 ± 10) at one-month follow-up (*p* = 0.049, Cohen’s d = 0, ninety-two). Cardiorespiratory fitness and systolic blood pressure assessed at the one-month follow-up were similarly improved (*p* < 0.05). In conclusion, this study provides preliminary evidence that individuals with prediabetes can adhere to HIIT in the short term and do so at a higher level than MICT. These findings support the potential utility of HIIT as an alternative exercise strategy that could strengthen exercise adherence by allowing patients to save and optimize exercise time [139].

A systematic review and meta-analysis by Jadhav RA et al. comparing and analyzing randomized controlled trials of individuals diagnosed with prediabetes and interventions delivered in the form of physical activity were included in this review. Adiponectin, leptin, C-reactive protein, interleukin-6, and tumor necrosis factor-α were the outcome measures considered. The authors found that physical activity promotion with or without dietary or lifestyle modifications did not affect adiponectin level; however, it did show a positive effect on leptin in individuals with prediabetes. There is a dose–response relationship of adiponectin and leptin with weight loss in human participants, indicating that a slight weight loss of up to 5% reduces the leptin level, but, to make a change in the level of adiponectin, weight reduction would have to be greater than 10%. The prediabetes stage is associated with an increased level of inflammatory markers, namely, CRP, IL-6, and TNF-α. IL-6 promotes the expression of leptin on mRNA and inhibits the expression of adiponectin. This explains the impact of physical activity on adiponectin and leptin reducing IL-6. Weight reduction causes a reduction in the size of adipocytes and a reduction in the secretions of inflammatory cytokines. IL-6 concentration is related to adipocyte size. However, TNF-α secretion is independent of adipocyte size. The cytokine production literature has also proposed that IL-6 suppresses TNF-α production. This review suggests that the physical activity promotion program may reduce the level of leptin and IL-6, but whether there is any effect on the level of adiponectin, CRP, and TNF-α in individuals with prediabetes is uncertain [142].

In conclusion, regular exercise can help prevent the progression of prediabetes to full-blown T2DM. Several studies, already collected in the meta-analysis, have shown that subjecting these patients to “high-intensity” training or moderate intensity training sessions leads to similar effects on systolic and diastolic blood pressure and on cardiometabolic adaptations in individuals with prediabetes. However, high-intensity interval training, requiring a lower time commitment, has the potential to be used as a treatment modality for people with prediabetes or T2D, positively impacting compliance, and one session of HIIT would also seem to have greater and more lasting effects on the reduction of the postprandial glucose increase.

Physical activity also seems to be able to modulate and reduce the level of leptin and interleukin-6 in these patients who, by definition, have an increase in pro-inflammatory markers.

### 4.14. Dietary Supplementation with Probiotics

The intestinal microbiota is not only a simple component of the gastrointestinal tract, but seems to have a specific role in the pathogenesis of many metabolic diseases, such as obesity [210], T2DM [143], and non-alcoholic fatty liver disease [211].

Evidence for the involvement of the gut microbiota in the regulation of glucose metabolism and the progression of T2DM is accumulating. Understanding microbial dysbiosis and the specific alterations in gut microbiota composition that occur during the early stages of glucose intolerance is of paramount importance, as recently reported in the systematic review by Letchumanan [150].

The factors that contribute to the diversity and interindividual variability of the microbiota are many and are related to genetics, nutrition and lifestyle, some drugs, such as antibiotics, and changes in the composition of the diet [212].

In the pathogenesis of prediabetes and T2DM, inflammatory response, nutrition, intestinal permeability, glycolipid metabolism, insulin sensitivity, and energy homeostasis play an important role [151].

It was observed that the number of bacteria responsible for the production of short-chain fatty acids (SCFAs) was lower in patients with T2DM [143]. An altered gut microbiota can lead to decreased SCFA production and increased inflammation, and can affect insulin secretion and pancreatic β-cell sensitivity, leading to IR [213]. SCFAs, especially butyrate, promote the secretion of GLP-1, which prevents glucagon secretion, inhibits gluconeogenesis in the liver, and improves insulin sensitivity [214]. Additionally, SCFAs may prevent low-grade inflammation caused by the migration of bacteria from the gut into the mesenteric adipose tissue and blood [215]. In conclusion, these data suggest that increasing SCFAs, especially butyrate, is important for preventing and controlling prediabetes.

Lipopolysaccharides (LPSs), components of the outer cell membrane of Gram-negative bacteria, can be found in high concentrations and be absorbed from the intestine [144]. LPS stimulates the immune system by binding to the toll-like receptor (TLR), triggering immune cells to release inflammatory cytokines, which promote IR caused by an endotoxin-induced inflammatory response [216]. Furthermore, a study conducted on animal models demonstrated how the intestinal microbiota protects against the development of obesity, metabolic syndrome, and prediabetes with an immune-mediated mechanism, inducing specific commensal Th17 cells. A diet rich in fats and sugars has been shown to promote the metabolic syndrome by depleting those micro-organisms that induce Th17; conversely, the recovery of commensal Th17 cells restores their protective effect. Indeed, it has been observed that the loss of protective Th17 cells is induced by diet, in particular, by simple sugars. The elimination of sugar from diets has shown a protective role against obesity and metabolic syndrome in the animal model through a mechanism dependent on specific commensal Th17 cells. Sugar and ILC3 promote the growth of bacterial species, such as Faecalibaculum rodentium, which dysregulate the Th17-inducing microbiota [217].

Another potential mechanism associated with intestinal ecosystem homeostasis is the endocannabinoid system. LPS interacts with endocannabinoid receptors (eCB1), modulating the intestinal permeability and translocation of LPS, increasing circulating levels of LPS and inducing metabolic endotoxemia [218]. Cholic acid and chenodeoxycholic acid are primary bile acids (BAs) produced from cholesterol in the liver and converted to secondary BAs in the intestine [219,220]. Furthermore, BAs are involved in the regulation of glucose homeostasis as signalling molecules and cellular receptors, directly activating nuclear farnesoid X receptor (FXR) and Takeda G protein-coupled receptor 5 (TGR5), signalling and indirectly promoting the FXR-dependent induction of intestinal fibroblast growth factor-19 (FGF19) [221]. TGR5 activation can induce pre-proglucagon gene expression and GLP-1 secretion [214,222]. Conversely, FXR activation suppresses pre-proglucagon gene expression and GLP-1 secretion by inhibiting glycolysis and ChREBP activity in L cells [223]. Thus, in intestinal endocrine L cells, BA acts through the opposing effects on TGR5 and FXR to regulate GLP-1 production and secretion, thereby maintaining weight loss and improving glucose tolerance.

Another mechanism related to the action of the microbiota on prediabetes involves intestinal bacteria that metabolize nutrients to produce trimethylamine (TMA), which is then converted into trimethylamine N-oxide (TMAO) in the liver. TMAO levels have been shown to be elevated in patients with T2DM [145]. Additionally, animal models have shown that nutrient-derived TMAO can exacerbate IGT and increase fasting insulin levels by blocking the hepatic insulin signalling pathway and causing inflammation in adipose tissue [145]. Although a prospective study demonstrated that a higher intake of phosphatidylcholine (the precursor to the generation of TMAO) was independently associated with an increased risk of T2DM [146], the association between TMAO and T2DM has not reached a consistent conclusion. Roi et al. observed that plasma TMAO levels are associated with an increased prevalence of prediabetes in a nonlinear manner but not related to IR or FPG [147].

Thus, overall, the gut microbiota influences host metabolic disorders through the modulation of metabolites, including SCFA, LPS endotoxin, BA, and TMAO, as well as mediating the interaction between the gastrointestinal system and other organs [152].

As regards the composition of the microbiota in prediabetes, in a 2018 Danish case–control study, it was demonstrated that individuals with this condition have an altered intestinal microbiota compared to healthy subjects, characterized by a decrease in the genus Clostridium and the mucin-degrading bacterium Akkermansia munichipila. These results are comparable to observations made in chronic diseases characterized by chronic low-grade inflammation [148].

A study published in the journal EBioMedicine in the same period came to different conclusions, especially with regard to the bacterium Akkermansia munichipila. The authors used a combination of in-depth metagenomics and metaproteomic analyses of stool samples from treatment-naïve (TN-DM2, *n* = 77), prediabetic (prediabetes, *n* = 80), and normoglucose-tolerant (NGT, *n* = 97) individuals with T2DM to study the relative functional and compositional changes in the gut microbiota, and the fecal content of microbial and host proteins to elucidate possible host–microbial interactions, characterizing the different stages of the disease, from prediabetes to treatment-naïve T2DM [149].

In total, 11,980 meta-proteins and 425 human proteins were identified. The analysis allowed us to observe several distinctive features in prediabetes and NGT compared to TN-DM2, such as a higher abundance of Akkermansia muciniphila and a lower abundance of Bacteroides spp. The first is a well-known mucin-degrading bacterium which, in some studies, has already been shown to be able to reduce some pathologies included in metabolic syndrome, both in mice and in humans. On the other hand, the relative abundance of several Firmicutes species that produce butyrate was lower in prediabetes and TN-DM2 than in NGT. These findings are in line with a gradual progression of the disease through prediabetes to overt T2DM. Specifically, the authors found higher abundances of Enterobacteriaceae species (dominated by *E. coli*) and lower levels of host proteins that are potentially involved in Proteobacteria-specific responses in prediabetes, such as galectin-3 and proteins within the immunoglobulin super-family. An increased abundance of intestinal Enterobacteriaceae has been widely reported in patients with metabolic diseases such as obesity and atherosclerotic cardiovascular disease, and in patients with chronic inflammatory bowel disease. These unique traits, associated with prediabetes, could link potential gut microbial signals to an increase in chronic low-grade systemic inflammation.

The authors also noted that pancreatic enzyme content differed in fecal samples among the three groups of individuals studied. These results suggest that unique and distinctive nonlinear changes to the gut ecosystem may exist in individuals with prediabetes prior to the transition to overt T2DM. The authors of the study themselves emphasize that further large-scale longitudinal follow-up studies will be needed to delineate how microbial functions change from prediabetes to T2DM and to address the nature of the interactions between the gut microbiota and the host, in the transient phases leading to frank T2DM, also in a future therapeutic perspective.

A subsequently published systematic review (2022) summarized the existing evidence regarding the composition and diversity of the microbiota in individuals with prediabetes and individuals newly diagnosed with T2DM (newDM) compared to individuals with normal glucose tolerance (nonDM). A total of 18 observational studies (5489 participants) that analyzed the gut microbiota of subjects with prediabetes and newDM were included. Low microbial diversity was found overall in both prediabetes and newDM compared with nonDM. Differences in gut microbiota composition between prediabetes and newDM and those with nonDM were inconsistent across included studies. Four of the eighteen studies found an increased abundance of phylum Firmicutes, together with a decrease in the abundance of Bacteroidetes, in the new DM. A decrease in *Faecalibacterium prausnitzii*, *Roseburia*, *Dialister*, *Flavonifractor*, *Alistipes*, *Haemophilus,* and *Akkermansia muciniphila* and an increase in the abundance of *Lactobacillus*, *Streptococcus*, *Escherichia*, *Veillonella,* and *Collinsella* in the disease groups (prediabetes and newDM) were observed at the genus/species level in at least two studies. In four studies, Lactobacillus was also found to correlate positively with FPG, HbA1c, and/or IR (HOMA-IR). This calls for further investigations on the species/strain-specific role of the endogenously present *Lactobacillus* in the glucose regulation mechanism and in the progression of T2DM disease. Differences in dietary intake caused significant variations in the variety of bacterial species [150].

A 2016 review of randomized controlled trials (RCTs) suggested that prebiotics have a neutral effect on body weight, decreasing fasting and postprandial blood glucose, and improving insulin sensitivity and lipid profile, while it appears that some inflammatory markers are decreased, sometimes substantially (20–30%). As for probiotics, these have shown significant but limited effects on body weight (<3%) and metabolic parameters. The effect was mainly observed with fermented milk or yoghurt as opposed to administration in capsule form and with consumption for at least eight weeks of multiple rather than single bacterial strains. Pickled and fermented foods, especially vegetables and legumes, could serve as a dietary source of pre-, pro-, and synbiotics. These foods have shown possible benefits with respect to morbidity and mortality in prospective cohort studies [152].

A more recent systematic review including randomized controlled trials also reached similar conclusions, with inconsistent results among the included studies, partly due to the limited sources. The authors highlighted that probiotics can reduce HbA1c and have the potential to improve blood glucose levels after oral glucose loading. Probiotic supplementation can suppress the increase in blood cholesterol, but the improvement cannot be verified. Prebiotics failed to show a clear improvement in glycemic control, but their use led to changes in the gut microbiota composition. A combination of probiotics and prebiotics through symbiotic supplementation is more effective than probiotics alone in glycemic control. The review concluded that the benefits of gut microbiota modulation in the treatment of prediabetes have been partially demonstrated. However, there is not enough evidence to show significant benefits on glucose metabolism, lipid metabolism, and body composition [153].

A 2022 meta-analysis that included seven publications, for a total of 460 patients, however, demonstrated how probiotics were able to significantly reduce HbA1c levels, quantitative insulin sensitivity check index (QUICKI), total cholesterol, triglycerides, and LDL cholesterol compared to the levels in the placebo group, while the effects on fasting blood glucose, HOMA index, and HDL cholesterol were not different from those of the placebo group. The meta-analysis, therefore, concluded that probiotics may play an important role in the regulation of HbA1c, QUICKI, total and LDL cholesterol, and TG in patients with prediabetes. Furthermore, based on existing studies, probiotics can regulate blood glucose homeostasis in various ways [154].

In the same year (2022), a systematic review reached similar conclusions stating that the administration of probiotics can provide beneficial and salutary effects in the clinical management of patients with prediabetes and metabolic syndrome. Different probiotic compositions have shown beneficial and remarkable effects on glucose homeostasis, lipid profiles, BMI, and inflammatory markers in subjects with prediabetes and metabolic syndrome, and healthy individuals, and could be beneficial in rebalancing the gut microbiota in the prediabetic condition [155].

Animal models of T2DM show that specific probiotic bacteria improve glucose control. Benefits have been seen with probiotics containing *Lactobacillus acidophilus*, *Lactobacillus casei*, *Lactobacillus plantarum*, *Lactobacillus gasseri*, *Lactobacillus reuteri*, and *Lactobacillus rhamnosus*. For example, *Lactobacillus casei* improved glucose tolerance, lowered lipid levels, boosted immunity, and reduced oxidative stress, while *Lactobacillus johnsoniii* improved intestinal barrier integrity. *Bifidobacterium lactis* has also been shown to lower lipid and insulin levels [156].

Regarding the duration of probiotic treatment, a 2016 meta-analysis suggested that the effect of probiotics on glucose metabolism was potentially greater when consumed for more than eight weeks or when multiple strains of probiotics were consumed simultaneously [157].

A currently highly regarded research topic is the study of the microbiota of patients with prediabetes. Scientific literature agrees in demonstrating that the intestinal microbiota plays an important role in regulating glucose metabolism. It was observed that the number of bacteria responsible for the production of short-chain fatty acids (SCFAs) was lower in patients with T2D. Decreased SCFA production leads to increased inflammation and can affect insulin secretion and pancreatic β-cell sensitivity, leading to IR. These data suggest that elevation of SCFAs, particularly butyrate, is important in glycemic control in prediabetes. Furthermore, a study conducted on animal models demonstrated how the intestinal microbiota protects against the development of obesity, metabolic syndrome, and prediabetes with an immune-mediated mechanism, while inducing specific commensal Th17 cells. The gut microbiota influences host metabolic disorders through the modulation of several metabolites, such as LPS endotoxin, BA, and TMAO, as well as SCFA.

As regards the composition of the microbiota in patients with prediabetes, to date, the studies do not agree, but they do agree in stating that the intake of probiotics (multiple strains for at least eight weeks) could be useful in managing blood sugar metabolism in subjects with prediabetes.

### 4.15. Monitoring Glucose Levels

Monitoring blood glucose levels is a daily routine for diabetic people. Education programs emphasize the importance of the proper disposal of needles to prevent contamination and the transmission of blood-borne viruses. Despite these precautions, outbreaks of hepatitis have been reported in settings where multiple T2DM patients engage in self- or assisted monitoring of blood glucose. In the United States, 18 outbreaks of hepatitis B virus (HBV) infection were investigated, all linked to the improper use of blood-glucose-monitoring equipment [162,163,224].

Monitoring glucose levels can be challenging, and patients often spend significant periods with high blood sugar levels (outside the target range). Intensive insulin administration, in these cases, have been associated with an increased risk of death, while more frequent assessments of blood sugar levels have been shown to reduce mortality and morbidity in critically ill individuals, leading to improved outcomes [158,164,165].

Regular self-monitoring of blood sugar levels helps individuals with T2DM understand how factors such as food intake, physical activity, stress, and illness affect their glucose levels. Frequent monitoring allows people to maintain a good quality of life and make necessary adjustments to their medication, ultimately improving their long-term health. By reducing the risk of T2DM-related complications such as heart attacks, strokes, and damage to the eyes, kidneys, and nerves, individuals can achieve better overall health outcomes [166,172].

As for patient compliance, traditional biological fluids (e.g., blood) require invasive sampling techniques like painful finger pricking that entail evident discomfort and health risks, such as infections and viral transmission. Hence, there is increasing attention being paid towards non-invasive sampling. Today, different biological fluids like blood (gold standard), serum, urine, cerebrospinal fluid, etc. are used as diagnostic tools, but they need precautions and skilled personnel to be collected with few complications. Despite advancements in technology, most of commercially available glucometers still require invasive sampling methods, such as venipuncture or finger-pricks, which can be uncomfortable for frequent measurements. Alternatively, there are implantable devices available, but they are not cost-effective and are typically reserved for specific chronic patients, making widespread monitoring of the growing global diabetic population impractical [167,168,169].

Blood has been extensively studied and widely used as a biomarker source for diagnostic purposes. Blood has a higher glucose concentration compared to other biofluids; however, it presents challenges such as rapid coagulation, intense red color, and a high concentration of biomolecules. These factors increase the likelihood of interference-related issues or limited specificity. Therefore, in most cases, in vitro analyses are performed on serum samples, often requiring dilution of up to 100 times to mitigate these challenges [225,226].

As a result, there is a growing interest in non-invasive sampling techniques. In this regard, the use of saliva as a diagnostic fluid is emerging as a viable option in clinical practice. Non-invasive biofluids offer various advantages, such as eliminating the need for needle-based sampling, reducing physical discomfort and anxiety (particularly for frequent measurements), not requiring trained personnel, thereby reducing healthcare costs, while eliminating the risks associated with blood-borne pathogens (a safer approach), and having a greater stability due to no rapid coagulation. Moreover, urine and saliva sampling are simple and can be easily self-collected, making them ideal for home-testing purposes, even when a relatively large amount is needed [227,228].

Although blood glucose monitoring is more commonly practiced, urine sample testing is valuable for assessing potential kidney dysfunctions. Glycosuria is primarily associated with untreated T2DM and, in rare cases, a syndrome where urine hyperglycemia corresponds to normal blood glucose levels. However, anomalous glucose concentrations in urine are predominantly linked to T2DM.

Typically, glucose is not present in urine or it is in very low concentrations. However, in cases of elevated blood glucose levels, glucose is excreted into urine. Generally, when the glucose concentration exceeds 50–100 mg/dL (2.77–5.55 mM), a urine test is considered positive for hyperglycemia. Since urine is stored in the bladder for a certain period before being excreted, the measured glucose concentration represents an average of blood glucose levels during that time. Therefore, shorter collection intervals result in more accurate measurements and a stronger correlation with blood glucose fluctuations [170,171,229].

In order to measure glucose levels in sweat, it is necessary to utilize wearable devices, such as skin patches. Among the various strategies for detecting glucose in sweat, wearable electrochemical sensors based on nanoparticles (NPs) have been extensively explored. The fabrication of these wearable skin pads involved transferring the system onto a transparent and biocompatible chitosan film. In vivo testing of this system was conducted on the back area of the neck of several volunteers before and after sleeping. However, this strategy has certain limitations. The sweat secretion changes drastically over the day and in the case of physical exercise or pathological conditions. Additionally, it is challenging to visually distinguish samples due to the low difference in glucose amounts in sweat, while the long assay time of 60 min could restrict the practicality and applicability of the sensor [159,230].

The glucose concentration can vary greatly among these biofluids, with some cases exhibiting glucose levels nearly two orders of magnitude lower than in blood. Achieving the necessary sensitivity for glucose detection in these biofluids requires technological advancements [231].

Saliva is a transparent, mildly acidic fluid that is secreted by various salivary glands, including the parotid (20–25% of total secretion), submandibular (70–75% of total secretion), sublingual, tubarial, and minor glands. It is a combination of mucus and serous secretions. The primary components of saliva include water (98% composition), mucopolysaccharides, glycoproteins, electrolytes, white blood cells, epithelial cells (which carry DNA), proteins, and enzymes such as amylase, lipase, and antimicrobial enzymes. The resting pH of saliva typically varies from 6.2 to 7.4. However, this value can change after consuming a meal or in pathological conditions. Saliva encompasses a wide range of molecules that provide insights into both normal and abnormal states. Within human saliva, there are naturally occurring substances present at concentrations representative of various tissues, as well as molecules associated with recreational use or therapeutic purposes. Additionally, saliva serves as a valuable source of markers indicating hormonal, immunological, neurological, and metabolic conditions [173,232,233,234,235].

Currently, there are five primary categories of salivary diagnostic elements that are considered as biomarkers: proteome, microbiome, transcriptome, micro-RNA, and metabolome. Among these, the most common and abundant salivary substances with clinical relevance (validated biomarkers) include microparticles, viruses, bacteria, various proteins, immune system components such as IgA, IgG, and IgM, polynucleotides, lipids like cholesterol and triglycerides, steroid hormones, electrolytes, and small signaling molecules. One attractive aspect of human saliva is its ease of collection, which can be carried out quickly and without the need for specialized training [236,237,238,239].

Saliva serves as an easily accessible biomarker source that can be collected non-invasively, making it highly advantageous for colorimetric tests. Unlike other biofluids, saliva is colorless, further enhancing its suitability for such tests. However, it is essential to ensure proper sampling techniques to avoid misleading results. Saliva samples can be categorized as stimulated or non-stimulated, and, while this distinction may not be critical in some cases, it is particularly important for glucose measurements. To establish a better correlation with blood glucose levels (and potential T2DM risks) and to enhance reproducibility, it is recommended to exclusively use non-stimulated saliva [160,161,240].

Numerous studies have shown a positive correlation between blood glucose levels and salivary glucose levels. However, the existing literature presents contradictory findings due to various factors that influence salivary glucose values, including the collection protocol and the criteria used to enroll subjects in the studies. Nonetheless, P. Balan et al. demonstrated that salivary glucose levels are significantly higher in both uncontrolled diabetic subjects (13.35 ± 6.61 mg/dL) and controlled diabetic subjects (4.95 ± 2.479 mg/dL) compared to non-diabetic subjects (1.18 ± 0.675 mg/dL), which aligns with previous research. Other studies have also reported significant correlations, with varying ratios ranging from 1:40 to 1:100 depending on factors such as the population being studied (healthy or diabetic), age, and body mass index [241,242,243,244,245,246,247,248].

Innovative glucose assessment systems, such as salivary assessment, would, therefore, represent an important support for the patient in terms of quality of life. To date, the results are promising, but many studies still need to be conducted before these assessments can be considered routine for patients with glycemic alterations.

## 5. Conclusions

The global prevalence of prediabetes is increasing. In 2019, the prevalence estimated was 7.5% (373.9 million people). Prediabetes is a risk factor for T2DM, and, for prediabetic individuals, lifestyle modification is the cornerstone to T2DM prevention, with evidence of a 40–70% relative-risk reduction. Given this background, the aim of this review is to evaluate the latest data regarding novel augmentation strategies in prediabetes in order to identify the best lifestyle modification and to construct a food pyramid that allows subjects with prediabetes to easily figure out what to eat, also considering the quality of life; therefore, certain technologies can be of support, such as the innovative salivary glucose monitoring system.

Considering carbohydrate intake, three portions per day of whole-meal carbohydrates with a low glycemic index or potatoes with their skins on represent the ideal foods to recommend to patients with prediabetes. In order to obtain weight loss in an obese patient with prediabetes, the evidence from scientific research shows that, in this case, it may be useful to consider low-carbohydrate diets.

Regarding proteins, they are essential in a balanced diet and evidence in the literature focuses on their important role in the prevention and treatment of the onset of prediabetes.

Foods rich in protein appear to be characterized by a low glycemic index (GI) and the intake of proteins induces a release of insulin in any case lower than that generated by the ingestion of carbohydrates. This suggests that high-protein (HP) diets may help preserve β-cells by increasing insulin sensitivity and decreasing the insulin load per meal.

Furthermore, the higher protein content in the diet can also contribute to maintaining or increasing lean body mass and, consequently, to favoring the uptake of glucose at the muscle level: the skeletal muscle is, in fact, essential for the clearance of glucose being substantially responsible for the postprandial uptake of about 80% of the glucose taken.

Considering milk and dairy products, the scientific literature agrees in demonstrating that a higher intake of milk and cheese, both low-fat and fat-free (three portions of low-fat dairy products, skimmed milk, or low-fat yogurt: 300–400 g/day), is a result associated with better glycemic control.

Whey protein supplementation (10–40 g of whey protein taken before a meal) may also be helpful in glycemic control, particularly in sarcopenic prediabetic patients.

Regarding the intake of red meat and processed meat, it must be occasional, a portion of 100 g no more than one day a week for the egg intake; 2–4 eggs per week should be recommended to patients with prediabetes.

Considering plant-based foods, from the evidence in the literature, it is possible to deduce that a consumption of vegetable proteins is recommended for the purpose of improving glycemic control and IR: a good amount of legumes, between 150 g fresh, corresponding to 50 g dried (standard portion), three times a week up to a maximum of 150 mg/day, is recommended. Furthermore, soy and its derivatives, such as tofu, and peanuts, preferably with skins and also in the form of peanut butter, are a valid addition to the diet as they can all contribute to preventing the progression of IR, typical of prediabetes, in overt T2DM.

Finally, given that the studies that have been carried out up to now, investigating the correlation between prediabetes and sarcopenia, agree in demonstrating that patients with prediabetes had reduced mass, strength, and muscle performance compared to non-diabetics, it is important that patients with prediabetes are screened in order to evaluate the possible presence of this pathology. Finally, given this background, it is equally important that protein intake is adequate in patients with prediabetes.

Regarding lipid intake, there are numerous studies that have investigated the role of fatty acids in prediabetes, in particular, the beneficial role of the intake of foods rich in mono- and polyunsaturated fats. The protective role of the intake of at least 2–3 portions of fish weekly in T2DM is linked to the presence of omega-3 fatty acids EPA and DHA and their anti-inflammatory action, capable of increasing membrane fluidity, and the number and efficiency of insulin receptors. Furthermore, the benefits of fish could also be linked to the content in the latter of proteins and amino acids, capable of increasing the sense of satiety and, consequently, facilitating weight loss. As regards monounsaturated fatty acids, the literature has shown that the daily intake of at least 10 mL per day (optimal 15–20 mL) of extra-virgin olive (EVO) oil represents an important strategy in glucide control in prediabetes. The effectiveness is due, in particular, to the presence of high quantities of polyphenols in the EVO oil.

Regarding vitamins, the evaluation of vitamin D in blood levels is mandatory in patients with prediabetes in order to identify a personalized supplementation of this vitamin. For B group vitamins, since it is possible with routine tests to carry out the evaluation of folate, vitamin B12, and homocysteine in the blood, it is essential that we carry out these analyses on patients with prediabetes in order to supplement in the case of folic acid and vitamin B12 deficiencies. As for vitamin C, personalized supplements of vitamin C could represent a possible future strategy with which to increase the benefits and effectiveness of interventions. However, the results should be interpreted with caution due to the limitations of the primary studies analyzed. Regarding vitamin E, based on the evidence from the randomized controlled trials analyzed, there is insufficient evidence supporting a potential beneficial effect of vitamin E supplementation on improving HbA1c and fasting glucose and insulin concentrations in patients with prediabetes.

With regard to minerals, the evaluation of the dietary intake of some minerals, such as zinc, magnesium, selenium, and calcium, should be considered in patients with prediabetes, in order to evaluate the need for oral supplementation.

Considering the link between prediabetes and osteoporosis, from the various studies and scientific evidence, the link between prediabetes and osteoporosis has been known for years, understood as an increased risk of decreased bone mineral density, and, therefore, as an increased risk of fractures in this population. The causes of this connection are not entirely clear but there are many possible factors at play: from pathophysiological ones such as microangiopathy at the bone tissue level, discussed as a possible cause of diabetic osteopenia, to molecular ones (it has been demonstrated that insulin, IGF-1 and IGF-2, cytokines, and hormones have an influence on bone metabolism itself so as to determine changes in the bone metabolism of subjects with glycemic imbalances). It is, therefore, evident how important the prevention of a chronic and progressive pathology such as osteoporosis is, also, and above all in this population of patients. The latter must sustain the correct monitoring of bone mineral density and patient management that includes the right tests and correct calcium and vitamin D supplements.

As far as fruit and vegetables are concerned, all the studies found that a consumption of fruit (particularly pears and apples) and vegetables equal to an average of 500 g per day led to a reduction in the development of prediabetes. This result can be explained both by the supply of fiber, especially water-soluble fiber, and by the presence of micronutrients such as vitamins and minerals, especially magnesium, and polyphenols, present in fruit and vegetables.

Considering the consumption of nuts, the average dose of 30 g per day was protective against IR, IFG, and HbA1c. Some studies show a reduction in inflammatory markers, but more studies are needed to establish the correlation between nuts and the levels of pro-inflammatory cytokines, such as IL6. It has been hypothesized that some polyphenols contained in dried fruit, such as ellagic acid, may be the substances that counterbalance lipoinflammation and are involved in glucose control.

With regard to hydration, the literature stated that water appears to be an essential element for normal metabolism and an adequate intake of water can also be specifically associated with a reduction of the risk of T2DM for patients with prediabetes.

Given what is reported on calcium intake in the section on minerals and on the relationship between osteoporosis and T2DM, it could be interesting to advise people with prediabetes to drink mineral water rich in calcium.

The consumption of 2 L of calcium-rich mineral water (when the calcium content is over 150 mg/L) is useful in order to reach the daily calcium requirement because it is a valuable source of highly bioavailable calcium.

Considering sugary drinks, studies agree in confirming the hypothesis that there is a close correlation in both young people and adults, between the consumption of these beverages and the development of T2DM and in reiterating how higher frequencies of water intake are instead associated with a reduction in the odds of prediabetes.

As far as NSSs are concerned, the lack of specificity in studying NSSs, the discrepancies in the findings of observational versus randomized controlled trials, the fact that NSSs are heterogeneous compounds that may affect different metabolic pathways with various impacts on health, and other methodological aspects make it difficult to draw firm conclusive recommendations for or against the use of NSSs in a specific population subgroup such as prediabetes subjects.

Regarding alcohol intake, the analysis of the literature shows that the consumption of one glass of red wine a day with a meal was found to be protective in prediabetes and reduce the risk of disease progression and macro-vascular and micro-vascular complications of diabetic disease. The risk-lowering consumption is 22 g of ethanol in men and 24 g in women. The beneficial effect would seem to be elicited by the various polyphenolic compounds present in red wine. Polyphenols may influence glycemia and T2DM through different mechanisms, such as promoting the uptake of glucose in tissues, and, therefore, improving insulin sensitivity. Conversely, alcohol abuse leads to an increased risk of prediabetes.

As far as coffee is concerned, the scientific literature has shown that coffee consumption is significantly associated with a lower risk of T2DM. Specifically, compared with no coffee consumption, consumption of six cups of coffee per day was associated with a 33% lower risk of T2DM. Chlorogenic acid, among the numerous bioactive compounds present in coffee, is mainly responsible for this positive activity.

Considering physical activity, regular exercise can help prevent the progression of prediabetes to full-blown T2DM. Several studies, already collected in the meta-analysis, have shown that subjecting these patients to “high-intensity” training or moderate-intensity training sessions leads to similar effects on systolic and diastolic blood pressure and on cardiometabolic adaptations to individuals with prediabetes. However, high-intensity interval training (HIIT), requiring a lower time commitment, has the potential to be used as a treatment modality for people with prediabetes or T2D, positively impacting compliance, and it would also appear that one session of HIIT has a greater and more lasting effect on the reduction of the postprandial glucose increase. Physical activity also seems to be able to modulate and reduce the level of leptin and interleukin-6 in these patients who, by definition, have an increase in pro-inflammatory markers.

Scientific literature recognizes that the intestinal microbiota plays an important role in regulating glucose metabolism. It was observed that the number of bacteria responsible for the production of short-chain fatty acids (SCFAs) was lower in patients with T2D. Decreased SCFA production leads to increased inflammation and can affect insulin secretion and pancreatic β-cell sensitivity leading to IR. The data suggest that the elevation of SCFAs, particularly butyrate, is important in glycemic control in prediabetes. Furthermore, a study conducted on animal models demonstrated how the intestinal microbiota protects against the development of obesity, metabolic syndrome, and prediabetes with an immune-mediated mechanism, inducing specific commensal Th17 cells. The gut microbiota influences host metabolic disorders through the modulation of several metabolites, such as the LPS endotoxin, BA, and TMAO, as well as SCFA. As regards the composition of the microbiota in patients with prediabetes, to date, the studies do not agree, but they do agree in stating that the intake of probiotics (multiple strains for at least eight weeks) could be useful in managing blood sugar metabolism in patients with prediabetes.

## Data Availability

The data presented in this study are available in the article and in the Supplementary Materials.

## Supplementary Materials

The following supporting information can be downloaded at: https://www.mdpi.com/article/10.3390/nu15234943/s1, Table S1. Tabelle CHO; Table S2a. Tabelle Milk and Dairy; Table S2b. Tabelle Eggs; Table S2c. Tabelle Meat; Table S2d. Tabelle Plant-Based Proteins; Table S3. Tabelle sarcopenia; Table S4. Tabelle Lipid; Table S5. Tabelle Vitamins; Table S6. Tabella Minerals; Table S7. Tabelle Osteoporosis; Table S8. Tabella Fruits and vegetables; Table S9. Tabella Nuts; Table S10a. Tabelle Plain water; Table S10b. Tabelle Sugary beverages; Table S11. Tabella Alcohol; Table S12. Tabella Coffee; Table S13. Tabelle Physical activity; Table S14. Tabelle Microbiota; Table S15. Tabelle Monitoring Glucose Levels.
