# Supplementary material for: A Narrative Review on Strategies for the Reversion of Prediabetes to Normoglycemia: Food Pyramid, Physical Activity, and Self-Monitoring Innovative Glucose Devices
Source: Nutrients. 2023 Nov 28;15(23):4943. doi: 10.3390/nu15234943 (PMC10707766; doi:10.3390/nu15234943)
Supplement: Supplementary file 1 [file nutrients-15-04943-s001.zip › Tables Prediabetes Nutrients/1 Tabelle CHO.pdf]

Authors	Type of study	Population characteristics	Type of intervention	Duration	End point	Results	Conclusion	Strength of evidence
Cea-Soriano L et al, 2021	Cohort prospective study	1184 participants with prediabetes based on levels of fasting plasma glucose and/or glycated hemoglobin	Hazard ratios of diabetes onset were estimated by Cox proportional regression models associated to high versus low/medium adherence to Mediterranean diet. Different propensity score methods were used to control for potential confounders.	4,2 years	Evaluate the effect from high adherence to Mediterranean diet on the risk of diabetes	Incidence rate of diabetes in participants with high versus low/medium adherence to Mediterranean diet was 2.9 versus 4.8 per 100 persons-years. The hazard ratios adjusted for propensity score and by inverse probability weighting (IPW) had identical magnitude: 0.63 (95% confidence interval, 0.43-0.93). The hazard ratio in the adjusted model using propensity score matching 1:2 was 0.56 (95% confidence interval, 0.37-0.84).	These propensity score analyses suggest that high adherence to Mediterranean diet reduces diabetes risk in people with prediabetes.	Moderate
Filippatos TD et al, 2016	Cohort Prospective Study	3042 men and women (>18y) were enrolled for the study.	In 2011 and 2012, the 10-year follow-up examinations were performed,	10 years	Examine the effect of the Mediterranean diet on diabetes and CVD risk in	The prediabetic subjects (343) had a significantly higher incidence of diabetes (25%	High adherence to the Mediterranean diet is associated with a low risk of	Moderate

			including a working sample of n = 1875 participants without diabetes at baseline. Adherence to the Mediterranean diet at baseline evaluation was assessed using the MedDietScore (range 0-55).		subjects with impaired fasting glucose (IFG, i.e. fasting plasma glucose 100-125 mg/dl).	vs. 10%, p < 0.001) and CVD (17.8% vs. 12.3%, p = 0.007) compared with subjects with normal glucose values. A significant trend towards lower diabetes and CVD incidence was observed with medium and high adherence to the Mediterranean diet compared with low adherence (p < 0.001). High adherence to the Mediterranean diet (>35/55 score) was associated with lower 10-year incidence of diabetes and CVD.	developing diabetes and CVD in prediabetic subjects	
Saslow LR et al, 2017	Parallel-group randomized (1:1) trial	Randomize 34 adults with glycated hemoglobin (HbA1c) > 6.0% and elevated body weight (BMI > 25) to a LCK diet (n = 16) or a MCCR diet (n = 18)	Participants attended 19 classes, All participants were encouraged to be physically active, get sufficient sleep, and practice behavioral adherence	12 months	Considerable uncertainty exists about the optimal level of carbohydrate intake. Previous research suggests that an ad libitum very low-carbohydrate ketogenic diet	Compared to the MCCR group, the LCK group reported consuming fewer non-fiber grams of carbohydrates (6 and 12 months), more grams of fat (6 and 12 months),	Adults with prediabetes or noninsulin-dependent type 2 diabetes may be able to improve glycemic control with less medication by following an ad libitum very low-	High

			strategies based on positive affect and mindful eating.		(LCK) may improve metabolic measures in adults with T2DM and reduce the need for diabetes-related medications	and more grams of protein (12 months), but not a different number of calories per day	carbohydrate ketogenic diet compared to a moderate-carbohydrate, calorie-restricted low-fat diet.	
Saslow LR et al 2014	Randomized pilot trial	Enrolled and randomized 34 participants to the MCCR (n=18) or LCK (n=16) diet groups.	MCCR diet (medium carbohydrate, low fat, calorie-restricted, carbohydrate counting diet) consistent with guidelines from ADA. LCK diet (very low carbohydrate, high fat, non calorie-restricted diet)whose goal is to induce a low level of ketosis, here referred to as LCK (low carbohydrate, ketogenic)	3 months	Compared a MCCR representative of conventional diabetic diet recommendations to a LCK (≤50 g carbohydrates per day not including fiber) in persons with HbA1c>6.0%. The primary outcome measure was change in glycated hemoglobin (HbA1c) from baseline to 3 months.	Both groups had significant weight loss, even though the LCK group tended to lose more weight that the MCCR group (although only significant to the *p*=0.09 level), even though only the MCCR group aimed to restrict calories.	In overweight and obese individuals with type 2 diabetes, a very low carbohydrate, high fat, non calorie-restricted diet may be more effective at improving blood glucose control than a medium carbohydrate, low fat, calorie-restricted, carbohydrate counting diet that remains the standard for most diabetes education efforts.	High

Gardner CD et al, 2022	Randomized, crossover, interventional trial	40 participants aged ≥18 years with prediabetes or T2DM followed the well-formulated ketogenic diet (WFKD) and the Mediterranean-plus diet (Med-Plus) for 12 weeks each, in random order.	Compared 2 low-carbohydrate diets with 3 key similarities (nonstarchy vegetables, avoid added sugars and refined grains) and 3 key differences (avoid legumes, fruits, and whole, intact grains) for their effects on glucose control and cardiometabolic risk factors in individuals with prediabetes and T2DM.	3 months	The primary outcome was the percentage change in glycated hemoglobin (HbA1c) after 12 weeks on each diet.	The primary analysis was of 33 participants with complete data. The HbA1c values did not differ between diets at 12 weeks.	HbA1c values were not different between diet phases after 12 weeks, but improved from baseline on both diets, likely due to several shared dietary aspects.	High
Li L et al, 2022	Prospective observational study	9793 adults with prediabetes from the NHANES 1999-2014 (age>20years old)	Dietary intake was measured by 24-h recalls in the NHANES. From 1999 to 2002, one 24-h dietary recall was conducted in person in the NHANES Mobile Examination Center. From 2003 to 2014, dietary intake was measured by a 24-h dietary recall for 2 nonconsecutive days.	15 years	Examine the associations of different types of lower-carbohydrate diets (LCDs) and lower-fat diets (LFDs) with mortality among individuals with prediabetes.	Higher healthy LCD score was associated with favorable blood glucose, insulin, HOMA-IR, C-reactive protein (CRP), and blood lipids, whereas higher healthy LFD score was associated with lower blood glucose and CRP at baseline (all P-trend < 0.05).	Healthy LCD and LFD scores were significantly associated with lower all-cause mortality, whereas unhealthy LCD and LFD scores tended to be associated with higher all-cause mortality, among people with prediabetes.	Moderate
