# Supplementary material for: A Narrative Review on Strategies for the Reversion of Prediabetes to Normoglycemia: Food Pyramid, Physical Activity, and Self-Monitoring Innovative Glucose Devices
Source: Nutrients. 2023 Nov 28;15(23):4943. doi: 10.3390/nu15234943 (PMC10707766; doi:10.3390/nu15234943)
Supplement: Supplementary file 1 [file nutrients-15-04943-s001.zip › Tables Prediabetes Nutrients/10a Tabelle Plain water.pdf]

Authors	Type of study	Population characteristics	Type of intervention	Duration	End point	Results	Conclusion	Strength of evidence
Carroll et al, 2015	Cross-sectional study	138 adult subjects	The risk of DM2, blood pressure, fruit and vegetable intake and beverage intake were evaluated. The DM2 risk score was calculated using the Diabetes UK risk assessment tool validated for use in the UK. Dietary variables were assessed using a FFQ. Water was measured in beakers and 1 beaker equaled 240mL.	The past 7 days	Investigate the correlation between water intake and risk of developing type 2 diabetes mellitus in an adult population in the United Kingdom	Water intake was negatively correlated with the risk of developing DM2. A trend of low water consumption emerged in groups classified as high risk for DM2. The analyzes showed that each 240 ml portion of water drunk per day was associated with a 0.72-point decrease in the risk of DM2. The mean risk reduction in each participant was 1.7 points.	A negative correlation was observed between water intake and risk score, suggesting that water intake may play a significant role in the development and prevention of DM2.	Moderate
Carroll et al, 2016	Cross-sectional study	Total of 1,035 subjects (456 men and 579 women, with an average age of 44 years.	Data includes a 4-day food diary and HbA1c values from blood sampling. The analyzes used linear and logistic regressions stratified by gender modeling the associations of glasses per day (240 mL) of still water with HbA1c and probabilities of HbA1c≥5-5%, respectively. Sugary drinks, fruit juices and artificially sweetened drinks have been transformed with modeling systems into still water.	From 2008 to 2012.	Analyze the association between simple water intake and HbA1c trend in the National Diet and Nutrition Survey (2008-2012) rolling survey.	1 glass/day of water was found to be associated with a reduction in HbA1c of -0-04% in men and men had a 22% reduced chance of HbA1c≥5-5%/day cup of plain water. Still water intake was associated with lower HbA1c in men but not in women.	Water can be specifically associated with a reduction in the risk of DM2 and that intake of simple water and the right hydration are very important in the prevention or influence on the evolution of prediabetes into diabetes.	Moderate
