# Supplementary material for: A Narrative Review on Strategies for the Reversion of Prediabetes to Normoglycemia: Food Pyramid, Physical Activity, and Self-Monitoring Innovative Glucose Devices
Source: Nutrients. 2023 Nov 28;15(23):4943. doi: 10.3390/nu15234943 (PMC10707766; doi:10.3390/nu15234943)
Supplement: Supplementary file 1 [file nutrients-15-04943-s001.zip › Tables Prediabetes Nutrients/10b Tabelle Sugary beverages.pdf]

Authors	Type of study	Number of studies	Subjects (total)	End point	Result	Conclusion	Strenght of evidence
Drouin-Chartier et al, 2019	Meta-analysis.	3 large prospective cohort studies.	76,531 women in the Nurses' Health Study (1986-2012); 81,597 women in the Nurses' Health Study II (1991-2013); 34,224 men in the Health Professionals' Follow-up Study (1986-2012).	Investigate the association between long-term changes due to the consumption of sugary drinks (including 100% fruit juices) and drinks containing artificial sweeteners (ASBs) and the consequent risk of DM2	Over 2,783,210 person-years of follow-up, 11,906 incident cases of DM2 are documented. Increase in total consumption of sugar-sweetened beverages by > 0.5 servings per day over 4 years was associated with a 16% higher risk of DM2 in the following 4 years. Increasing ASB consumption by > 0.5 servings per day was associated with an 18% higher risk of DM2. Replacing a daily serving of sugary drink with water, coffee or tea was associated with a 2-10% lower risk of DM2.	Increased consumption of sugary drinks or ASB was associated with a higher risk of DM2, although the latter association may be influenced by reverse causality and surveillance biases	High
