# Supplementary material for: A Narrative Review on Strategies for the Reversion of Prediabetes to Normoglycemia: Food Pyramid, Physical Activity, and Self-Monitoring Innovative Glucose Devices
Source: Nutrients. 2023 Nov 28;15(23):4943. doi: 10.3390/nu15234943 (PMC10707766; doi:10.3390/nu15234943)
Supplement: Supplementary file 1 [file nutrients-15-04943-s001.zip › Tables Prediabetes Nutrients/11 Tabella Alcohol.pdf]

Authors	Type of study	Population characteristics	Type of intervention	Duration	End point	Results	Conclusion	Strength of evidence
Rochitte et al, 2014	Case control study	T205 people healthy males between the ages of 50 and 75.	two groups of people were retrospectively compared, 101 people drinking red wine and 104 with complete abstinence from alcohol	5 years	To evaluete the effects of red wine on glycometabolic patterns	Drinking one glass of red wine a day has led to better glycometabolic parameters	Drinking one glass of red wine is beneficial in preventing T2DM	Moderate
Blomster et al,	Prospective cohort study	11140 people with type 2 diabetes mellitus	To define groups based on their alcohol consumption	5 years	To establish the risk of complications in people with different levels of alcohol consumption	Those who consumed moderate alcohol saw a reduction in cardiovascular events, fewer microvascular complications and reduced all-cause mortality	Moderate alcohol consumption is beneficial for all cause mortality	Moderate
Gepner et al, 2015	Randomized controlled trial	224 subjects	3 groups: one group to drink 150 ml of mineral water at dinner, another 150 ml of white wine and finally 150 ml of red wine.	2 years	To establish any effects of wine on metabolic and diabetes blood markers	No significant differences among groups, except for red wine which can reduce the risk of metabolic syndrome	Red wine in moderate consumption can reduce the risk of metabolic syndrome	High

**Li et al, 2022**	Cohort study	15726 participants	Groups based on daily alcohol consumption	4 years	To assess the association between the average consumption of alcohol with the incidence of type 2 diabetes mellitus	Occasional or moderate alcohol use was not associated with risk of hyperglycemia, while high consumption increased the incidence of dysglycemia	High alcohol consumption is detrimental, while moderate consumption is not associated with risk of disglycaemia	Moderate
Cullman et al, 2011	Cohort study	2070 men and 3058 women with normiglycemia and 70 men and 41 women with prediabetes	Groups based on alcohol consumption in quantity and quality	8-10 years follow-up	To evaluate the effect on glicometabolic patterns of different types and dosages of alcoholic beverages	Both women and men had higher risk with heavy alcohol consumption	Heavy alcohol consumption is detrimental for both sexes	Moderate
Suebsamran et al, 2016	Cross sectional analytical study	383442 patients	Enrolled were divided into 6 different groups based on the amount of alcohol consumed		Relationship between alcohol consumption and prediabetes	After adjustment for other risk factors, alcohol consumption was independently associated with pre-diabetes, with dose-response relationship	The more the alcohol consumption, the more the risk of prediabetes	Moderate

Review and meta-analysis

Authors	Type of study	Number of studies	Subjects (total)	End point	Result	Conclusion	Strenght of evidence
Bajuna et al, 2009	Metanalisis	20 cohort studies	477,200 individuals	To establish a relationship between alcohol consumption and diabetes type 2	Our analysis confirms previous research findings that moderate alcohol consumption is protective for type 2 diabetes in men and women.	Moderate alcohol consumption is beneficial for preventing the risk of diabetes	High
