# Supplementary material for: A Narrative Review on Strategies for the Reversion of Prediabetes to Normoglycemia: Food Pyramid, Physical Activity, and Self-Monitoring Innovative Glucose Devices
Source: Nutrients. 2023 Nov 28;15(23):4943. doi: 10.3390/nu15234943 (PMC10707766; doi:10.3390/nu15234943)
Supplement: Supplementary file 1 [file nutrients-15-04943-s001.zip › Tables Prediabetes Nutrients/12 Tabella Coffee.pdf]

Authors	Type of study	Population characteristics	Type of intervention	Duration	End point	Results	Conclusion	Strength of evidence
Poole, R. et al, 2017	Meta-analysis	201 meta-analyses of observational research with 67 unique health outcomes and 17 meta-analyses of interventional research with nine unique outcomes.	Umbrella review of the evidence across meta-analyses of observational and interventional studies of coffee consumption and any health outcome		To evaluate the existing evidence for associations between coffee consumption and multiple health outcomes	There was evidence of a non-linear association between consumption and some outcomes, with summary estimates indicating largest relative risk reduction at intakes of three to four cups a day versus none, including all cause mortality (relative risk 0.83 (95% confidence interval 0.79 to 0.88), cardiovascular mortality (0.81, 0.72 to 0.90), and cardiovascular disease (0.85, 0.80 to 0.90).	Coffee consumption seems generally safe within usual levels of intake, with summary estimates indicating largest risk reduction for various health outcomes at three to four cups a day, and more likely to benefit health than harm.	High
Ding, M. et al, 2014	dose-response meta-analysis	Twenty-eight prospective studies were included in the analysis, with 1,109,272 study participants and 45,335 cases of type 2 diabetes		The follow-up duration ranged from 10 months to 20 years	association of coffee consumption with the risk of type 2 diabetes	Compared with no or rare coffee consumption, the relative risk (RR; 95% CI) for diabetes was 0.92 (0.90-0.94), 0.85 (0.82-0.88), 0.79 (0.75-0.83), 0.75 (0.71-0.80), 0.71 (0.65-0.76), and 0.67 (0.61-0.74) for 1-6 cups/day, respectively. The RR of diabetes for a 1	Coffee consumption was inversely associated with the risk of type 2 diabetes in a dose-response manner. Both caffeinated and decaffeinated coffee was associated with reduced diabetes risk.	High

						cup/day increase was 0.91 (0.89-0.94) for caffeinated coffee consumption and 0.94 (0.91-0.98) for decaffeinated coffee consumption (P for difference = 0.17).		
van Dam, R.M., 2008	Narrative review	/	/	/	This paper briefly reviews the evidence for a relation between coffee consumption and these conditions, with particular attention to methodological issues	Several early studies suggested that coffee consumption could result in a marked increase in risk of coronary heart disease and several types of cancer. High consumption of unfiltered types of coffee, such as French press and boiled coffee, has been shown to increase low-density-lipoprotein-cholesterol concentrations. In addition, limiting caffeinated coffee intake during pregnancy seems a prudent choice.	In sum, the currently available evidence on coffee and risk of cardiovascular diseases and cancer is largely reassuring, and suggests that, for the general population, addressing other health-related behaviors has priority for the prevention of chronic diseases.	Low

Arion, W.J. et al, 1997	in vitro study				the interactions of chlorogenic acid (CHL) and 2-hydroxy-5-nitrobenzaldehyde (HNB) with the components of the rat hepatic glucose 6-phosphatase (Glc-6-Pase) system	CHL is without effect on the enzyme of fully disrupted microsomes or the system inorganic pyrophosphatase (PPiase) activity. HNB is a potent competitive inhibitor of the system PPiase activity (Ki = 0.56 mm) and a somewhat weaker noncompetitive inhibitor of enzyme activity (Ki = 2.1 mm).	The presence of CHL effectively protects T1, but not T2, against the irreversible inhibition by HNB. In contrast, PPi and Pi are effective in protecting T2, but not T1.	Low
Svilaas A. et al, 2004	Cross-sectional study	a group of 61 adults with corresponding plasma samples and data from a nationwide survey of 2672 Norwegian adults based on an extensive FFQ.			to determine the contribution of various food groups to total antioxidant intake, and to assess the correlations of the total antioxidant intake from various food groups with plasma antioxidants	The total intake of antioxidants was approximately 17 mmol/d with beta-carotene, alpha-tocopherol, and vitamin C contributing <10%. The intake of coffee contributed approximately 11.1 mmol	dietary antioxidants other than the well-known antioxidants contribute to our antioxidant defense. Surprisingly, the single greatest contributor to the total antioxidant intake was coffee	Moderate
Meng, S. et al, 2013	Narrative review				Roles of chlorogenic Acid on regulating glucose and lipids metabolism	Chlorogenic acid (CGA) exerts many biological properties, including antibacterial, antioxidant, and anticarcinogenic activities	the mechanism on glucose and lipid metabolism has not yet been conclusively elucidated.	Low

Yanagimoto, A. et al, 2023	RCT randomized, double-blind, placebo-controlled crossover study		Study 1 (n = 18) evaluated two doses of GTC (270 or 540 mg) containing a fixed dose of CCA (270 mg) with 113 mg of caffeine and a placebo (0 mg GTC and 0 mg CCA) with 112 mg of caffeine. Study 2 (n = 18) evaluated two doses of CCA (150 or 300 mg) containing a fixed dose of GTC (540 mg) and a placebo with 99 mg of caffeine.	1 years	examined the effective dose of GTC and CCA on postprandial glucose, insulin, and incretin responses to a high-fat and high-carbohydrate cookie meal containing 75 g of glucose in healthy men.	The single combined ingestion of GTC and CCA significantly altered the incretin response and suppressed glucose and insulin levels.	These findings suggest that the effective minimum dose is 540 mg of GTC and 150 mg of CCA	High
