# Supplementary material for: A Narrative Review on Strategies for the Reversion of Prediabetes to Normoglycemia: Food Pyramid, Physical Activity, and Self-Monitoring Innovative Glucose Devices
Source: Nutrients. 2023 Nov 28;15(23):4943. doi: 10.3390/nu15234943 (PMC10707766; doi:10.3390/nu15234943)
Supplement: Supplementary file 1 [file nutrients-15-04943-s001.zip › Tables Prediabetes Nutrients/13 Tabelle Physical activity.pdf]

Authors	Type of study	Number of studies	Subjects	End point	Results	Conclusion
Snowling NJ et al, 2006	Meta-analysis	27 eligible studies	1003 type 2 diabetic patients (age 55 +/- 7 years) over 5-104 weeks	Meta-analyze the effects of different modes of exercise training on measures of glucose control and other risk factors for complications of diabetes.	Differences among the effects of aerobic, resistance, and combined training on HbA(1c) (A1C) were trivial; for training lasting >/=12 weeks, the overall effect was a small beneficial reduction (A1C 0.8 +/- 0.3% [mean +/- 90% confidence limit]). There were generally small to moderate benefits for other measures of glucose control. Effects of covariates were generally trivial or unclear, but there were small additional benefits of exercise on glucose control with increased disease severity.	All forms of exercise training produce small benefits in the main measure of glucose control: A1C. The effects are similar to those of dietary, drug, and insulin treatments. The clinical importance of combining these treatments needs further research.
De Nardi AT et al, 2018	Meta-analysis	818 eligible studies	7 studies were included in systematic review (64 prediabetes and 120 T2D patients) and 5 with T2D were meta-analyzed.	To compare the effects of high-intensity interval training (HIIT) versus moderate-intensity continuous training (MICT) on functional capacity and cardiometabolic markers	No differences were found between two modalities of exercises considering the outcomes HbA1c, systolic and diastolic blood pressure, total cholesterol, HDL and LDL cholesterol, triglycerides, BMI, and waist-to-hip ratio. Most of the studies presented unclear risk of bias, and low and very low quality of evidence.	HIIT induces cardiometabolic adaptations similar to those of MICT in prediabetes and T2D, and provides greater benefits to functional capacity in patients with T2D.
Jadhav RA et al, 2021	Systematic review and meta-analysis of randomized controlled trials	1688 citations, 31 full-text articles assessed for eligibility of inclusion. 9 studies satisfied the pre-specified criteria for inclusion.	1906 participants with the age group ranging from 20 to 70 years. All participants were diagnosed by either WHO or ADA.	Strengthen the evidence on the impact of physical activity promotion on inflammatory markers in prediabetes considering that the inflammatory stage in prediabetes is associated with increased levels of adipokines and proinflammatory cytokines.	Meta-analysis found that physical activity with or without dietary or lifestyle modification reduces level of leptin (MD-2.11 ng/mL, 95% CI -3.81 - -0.42) and interleukin-6 (MD -0.15 pg/mL, 95% CI -0.25--0.04). It has no effect on level of adiponectin (MD 0.26 µg/mL, 95% CI -0.42- 0.93), C-reactive protein (MD -0.05 mg/L, 95% CI -0.33-0.23) and tumour necrosis factor-α (MD 0.67 pg/mL, 95% CI -2.56-3.89).	Physical activity promotion with dietary and lifestyle modification may reduce the level of leptin and interleukin-6 but are uncertain if there is any effect on levels of adiponectin, C-reactive protein and tumour necrosis factor-α in the individuals with prediabetes.

.

Table 1. Physical activity and prediabetes – RCT.

Authors	Type of study	Population Characteristic	Type of intervention	Duration	End point	Result	Conclusion
Jung ME et al, 2015	RCT	32 inactive individuals with prediabetes between the ages of 30 and 60 years	15 individuals received HIIT and 17 individuals received MICT 1-month follow-up testing	After completing 10 sessions of supervised training participants were asked to perform HIIT or MICT three times per week for four weeks.	To compare self-report and objective measures of physical activity after one month of independent exercise in individuals with prediabetes who were randomized to HIIT (n = 15) or traditional moderate-intensity continuous training (MICT, n = 17).	Individuals in HIIT (89 ± 11%) adhered to their prescribed protocol to a greater extent than individuals in MICT (71 ± 31%). Minutes spent in vigorous physical activity per week measured by accelerometer were higher in HIIT (24 ± 18) as compared to MICT (11 ± 10) at one-month follow-up ($P = 0.049$, Cohen's $d = 0.92$). Cardiorespiratory fitness and systolic blood pressure assessed at one-month follow-up were equally improved (P's < 0.05).	This study provides preliminary evidence that individuals with prediabetes can adhere to HIIT over the short-term and do so at a level that is greater than MICT.
