# Supplementary material for: A Narrative Review on Strategies for the Reversion of Prediabetes to Normoglycemia: Food Pyramid, Physical Activity, and Self-Monitoring Innovative Glucose Devices
Source: Nutrients. 2023 Nov 28;15(23):4943. doi: 10.3390/nu15234943 (PMC10707766; doi:10.3390/nu15234943)
Supplement: Supplementary file 1 [file nutrients-15-04943-s001.zip › Tables Prediabetes Nutrients/14 tabelle Microbiota.pdf]

Authors	Type of study	Populationcharacteristics	Type of intervention	Duration	End point	Results	Conclusion	Strength of evidence
Qin, J et al, 2012	Case-Control study	123 patients with Metabolic syndrome (MetS) and 304 controls	Whole-genome shotgun sequencing was used and approximately 60,000 type-2-diabetes-associated markers was identified		To determine whether the gut microbiome plays a role in MetS development and progression	Gut microbiomes of MetS patients possess significantly lower gut microbiome diversity. Microbiome changes in MetS patients may aggravate inflammation and contribute to MetS diseases by inhibiting the production of short-chain fatty acids (SCFAs).	Potential utility of beneficial gut microbiota as a potential therapeutic to alleviate MetS.	Moderate
Larsen, N et al, 2010	Case-Control study	36 male adults with different ages and body-mass indices (BMIs), including 18 subjects with diabetes type 2 (DT2)	The fecal bacterial composition was investigated by real-time quantitative PCR (qPCR) and in a subgroup of subjects (N = 20) by tag-encoded amplicon pyrosequencing of the V4 region of the 16S rRNA gene.		To assess the differences between the composition of the intestinal microbiota in humans with type 2 diabetes and non-diabetic persons	The proportions of phylum Firmicutes and class Clostridia were significantly reduced in diabetic group compared to control group. Bacteroidetes/Firmicutes ratios as well as Bacteroides/Prevotella ratios correlated positively and significantly with plasma glucose concentration but not with BMIs. Class Betaproteobacteria was highly enriched in diabetic compared to non-diabetic persons (P = 0.02)	DT2 in humans is associated with compositional changes in intestinal microbiota.	Moderate
Tang, W.H.W et al, 2017	Prospective cohort study	2 cohorts: the first cohort comprehends 1216 patients with T2DM, second cohort comprehends 300 apparently healthy individuals ≥21 years old	Exame of relationship between fasting TMAO and 2 of its nutrient precursors, choline and betaine, vs 3-year major adverse cardiac events and 5-year mortality	5-y period	To study prognostic value of TMAO concentrations are increased in T2DM patients and their relation to glycemic control.	TMAO and choline concentrations were higher in individuals with T2DM vs healthy controls. Within T2DM patients, higher plasma TMAO was associated with a significant 3.0-fold increased 3-year major adverse cardiac event risk and a 3.6-fold increased 5-year mortality risk.	Fasting plasma concentrations of the proatherogenic gut microbe-generated metabolite TMAO are higher in T2DM patients and are independent risk factor for major adverse cardiac events and mortality risks.	Moderate
Li, Y. et al, 2015	Prospective cohort study	203,308 men and Womenextracted from three cohorts: the Nurses' Health Study (NHS), NHS II, and the Health Professionals	Dietary phosphatidylcholine was estimated by afood-frequency questionnaire	4 years	To study the association between dietary phosphatidylcholine and risk of type 2 diabetes (T2D).	7,063, 4,465, and 3,531 cases of T2D (during NHS, NHS II and HPFS, respectively) were documented. Compared with people in the lowest quintiles of dietary phosphatidylcholine intakes, the RR	Dietary intakes of phosphatidylcholine is associated with incident T2D risk.	Moderate

		Follow-Up Study (HPFS).				of T2D for those in the highest quintiles was 1.36 in NHS, 1.35 in NHS II, 1.28 in HPFS, and 1.34 in the pooled analysis. The association was 1.24 after further adjustment for the three major food sources (red meat, eggs, and seafoods) and 1.27 with all choline-containing components and betaine mutually adjusted. With an increase of 100 mg choline from phosphatidylcholine, the risk of T2D increased by 17%.		
Roy, S et al, 2020	Longitudinal cohort study	300 diabetes-free men and women (77%) aged 20–55 years (mean = 34±10)	Multivariable generalized linear models regressed; i) FPG change (year 2 minus baseline) on baseline TMAO tertiles; and ii) HOMA-IR and HbA1c on TMAO tertiles. Multivariable relative risk regressions modeled prevalent prediabetes across TMAO tertiles.	2 years	To investigate the association between TMAO and biomarkers of diabetes risk.	Mean values of 2-year longitudinal FPG±SE across tertiles of TMAO were 86.6±0.9, 86.7±0.9, 86.4±0.9 (p = 0.98). Trends were null for FPG, HbA1c, HOMA-IR, cross-sectionally. The prevalence ratio of prediabetes among participants in 2nd and 3rd TMAO tertiles (vs. the 1st) were 1.94 [95%CI 1.09–3.48] and 1.41 [95%CI: 0.76–2.61].	TMAO levels are associated with increased prevalence of prediabetes in a nonlinear fashion but not with insulin resistance or longitudinal FPG change.	Moderate
Allin, K.H et al, 2018	Case-Control study	134 Danish adults with prediabetes, overweight, insulin resistance, dyslipidaemia and low-grade inflammation and 134 individuals with normal glucose regulation.	Comparison of biochemical markers, anthropometric parameters and gut microbiota in control and case groups.	12 week	To test whether specific gut microbiota profiles are associated with prediabetes	Five bacterial genera and 36 operational taxonomic units (OTUs) were differentially abundant between individuals with prediabetes and those with normal glucose regulation. At the genus level, the abundance of Clostridium was decreased, whereas the	Individuals with prediabetes have intestinal microbiota characterised by a decreased of the genus Clostridium and the mucin-degrading bacterium A.	Moderate

						abundances of Dorea, Sutterella and Streptococcus were increased. The two OTUs that differed the most were a member of the order Clostridiales and Akkermansia muciniphila, which both displayed lower abundance among individuals with prediabetes.	muciniphila. These findings are comparable to observations in overt chronic diseases characterised by low-grade inflammation.	
Zhong, H et al, 2019	Cohort study	77 type 2 diabetic individualstreatment-naïve, 80 pre-diabetic individuals and 97 normal glucose tolerant individuals.	Combination of in-depth metagenomics and metaproteomics analyseson faecal samples		To investigate compositional and functional changes of the gut microbiota and the faecal content of microbial and host proteins in Pre-DM and treatment-naïve T2D individuals	Distinct differences characterizing the gut microbiota of the three groups were observed. The content of several human antimicrobial peptides and pancreatic enzymes differed in faecal samples between three groups.	There is a a complex, disease stage-dependent interplay between the gut microbiota and the host.	Moderate

Review and meta-analysis

Authors	Type of study	Number of studies	Subjects (total)	End point	Result	Conclusion	Strenght of evidence
Letchumanan, G et al, 2022	Systematic Review of Observational Studies	18 eligible studies	5489 participants	To summarise the existing evidence related to microbiota composition and diversity in individuals with prediabetes (preDM) and individuals newly diagnosed with T2DM (newDM) in comparison to individuals with normal glucose tolerance (nonDM).	Four out of the 18 studies found increased abundance of phylum Firmicutes along with decreased abundance of Bacteroidetes in newDM. At the genus/species levels, decreased abundance of Faecalibacterium prausnitzii, Roseburia, Dialister, Flavonifractor, Alistipes, Haemophilus and Akkermansia muciniphila and increased abundance of Lactobacillus, Streptococcus, Escherichia, Veillonella and Collinsella were observed in the disease groups in at least two studies. Lactobacillus was also found to positively correlate with fasting plasma glucose (FPG), HbA1c and/or homeostatic assessment of insulin resistance (HOMA-IR) in four studies.	There is a need for further investigations on the species/strain-specific role of endogenously present Lactobacillus in glucose regulation mechanism and T2DM disease progression and more studies are needed to establish more consistent associations, between clinical biomarkers or dietary intake and specific gut bacterial composition in prediabetes and early T2DM.	High
Gurung, M et al, 2020	Systematic Review	42 human	not available	To study the potential role of different	The genera of Bifidobacterium, Bacteroides, Faecalibacterium, Akkermansia and Roseburia were	Some microbial taxa and related molecular mechanisms may be	High

		studies		bacterial taxa affecting diabetes and to discuss potential molecular mechanisms of microbiota effects in the onset and progression of T2D.	negatively associated with T2D, while the genera of Ruminococcus, Fusobacterium, and Blautia were positively associated with T2D.	involved in glucose metabolism related to T2D. However, the heterogeneity of T2D and redundancy of gut microbiota do not promises impleinter pretations (e.g.lowdiversity) and easy solutions (such as fecal transplant from non-diabetic/non-obese donor).	
Barengolts, E, 2016	Review of Randomized Controlled Trials.	not available	not available	To review the data from randomized controlled trials (RCTs) for the roles of microbiota, pre-, pro- and synbiotics in metabolic conditions (obesity, prediabetes, and diabetes mellitus type 2 [DM2]).	Results of RCTs of prebiotics suggested a neutral effect on body weight, decreased fasting and postprandial glucose, and improved insulin sensitivity and lipid profile. Some inflammation markers were reduced, sometimes substantially (20-30%). The effect was seen mostly with fermented milk or yogurt compared to capsule form, consumption for at least 8 weeks, and use of multiple rather than a single bacterial strain. Changes in microbiota were seen at times with both pre- and probiotics. Pickled and fermented foods, particularly vegetables and beans, could serve as a dietary source of pre-, pro-, and synbiotics.	Pre-, pro-, and synbiotics could prove useful, but further research is needed to clarify their clinical relevance for the prevention and management of metabolic disease.	High
Wang, X et al, 2021	Systematic Review of Randomized Controlled Trials.	8 Randomized Controlled Trials.	391 Participants	To identify evidence for microbiota's role and use of probiotics, pre-biotics, or synbiotics in pre-diabetes.	. Probiotics can decrease glycated hemoglobin (HbA1c) and have the potential to improve post-load glucose levels. Pre-biotics are failed to show an evident improvement in glycemic control, but their use caused the changes in the composition of gut microbiota. A combination of probiotics and pre-biotics in the synbiotics supplementation is more effective than probiotics alone in glycemic control.	Using probiotics, pre-biotics or synbiotics for the treatment of pre-diabetes, the benefits of modulating the abundance of gut microbiota were partially demonstrated. However, there is insufficient evidence to show significant benefits on glucose metabolism.	High
Ya Liet al, 2022	Systematic review and Meta-analysis	7 publications	460 patients	To examine the effects of probiotics on eight factors in the prediabetic population.	Probiotics were able to significantly decrease the levels ofglycated haemoglobin A1c (HbA1c), quantitative insulin sensitivity check index (QUICKI), total cholesterol (TC), triglyceride (TG)and low-density lipoprotein cholesterol (LDL-C)compared to levels in the placebo group.	Probiotics may fulfil an important role in the regulation of HbA1c, QUICKI, TC, TG and LDL-C in patients with prediabetes. In addition, probiotics may regulate blood glucose homeostasis in a variety of ways.	High
Zeighamy Alamdary, S et al, 2022	Systematic Review of Randomized	15 articles	1295 patients	To compile the results of clinical trials investigating the	Different probiotics compositions have shown beneficial and noticeable effects on glucose homeostasis, lipid profiles, BMI, and inflammatory	Administration of probiotics may provide beneficial and healthful effects in the clinical management	High

	Controlled Trials.			effects of pro-/pre-/synbiotics on prediabetes subjects from 2010 to 2020.	markers in subjects with prediabetes, metabolic syndrome, and healthy individuals.	of patients with prediabetes and metabolic syndrome and could be advantageous in recomposing the gut microbiota back into the normal state during the prediabetic state.	
Bock, P.M et al, 2021	Systematic review and Meta-analysisof randomised controlled clinical trials	130 articles for review, 38 of which included in the meta-analysis	2086 participants	To assess the effect of probiotic, prebiotic or synbiotic supplementation on gut microbiota and glucose control and lipid levels in individuals with diabetes.	The use of prebiotics, probiotics or synbiotics reduced HbA1c levels, but did not reach the threshold for significance and had no effect on LDL-cholesterol levels. However, their consumption decreased levels of fasting blood glucose, total cholesterol, triacylglycerols and insulinaemia and increased HDL-cholesterol levels.	In individuals with diabetes mellitus, supplementation with probiotics, prebiotics or synbiotics improved metabolic variables, although the magnitude of this effect is low.	High
Zhang, Q.et al, 2015	Meta-analysis of randomized controlled trials	7 trials	386 participants	To investigate the effects of probiotics on glucose metabolism in patients with type 2 diabetes mellitus.	Probiotic consumption significantly changed FPG by -15.92mg/dL and HbA1cby -0.54% compared with control groups. Subgroup analysis was conducted to trials with non-yogurts control. Meta-analysis of trials with multiple species of probiotics found a significant reduction in FPG The duration of intervention for ≥8 weeks resulted in a significant reduction in FPG. The results also showed that probiotic therapy significantly decreased homeostasis model assessment of insulin resistance (HOMA-IR) and insulin concentration.	Consuming probiotics may improve glucose metabolism by a modest degree, with a potentially greater effect when the duration of intervention is ≥8 weeks, or multiple species of probiotics are consumed.	High
