# Supplementary material for: A Narrative Review on Strategies for the Reversion of Prediabetes to Normoglycemia: Food Pyramid, Physical Activity, and Self-Monitoring Innovative Glucose Devices
Source: Nutrients. 2023 Nov 28;15(23):4943. doi: 10.3390/nu15234943 (PMC10707766; doi:10.3390/nu15234943)
Supplement: Supplementary file 1 [file nutrients-15-04943-s001.zip › Tables Prediabetes Nutrients/15 Tabelle Monitoring Glucose Levels.pdf]

Authors	Type of study	Population characteristics	Type of intervention/exposure	Duration	End point	Results	Conclusion	Strength of evidence
Schaffzin J.K. et al	Retrospective cohort study	Any resident with diabetes living in the assisted living facilities between August 2006 and January 2007 (34 residents with diabetes and 12 epidemiologically linked residents). A case was defined as any member of the cohort with serologic evidence of current or previous HBV infection. Diabetes was identified based on notation in the medical record.	/	/	HBV transmission in patient during assisted monitoring of blood glucose (AMBG) and implemented preventive measures.	Serologic testing detected 6 residents with diabetes with current HBVI and 4 residents with diabetes and 1 epidemiologically linked resident with previous HBVI.	AMBG was significantly associated with HBVI in ALF residents with diabetes.	Medium
Han B. et al	Cross-sectional comparative study	408 patients with diabetes and 408 people without diabetes randomly matched 1:1	Venous blood was collected for HBV serological testing and blood glucose testing	/	Comparison of hepatitis B surface antigen (HBsAg) positive rates between the two groups	HBsAg positive rate in people without diabetes was 2.0% and in those with diabetes was 4.2%. Whether in people without diabetes or patients with diabetes, higher frequency of SMBG was associated with higher HBsAg positive rate. Increases in the duration of diabetes were correlated with increasing rates of HBsAg.	Routine blood glucose monitoring at home was associated with HBV infection, which meant people with diabetes may be at high risk of HBV infection	Medium

Pierre K.B. et al	Randomized controlled trial	1548 patients receiving mechanical ventilation admitted to the surgical intensive care	A total of 1548 critically ill surgical intensive care unit patients were randomly assigned to receive either intensive insulin therapy (BG 80–110 mg/dl) or conventional treatment (initiation of insulin infusion for BG greater than 215 mg/dl and maintenance of BG between 180 and 200 mg/dl).	February 2, 2000 and January 18, 2001	To determine whether intensive insulin therapy to normalize blood glucose levels (BG) reduces mortality and morbidity among critically ill patients.	In the intensive insulin therapy group, BG was maintained between 80 and 110 mg/dl. Overall, 98.7% of the intensive treatment group required insulin based on this algorithm. Of the conventional treatment group, 39.2% of patients received insulin therapy with maintenance of BG at a mean of 173 ± 33 mg/dl.	Maintenance of normoglycemia (80–110 mg/dl) with intensive insulin therapy reduces both mortality and morbidity associated with critical illness.	High
Finney S.J. et al	Single-center, prospective, observational study	531 patients (median age, 64 years)	/	Median lengths of stay were 1.8 (interquartile range, 0.9-3.7) days and 6 (interquartile range, 4.5-8.3) days, respectively.	Intensive care unit (ICU) mortality.	Of 531 patients admitted to the ICU, 523 underwent analysis of their glycemic control. Twenty-four–hour control of blood glucose levels was variable. Rates of ICU and hospital mortality were 5.2% and 5.7%, respectively.	Increased insulin administration is positively associated with death in the ICU regardless of the prevailing blood glucose level. Thus, control of glucose levels rather than of absolute levels of exogenous insulin appear to account for the mortality benefit associated with intensive insulin therapy demonstrated by others.	Medium

Evans J.M. et al,	Retrospective study	Patients resident in Tayside in 1993-5 who were using insulin and were registered on the database and diagnosed with insulin dependent (type 1) or non-insulin dependent (type 2) diabetes before 1993.			Investigate patterns of self monitoring of blood glucose concentration in diabetic patients who use insulin and to determine whether frequency of self monitoring is related to glycaemic control.	Among 807 patients with type 1 diabetes, 128 (16%) did not redeem any prescriptions for glucose monitoring reagent strips in the 3 year study period. Only 161 (20%) redeemed prescriptions for enough reagent strips to test glucose daily. The corresponding figures for the 790 patients with type 2 diabetes who used insulin were 162 (21%; no strips) and 131 (17%; daily tests)	Self monitoring of blood glucose concentration is associated with improved glycaemic control in patients with type 1 diabetes. Regular self monitoring in patients with type 1 and type 2 diabetes is uncommon	Medium
Van den Berghe G. et al,	Prospective Randomized controlled trial	1,548 patients	Subjects randomly assigned to either strict normalization of blood glucose (80-110 mg/dL) with insulin infusion or the conventional approach, in which insulin is only given to maintain blood glucose levels at 180-200 mg/dL	12 days	Report the factors determining insulin requirements and the impact of insulin dose vs. blood glucose control on the observed outcome benefits	The lowered blood glucose level rather than the insulin dose was related to reduced mortality (p <.0001), critical illness polyneuropathy (p <.0001), bacteremia (p =.02), and inflammation (p =.0006) but not to prevention of acute renal failure.	Normoglycemia was safely reached within 24 hrs and maintained during intensive care by using insulin titration guidelines.	High

Malanda U.L. et al, 2012	Systematic review	Twelve randomised controlled trials were included and evaluated outcomes in 3259 randomised patients.	Nine trials compared SMBG with usual care without monitoring, one study compared SMBG with SMUG, one study was a three-armed trial comparing SMBG and SMUG with usual care and one study was a three-armed trial comparing less intensive SMBG and more intensive SMBG with a control group. Seven out of 11 studies had a low risk of bias for most indicators.	Intervention duration ranged from6 months (26 weeks) to 12 months (52 weeks).	To assess the effects of SMBG in patients with type 2 diabetes mellitus who are not using insulin.	/	When diabetes duration is over one year, the overall effect of self-monitoring of blood glucose on glycaemic control in patients with type 2 diabetes who are not using insulin is small up to six months after initiation and subsides after 12 months.	High

Sørgård, B. et al,	Cross-sectional study	23 adults with type 1 diabetes, including current and former users of continuous glucose monitoring from four different outpatient clinics	/	/	To describe positively and negatively perceived situations experienced by adults with type 1 diabetes using continuous glucose monitoring and the actions they take to deal with these situations	The participants described that they felt that the use of continuous glucose monitoring was a balance between benefits and barriers, and how, through their actions, they tried to adapt their use of continuous glucose monitoring to fit their lifestyles.	Continuous glucose monitoring is perceived as an effective and important tool in the self-management of diabetes type 1. It enables a better everyday life and increased satisfaction with treatment.	Medium
Hilliard M.E. et al,	Cross-sectional study	Transcripts from semistructured qualitative interviews with 55 parents of children aged 1 to <8 years, with T1D duration ≥6 months, and whose child currently or previously used Continuous glucose monitoring (CGM).	/	/		Parents described benefits of CGM use: decreased worry about glucose excursions, improved sleep, increased sense of safety with children who cannot recognize or express symptoms of hypo- or hyperglycemia, and greater comfort with other caregivers.	CGM may address unique challenges of T1D in young children and increase parental comfort with diabetes management, yet there are multiple barriers to initiating or maintaining CGM use.	Medium
Engler R. et al,	Cross-sectional study	People with diabetes and the parents of children with diabetes (n = 1,348)	Results from two surveys regarding continuous glucose monitoring (CGM) devices	/	The importance of the concept of “user burden” in patients’ and caregivers’ evaluations of the acceptability of available devices	/	Minimizing system obtrusiveness will likely be of significant value in reducing hurdles to CGM device use and adherence	Medium

Diabetes Care	Position statement						SMBG is recommended for all patients who use insulin. Recommendations for testing urine for glucose and ketones as part of diabetes management are described here.	Low
**Authors**	**Type of study**	**Population characteristics**	**Type of intervention/exposure**	**Duration**	**End point**	**Results**	**Conclusion**	**Strength of evidence**
Hayford J.T. et al, 1983	Cross-sectional study	24 diabetic subjects (15 female and 9 male), all subject had type I diabetes, with the exception of one subject (insulin-treated type II diabetic patient)	Subjects were maintained on their previous insulin regimens with no attempt to optimize blood glucose control.	/	Correlation between mean plasma glucose concentration with simultaneous urine glucose concentration on excretion rate.	Both the urine glucose concentration and urine glucose excretion rate are significantly correlated (P<0.0001).	Observations on the correlation between mean plasma glucose concentration with simultaneous urine glucose concentration or excretion rate re-emphasize the limitations of this approach.	Medium

Morris L.R., 1981	Cross-sectional study	246 adult diabetics.	Were compared reported levels from 400 second-voided urines to simultaneous plasma determinations	/	Determine whether semiquantitative glucose measurements of spot urine specimens accurately reflect prevailing plasma glucose levels	Quantitative urine levels and plasma glucose levels correlated. However, when semiquantitative urinary determinations were compared to plasma glucose stratified into 0 to 149, 150 to 199, and greater than 200 mg/dL, 75% of the urine samples associated with plasma levels from 150 to 199 mg/dL were negative by Diastix, and 16.5% of samples negative by Diastix were in the 200+ mg/dL plasma range.	Except for detection of marked hyperglycemia, spot urine glucose determinations are inadequate as the sole means of clinical assessment for management of diabetic patients.	Medium
Zhang Z., et al, 2019	Experimental study	12 adults (10 completed)	The photos of the patches were used for the determination of sweat loss and pH level. The sweat patches were then placed in Petri dishes and air-dried before a second photograph was taken to capture the color intensity on the detection zones.	/	They introduce a versatile, cost-effective, flexible, and wearable POC biomarker patch for effective sweat collection and health monitoring.	It was observed glucose concentrations between 4.7 and 98.4 μM for eight of the subjects, which is in the normal range of sweat glucose (<120 μM).54 Subject 2 and 10 have a relatively high sweat glucose concentration of 163.5 μM and 336.1 μM, respectively. The high glucose readout in subject 10, corrected by	The device can detect sweat loss, pH, glucose concentrations and lactate concentrations in sweat, with the ability to detect glucose levels in the physiological range of 50–300 μM.	Low

						the sweat volume, could potentially indicate a prediabetic or diabetic condition for the subject.		
Ilea A. et al, 2019	Systemati review	49 papers selected	/	/	to present the development in the biosensors research and their utility using salivary assessment.	The 49 papers selected for the present review focused on assessing the salivary biomarkers used in general diseases, oral pathologies, and pharmacology. The biosensors proved to be reliable tools for measuring the salivary levels of biochemical metabolic compounds such as glucose, proteinases and proteins, heavy metals and various chemical compounds, microorganisms, oncology markers, drugs, and neurotransmitters.	Saliva is a biofluid with a significant clinical applicability for the evaluation and monitoring of a patient's general health. Biosensors designed for assessing a wide range of salivary biomarkers are emerging as promising diagnostic or screening tools for improving the patients' quality of life	High

Cui Y. et al, 2021	Randomized controlled trial	A total amount of six saliva collection methods were employed in 80 healthy participants in the morning	/	/	To identify the ideal saliva collection method	he improved method obtained absorbance at the wavelength of 520 nm, and the optimized parameter combination was pH 6.5 and 5 mg/dL NaCl. The lower limit of glucose detection was 0.1 mg/dL. Unstimulated saliva glucose concentration was higher than stimulated saliva glucose concentration. Unstimulated parotid saliva glucose concentration was the highest. Besides, unstimulated saliva glucose has a better normal distribution effect. Meantime, it was found that unstimulated parotid saliva was the most highly correlated with blood glucose (R2 = 0.707).	The saliva collection method was an important factor that affected saliva glucose concentration. Unstimulated parotid saliva was the most highly correlated with blood glucose, which provided a reference for prediction of diabetes mellitus	High

Cui Y. et al, 2022	Randomized controlled trial	40 age-matched DM patients and 40 healthy controls	The correlations between salivary glucose and blood glucose before and after breakfast were analyzed.	/	This study aims to identify an ideal saliva collection method and to use this method to determine the population and individual correlations between salivary glucose and blood glucose levels in DM patients and healthy controls	Compared with unstimulated saliva, stimulated saliva had decreased glucose level and increased salivary flow. In addition, unstimulated parotid salivary glucose was most correlated with blood glucose level (R2 = 0.9153), and the ROC curve area was 0.9316, which could accurately distinguish DM patients.	The unstimulated parotid salivary glucose before breakfast presents an ideal saliva collecting method with which to replace blood-glucose use to detect DM, which provides a reference for the prediction of DM	High
