# Supplementary material for: A Narrative Review on Strategies for the Reversion of Prediabetes to Normoglycemia: Food Pyramid, Physical Activity, and Self-Monitoring Innovative Glucose Devices
Source: Nutrients. 2023 Nov 28;15(23):4943. doi: 10.3390/nu15234943 (PMC10707766; doi:10.3390/nu15234943)
Supplement: Supplementary file 1 [file nutrients-15-04943-s001.zip › Tables Prediabetes Nutrients/2a Tabelle Milk and Dairy.pdf]

Authors	Type of study	Population characteristics	Type of intervention	Duration	End point	Results	Conclusions	Strength of evidence
Hoffman J.R. and Falvo M.J.	Narrative review				to examine and analyze key factors responsible for making appropriate choices on the type of protein to consume in both athletic and general populations.		Animal sources of dietary protein, despite providing a complete protein and numerous vitamins and minerals, have some health professionals concerned about the amount of saturated fat common in these foods compared to vegetable sources.	Low
Séverin Sindayikengera and Wen-shui Xia	Experimental study		Whey protein chemical composition, protein solubility, amino acid composition, essential amino acid index (EAA index), biological value (BV), nutritional index (NI), chemical score, enzymic protein efficiency ratio (E-PER) and in			The results indicated that the enzymatic hydrolysis of WPC 80 and sodium caseinate by Protamex improved the solubility and IVPD of their hydrolysates.	The nutritional qualities of WPC 80, sodium caseinate and their hydrolysates were good and make them appropriate for food formulations or as nutritional supplement	Moderate

			vitro protein digestibility (IVPD) were determined					
Yau W.J. et al, 2020	Systematic review	Ninety-five articles involving a total of 11,211 participants were included in this review	Nutritional strategies were broadly classified into four groups: low calorie diet, low glycemic index diet, specific foods, and a combination of diet and exercise.		review on recently reported nutritional interventions for individuals with prediabetes.	More than 50% of reported interventions resulted in significant improvements in these parameters.	ritional interventions have demonstrated feasibility and practicality as an effective option for prediabetes management. However, the intervention variability demonstrates the challenges of a 'one-size-fits-all' approach.	High
Rideout T.C. et al, 2013	Randomized Controlled Trial	Twenty-three healthy subjects completed a randomized, crossover trial	Randomly assigned to one of two treatment groups: a high dairy supplemented group instructed to consume 4 servings of dairy per day (HD); or a low dairy supplemented group limited to no more than 2 servings of dairy per day (LD).	12 months	examine the influence of long-term (6 month) dairy consumption on metabolic parameters in healthy volunteers	Body weight and composition, energy expenditure, blood pressure, blood glucose, and blood lipid and lipoprotein responses did not differ ($p > 0.05$) between the LD and HD groups. HD consumption improved ($p < 0.05$) plasma insulin (-9%) and insulin	Study results suggest that high dairy consumption (4 servings/d) may improve insulin resistance without negatively impacting bodyweight or lipid status under free-living conditions.	High

						resistance (-11%, p = 0.03) as estimated by HOMA-IR compared with the LD group.		
Slurink I.A. et al, 2022	Prospective cohort study	2262 participants without (pre-) diabetes at enrolment (mean age 56 ± 7.3 years; 50% male).	consumption of total dairy and dairy types	6.4 ± 0.7 years of follow-up	investigate prospective associations of consumption of total dairy and dairy types with incident prediabetes in a Dutch population-based study	810 participants (35.9%) developed prediabetes. High fat fermented dairy, cheese and high fat cheese were associated with a 17% (ptrend = 0.04), 14% (ptrend = 0.04) and 21% (ptrend = 0.01) lower risk of incident prediabetes, respectively, in top compared to bottom quartiles, after adjustment for confounders. Total dairy and other dairy types were not associated with prediabetes risk in adjusted models	The highest intake of high fat fermented dairy, cheese and high fat cheese were associated with a lower risk of prediabetes, whereas other dairy types were not associated. Cheese seems to be inversely associated with type 2 diabetes risk, despite high levels of saturated fatty acids and sodium.	Moderate
Slurink I.A. et al, 2022	Prospective cohort study	6770 participants (aged 62 ± 4 years, 59%		11.3 ± 4.8 years	to examine associations between dairy-type intake with	1139 incident prediabetes cases were documented	a higher intake of high-fat yogurt was associated with	Moderate

		female) free of (pre-)diabetes			prediabetes risk and longitudinal insulin resistance.	(18.8%); a higher intake of high-fat yogurt was associated with lower prediabetes risk; higher intake of high-fat milk was associated with lower prediabetes risk.	a lower prediabetes risk and lower longitudinal insulin resistance. Additionally, high-fat milk was associated with a lower prediabetes risk.	
Hruby A. et al, 2017	Retrospective cohort study	2809 participants [mean ± SD age: 54.0 ± 9.7 y; body mass index (in kg/m2): 27.1 ± 4.7; 54% female].	consumption of milk-based products		To assess associations between consumption of milk-based products, incident prediabetes, and progression to T2D in the Framingham Heart Study Offspring Cohort	902 (48%) developed prediabetes	Associations of dairy with incident prediabetes or diabetes varied both by dairy product and type and by baseline glycemic status in this middle-aged US population.	Moderate
Slurink I.A. et al, 2023	Prospective cohort study	4891 participants with normal glucose tolerance (aged 49.0 ± 12.3 y, 57% female)	consumption of dairy, including different types of dairy products	12 years	To examine the relationship between the consumption of dairy, including different types of dairy products and risk of prediabetes	765 (15.6%) incident cases of prediabetes were observed. The mean intake of dairy foods was 2.4 ± 1.2 servings/d, mostly consisting of low-fat milk (0.70 ± 0.78 servings/d) and high-fat milk	protective associations were found for high-fat dairy types, whereas neutral associations were seen for low-fat dairy types.	Moderate

						(0.47 ± 0.72 servings/d).		
Brouwer-Brolsma E.M. et al, 2018	Cross-sectional study	112 086 adults	a broad variety of dairy subgroups	/	To assess cross-sectional associations of a broad variety of dairy subgroups with pre-diabetes and newly diagnosed type 2 diabetes (ND-T2DM) among Dutch adults.	Median dairy product intake was 324 (interquartile range 227) g/d; 25 549 (23 %) participants had pre-diabetes; and 1305 (1 %) had ND-T2DM. After full adjustment, inverse associations were observed of skimmed dairy (OR100 g 0·98; 95 % CI 0·97, 1·00), fermented dairy (OR100 g 0·98; 95 % CI 0·97, 0·99) and buttermilk (OR150 g 0·97; 95 % CI 0·94, 1·00) with pre-diabetes.	data showed inverse associations of skimmed and fermented dairy products with pre-diabetes. Positive associations were observed for full-fat and non-fermented dairy products with pre-diabetes and ND-T2DM.	Moderate
Pestoni G. et al, 2021	Cross-sectional study	1305 participants of the cross-sectional population-based KORA FF4 study	/	/	to identify dietary patterns and to investigate their association with prediabetes, undetected diabetes and	Participants following the Western pattern had significantly higher chances of having prediabetes (odds ratio [OR] 1.92; 95%	These results suggest a very important role of dietary habits in the prevention of prediabetes and type 2 diabetes	Moderate

					prevalent diabetes	confidence interval [CI] 1.35, 2.73), undetected diabetes (OR 10.12; 95% CI 4.19, 24.43) or prevalent diabetes (OR 3.51; 95% CI 1.85, 6.67), compared to participants following the Prudent pattern.		
Akhavan T. et al, 2010	Randomized Controlled Study	experiment 1: 16 men; experiment 2: 12 men and 10 women	Whey Protein (10–40 g) in 300 mL water was provided in experiment 1, and Whey Protein (5–40 g) and Whey Protein Hydrolysate (10 g) in 300 mL water were provided in experiment 2.		to describe the effect of whey protein or its hydrolysate when consumed before a meal on food intake, pre- and postmeal satiety, and concentrations of blood glucose and insulin in healthy young adults.	In experiment 1, 20–40 g WP suppressed food intake ($P < 0.0001$) and 10–40 g WP reduced postmeal blood glucose concentrations and the area under the curve (AUC) ($P < 0.05$). In experiment 2, 10–40 g WP, but not WPH, reduced postmeal blood glucose AUC and insulin AUC in a dose-dependent manner ($P < 0.05$).	WP consumed before a meal reduces food intake, postmeal blood glucose and insulin, and the ratio of cumulative blood glucose to insulin AUCs in a dose-dependent manner.	High

Hidayat K. Et al, 2019	Narrative review	/	/	/	Providing a clear presentation of the potential implementation of milk proteins as a dietary supplement in the prevention and management of T2DM by summarizing the relevant supporting evidence for this particular topic	The results from these trials showed that milk proteins may have functional properties for stimulating postprandial insulin, resulting in lower postprandial glucose levels.	/	Low
