# Supplementary material for: A Narrative Review on Strategies for the Reversion of Prediabetes to Normoglycemia: Food Pyramid, Physical Activity, and Self-Monitoring Innovative Glucose Devices
Source: Nutrients. 2023 Nov 28;15(23):4943. doi: 10.3390/nu15234943 (PMC10707766; doi:10.3390/nu15234943)
Supplement: Supplementary file 1 [file nutrients-15-04943-s001.zip › Tables Prediabetes Nutrients/2b Tabelle Eggs.pdf]

Authors	Type of study	Population characteristics	Type of intervention	Duration	End point	Results	Conclusion	Strenght of Evidence
Richard, C 2017	Sistematic review	6 original controlled randomized trials	/	/	Evaluated the link between egg consumption and the main cardiovascular risk factors in subjects with type 2 diabetes or at risk of the disease (prediabetes, insulin resistance or metabolic syndrome)	The majority of studies found that egg consumption did not affect major CVD risk factors. Consumption of 6 to 12 eggs per week had no impact on plasma concentrations of total cholesterol, low-density lipoprotein-cholesterol, triglycerides, fasting glucose, insulin or C-reactive protein	Results from randomized controlled trials suggest that consumption of 6 to 12 eggs per week, in the context of a diet that is consistent with guidelines on cardiovascular health promotion, has no adverse effect on major CVD risk factors in individuals at risk for developing diabetes or with type 2 diabetes	High

Drouin-Chartier, J.P., 2020	systematic review and meta-analysis of prospective cohort studies	3 prospective cohort studies	82,750 women from the Nurses' Health Study (NHS; 1980-2012), 89,636 women from NHS II (1991-2017), and 41,412 men from the Health Professionals Follow-up Study (HPFS; 1986-2016) with no diabetes , cardiovascular disease and baseline cancer.		Evaluated the association between egg consumption and T2D risk	Documented 20,514 incident cases of T2D in the NHS, NHS II, and HPFS. In the pooled multivariable model adjusted for updated BMI, lifestyle, and dietary confounders, a 1-egg/d increase was associated with a 14% higher T2D risk. There were, however, significant differences by geographic region (P for interaction = 0.01). Each 1 egg/d was associated with higher T2D risk among US studies (RR: 1.18; 95% CI: 1.10, 1.27; I2 = 51.3%), but not among European (RR: 0.99; 95% CI: 0.85, 1.15; I2 = 73.5%) or Asian (RR: 0.82; 95% CI: 0.62, 1.09; I2 = 59.1%) studies.	Results from the updated meta-analysis show no overall association between moderate egg consumption and risk of T2D.	High

Pourafshar, S 2018	RCT	42 overweight or obese individuals between the ages of 40 and 75 years with pre- and type II-diabetes were included	Participants were randomly assigned to receive either one large egg per day or an equivalent amount of egg substitute for 12 weeks. Blood samples were obtained to analyze lipid profile and biomarkers associated with glycemic control at all time points	12 weeks	Evaluated if egg consumption may improve factors associated with glycemic control and insulin sensitivity	Regular egg consumption resulted in improvements of fasting blood glucose, which was significantly (P = 0.05) reduced by 4.4% at the final visit in the egg group. Participants in the egg group had significantly (P = 0.01) lower levels of homeostatic model assessment of insulin resistance (HOMA-IR) at all visits.	Regular egg consumption has been shown to lead to an improvement in fasting blood sugar and insulin resistance levels (HOMA-IR). There were no significant changes in the levels of total cholesterol and LDL cholesterol.	High
