# Supplementary material for: A Narrative Review on Strategies for the Reversion of Prediabetes to Normoglycemia: Food Pyramid, Physical Activity, and Self-Monitoring Innovative Glucose Devices
Source: Nutrients. 2023 Nov 28;15(23):4943. doi: 10.3390/nu15234943 (PMC10707766; doi:10.3390/nu15234943)
Supplement: Supplementary file 1 [file nutrients-15-04943-s001.zip › Tables Prediabetes Nutrients/2c Tabelle Meat.pdf]

Authors	Type of study	Number of studies	Subjects (total)	End point	Result	Conclusion	Strenght of evidence
A Pan et al, 2011	Meta-analysis combined with 3 cohort studies	3 cohort studies + 1 meta analysis	meta-analysis (442,101 participants and 28,228 diabetes cases)	Evaluated the association between unprocessed and processed red meat consumption and incident T2D in US adults	Documented 13,759 incident T2D cases. Both unprocessed and processed red meat intakes were positively associated with T2D risk in each cohort (all P-trend <0.001). The pooled HRs (95% CIs) for a one serving/d increase in unprocessed, processed, and total red meat consumption were 1.12, 1.32, and 1.14, respectively. The results were confirmed by a meta-analysis: the RRs (95% CIs) were 1.19 and 1.51 for 100 g unprocessed red meat and for 50 g processed red meat, respectively.	Red meat consumption, particularly processed red meat, is associated with an increased risk of T2D	High

A Pan et al, 2011	Meta-analysis combined with 3 cohort studies	3 cohort studies	26,357 men in the Health Professionals Follow-up Study (1986-2006), 48,709 women in the Nurses' Health Study (1986-2006), and 74,077 women in the Nurses' Health Study II (1991-2007).	Evaluate the association between changes in red meat consumption during a 4-year period and subsequent 4-year risk of T2DM in US adults	Documented 7540 incident T2DM cases. Compared with the reference group of no change in red meat intake, increasing red meat intake of more than 0.50 servings per day was associated with a 48% elevated risk. Reducing red meat consumption by more than 0.50 servings per day from baseline to the first 4 years of follow-up was associated with a 14% lower risk	Increasing red meat consumption over time is associated with an elevated subsequent risk of T2DM, and the association is partly mediated by body weight. Limiting red meat consumption over time confers benefits for T2DM prevention.	High
Sanders L.M et al, 2022	Systematic review and meta-analysis of randomized controlled trials	21 RCTs	Adult humans (≥18 y of age)	Evaluating the effects of diets containing red meat compared to diets with lower or no red meat, on markers of glucose homeostasis in adults.	Compared to diets with reduced or no red meat intake, there was no significant impact of red meat intake on insulin sensitivity, insulin resistance, fasting glucose, fasting insulin, glycated hemoglobin, pancreatic beta-cell function, or glucagon-like peptide-1. Red meat intake modestly reduced postprandial glucose compared to meals with reduced or no red meat intake.	The results suggest red meat intake does not impact most glycemic and insulinemic risk factors for T2D. Further investigations are needed on other markers of glucose homeostasis.	High

Nguyen et al, 2022	Cross-sectional study	3000 subjects,, aged 40-60 years, living in rural communes in Khánh Hòa Province for at least 6 months	Anthropometry results, blood samples for biochemical measurements and questionnaire information via face-to-face interviews were collected.	2-4 years	Examine the association between daily consumption of red/processed meat (0-99 g, 100-199 g or ≥ 200 g) and preDM/DM	The relative-risk ratios for DM were 1·00 (reference), 1·11 (95 % CI = 0·75, 1·62) and 1·80 (95 % CI = 1·40, 2·32) from the lowest to the highest red/processed meat consumption categories (*P*trend = 0·006). The corresponding values for preDM were 1·00 (reference), 1·25 (95 % CI = 1·01, 1·54) and 1·67 (95 % CI = 1·20, 2·33) (*P*trend = 0·004).	Increased red/processed meat consumption, but not poultry consumption, was positively associated with the prevalence of preDM/DM in rural communes in Khánh Hòa Province, Vietnam.

Song et al, 2004	Prospective cohort study	37,309 participants in the Women's Health Study aged >/=45 years who were free of cardiovascular disease, cancer, and type 2 diabetes	Validated semiquantitative food frequency questionnaire.	8.8 years	To prospectively assess the relation between red meat intake and incidence of type 2 diabetes.	It was documented 1,558 incident cases of type 2 diabetes and it was found positive associations between intakes of red meat and processed meat and risk of type 2 diabetes. Comparing women in the highest quintile with those in the lowest quintile, the multivariate-adjusted relative risks (RRs) of type 2 diabetes were 1.28 for red meat (95% CI 1.07-1.53, P < 0.001 for trend) and 1.23 for processed meat intake (1.05-1.45, P = 0.001 for trend).	Higher consumption of total red meat, especially various processed meats, may increase risk of developing type 2 diabetes in women.
