# Supplementary material for: A Narrative Review on Strategies for the Reversion of Prediabetes to Normoglycemia: Food Pyramid, Physical Activity, and Self-Monitoring Innovative Glucose Devices
Source: Nutrients. 2023 Nov 28;15(23):4943. doi: 10.3390/nu15234943 (PMC10707766; doi:10.3390/nu15234943)
Supplement: Supplementary file 1 [file nutrients-15-04943-s001.zip › Tables Prediabetes Nutrients/2d Tabelle Plant-Based Proteins.pdf]

Authors	Type of study	Population characteristics	Type of intervention	Duration	End point	Results	Conclusion	Strength of evidence
Becerra-Tomás N et al, 2017	Prospective cohort study	3349 participants without type 2 diabetes at baseline. Aged 55-80 years.	-	4.3 years	Examine the associations between consumption of total legumes and specific subtypes, and type 2 diabetes risk.	266 new cases of type 2 diabetes. Individuals in the highest quartile of total legume and lentil consumption had a lower risk of diabetes than those in the lowest quartile (HR: 0.65; 95% CI: 0.43, 0.96; P-trend = 0.04; and HR: 0.67; 95% CI: 0.46-0.98; P-trend = 0.05, respectively)	A frequent consumption of legumes, particularly lentils, may provide benefits on type 2 diabetes prevention in older adults	Moderate
Shams H. et al, 2008	Case control study	30 patients with type 2 diabetes mellitus	2 groups.<br>Group A normal diet<br>Group B normal diet with 50g cooked lentil and 6g canula oil (substitute of 30g bread and 20g cheese)	6 weeks	Assess the effects of cooked lentil on serum blood glucose and lipid profile among type 2 diabetic patients	Total cholesterol and fasting blood glucose decreased significantly in regimen containing lentils ($P<0.05$)	Consumption of cooked lentil in breakfast led to reduction of FBS and TC and improvement of glycemic control in type 2 diabetic patients	Moderate
Kwak JH et al, 2010	RCT	42 prediabetic or diabetic subjects	Subjects with prediabetes and type 2 DM .<br>Black soy peptide intervention group or placebo control group	12 week	Effect of black soy peptide supplementation on glucose control in subjects with prediabetes and newly diagnosed type 2 diabetes mellitus	Subjects with fasting glucose ≥ 110 mg/dL who consumed black soy peptides tended to have lower fasting glucose levels (two-tailed test, P = .098; one-tailed test, P = .049) and had a significant reduction in 2-hour PG level (two-tailed P = .012, one-tailed P = .006), compared	Black soy peptide supplementation may be beneficial for controlling fasting blood glucose levels and 2-hour PG levels	High

						with baseline levels. The changes in 2-hour PG levels were also statistically significant in the intervention group (-41.25 ± 13.67 mg/dL) compared with the placebo group (12.42 ± 9.80 mg/dL; two-tailed P = .015, one-tailed P = .008)		
Jiang R et al, 2002	Prospective cohort study	83 818 women, aged 34 to 59 years, no history of diabetes, cardiovascular disease or cancer	Subjects completed a validated dietary questionnaire at baseline in 1980, and were followed up for 16 years.	16 years	Examine prospectively the relationship between nut consumption and risk of type 2 diabetes.	3206 new cases of type 2 diabetes were documented. The consumption was inversely associated with risk of type 2 diabetes.	A higher consumption of nut and peanut butter may have some benefits in lowering risk of type 2 diabetes in women	Moderate
Reis CE et al, 2011	RCT	13 subjects (4 men and 9 women), with a mean age of 28.5 ± 10 years	4 types of test meals were consumed: raw peanuts with skin (RPS), roasted peanuts without skin, ground-roasted peanuts without skin (GRPWS) or control meal. The test meals had the same nutrient composition.	24h	Evaluate the effect of peanut processing on glycemic response, and energy and nutrients intake.	The area under the glycemic response curve after GRPWS was lower (p = 0.02) the one obtained for RPS. There was no treatment effect on energy intake, macronutrients and fiber consumption	The consumption of ground-roasted peanuts may favor the control and prevention of diabetes due to its reduction on postprandial glucose response	High

Review and meta-analysis

Authors	Type of study	Number of studies	Subjects (total)	End point	Result	Conclusion	Strenght of evidence
Ley SH et al, 2014	Narrative review	-	-	To examine the role of diet in prevention and management of diabetes in the scientific literature	-	-	Low
Kim Y, Keogh JB, Clifton PM, 2016	Narrative review	-	-	To provide a comprehensive overview of the anti-diabetic effects of commonly consumed dietary polyphenols	In vitro and in vivo studies have shown that dietary polyphenolic compounds improved glucose homeostasis in the intestine, liver, muscle adipocytes and pancreatic β-cells, as well as through prebiotic effects in the digestive tract.	Polyphenols may play a role in diabetes treatment and prevention but further investigations are needed	Low
Schwingshackl L et al, 2018	Meta-analysis	66 randomized trials (86 reports) comparing 10 food groups	3595 participants	To assess the effects of main food groups (refined grains, whole grains, nuts, legumes, fruits and vegetables, eggs, dairy, fish, red meat, and sugar-sweetened beverages) on intermediate-disease markers across randomized intervention trials	Legumes, but also whole grains, nuts, and refined grains, were more effective at reducing HOMA-IR (−1.01 to −0.53) compared with eggs and dairy. No significant effects were detected for HbA1c	Increased intake of legumes (but also nuts and whole grains) is more effective at improving metabolic health than other food groups	High
Schwingshackl L et al, 2017	Meta-analysis	103 studies where included in the meta-analysis	-	Synthesize the knowledge about the relation between intake of 12 major food groups (whole grains, refined grains, vegetables, fruits, nuts, legumes, eggs, dairy, fish, red meat, processed meat, and sugar-sweetened beverages) with risk of all-cause mortality	An inverse association was observed for the highest compared with lowest legume intake categories (RR: 0.96; 95% CI: 0.93, 1.00; I2 = 48%; P-heterogeneity = 0.01). Consumption of legumes was associated with a decreased by 16% risk of all-cause mortality with increasing intake of legumes up to 150 g daily intake	Legumes intake up to 150 g/d may be helpful in reducing mortality for all causes (including diabetes and its complications)	High
Tang J et al, 2020	Meta-analysis	15 cohort studies	565,810 individuals and 32,093 incident cases	Summarize the longitudinal associations between legume and soy intake and risk of type 2 diabetes.	In dose–response analysis, significant linear inverse associations were observed for tofu, soy protein, and soy	Dietary intakes of tofu, soy protein, and soy isoflavones, but not total legumes or total soy, are inversely	High

			(diabetes occursions)		isoflavones (all P < 0.05). Overall quality of evidence was rated as moderate for total legumes and low for total soy and soy subtypes.	associated with incident type 2 diabetes	
Li W et al, 2018	Meta-analysis	Eight studies with 19 reports	335230 participants	Evaluate the relationship between soy intake and type 2 diabetes mellitus risk	A significant inverse association was shown between soy intake and type 2 diabetes mellitus risk with an overall RR of 0.77 (95% CI = 0.66-0.91) with high heterogeneity. Besides, there was an obvious relationship between soy protein and isoflavones intake and risk of T2DM with the summary RR was 0.88 (95% CI = 0.80-0.97) with no heterogeneity.	Soy products and soy constituents (soy protein and soy isoflavones) may be associated with a lower risk of type 2 diabetes mellitus.	High
