# Supplementary material for: A Narrative Review on Strategies for the Reversion of Prediabetes to Normoglycemia: Food Pyramid, Physical Activity, and Self-Monitoring Innovative Glucose Devices
Source: Nutrients. 2023 Nov 28;15(23):4943. doi: 10.3390/nu15234943 (PMC10707766; doi:10.3390/nu15234943)
Supplement: Supplementary file 1 [file nutrients-15-04943-s001.zip › Tables Prediabetes Nutrients/3 Tabelle sarcopenia.pdf]

Authors	Type of study	Population characteristics	Type of intervention	Duration	End point	Results	Conclusion	Strength of evidence
Xu J. et al., 2023	Population study	16,116 U.S. adults aged 20-59 y with dual energy X-ray absorptiometry (DXA) from the National Health and Nutrition Examination Surveys (NHANES)	evaluation of sarcopenia by DXA	//	To explore the specific association between sarcopenia and prediabetes based on large population samples	Sarcopenia was strongly associated with an increased risk of prediabetes after full adjustment (OR = 1.21, 95CI%: 1.05, 1.39, P = 0.009).	Sarcopenia was positively associated with prevalent prediabetes, especially IGT in the non-elderly.	Moderate
Kaga H. et al., 2022	Cross-sectional study	1629 elderly (mean age 73.1 ± 5.4 years) living in Japan	Evaluation of glucose metabolism (75-g oral glucose tolerance test and glycated haemoglobin) and sarcopenia (BIA and hand grip strength)	//	To examine the relationship between sarcopenia and prediabetes	Prediabetes and diabetes are independent risk factors for sarcopenia in men (prediabetes, odds ratio [OR] = 2.081 [95% confidence interval {CI}: 1.031–4.199]; diabetes, OR = 2.614 [95% CI: 1.362–5.018]) and diabetes, but not prediabetes, is an independent risk factor for sarcopenia in women (prediabetes, OR = 1.036 [95% CI: 0.611–1.757]; diabetes, OR = 2.099 [95% CI: 1.146–3.844]).	Although diabetes mellitus is associated with sarcopenia in both sexes, prediabetes is associated with sarcopenia in men, but not in women.	Moderate

Li S. et al., 2023	Observational study	22,482 adults aged ≥ 20 years in the National Health and Nutrition Examination Survey (NHANES)	Sarcopenia defined as ASMBMI (appendicular skeletal muscle mass/body mass index) < 0.789 for males, and <0.512 for females	//	To explore the detailed correlation between pre-diabetes and sarcopenia	Sarcopenia was directly correlated with pre-diabetes [OR (95%CI) = 1.230 (1.057, 1.431), p = 0.008] and T2DM [OR (95%CI) = 2.106 (1.625, 2.729), p < 0.001]. In non-T2DM population, HbA1c was negatively correlated with ASMBMI [β (95%CI) = −0.009 (−0.013, −0.005), p < 0.001].	Pre-diabetes is associated with increased risk of sarcopenia. HbA1c is an independent risk factor for loss of appendicular skeletal muscle mass and sarcopenia when HbA1c greater than 5.2% in the male non-T2DM population.	Moderate

Review and meta-analysis

**Authors**	**Type of study**	**Number of studies**	**Subjects (total)**	**End point**	**Result**	**Conclusion**	**Strenght of evidence**
Qiao Y.S. et al., 2021	Meta-analysis	16 observational studies	//	To investigate the association between the presence of sarcopenia and HbA1c values, prediabetes, diabetes and its complications	Three studies showed that high HbA1c levels lead to loss of muscle mass, and one study showed that people with prediabetes had lower muscle mass, strength, and performance than non-diabetic population. Seven studies showed that people with diabetes had a higher risk of sarcopenia than those without diabetes. The remaining five studies suggested that diabetic complications increased the risk of sarcopenia.	Subjects with prediabetes had reduced mass, strength and muscle performance compared to non-diabetics.	HIGH
