# Supplementary material for: A Narrative Review on Strategies for the Reversion of Prediabetes to Normoglycemia: Food Pyramid, Physical Activity, and Self-Monitoring Innovative Glucose Devices
Source: Nutrients. 2023 Nov 28;15(23):4943. doi: 10.3390/nu15234943 (PMC10707766; doi:10.3390/nu15234943)
Supplement: Supplementary file 1 [file nutrients-15-04943-s001.zip › Tables Prediabetes Nutrients/4 Tabelle Lipid.pdf]

Authors	Type of study	Population characteristics	Type of intervention	Duration	End point	Results	Conclusion	Strength of evidence
Abshirini et al, 2020	Case-Control study	150 subjects with normal fasting glucose and 147 prediabetic subjects, age 35-65.	FFQ with 168 food items.		Determine the association of DFQ (dietary fat quality) and fatty acid intake with prediabetes	A positive association between intake of total SFA, myristic acid, palmitic acid, and prediabetes, and a negative association among n-3 PUFA, eicosapentaenoic, docosahexaenoic and arachidonic acids intake and prediabetes.	Higher intake of dietary n-3 fatty acids was adversely, whereas SFA intake was positively related to pre-diabetes morbidity.	Moderate
Mirmian P et al, 2018	Cohort prospective study	2139 adults, free of T2DM, aged 20-70y-old.	Diet information was collected with the use of a validated questionnaire at baseline.	5.8 years	Examine the association between fatty acid quantity and quality with risk of T2DM in adults	Identified 143 incident T2D cases. When extreme quintiles were compared, cholesterol, monounsaturated fatty acids, polyunsaturated fatty acids and ω-3 fatty acids were associated with T2DM.<br>ω-6 to ω-3 ratio intake was associated with a higher risk of T2D. Also found positive associations between the ratios of total fat to ω-3	Findings indicate that diets with high cholesterol, monounsaturated, polyunsaturated and ω-3 fatty acids are associated with a lower risk of T2DM. Also the ratios of ω-6/ω-3 and total fat/ω-3 were positively associated with T2DM.	Moderate

Nanri A et al, 2011	Cohort prospective study	22,921 men and 29,759 women aged 45-75 y	Participants completed a questionnaire of the second survey for the Japan Public Health Center-based Prospective Study	5-y period	Prospectively investigated the association between fish intake and type 2 diabetes risk in Japanese adults	971 new cases (572 men and 399 women) of type 2 diabetes were self-reported. In men, fish intake was significantly associated with a decreased risk of type 2 diabetes; In women, fish intake was not appreciably associated with type 2 diabetes risk.	In a population with high fish and seafood intake, fish consumption was associated with a lower risk of type 2 diabetes in men but not in women.	Moderate
Díaz-Rizzolo et al, 2021	RCT	152 subjects with fasting glucose between 100-124 mg/dL aged ≥65 yo randomly distributed among control group (CG, n=77) and sardine group (SG, n=75).	Both groups received same T2D-prevention nutritional during a year but only SG had to add 200 g of sardine per week.	1 years	The study hypothesized that the consumption of twice a week of sardine, during one year would reduce T2D-developing risk in a population with prediabetes (preDM) and old age.	Subjects in SG, significantly compared to CG, decreased percentage classified-individuals in a very high-risk group to develop T2D (p=0.035). SG showed a lower HOMA-IR (p=0.032).	A year T2D-prevention diet with sardine supplementation has a greater protective effect against developing T2D and CV events	High

Rajabi-Naeeni M et al, 2020	RCT	168 women of reproductive age with prediabetes and hypovitaminosis D, assigned to 4 groups.	Placebo group (omega-3 and vitamin D placebos);<br>the omega-3 group (omega-3 supplements and vitamin D placebos);<br>the vitamin D group (omega-3 placebos and vitamin D supplements);<br>co-supplementation group.<br>The groups received every 2 weeks 50,000 IU pearls of vitamin D and twice-daily doses of 1000-mg omega-3 tablets or placebos.	8 weeks	Determine the effectiveness of vitamin D and omega-3 co-supplementation on glycemic control and serum lipid profiles in women of reproductive age with prediabetes and hypovitaminosis D.	A significant reduction was observed in fasting glucose, insulin, homeostasis model assessment-beta cell function, weight and waist circumference in the co-supplementation group compared to the other three groups ($P < 0.05$).	Vitamin D and omega-3 co-supplementation improved fasting serum glucose, insulin, high-density lipoprotein-cholesterol level, homeostasis model assessment-beta cell function, weight and waist circumference in women of reproductive age with prediabetes and hypovitaminosis D.	High
Weta I W et al, 2017	RCT	45 young obese women randomized into two group	22 participants were supplemented with 2000mg and 1000mg of LA and ALA (Intervention), and 23 participants were given placebo (Control)	12 week	Elucidate the effect of the linoleic acid (LA) and alfa linolenic acid (ALA) supplementation to fasting plasma glucose in young women obese patients.	In the Intervention group no significant change in fasting plasma glucose, contrary it increased ($p=0.007$) in the Control group. The impaired plasma glucose increased in the Control group ($OR=8$, $p=0.039$), but not in the Intervention group ($p=0.508$).	Restriction energy intake with supplementation 2000mg and 1000mg of LA and ALA control pre diabetes of young obese women.	High

Jamilian M et al, 2018	Randomized, double-blinded, placebo-controlled clinical trial	60 subjects, aged 18-40 years old with PCOS	50,000 IU vitamin D every 2 weeks plus 2000 mg/day omega-3 fatty acid from fish oil (n = 30)<br>or<br>placebo (n = 30)	12 weeks	Evaluate the effect of the co-administration of vitamin D and omega-3 fatty acid on clinical, metabolic and genetic parameters in women with polycystic ovary syndrome (PCOS)	Vitamin D and omega-3 fatty acid co-administration significantly decreased serum high-sensitivity C-reactive protein (hs-CRP) (P = 0.001) and malondialdehyde (MDA) levels (P < 0.001), and significantly increased plasma total antioxidant capacity (TAC) levels (P = 0.003) compared with the placebo.<br>Vitamin D and omega-3 fatty acid co-supplementation significantly downregulated gene expression of interleukin-1 (IL-1) (P = 0.03), and upregulated vascular endothelial growth factor (VEGF) (P = 0.004) in PBMCs of subjects with PCOS, when compared with placebo	The co-administration of vitamin D and omega-3 fatty acid for 12 weeks had beneficial effects on mental health parameters, serum total testosterone, hs-CRP, plasma TAC and MDA levels, and gene expression of IL-1 and VEGF among women with PCOS.	High

Ibsen D B et al, 2019	Cohort Study	A cohort of 53,163 participants from the Danish Diet, Cancer and Health study were followed for incident type 2 diabetes	Diet was assessed by a validated 192-item food frequency questionnaire at baseline.	Median follow-up time 15.4 years	It was examined the associations between substitution of red meat (total, processed and unprocessed, low fat and high fat) with poultry or fish and substitution of processed red meat with unprocessed red meat and the risk of type 2 diabetes.	6879 cases. Replacing total red meat with fish was associated with a lower risk of type 2 diabetes as was replacement of processed red meat with poultry or fish. Replacing low fat red meat or high fat red meat with fish was associated with a lower risk of type 2 diabetes whereas similar substitutions, with poultry, were not. Replacing processed red meat with unprocessed red meat was also associated with a lower risk of type 2 diabetes	Replacing processed red meat with poultry, replacing total or processed red meat with fish, and replacing processed red meat with unprocessed red meat were all associated with a lower risk of type 2 diabetes.	Moderate
Chen G.C. et al, 2021	Prospective cohort study	392,287 middle-aged and older participants (55.0% women) in the UK Biobank who were free of diabetes, major cardiovascular disease, and cancer	163,706 participated in one to five rounds of 24-h dietary recalls during 2009–2012	Median 10.1 years of follow-up	Evaluate associations of oily and nonoily fish consumption and fish oil supplements with incident type 2 diabetes	7,262 incident cases of T2D were identified.	Consumption of oily fish but not nonoily fish was associated with a lower risk of T2D. Use of fish oil supplements, especially constant use over time, was also associated with a lower risk of T2D	Moderate
Bartimoccia, S et al, 2022	Clinical trial	20 patients with impaired fasting glucose (IFG) and 20 healthy subjects (HS) matched for sex and age.	Variables were measured before and after a Mediterranean diet with 10 g EVOO added or not or in IFG patients before and after intake of 40 g chocolate with		Was tested the hypothesis that EVOO improves post-prandial glycemia by reducing gut permeability-derived low-grade endotoxemia.	IFG had higher levels of LPS and zonulin. Two hours after a meal intake containing EVOO, IFG patients showed a less significant increase of blood glucose, a more marked	Addition of EVOO to a Mediterranean diet or chocolate improves gut permeability and low-grade endotoxemia.	High

			EVOO added or not.			increase of blood insulin and GLP1 and a significant reduction of LPS and zonulin compared to IFG patients not given EVOO.		
Njike, V.Y. et al, 2021	RCT cross-over	20 adults (mean age 56.1 years; 10 women, 10 men) at risk for T2DM	Subjects were assigned to one of two possible sequence permutations of two different single dose treatments (50 mL of high-polyphenolic EVOO or 50 mL of refined olive oil without polyphenols), with 1-week washout.		To compare the effects of high-polyphenolic extra virgin olive oil (EVOO) and refined olive oil without polyphenols on endothelial function (EF) in adults at risk for Type 2 diabetes mellitus (T2DM)	EVOO acutely improved EF as compared to refined olive oil (1.2 ± 6.5% versus −3.6 ± 3.8%; p = 0.0086).	High-polyphenolic EVOO acutely enhanced EF in the study cohort, whereas refined olive oil did not.	High
Carnevale, R et al, 2017	RCT	30 IFG patients	Patients were randomly allocated to a meal containing or not 10 g of EVOO in a cross-over design.	6 months	Was investigated if EVOO affects post-prandial glucose and lipid profile in patients with impaired fasting glucose (IFG)	The meal containing EVOO was associated with a reduction of glucose (p = 0.009) and DPP4 activity (p < 0.001) and a significant increase of insulin (p < 0.001) and GLP-1 (p < 0.001) compared with the meal without EVOO.	in IFG patients EVOO improves post-prandial glucose and lipid profile with a mechanism probably related to incretin up-regulation	High

Review and Meta-analysis

Authors	Type of study	Number of studies	Subjects (total)	End point	Result	Conclusion	Strenght of evidence
Xun P et al, 2012	Meta-analysis	9 eligible studies	12 independent cohorts - 438,214 individuals	Assess the literature and determine the association between fish consumption and diabetes risk quantitatively.	Compared with those who never consumed fish or ate fish less than once per month, the pooled RR of incident diabetes was 0.99 (95% CI 0.85–1.16) for individuals who ate fish five or more times per week (P trend = 0.80). Similar results were found for long-chain n-3 polyunsaturated fatty acid intake.	Evidence generated from this meta-analysis does not support an overall inverse association of fish or fish oil intake with incidence of diabetes.	High
Schwingshackl, L et al, 2017	Systematic Review and Meta-analysis	4 cohort studies<br>29 trials included in the metanalysis	15784 T2D	Illustrate the impact of Olive Oil (OO) on type 2 diabetes (T2D) by investigating the association between OO intake and risk of T2D, and the effect of OO intake in the management of T2D	The highest OO intake category showed a 16% reduced risk of T2D. In T2D patients OO supplementation resulted in a significantly more pronounced reduction in HbA1c	The intake of OO could be beneficial for the prevention and management of T2D.	High
