# Supplementary material for: A Narrative Review on Strategies for the Reversion of Prediabetes to Normoglycemia: Food Pyramid, Physical Activity, and Self-Monitoring Innovative Glucose Devices
Source: Nutrients. 2023 Nov 28;15(23):4943. doi: 10.3390/nu15234943 (PMC10707766; doi:10.3390/nu15234943)
Supplement: Supplementary file 1 [file nutrients-15-04943-s001.zip › Tables Prediabetes Nutrients/5 Tabelle Vitamins.pdf]

Authors	Type of study	Population characteristics	Type of intervention	Duration	End point	Results	Conclusion	Strength of evidence
Banerjee et al, 2017	cross-sectional study	202 subjects: 77 subjects with type 2 diabetes (mean age 48.09±6.8), 73 with prediabetes (mean age 49.96±7.6) and 52 healthy subjects constituting the control group (mean age 50.08±7.1).	All subjects were matched for age, gender, and BMI within study groups. In all study groups, fasting serum levels of adiponectin, insulin and 25(OH)D were measured and routine biochemical parameters were analysed.		Investigate the roles of deficient or deranged insulin, adiponectin and 25 hydroxy vitamin D (25[OH]D) levels and to establish their interrelationship	A statistically significant lower levels of serum adiponectin and serum 25(OH)D and higher serum insulin levels in prediabete or type 2 diabetes. The changes in the serum adiponectin or serum 25(OH)D in prediabete and type 2 diabete were inversely correlated with the serum levels of insulin.	The association of these hormones might act as a significant predictor of progression of prediabetes to type 2 diabetes.	Moderate
Rasouli et al, 2022	RCT	1774 subjects with a mean age of 60.5 ± 9.8 years and a mean BMI of 31.9 ± 4.4 kg/m2 (44% female and 69% white). Participants had to meet at least 2 of 3 glycemic criteria for prediabetes defined by the 2010 American Diabetes Association guidelines.	Overweight/obese adults at high risk for type 2 diabetes (prediabetes) were randomly treated with vitamin D3 4000 IU or matching placebo daily. Disposition index (DI), as an estimate of β-cell function, was calculated as the product of HOMA2%Scpep and C-peptide response during the first 30 minutes of a 75-g oral glucose tolerance test (OGTT).	24 months	Investigate the effects of **vitamin D3** supplementation on insulin sensitivity and β-cell function in adults at high risk for type 2 diabetes	In the entire cohort, there were no significant differences in changes in DI, HOMA2%Scpep, or C-peptide response between the 2 groups. vitamin D improved β-cell function among those who had baseline 25(OH)D levels below 12 ng/mL.	Supplementation with vitamin D3 for 24 months did not improve an OGTT-derived index of β-cell function in people with prediabetes not selected based on baseline vitamin D status; however, there was benefit among those with very low baseline vitamin D status.	High
Jie Zhu et al, 2020	Prospective cohort study	4704 American adults (52% female and 51% black) between 18 and 30 years of age (mean age, 24.9 ± 3.6 years) and without diabetes enrolled in 1985-1986 and monitored through 2015-2016 in the Coronary Artery Risk Development	Dietary assessment conducted by means of a validated anamnestic questionnaire at baseline, year 7 and year 20.	30 years	Prospectively examine intakes of folate, vitamin B6, and vitamin B12 in relation to diabetes incidence in a large U.S. cohort.	During 30 years of follow-up, 655 incident cases of diabetes occurred. Intake of folate was inversely associated with diabetes incidence after adjustment for potential confounders. Higher folate intake was also associated with lower plasma homocysteine (P trend <	Intake of folate in young adulthood was inversely associated with diabetes incidence in midlife among Americans.	Moderate

		in Young Adults (CARDIA) study.				0.01) and insulin (P trend < 0.01).		
Jin G et al, 2021	Cross-sectional study	22,041 participants (10,672 men and 11,369 women) over the age of 20.	5 diagnostic criteria (3 based on laboratory data and 2 on questionnaires) were applied to define the condition of diabetes or prediabetes. It was used a 24-hour recalls of two different days		Define the association between folate, B12 and B6 obtained from diet and supplementation and diabetes and prediabetes in American adults	Of the 22,041 participants, 18.3% had diabetes and most were over 60 years old. Dietary folate and B6 were associated with a lower risk of diabetes, and after adjusting for confounders, folate, B6, and B12 levels were inversely associated with diabetes. Dietary folate and B6 intakes were negatively associated with new diabetes diagnoses, and B12 and B6 were inversely associated with prediabetes.	There is an association between low values of B vitamins and the risk of diabetes, especially in the population over 60 years of age	Moderate
Wilson et al, 2017	Cross-sectional study	89 participants, including individuals with normal glucose tolerance (n = 35), prediabetes (n = 25), and DM2 managed with diet alone or a metformin-only regimen (n = 29).	Participants completed a 4-day food diary.		Analyzing plasma vitamin C concentrations across the glycemic spectrum and investigating the correlation with indices of metabolic health in adults with glycemic conditions ranging from normal glucose tolerance (NGT) to those with DM2.	Vitamin C plasma concentrations were significantly lower in subjects with DM2 than in those with NGT (41.2 µmol/L versus 57.4 µmol/L, $p < 0.05$), and a higher rate of deficiency was observed of vitamin C (i.e. <11.0 µmol/L) in both the prediabetic and DM2 groups. Fasting glucose, BMI, smoking history and dietary vitamin C intake are predictors significant independents of plasma vitamin C concentrations.	These results suggest that adults with a history of smoking, prediabetes or DM2, and/or obesity have higher vitamin C requirements.	Moderate

Review and Meta-analysis

**Authors**	**Type of study**	**Number of studies**	**Subjects (total)**	**End point**	**Result**	**Conclusion**	**Strenght of evidence**
Zhang Y et al, 2020	Systematic review and meta-analysis	8 eligible studies	Total of 4,896 subjects	Evaluate whether vitamin D supplementation reduces the risk of developing type 2 diabetes in patients with prediabetes.	All 8 studies reported the development of new-onset diabetes, particularly in 1,022 (20.9%) of 4,896 participants. Combining data from all 8 studies, vitamin D supplementation reduced the incidence of new-onset diabetes by 11%. Reversion of prediabetes to normoglycemia occurred in 116 of 548 (21.2%) participants in the vitamin D group and 75 of 532 (14.1%) participants in the control group.	In people with prediabetes, vitamin D supplementation reduces the risk of T2DM and increases the rate of reversion of prediabetes to normo-glycemia.	High
Lind MV and al, 2019	Meta-analysis of RCTs	29 studies (4 with crossover design and 25 with parallel design),	Total of 22,250 participants	Investigate the effects of folate supplementation on the outcome of insulin resistance and diabetes, evaluating the effect of placebo-controlled folate supplementation, alone or in combination with other B vitamins, on fasting glucose, insulin, HOMA-IR, HbA1c or risk of type 2 diabetes.	Compared with placebo, folate supplementation lowered fasting insulin and HOMA-IR, but no overall effects were observed for fasting glucose or HbA1c. Changes in homocysteine after folate supplementation correlated with changes in fasting glucose and HbA1c.	Folate supplementation might be beneficial for glucose homeostasis and lowering IR, but at present there are insufficient data to conclusively determine the effect on development of T2D.	High
Ashor AW and al, 2017	Systematic review and	22 studies (16 with parallel	total of 937 participants	Test the effect of vitamin C administration on glucose,	Vitamin C did not change glucose, insulin and HbA1c	The greatest reduction in glucose concentrations was	High

	meta-analysis of RCTs	design and 6 with crossover design)		HbA1c and insulin concentrations and on insulin sensitivity.	concentrations. Subgroup analyzes showed that vitamin C significantly decreased glucose concentrations in patients with DM2. The analyzes showed a better benefit of vitamin C administration on glycemia in those with a higher baseline BMI and glucose concentration, with the greatest effects in the longer duration studies. Positive effects were observed on fasting insulin concentration values.	observed in patients with diabetes, older individuals, and with more prolonged supplementation	
Xu R and al, 2014	Meta-analysis of RCTs	14 RCTs (12 evaluated the effects on HbA1c, 12 on fasting glucose, 6 on fasting insulin)	714 subjects (363 for the vitamin E group and 351 for the control group).	Characterise vitamin E impact (range of vitamin E administered 200-1,600 IU/day, taken from 6 to 27 weeks) on HbA1c, fasting glucose and fasting insulin.	Vitamin E supplementation did not lead to significant benefits in glycemic control, but subgroup analyzes revealed significant reductions in HbA1c and fasting insulin versus controls in patients with low baseline vitamin E status.	There is insufficient evidence supporting a potential beneficial effect of vitamin E supplementation on improving HbA1c and fasting glucose and insulin concentrations in subjects with prediabetes.	High
