# Supplementary material for: A Narrative Review on Strategies for the Reversion of Prediabetes to Normoglycemia: Food Pyramid, Physical Activity, and Self-Monitoring Innovative Glucose Devices
Source: Nutrients. 2023 Nov 28;15(23):4943. doi: 10.3390/nu15234943 (PMC10707766; doi:10.3390/nu15234943)
Supplement: Supplementary file 1 [file nutrients-15-04943-s001.zip › Tables Prediabetes Nutrients/6 Tabella Minerals.pdf]

Authors	Type of study	Population characteristics	Type of intervention	Duration	End point	Results	Conclusion	Strength of evidence
Capdor, J. et al, 2013	Meta-analysis	3978 subjects were included in the meta-analysis	A systematic review and meta-analysis of randomised placebo controlled trials was conducted		to determine the effect of zinc supplementation on fasting blood glucose, HbA1c, serum insulin and serum zinc concentrations.	a small but statistically significant reduction in fasting glucose concentrations was observed (-0.19±0.08mmol/L, P=0.013) after zinc supplementation. HbA1c tended to decrease in zinc-supplemented individuals (-0.64±0.36%, P=0.072). No significant effect was observed for serum insulin concentrations.	The significant albeit modest reduction in glucose concentrations and tendency for a decrease in HbA1c following zinc supplementation suggest that zinc may contribute to the management of hyperglycemia in individuals with chronic metabolic disease	High
Wang X. et al. 2019	Meta-analysis	Thirty-two placebo-controlled interventions were extracted from 36 publications, involving a total of 1700 participants in 14 countries.	zinc supplementation		to assess the effects of zinc supplementation in preventing and managing diabetes	the subjects in the zinc-supplementation group had a statistically significant reduction in fasting glucose [FG, weighted mean difference (WMD): -14.15 mg/dL; 95% CI: -17.36, -10.93 mg/dL], 2-h postprandial glucose (WMD: -36.85 mg/dL; 95% CI: -62.05, -11.65 mg/dL), fasting insulin (WMD: -1.82 mU/L; 95% CI: -	several key glycemic indicators are significantly reduced by zinc supplementation, particularly the FG in subjects with diabetes and in subjects who received an inorganic zinc supplement.	High

						3.10, -0.54 mU/L), homeostasis model assessment for insulin resistance (WMD: -0.73; 95% CI: -1.22, -0.24), glycated hemoglobin (WMD: -0.55%; 95% CI: -0.84, -0.27%), and high-sensitivity C-reactive protein (WMD: -1.31 mg/L; 95% CI: -2.05, -0.56 mg/L) concentrations.		
Zhou, Q. et al, 2019	Cross-sectional Study	patients with IFG (n = 12), IGT (n = 15), T1D (n = 25), T2D (n = 137), and healthy controls (n = 50)	Se was detected using inductively coupled plasma spectrometer.	/	This study investigated serum and urinary Se levels in patients with impaired fasting glucose (IFG), impaired glucose tolerance (IGT), type 1 diabetes (T1D), and type 2 diabetes (T2D) in Northeast Chinese populations.	The serum Se level was dramatically lower in patients with T1D and was significantly higher in IFG subjects, and the urinary Se concentration was markedly lower in IGT and T2D groups. The serum Se levels were positively correlated with serum zinc (Zn) in both IFG and IGT groups, while urinary Se were positively associated with urinary Zn and copper (Cu) in IGT group.	The serum Se levels were positively correlated with serum Cu in both T1D and T2D groups, and urinary levels of Se were positively associated with serum zinc and urinary Cu, Zn, calcium (Ca), and magnesium (Mg) and negatively correlated with serum Ca and Mg in T2D group, while the urinary levels of Se were positively correlated with urinary Zn and Mg both in peripheral neuropathy (DPN) and retinopathy (DR) groups	Moderate

Xu, J. et al, 2013	Cross-sectional study	patients with type 1 diabetes (T1D, n = 25), type 2 diabetes (T2D, n = 137), impaired fasting glucose (IFG, n = 12) or impaired glucose tolerance (IGT, n = 15), and age/gender matched controls (n = 50)		/	the association of copper and zinc levels in the serum or urine of patients living in northeast China, with either prediabetes or diabetes	Serum copper levels were significantly higher in IFG, IGT, and T2D groups. Serum zinc level was dramatically lower, and urinary zinc level was significantly higher in both T1D and T2D subjects compared with controls.	Simvastatin treatment in T2D patients had no significant effect on serum and urinary copper and zinc. These results suggest the need for further studies of the potential impact of the imbalanced serum copper and zinc levels on metabolic syndrome, diabetes, and diabetic complications.	Moderate
Hruby, A. et al, 2014	Prospective cohort study	2,582 community-dwelling participants 26-81 years old at baseline		7 years	To assess 7-year associations between magnesium intake and incident prediabetes and/or insulin resistance (IR), and progression from these states to type 2 diabetes	Higher magnesium intake tended to associate with lower follow-up FG and IR, but not fasting insulin, postload values, or insulin sensitivity.	Magnesium intake may be particularly beneficial in offsetting risk of developing diabetes among those at high risk. Magnesium's long-term associations with non-steady-state (dynamic) measures deserve further research	Moderate
Kieboom, B.C.T. et al, 2017	Retrospective cohort study	8555 participants (mean age, 64.7 years; median follow-up, 5.7 years) with normal glucose levels (mean ± SD: 5.46 ± 0.58 mmol/l) at baseline			study the directionality of the association between serum magnesium levels and diabetes	A 0.1 mmol/l decrease in serum magnesium level was associated with an increase in diabetes risk (HR 1.18 [95% CI 1.04, 1.33]); similar association was found between serum magnesium levels and prediabetes risk (HR	Low serum magnesium levels are associated with an increased risk of prediabetes and this increased risk is similar to that of diabetes.	Moderate

						1.12 [95% CI 1.01, 1.25])		
Guerrero-Romero, F. et al, 2008	Prospective cohort study	1122 individuals (20-65 years of age) were enrolled between 1996 and 1997, and 817 individuals re-examined about 10 years later	ew-onset IFG (5.6-7.0 mmol L(-1) fasting glucose), IGT (7.8-11.1 mmol L(-1) glucose 2-h postload), and type 2 diabetes were determined from the number of subjects who had these conditions at the second examination without evidence that they were present at the first one.	10 years	to examine the association between serum magnesium levels and the risk for developing IFG, IGT and type 2 diabetes	New-onset IFG and IGT was identified in 276 (33.8%) individuals. The relative risk for IFG, IGT and IFG + IGT was 1.11 (95% confidence interval, 0.5-5.1), 1.38 (95% confidence interval, 1.1-6.3) and 1.49 (95% confidence interval, 1.1-4.9), respectively. New-onset diabetes was identified in 78 (9.5%) individuals (relative risk 2.54; 95% confidence interval, 1.1-4.1).	Hypomagnesaemia is independently associated with the development of IGT, IFG + IGT and type 2 diabetes, but not with the development of IFG	Moderate
Wu, F. et al, 2019	Prospective cohort study	1134 subjects; age 3 to 18 years at baseline	Dietary calcium intake was assessed at baseline (1980) and adult follow-up visits (2001, 2007, and 2011). Long-term (mean between youth and adulthood) dietary calcium intake was	31-year	examine whether youth and long-term (between youth and adulthood) dietary calcium intake is associated with adult impaired glucose metabolism and type 2 diabetes (T2D).	no evidence for nonlinear associations between calcium intake and IFG or T2D among females and males (all P for nonlinearity > 0.05). Higher youth and long-term dietary calcium intake was not associated with	Youth or long-term dietary calcium intake is not associated with adult risk of developing impaired glucose metabolism or T2D	Moderate

			calculated.			the risk of IFG or T2D among females or males		
Pittas, A.G. et al, 2007	Double blinded randomized controlled trial	314 Caucasian adults	ceived either 500 mg calcium citrate and 700 IU vitamin D(3) or placebos daily for 3 years	3 years	compare the effects of combined calcium and vitamin D supplementation versus placebo on blood glucose and markers of inflammation in nondiabetic adults aged > or =65 years.	e effects of combined calcium-vitamin D supplementation on 3-year change in FPG depended on baseline FPG (P = 0.02 for interaction). Among participants with IFG at baseline, those who took combined calcium-vitamin D supplements had a lower rise in FPG at 3 years compared with those on placebo (0.02 mmol/l [0.4 mg/dl] vs. 0.34 mmol/l [6.1 mg/dl], respectively, P = 0.042) and a lower increase in HOMA-IR (0.05 vs. 0.91, P = 0.031).	In healthy, older adults with IFG, supplementation with calcium and vitamin D may attenuate increases in glycemia and insulin resistance that occur over time.	High

Alissa, E.M. et al, 2009	Cross-sectional study	130 Saudi men with an established history of myocardial infarction and 130 age-matched controls without established CVD	measured serum and urine chromium concentrations, fasted lipid profile, plasma glucose, and serum lipid peroxide		to investigate chromium status among Saudi men with and without established cardiovascular disease (CVD) and its relationship to glucose tolerance, lipid profile and other established CVD risk factors	tients with established CVD had higher serum triglycerides ($p < 0.05$) and plasma glucose ($p < 0.0001$) and lower serum and urinary chromium concentrations ($p < 0.0001$) than controls. Serum chromium was inversely correlated with plasma glucose among cases and controls ($r = -0.189$, $p < 0.05$ and $r = -0.354$, $p < 0.00001$, respectively).	While chromium metabolism appears to be altered in individuals with CVD, it is unclear whether chromium supplementation would be effective in CVD prevention among patients with IGT.	Moderate
Althuis, M.D. et al, 2002	Meta-analysis	This review summarizes data on 618 participants from the 15 trials			to determine the effect of chromium on glucose and insulin responses in healthy subjects and in individuals with glucose intolerance or type 2 diabetes	The meta-analysis showed no association between chromium and glucose or insulin concentrations among nondiabetic subjects. Three trials reported data on Hb A(1c): one study each of persons with type 2 diabetes, persons with impaired glucose tolerance, and healthy subjects	Data from RCTs show no effect of chromium on glucose or insulin concentrations in nondiabetic subjects. The data for persons with diabetes are inconclusive.	High

Ali, A. et al, 2011	Double blind randomized controlled trial	59 adult with IFG	randomized, double-blind, placebo-controlled, modified cross-over clinical trial. Participants received 6-month sequences of chromium picolinate or placebo at 1 of 2 dosages (500 or 1000 mcg daily).	6 months	To investigate the effects of daily chromium picolinate supplementation on serum measures of glucose tolerance and insulin sensitivity in patients at high risk for type 2 diabetes mellitus	No changes were seen in glucose level, insulin level, or HOMA-IR (all P>.05) after 6 months of chromium at either dosage level (500 mcg or 1000 mcg daily) when compared with placebo.	Chromium supplementation does not appear to ameliorate insulin resistance or impaired glucose metabolism in patients at risk for type 2 diabetes and thus is unlikely to attenuate diabetes risk.	High
Wang, Z.Q. et al, 2010	Narrative review				Chromium Supplementation in Type 2 Diabetes and Insulin Resistance	a consistent significant and beneficial effect of chromium may not be observed. Specifically, recent data fail to demonstrate significant improvement in carbohydrate metabolism in individuals with metabolic syndrome, impaired glucose tolerance, or consistently in individuals with type 2 diabetes.		Low
