# Supplementary material for: A Narrative Review on Strategies for the Reversion of Prediabetes to Normoglycemia: Food Pyramid, Physical Activity, and Self-Monitoring Innovative Glucose Devices
Source: Nutrients. 2023 Nov 28;15(23):4943. doi: 10.3390/nu15234943 (PMC10707766; doi:10.3390/nu15234943)
Supplement: Supplementary file 1 [file nutrients-15-04943-s001.zip › Tables Prediabetes Nutrients/7 Tabelle Osteoporosis.pdf]

Authors	Type of study	Population characteristics	Type of intervention	Duration	End point	Results	Conclusion	Strength of evidence
Liu Y et al, 2023	Multistage cross-sectional study	A total of 23,825 subjects (7,427 with prediabetic).	Data from the U.S. National Health and Nutrition Examination Surveys. Bone mineral density and the skeletal muscle mass index (SMI) were measured with dual-energy X-ray absorptiometry (DXA).	Data from 2009 to 2018	Investigate the effect of sarcopenia, osteoporosis and osteosarcopenia on spine fracture in patients with prediabetes.	Regarding bone health, the lumbar and spinal bone mineral density of the prediabetes group was lower than in the healthy group, while there were no differences between diabetic and healthy subjects. The prevalence of osteoporosis was higher in diabetics than in prediabetics, and higher in the latter than in healthy subjects.	Osteoporosis is a risk factor for spine fracture in prediabetic adults and the combination of sarcopenia and osteoporosis further increases the prevalence of spine fracture.	Moderate
