# Supplementary material for: A Narrative Review on Strategies for the Reversion of Prediabetes to Normoglycemia: Food Pyramid, Physical Activity, and Self-Monitoring Innovative Glucose Devices
Source: Nutrients. 2023 Nov 28;15(23):4943. doi: 10.3390/nu15234943 (PMC10707766; doi:10.3390/nu15234943)
Supplement: Supplementary file 1 [file nutrients-15-04943-s001.zip › Tables Prediabetes Nutrients/8 Tabella Fruits and vegetables.pdf]

Authors	Type of study	Population characteristics	Type of intervention	Duration	End point	Results	Conclusion	Strength of evidence
Barouti et al, 2022	Cohort prospective study	6961 men and women, aged between 35 and 56	Questionnaire for diet assesment	20 years	To establish a relationship between consumption of fruit and vegetables and the development of disglycemia	An inverse relationship was seen between fruit and vegetable consumption and the development of a disglycemia	Increasing fruits and vegetables in the diet reduces the risk of disglicemia (HR: 0,86)	High
Li et al, 2023	Cohort prospective study	79,922 patients aged over 40 years	Questionnaire for diet assesment	4 years	To establish a relationship between consumption of fruit and vegetables and the development of disglycemia	The risk of development of disglycemia decreased especially in normoglycemic patients in whom the consumption of more than 7 portions of fruit per week decreased the risk of developing diabetes by 48%.	Increasing fruits and vegetables in the diet reduces the risk of disglycemia	High
Wu et al, 2021	Cross-sectional study	6802 participants between 18 and 65 years old	Questionnaire for diet assesment		To establish a relationship between diet and the development of DM2 or IFG	A reduction in the risk of prediabetes was seen with a consumption of fruit and vegetables corresponding to the third and fourth quartile, i.e. between 320 and 530 grams (vegetables + fruit/day).	Increasing fruits and vegetables in the diet reduces the risk of disglycemia	High

Zhang et al, 2022	Cohort prospective study	18,085 participants who at baseline had neither diabetes nor prediabetes or other CVD nor cancer.	Questionnaire for diet assesment	63175 person-years	To establish a relationship between the consumption of fibre and DM2 or IFG	4139 cases of diabetes occurred.	Fiber intake was inversely related to the incidence of prediabetes. The type of fiber that was found to be most effective in preventing prediabetes is soluble fiber, mostly found in fruit and vegetables.	High
Lopez Ridarura et al, 2004	Cohort prospective study	85,060 women and 42,872 men	Magnesium intake was evaluated using a validated food frequency questionnaire every 2-4 years.	18 years	To establish if magnesium intake could reduce the risk of developing diabetes mellitus	Magnesium reduces the risk of diabetes	The results of this study confirmed the inverse association between magnesi-um intake and diabetes risk	High

Review and meta-analysis

Authors	Type of study	Number of studies	Subjects (total)	End point	Result	Conclusion	Strenght of evidence
Min Li et al, 2014	Meta-analysis and systematic review of prospective cohort studies	A total of 10 articles including 13 comparisons with 24,013 cases of type 2 diabetes.	434,342 participants	To assess the relationship between fruit and vegetable consumption and the risk of developing diabetes	Evidence of curve linear associations was seen between fruit and green leafy vegetables consumption and risk of type 2 diabetes.	Higher fruit or green leafy vegetables intake is associated with a significantly reduced risk of type 2 diabetes.	HIGH
