# Supplementary material for: A Narrative Review on Strategies for the Reversion of Prediabetes to Normoglycemia: Food Pyramid, Physical Activity, and Self-Monitoring Innovative Glucose Devices
Source: Nutrients. 2023 Nov 28;15(23):4943. doi: 10.3390/nu15234943 (PMC10707766; doi:10.3390/nu15234943)
Supplement: Supplementary file 1 [file nutrients-15-04943-s001.zip › Tables Prediabetes Nutrients/9 Tabella Nuts.pdf]

**Authors**	**Type of study**	**Population characteristics**	**Type of intervention**	**Duration**	**End point**	**Results**	**Conclusion**	**Strength of evidence**
Gulati et al, 2022	RCT with two parallel groups	66 participants with prediabetes in the range of 18-60 years	The effects of a dose of 20g of almonds before lunch and dinner was evaluated	3 months	To evaluate the risk of prediabetes and DM2	The intake of almonds before main meals would therefore seem to help in the prevention of the evolution from prediabetes to frank diabetes.	Eating almonds before meals can help reduce the risk of DM2 and IFG	High
Cascas et al, 2011	Randomized parallel group study	50 patients with metabolic syndrome were instructed to consume 30g of dried fruit per day	People were instructed to consume 30g of dried fruit per day (15g walnuts, 7.5g almonds, 7.5g hazelnuts)	12-week	To evaluate the effects of nuts in inflammatory markerkers and HOMA index	The nut group reduced fasting insulin by 2.60 µU/mL (95% CI, -4.62 to -0.59) and HOMA by 0.72 (-1.28 to -0.16) ($P<0.05$) . Among inflammatory markers, IL6 levels decreased by 1.1 ng/ml	Eating nuts seems to reduce HOMA index and inflammatory markers	High
Hou et al, 2018	RCT	32 patients with DM2	60 g/day peanuts for men and 50 g/day for women in the Peanut group, and 55 g/day almonds for men and 45 g/day for women in the Almond group	3-month	To evaluate the relationship with peanuts and almonds in the diet and BMI and glicometabolic patterns	There were significant pre-post changes in fasting and postprandial blood glucose ($p < 0.05$). The glycated value was reduced in the almond group ($p < 0.05$).	Introducing in the diet peanuts and almonds can improve glicometabolic patterns	High

Wien et al, 2010	Randomized parallel group study	65 people with prediabetes	Almond intake was approximately 20% of the daily caloric intake, approximately 60g/day.	16 weeks	To evaluate the relationship with almonds in the diet and BMI and glicometabolic patterns	The intervention group showed both a reduction in insulin HOMA IR and HOMA 2B compared with the control group without almonds.	Introducing in the diet almonds can improve glicometabolic patterns and can help reduces insulin resistance	High
Lu et al, 2021	Randomized crossover study	Ten overweight Chinese men with a mean age of 47.9 years	Ingestion of a nut-based, high-protein snack bar or an isocaloric bar contain more carbohydrates. The bar used contained a caloric quantity of about 1000 KJ.	0-120 min	Evaluate the glicemic response in a 2h window with two different bars	In the patients who received the bar rich in dried fruit, there was a reduction in blood sugar ($P < 0{\cdot}05$) in the 30-120 minutes after ingestion of both the bar alone and the bar associated with 50g of white bread (a food that normally raises blood sugar), with an area under the glycemic curve 10 times lower.	The nuts bar can reduce post prandial glycemia	High
